# Supplementary material for: Improved Diazo-Transfer Reaction for DNA-Encoded Chemistry and Its Potential Application for Macrocyclic DEL-Libraries
Source: Molecules. 2021 Mar 22;26(6):1790. doi: 10.3390/molecules26061790 (PMC8004608; doi:10.3390/molecules26061790)
Supplement: Supplementary file 1 [file molecules-26-01790-s001.zip › Supporting Information_revised.pdf]

Supporting Information

New Improved Diazo-Transfer Reaction for DNA-Encoded Chemistry and their Potential Application for Macrocyclic DEL-Libraries

Selahattin Ede[1,3], Mandy Schenk,[1,3] Donald Bierer,[2] Hilmar Weinmann[1†] and Keith Graham[1,3*§]

1 Bayer AG, Innovation Campus Berlin, 13353 Berlin, Germany
2 Bayer AG, Medicinal Chemistry, 42096 Wuppertal, Germany
3 Nuvisan ICB GmbH, 13353 Berlin, Germany
* Correspondence: keith.graham@boehringer-ingelheim.com
† Present address: Janssen Pharmaceuticals, B-2340 Beerse, Belgium
§ Present address: Boehringer-Ingelheim, 55216, Biberach, Germany

Table of Contents

1 Abbreviations ... 4

2 Tables ... 5

3 Materials and Instrumentation ... 6

3.1 General Procedures ... 7

3.2 Example for DNA-Damage during optimisation of Diazo-Transfer Reactions ... 9

3.2.1 6-Azidohexanoic acid conjugated with HP280 (3) ... 9

3.2.2 4-Azidobenzoic acid conjugated with HP280 (4, Table 2 Entry 20) ... 11

3.3 Chromatograms of DNA-conjugated Amino acids and Azides ... 13

3.3.1 6-Azidohexanoic acid conjugated with HP-280 (3, Table 2 Entry 6) ... 13

3.3.2 4-Azidobenzoic acid conjugated with HP-280 (4, Table 2 Entry 20) ... 15

3.3.3 HP-280 Azide (Table 2 Entry 1) ... 17

3.3.4 Azido-Gly-OH conjugated with HP-280 (Table 2 Entry 2) ... 19

3.3.5 Azido-β-Ala-OH conjugated with HP-280 (Table 2 Entry 3) ... 21

3.3.6 Azido-γ-Abu-OH conjugated with HP-280 (Table 2 Entry 4) ... 23

3.3.7 Azido-5-Ava-OH conjugated with HP-280 (Table 2 Entry 5) ... 25

3.3.8 *Azido-Sar*-OH conjugated with HP-280 (Table 2 Entry 7) ... 27

3.3.9 Azido-Ala-OH conjugated with HP-280 (Table 2 Entry 8) ... 29

3.3.10 Azido-α-methylalanine conjugated with HP-280 (Table 2 Entry 9) ... 31

3.3.11 Azidocycloleucine conjugated with HP-280 (Table 2 Entry 10) ... 33

3.3.12 Azido-propargylglycine conjugated with HP-280 (Table 2 Entry 11) ... 35

3.3.13 Azido-Lys(Alloc)-OH conjugated with HP-280 (Table 2 Entry 12) ... 37

3.3.14 Azido-Gln-OH conjugated with HP-280 (Table 2 Entry 13) ... 39

3.3.15 Azido-Phe(4-Br)-OH conjugated with HP-280 (Table 2 Entry 14) ... 41

3.3.16 Azido-Tyr-OH conjugated with HP-280 (Table 2 Entry 15) ... 43

3.3.17 Azido-Phe(4-N-Boc)-OH conjugated with HP-280 (Table 2 Entry 16) ... 45

3.3.18 Boc-Phe(4-azido)-OH conjugated with HP-280 (Table 2 Entry 17) ... 47

3.3.19 Azido-Trp-OH conjugated with HP-280 (Table 2 Entry 18) ... 49

3.3.20 3-(Azidomethyl)benzoic acid conjugated with HP-280 (Table 2 Entry 19) ... 51

3.3.21 Azido-3-Abz-OH conjugated with HP-280 (Table 2 Entry 21) ... 53

3.3.22 Azido-2-Abz-OH conjugated with HP-280 (Table 2 Entry 22) ... 55

3.3.23 Azido-3-Abz(4-F)-OH conjugated with HP-280 (Table 2 Entry 23) ... 57

3.3.24 Azido-3-Abz(6-Cl)-OH conjugated with HP-280 (Table 2 Entry 24) 59
3.3.25 Azido-2-Abz(6-Br)-OH conjugated with HP-280 (Table 2 Entry 25)............................ 61
3.3.26 H-2-Abz(4-Br)-OH conjugated with HP-280 (Table 2 Entry 26) 63
3.3.27 H-3-Abz(3-Br)-OH conjugated with HP-280 (Table 2 Entry 27) 65
3.3.28 Azido-3-Abz(2-Br)-OH conjugated with HP-280 (Table 2 Entry 28)............................ 67
3.3.29 Azido-4-Abz(2-Br)-OH conjugated with HP-280 (Table 2 Entry 29)............................ 69
3.3.30 Azido-4-Abz(3-Br)-OH conjugated with HP-280 (Table 2 Entry 30)............................ 71
3.3.31 4-Azido-Abz(2-I)-OH conjugated with HP-280 (Table 2 Entry 31) 73
3.3.32 5-Azidonicotinic acid conjugated with HP-280 (Table 2 Entry 32) 75
3.3.33 3-Azidopicolinic acid conjugated with HP-280 (Table 2 Entry 33)............................... 77
3.3.34 6-Azidonicotinic acid conjugated with HP-280 (Table 2 Entry 34) 79
3.3.35 2-Azidopyrimidine-5-carboxylic acid conjugated with HP-280 (Table 2 Entry 35) 81
3.3.36 4-Azido-1-*H*-pyrazole-1-acetic acid conjugated with HP-280 (Table 2 Entry 36).......... 83
3.4 Chromatograms of DNA-conjugated Tripeptides and Cyclization 85
3.4.1 [cyclo-3-Abz-Phe-Pra]-OH conjugated with HP-280 (Table 3 Entry 1) 86
3.4.2 [cyclo-2-Abz-Phe-Pra]-OH conjugated with HP-280 (Table 3 Entry 2) 92
3.4.3 [cyclo-β-Ala-Phe-Pra]-OH conjugated with HP-280 (Table 3 Entry 3) 95
3.4.5 [cyclo-β-Ala-Trp-Pra]-OH conjugated with HP-280 (Table 3 Entry 4) 99
3.4.6 [cyclo-Gly-Trp-Pra]-OH conjugated with HP-280 (Table 3 Entry 5) 103
3.4.7 [cyclo-γ-Abu-Trp-Pra]-OH conjugated with HP-280 (Table 3 Entry 6) 108
3.4.8 [cyclo-βAla-βAla-Pra]-OH conjugated with HP-280 (Table 3 Entry 7)........................ 113
3.4.9 [cyclo-γ-Abu-β-Ala-Pra]-OH conjugated with HP-280 (Table 3 Entry 8) 118
3.4.10 [cyclo-3-Abz-β-Ala-Pra]-OH conjugated with HP-280 (Table 3 Entry 9) 123
3.5 Methods and NMRs of Off-DNA Compounds (8-19) 128
3.5.1 Azido-Gly-Phe-Pra-NH_2 (12).. 128
3.5.2 Cyclo-1,4-Triazol[-β-Ala-Phe-Pra]-NH_2 (17) 134
3.5.3 Cyclo-1,4-Triazol[-2-Abz-Phe-Pra]-OH (18) 143
3.5.4 Cyclo-1,4-Triazol-[-3-Abz-Phe-Pra]-OH (19): 152

1 Abbreviations

°C	degree Celsius
µm	micrometre
µmol	micromole
A	Adenine
Abu	Aminobutyric acid
Abz	Aminobenzoic acid
Ala	Alanine
aq.	aqua
Ava	Aminovaleric acid
BB	broad band decoupling
br	broad
C	Cytosine
calc.	Calculated
d	*Doublet*
dd	Doublet of a Doublet
ddd	Doublet of a Doublet of a Doublet
DEPT	Distortionless Enhancement by Polarisation Transfer
DIC	N,N′-Diisopropylcarbodiimide
DMF	Dimethylformamide
DMT-MM	4-(4,6-dimethoxy-1,3,5-triazin-2-yl)-4-methyl-morpholinium chloride
eq	Equivalents
Fmoc	Fluorenylmethoxycarbonyl
G	Guanine
Gln	Glutamine
Gly	Glycine
HOBt	1-Hydroxybenzotriazole hydrate
Hz	*Hertz*
ISA	Imidazole-1-sulfonyl azide
J	Coupling Constant
l	litre
LC-MS	Liquid chromatography–mass spectrometry
Lys	Lysine
m	Multiplet
m/z	mass-to-charge ratio
mg	milligram
ml	millilitre
mm	millimetre
mM	millimolar
PDA	Photodiodearray-Detetor
Phe	Phenylalanine
ppm	Parts Per Million
RT	Room temperature
s	*Singlet*
Sar	Sarcosine
SQ	Single Quadrupol
t	Triplet
T	Thymine
TBTA	Tris[(1-benzyl-1H-1,2,3-triazol-4-yl)methyl]amine)
td	Triplet of a Doublet
Temp.	temperature
t_R	Retention time
Trp	Tryptophan

Tyr	Tyrosine
α	Alpha
β	Beta
γ	Gamma
δ	Chemical shift

2 Tables

*Table 2.1: Optimizing of the DNA-compatible Diazo-Transfer Reaction with aliphatic **(1)** and aromatic **(2)** Amines*

Entry[a]	ISA (Eq)	CuSO4 (Eq)	Buffer (Mol)	Temp. . (°C)	Reaction Time (h)	Conversion Amine **1** to Azide **3** (%)[b]	Conversion Aniline **2** to Azide **4**(%)[b]
1	50	10	Borate (0.5) pH9.4	RT	1	81	2
2	50	10	Borate (0.5) pH9.4	RT	16	100	39
3	50	1	Borate (0.5) pH9.4	RT	16	61	49
4	50	10	$NaHCO_3$ (0.2)	RT	1	49	9
5	50	10	$NaHCO_3$ (0.2)	RT	16	98	55
6	50	1	$NaHCO_3$ (0.2)	RT	16	100	48
7	50	1	$NaHCO_3$ (0.2)	60°C	16	93[c]	50[c]
8	50	10	K_2CO_3 (0.05)	RT	16	98	96
9	50	1	K_2CO_3 (0.05)	RT	16	7	53

[a]All the reactions were conducted at 0.7 mM in 10 nmol scale; [b]Conversion observed by UPLC–MS; [c]DNA degradation observed by mass spectrometry

*Table 2.2: Optimizing of the DNA-compatible Diazo-Transfer Reaction with aromatic Amine **(2)***

eq. Azide	eq. $CuSO_4$	Buffer	Temp.	Timepoint for aromatic amine 2[a]			
				1 day	2 days	3 days	4 days
50	1	$NaHCO_3$ 200mM	RT	37	63	79	89
50	0	$NaHCO_3$ 200mM	RT	37	62	80	88
50	1	K_2CO_3 50mM	RT	40	67	84	91
50	0	K_2CO_3 50mM	RT	24	59	76	87
50	1	Borate 500mM	RT	37	66	80	90
50	0	Borate 500mM	RT	36	63	79	88

Number of experiments n=1; [a] Conversion observed by UPLC-MS

3 Materials and Instrumentation

HP-280: Biosearch Technologies PN: 185392 Ref.: SS453295-01

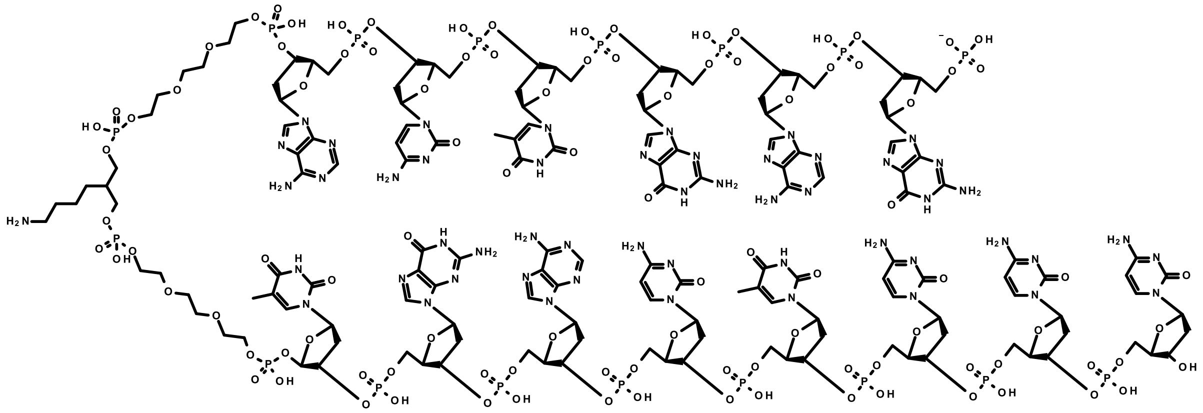

Figure 3.1: Structure of HP-280

ISA.H_2SO_4: 1H-Imidazole -1-sulfonylazide sulfate ABCR PN: AB457437 CAS: 1357503-23-1

Chemicals: The commercial compound and solvent were bought from the following Vendors: Sigma-Aldrich, Bachem, Fluka, Adv. ChemTech, ABCR, Neosystem, VWR and Biotech GmbH.
The Fmoc-Amino acids (**Table 2 Entry 22, 28, 29, 31**) were made from the Amino acids.

NMR: The off-DNA measurements were carried out by the Analytic department. The measurements were done for ^{1}H NMR at 600 MHz or 500 MHz, for ^{13}C NMR-BB and ^{13}C NMR-DEPT at 151 MHz or 126 MHz. DMSO-d_6 was used as solvent. ACD/Spectrus Processor 2018.1.1 from Advanced Chemistry Development, Inc. was used for the evaluation.

LC-MS System: **I**) Waters Acquity UHPLC Plus-System with Xevo Ge-XS TOF MS, PDA, oven (40°C) and automated sample injection ; Mass detection: 300 – 3000 m/z (on-DNA)
II) Waters Acquity UHPLC-MS with PDA and SQ- detector, oven (60°C) and automated sample injection ; Mass detection: 100 – 1000 m/z (off-DNA)
III) Lavomatic HD-3000 preparative HPLC with DAD and fraction collector,

Column: **I**) Kinetex EVO C18 2.6 µm 50 × 2.1 mm PN: 00B-4725-AN from Phenomenex (on-DNA)
II) Acquity UHPLC BEH C18 1.7 µm, 50 ×2.1 mm PN: 186002350 from Waters (off-DNA)

III) Chromatorex C18, 10 µm 125 × 30 mm (preparative HPLC)

Solvent: **I**) A: 2.92 mg EDTA, 10 ml HFIP, 1 ml DIPEA in 1 l Millipore water (on-DNA)

B: 2.92 mg EDTA, 0.75 ml HFIP, 0.375 ml DIPEA in 650 ml Acetonitrile und 350 ml Millipore water (on-DNA)

II+III) A: H_2O + 0.1% (v/v) formic acid (w=99%)

B: Acetonitrile (off-DNA and preparative)

Gradient: **I**) 0.65 ml/min (on-DNA)

II) 0.80 ml/min (off-DNA)

III) 65 ml/min (preparative)

(on-DNA)		(off-DNA)		(preparative)	
min	%B	min	%B	min	%B
0.00	5	0.00	1	0.00	30
0.50	5	1.60	99	2.50	30
3.00	60	2.00	99	8.00	70
3.01	5	2.01	1	8.10	100
3.50	5	2.50	1	10.10	100

3.1 General Procedures

General Procedure 1: Ethanol precipitation (GP1): To the aqueous DNA reaction solutions was added 10% (v/v) of 5 M NaCl, followed by 3 volumes of cold ethanol (-78°C). The suspension was allowed to stand for 30min at -78°C and then centrifuged at 5000 rpm. (>0.15 µmol reaction) or 15000 rpm (≤0.15 µmol reaction) for 15 min at 4 °C. The resulting supernatant was discarded. The pellet was dissolved in water to get same volume as the reaction volume and was freeze-dried or further purified by spin-filtration **(GP2)**. The freeze-dried sample was dissolved in deionized water to get 10 mM solution.

General Procedure 2: Spin-filtration (GP2): The solution from the GP1 was transferred into Amicon Ultra-4 3K Centrifugal-Filter and centrifuged to ¼ of the starting volume. The Centrifugal-Filter was filled twice to the starting volume and centrifuged to ¼ of the starting volume. The solution above the filter was transferred and was freeze-dried. The freeze-dried sample was dissolved in deionized water to get 10 mM solution.

Amide bond formation method with DMT-MM (ABF 1): To a stock solution of **HP-280** (100 µl, 10 mM aq.) was added borate-buffer pH9.5 (900 µl, 500 mM aq.), followed by the addition of a solution of Fmoc-amino acid (100 eq, 250 µl, 400 mM in DMF) the DMT-MM (30 eq, 75 µl, 400 mM aq.). The reaction was allowed to proceed overnight at RT. The DNA was precipitated following the procedure **GP1**. The reaction was analysed by LC-MS.

Amide bond formation method with HOBt/DIC (ABF 2): The **HP-280** (100 µl, 10 mM aq.) was added to borate-buffer pH9.5 (900 µl, 500 mM aq). The HOBt.H2O solution (100 eq, 250 µl, 400 mM in DMF), DIC-solution (100 eq, 250 µl, 400 mM in DMF) and Fmoc-amino acid solution (100 eq, 500 µl, 200 mM in DMF) were combined and allowed to stand for 10 min at room temperature and then was added to the HP-280 solution. The reaction was let to proceed

overnight at RT. The DNA was precipitated following the procedure **GP1**. The reaction was analysed by LC-MS.

Fmoc deprotection: The DNA-conjugated Fmoc-amino acid was diluted in water (1 mM) and 10% v/v Piperidine was added for 2 h at room temperature. The DNA was precipitated following the procedure **GP1** and **GP2**.

Optimized Diazo-Transfer Reaction 1 (DTR 1): 1 µl of DNA-Conjugated amino acid (10mM) was added to 13 µl premixed solution of Copper(II)sulfate pentahydrate (1 eq, 0.5 µl, 20 mM aq.), ISA.H_2SO_4 (50 eq, 2.5 µl, 200 mM aq.) and $NaHCO_3$ (200 eq, 10 µl, 200 mM aq.) and mixed. The reaction was allowed to stand overnight at RT. The reaction was analysed by LC-MS.

Optimized Diazo-Transfer Reaction 2 (DTR 2): 1 µl of DNA-Conjugated amino acid (10 mM) was added to 13 µl premixed solution of Copper(II)sulfate pentahydrate (10 eq, 0.5 µl, 20 mM aq.), ISA.H_2SO_4 (50 eq, 2.5 µl, 200 mM aq.) and K_2CO_3 (50 eq, 10 µl, 50 mM aq.) and mixed. the reaction was allowed to stand overnight at RT. The reaction was analysed by LC-MS. For the LC-MS 1 µl of the reaction solution was added to 50 µl Sodium diethyldithiocarbamate (2 mM aq.) as a copper scavenger. The brown precipitant was centrifuged for 1 min and the supernatant was transferred for LC-MS.

Copper(I)-catalysed intramolecular Alkyne-Azide Cycloaddition "click" reaction method (CuAAC 1): The DNA Conjugated alkyne-azide (1 µL, 10 mM aq.) was diluted in phosphate buffer (9 µL, 500 mM, pH 8). 5µl of Cu(OAc)$_2$.H_2O (2 µl, 2 eq, 10mM DMF), (+)-sodium *L*-ascorbate (2 µl, 4 eq, 20 mM aq.) and TBTA (1µl, 1 eq, 10 mM DMF) mixture was added and the reaction was allowed to proceed for 16 h at RT. The reaction was analysed by LC-MS.

Copper(I)-catalysed intermolecular Alkyne-Azide Cycloaddition "click" reaction method (CuAAC 2): The DNA Conjugated alkyne-azide (1 µL, 10 mM aq.) was diluted in phosphate buffer (9 µL, 500 mM, pH 8). Fmoc-Propargylglycine (1 µl, 10 eq, 100 mM DMF) and 5µl of Cu(OAc)$_2$.H_2O (2 µl, 2 eq, 10mM DMF), (+)-sodium *L*-ascorbate (2 µl, 4 eq, 20 mM aq.) and TBTA (1µl, 1 eq, 10 mM DMF) mixture were subsequently added and the reaction was allowed to proceed for 16 h at RT. The reaction was analysed by LC-MS.

3.2 Example for DNA-Damage during optimisation of Diazo-Transfer Reactions

3.2.1 6-Azidohexanoic acid conjugated with HP280 (3)

Optimization reaction of **6-Azidohexanoic acid (3)** with condition from Table 1 Entry 7 DNA Damage clearly visible in the UV and MS traces with this reaction at 60°C compared to RT

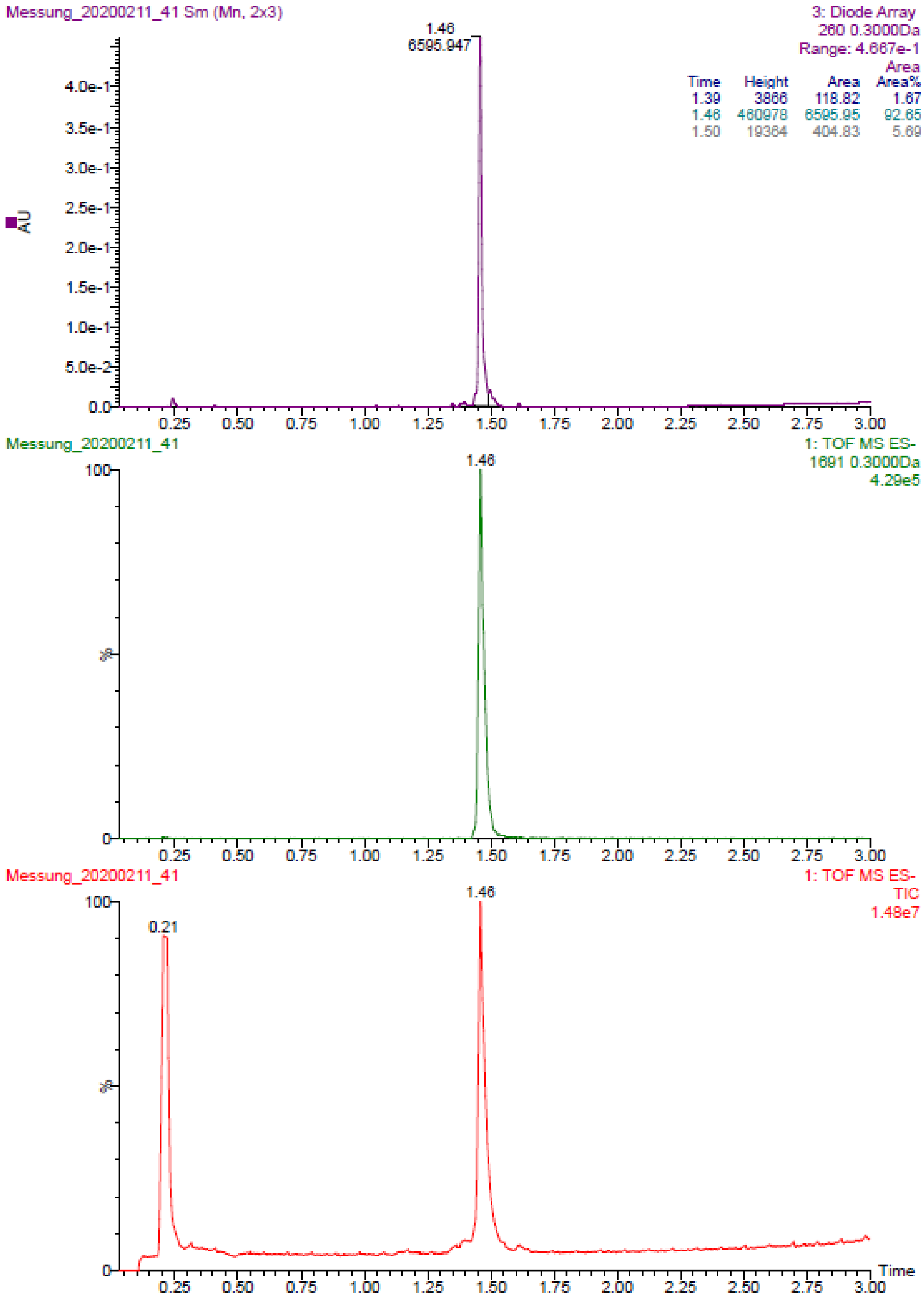

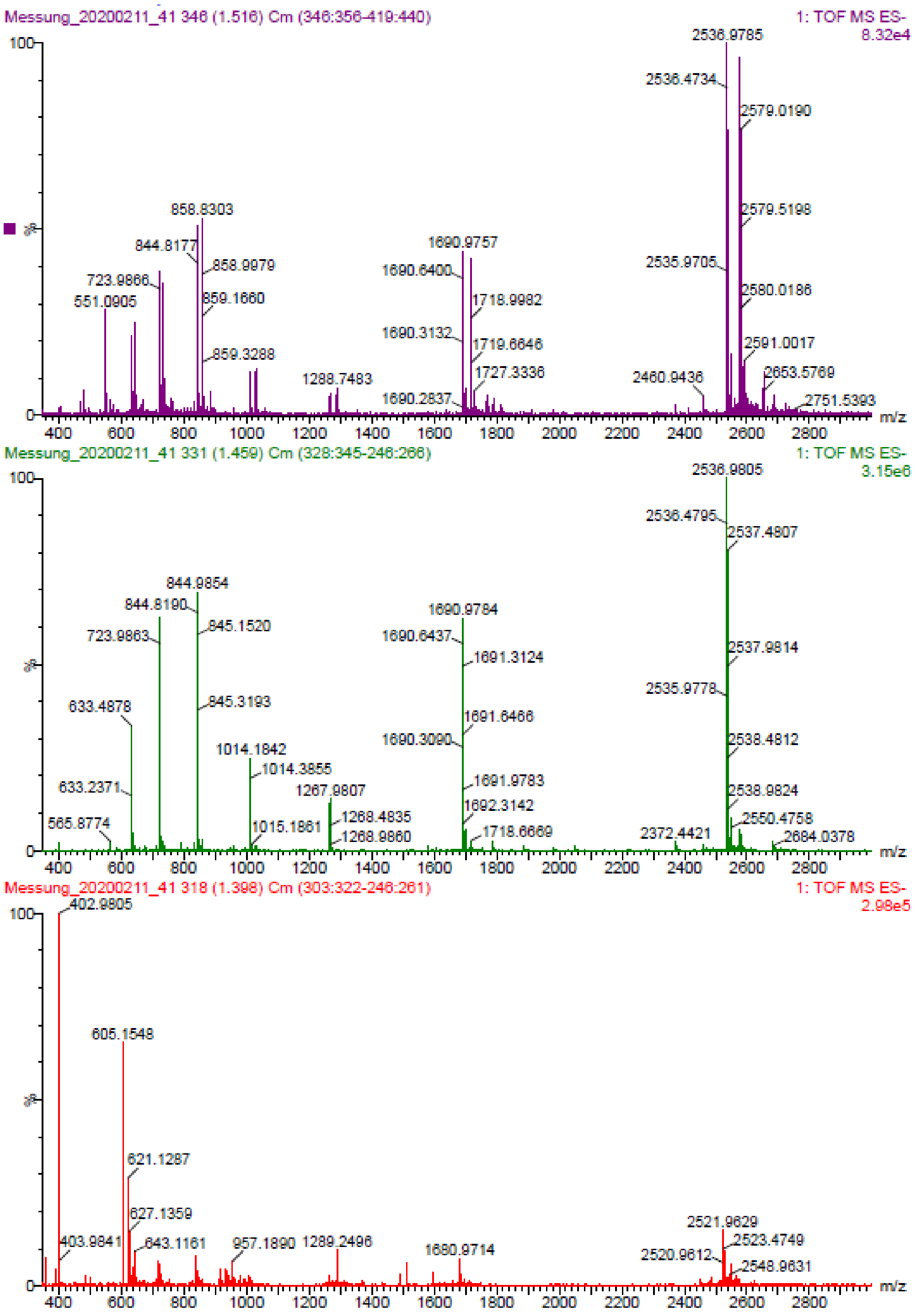

*Figure 3.1: LC-MS chromatogram for compound **3** under condition from **Table 1 Entry 7***

3.2.2 4-Azidobenzoic acid conjugated with HP280 (4, Table 2 Entry 20)

Optimization reaction of **4-Azidobenzoic acid (4)** with condition from Table 1 Entry 20 DNA Damage clearly visible in the UV and MS traces with this reaction at 60°C compared to RT

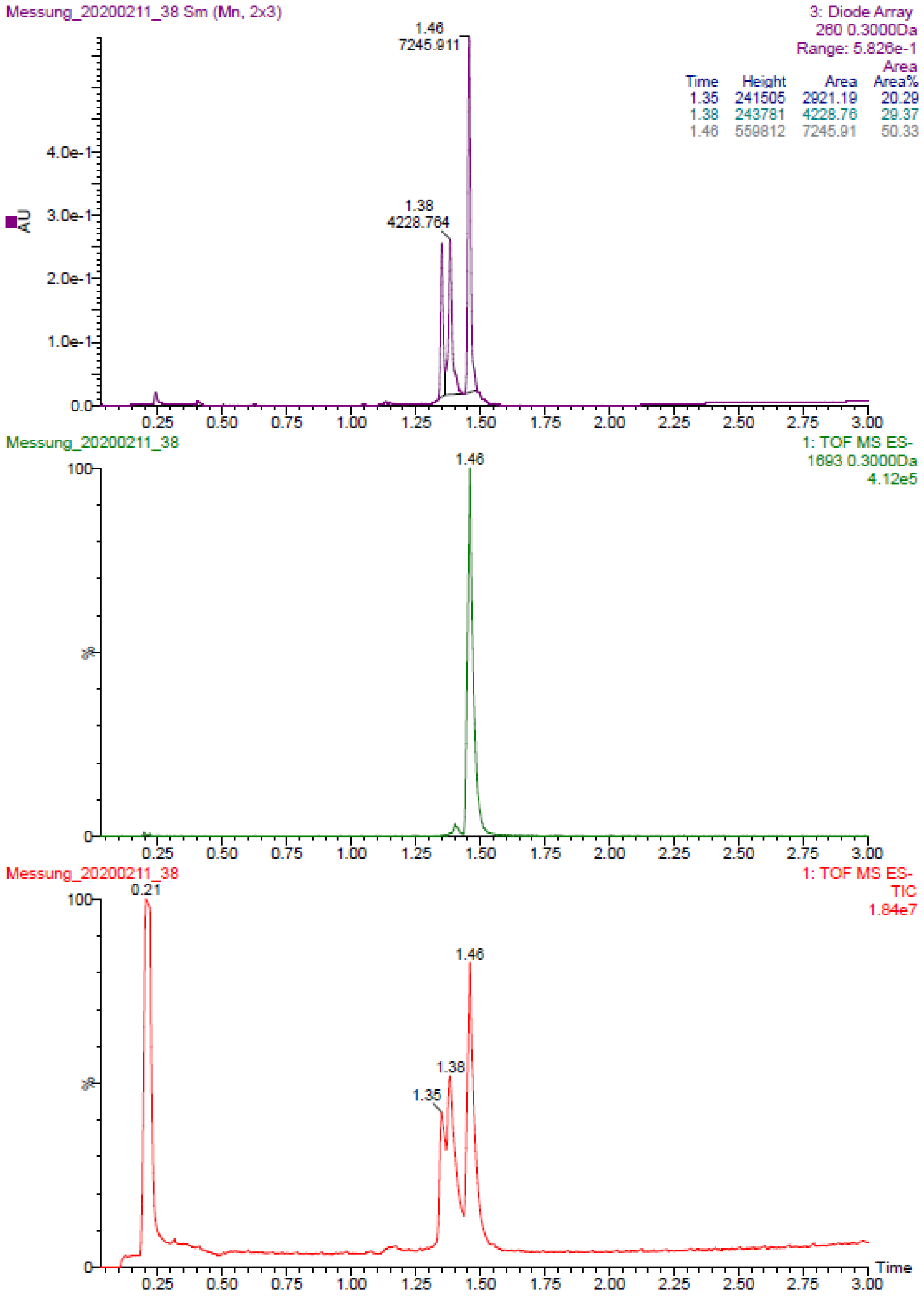

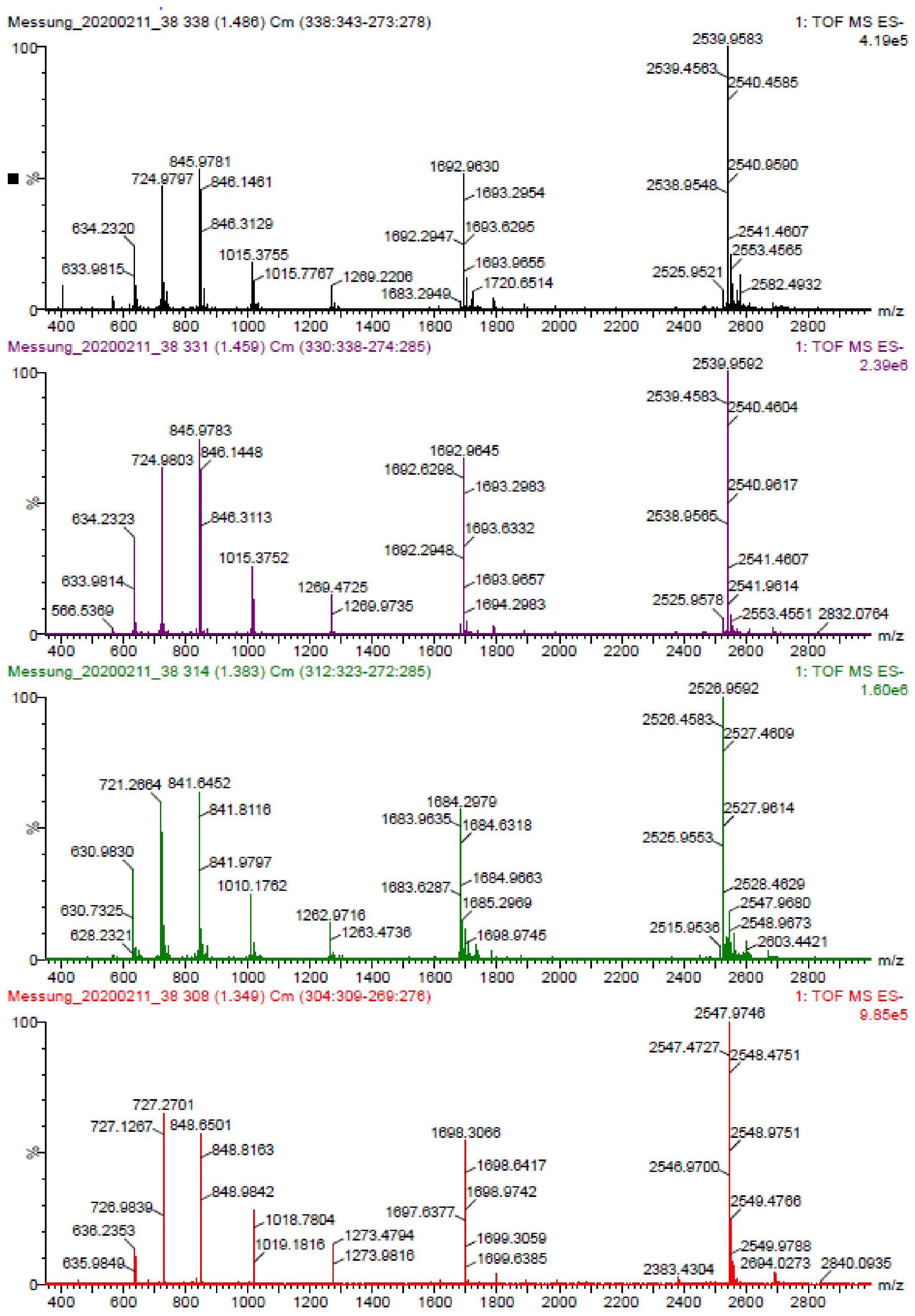

*Figure 3.2: LC-MS chromatogram for compound **4** under condition from **Table 1 Entry 7***

3.3 Chromatograms of DNA-conjugated Amino acids and Azides

3.3.1 6-Azidohexanoic acid conjugated with HP-280 (3, Table 2 Entry 6)

6-Aminohexanoic acid conjugated with HP-280 (2):

Fmoc-6-Aminohexanoic acid (CAS 88574-06-5) was conjugated using general method **ABF 1** (100 µl, 10 mM HP-280, 1µmol scale) and purified with general purification method **GP1**. The Fmoc deprotection was carried out using general method for Fmoc deprotection and was purified using the methods **GP1** and **GP2**:

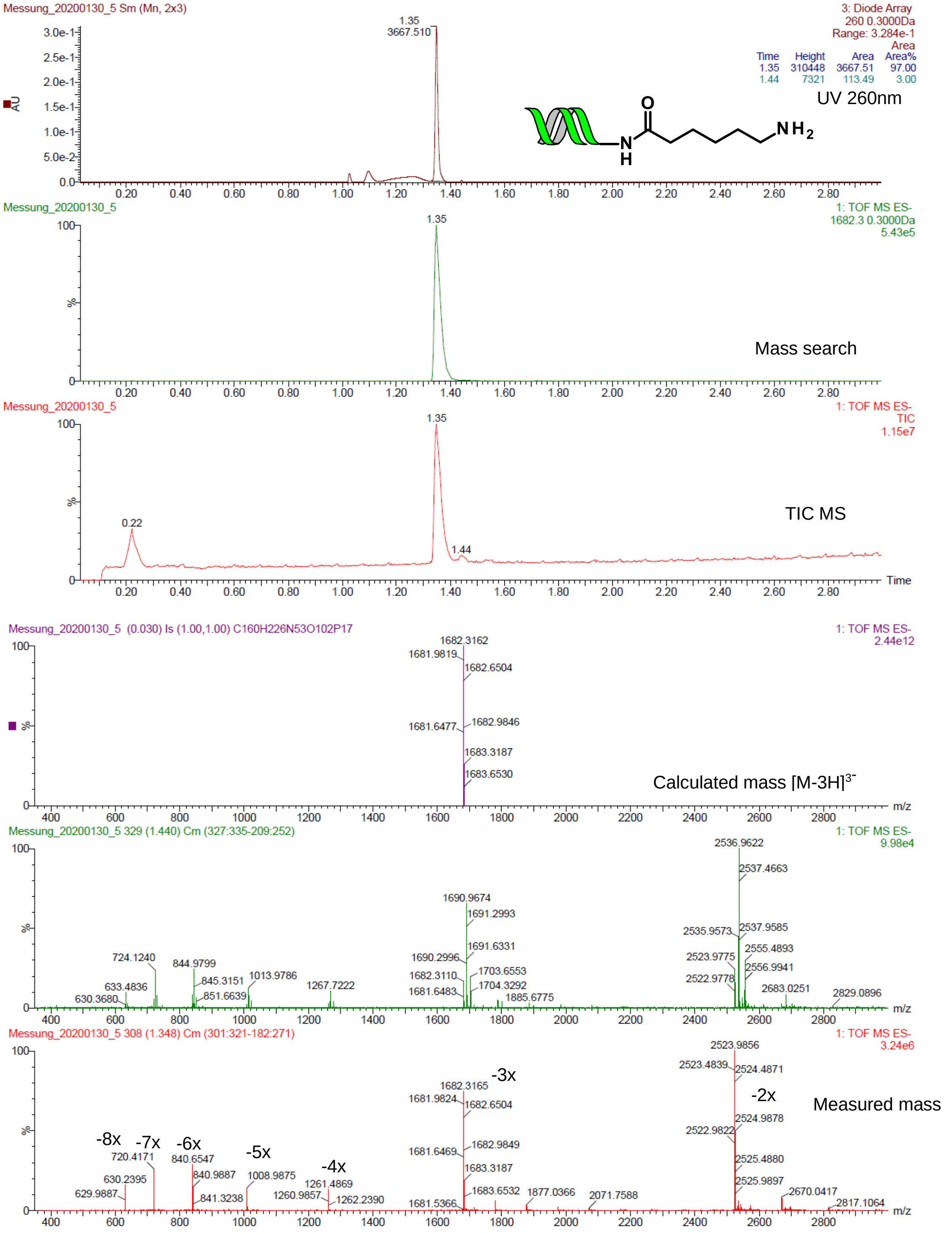

*Figure 3.2: LC-MS chromatogram for compound **1** t_R = 1,35 min TOF-MS-ESI$^-$ m/z =1682,317 (100%) $[M-3H]^{3-}$ (calc. 1682,316 for $C_{160}H_{226}N_{53}O_{102}P_{17}$)*

6-Azidohexanoic acid conjugated with HP-280 (3, Table 2 Entry 6)

The reaction was carried out by using the general method **DTR 1** (1 µl, 10 mM of **(1)**, 10nmol scale).

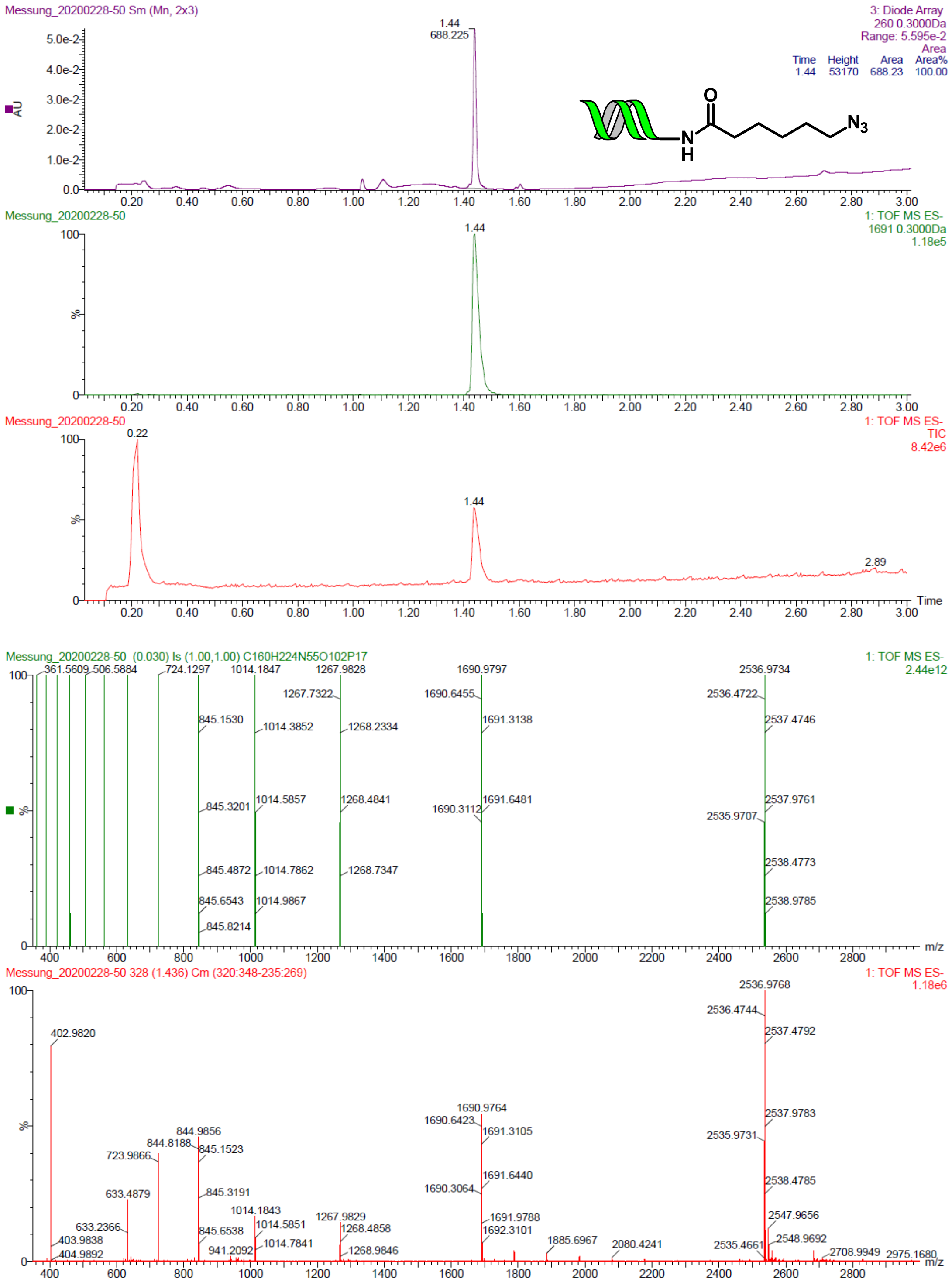

Figure. 3.3: LC-MS chromatogram of compound 2 t_R = 1,44 min TOF-MS-ESI$^-$ m/z = 1690,976 (100%) [M-3H]$^{3-}$ (calc. 1690,980 for $C_{160}H_{224}N_{55}O_{102}P_{17}$)

3.3.2 4-Azidobenzoic acid conjugated with HP-280 (4, Table 2 Entry 20)

4-Aminobenzoic acid conjugated with HP-280 (3):

Fmoc-4-Aminobenzoic acid (CAS 185116-43-2) was conjugated using general method **ABF 2** (100 µl, 10 mM HP-280, 1µmol scale) and purified with general purification method **GP1**. The Fmoc deprotection was carried out using general method for Fmoc deprotection and was purified using the methods **GP1** and **GP2**:

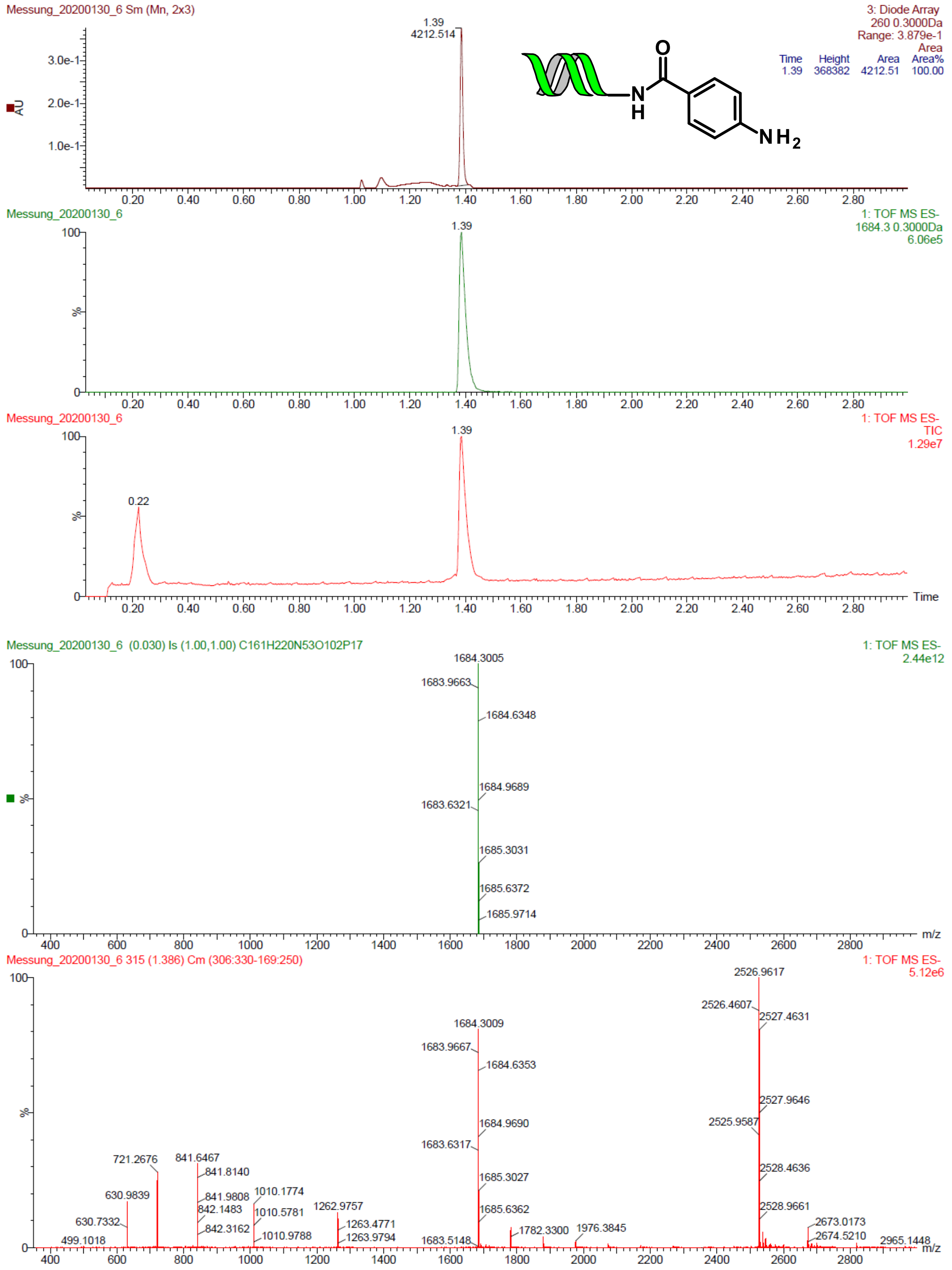

*Figure 3.4: LC-MS chromatogram of compound **3** t_R = 1,39 min TOF-MS-ESI$^-$ m/z = 1684,301(100%) [M-3H]$^{3-}$ (calc. 1684,301 for $C_{161}H_{220}N_{53}O_{102}P_{17}$)*

4-Azidobenzoic acid conjugated with HP-280 (4, Table 2 Entry 20):

The reaction was carried out by using the general method **DTR 2** (1 µl, 10 mM of **(3)**, 10nmol scale).

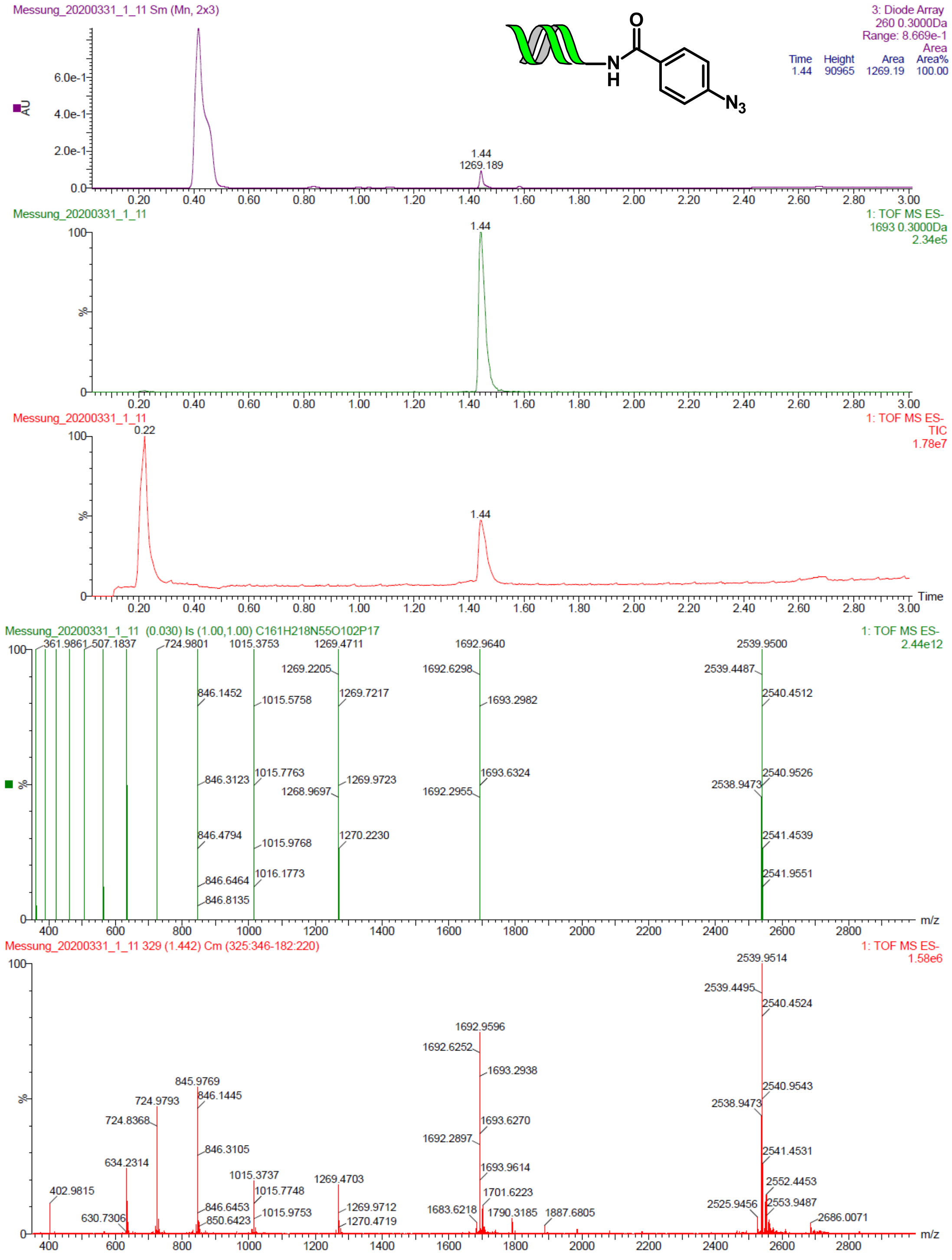

*Figure 3.5: LC-MS chromatogram of compound **4** t_R = 1,44 min TOF-MS-ESI$^-$ m/z = 1692,960(100%) [M-3H]$^{3-}$ (calc. 1692,964 for $C_{161}H_{218}N_{55}O_{102}P_{17}$)*

3.3.3 HP-280 Azide (Table 2 Entry 1)

HP-280 Amine:

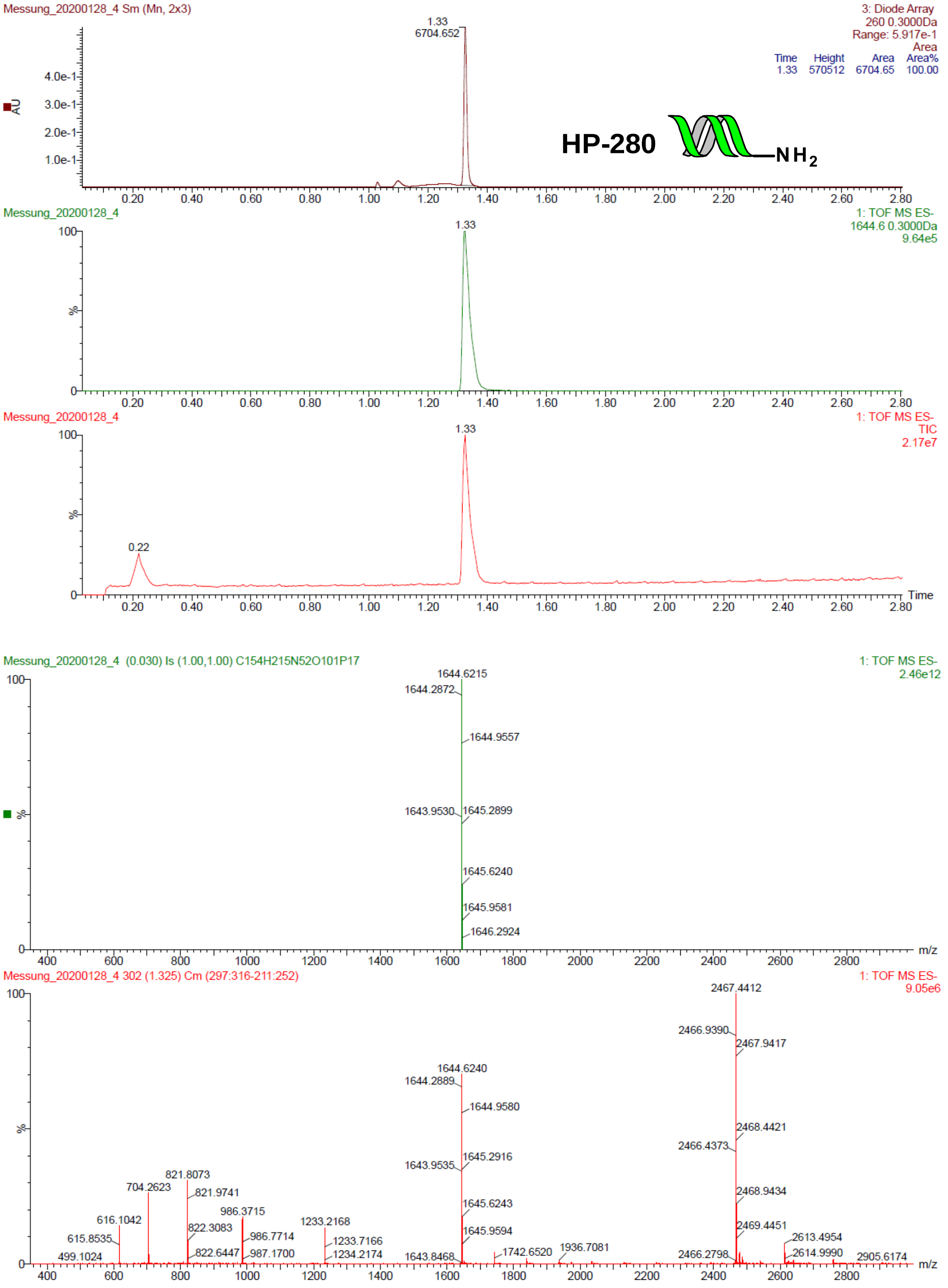

*Figure 3.6 LC-MS chromatogram of compound **Table 2 Entry 1 amine** t_R = 1,31 min TOF-MS-ESI$^-$ m/z = 1644,624 (100%) [M3H]$^{3-}$ (calc. 1644,622 for $C_{154}H_{215}N_{52}O_{101}P_{17}$)*

HP-280 Azide (Table 2 Entry 1):

The reaction was carried out by using the general method **DTR 1** (1 µl, 10 mM of **(HP-280)**, 10nmol scale).

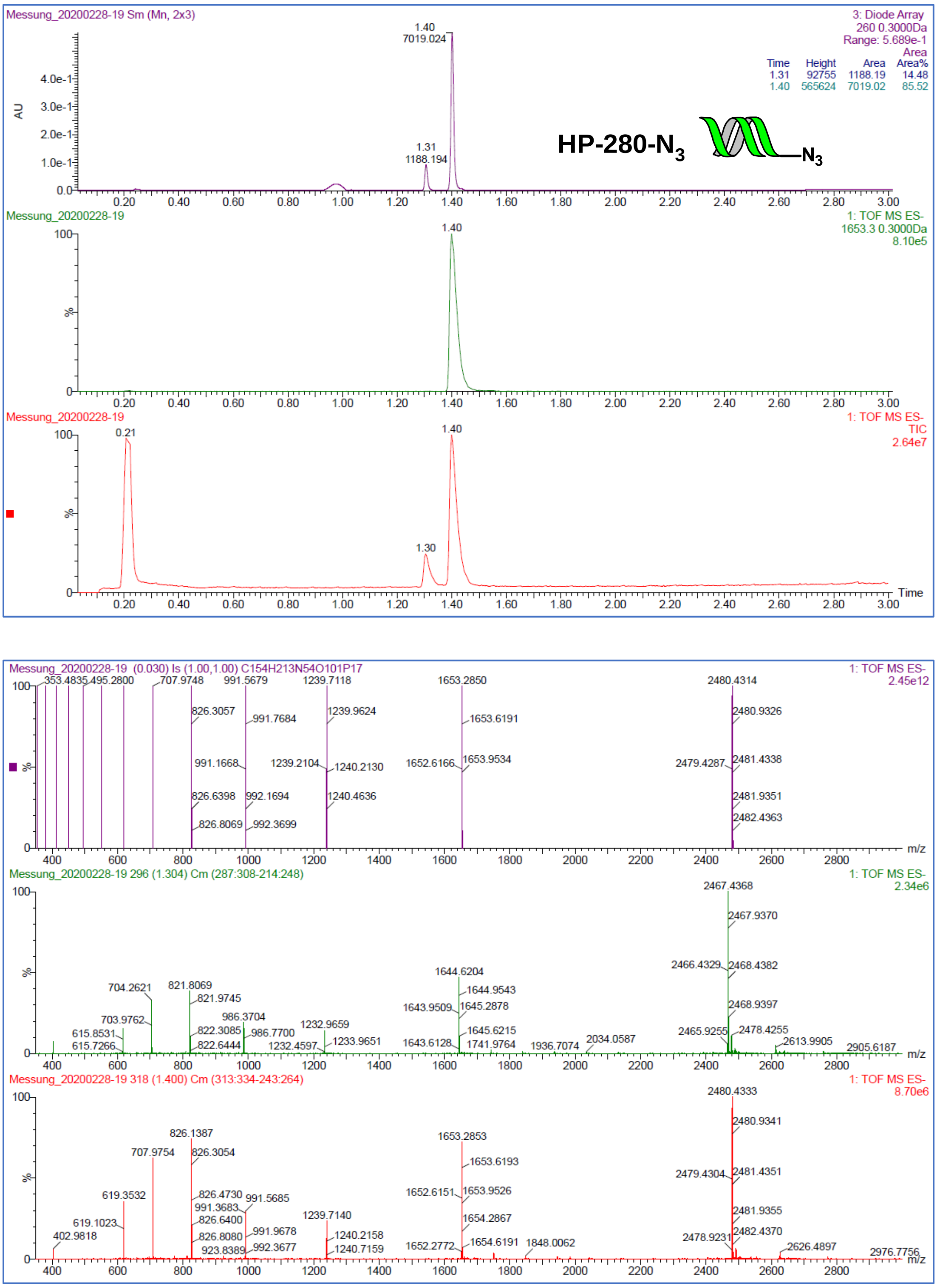

*Figure 3.7: LC-MS chromatogram of compound **Table 2 Entry 1** t_R = 1,40 min TOF-MS-ESI$^-$ m/z = 1653,285 (100%) $[M-3H]^{3-}$ (calc. 1653,285 for $C_{154}H_{213}N_{54}O_{101}P_{17}$)*

3.3.4 Azido-Gly-OH conjugated with HP-280 (Table 2 Entry 2)

H-Gly-OH conjugated with HP-280:

Fmoc-Gly-OH (CAS 29022-11-5) was conjugated using general method **ABF 1** (15 µl, 10 mM HP-280, 150mmol scale) and purified with general purification method **GP1**. The Fmoc deprotection was carried out using general method for Fmoc deprotection and was purified using the methods **GP1** and **GP2**:

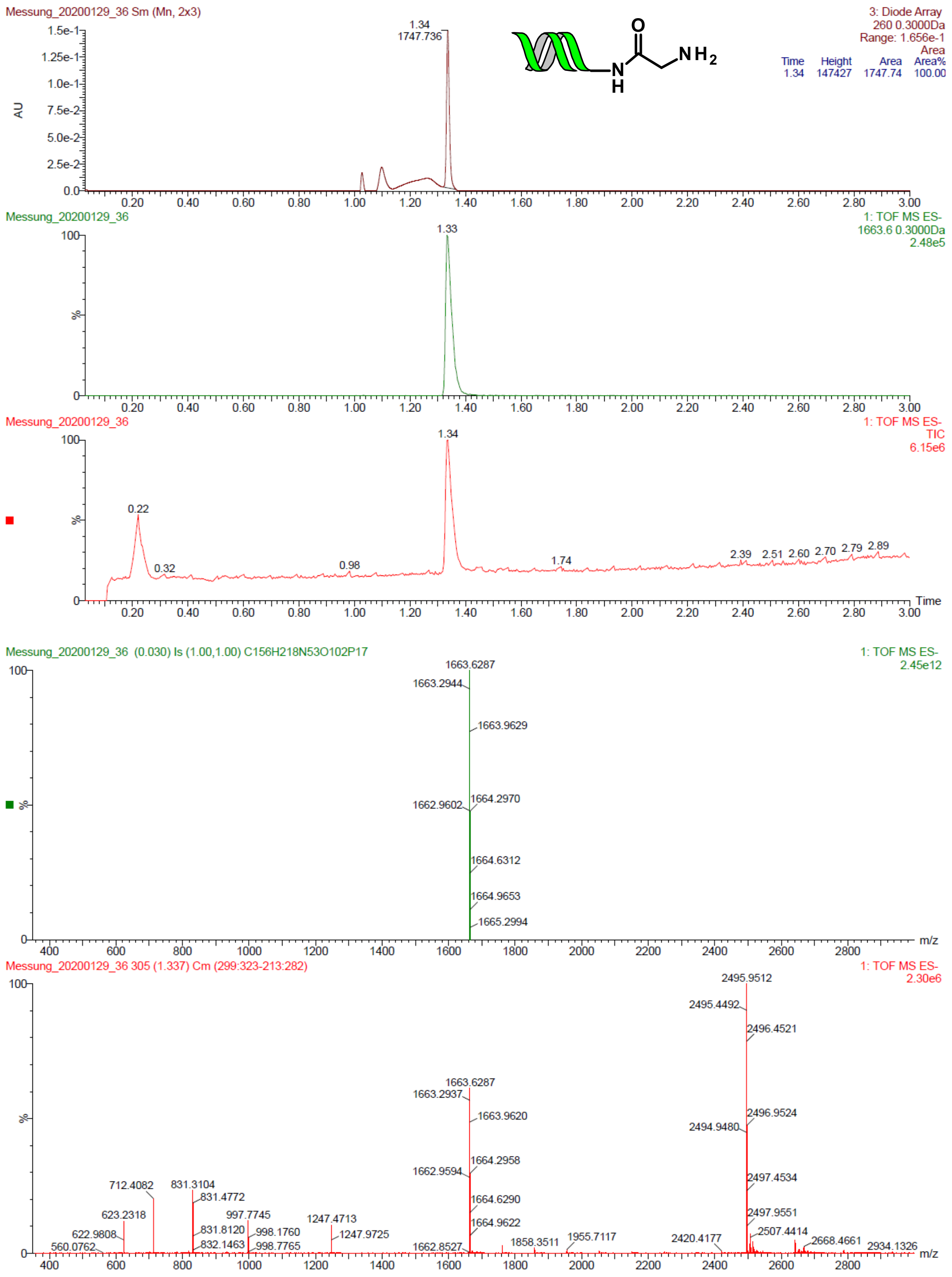

*Figure 3.8: LC-MS chromatogram of compound **Table 2 Entry 2 amine** t_R = 1,34 min TOF-MS-ESI$^-$ m/z = 1663,629(100%) [M-3H]$^{3-}$ (calc. 1663,629 for $C_{156}H_{218}N_{53}O_{102}P_{17}$)*

Azido-Gly-OH conjugated with HP-280 (Table 2 Entry 2):

The reaction was carried out by using the general method **DTR 1** (1 µl, 10 mM of **(Table 2 Entry 2 amine)**, 10nmol scale).

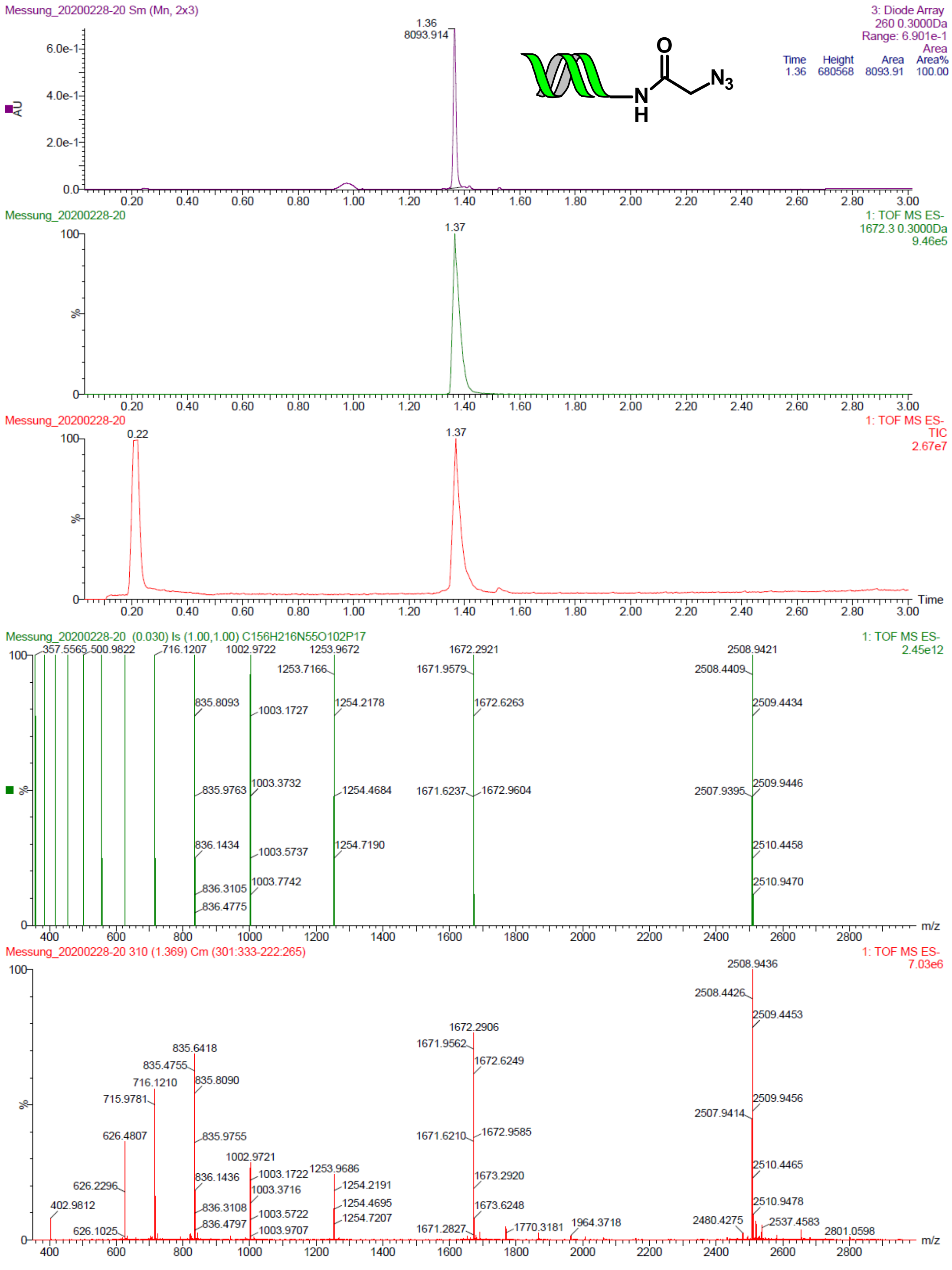

*Figure 3.9: LC-MS chromatogram of compound **Table 2 Entry 3 amine** t_R = 1,36 min TOF-MS-ESI$^-$ m/z = 1672,291100%) [M-3H]$^{3-}$ (calc. 1672,292 for $C_{156}H_{216}N_{55}O_{102}P_{17}$)*

3.3.5 Azido-β-Ala-OH conjugated with HP-280 (Table 2 Entry 3)

H-β-Ala-OH conjugated with HP-280:

Fmoc-β-Ala-OH (CAS 35737-10-1) was conjugated using general method **ABF 1** (15 µl, 10 mM HP-280, 150 nmol scale) and purified with general purification method **GP1**. The Fmoc deprotection was carried out using general method for Fmoc deprotection and was purified using the methods **GP1** and **GP2**:

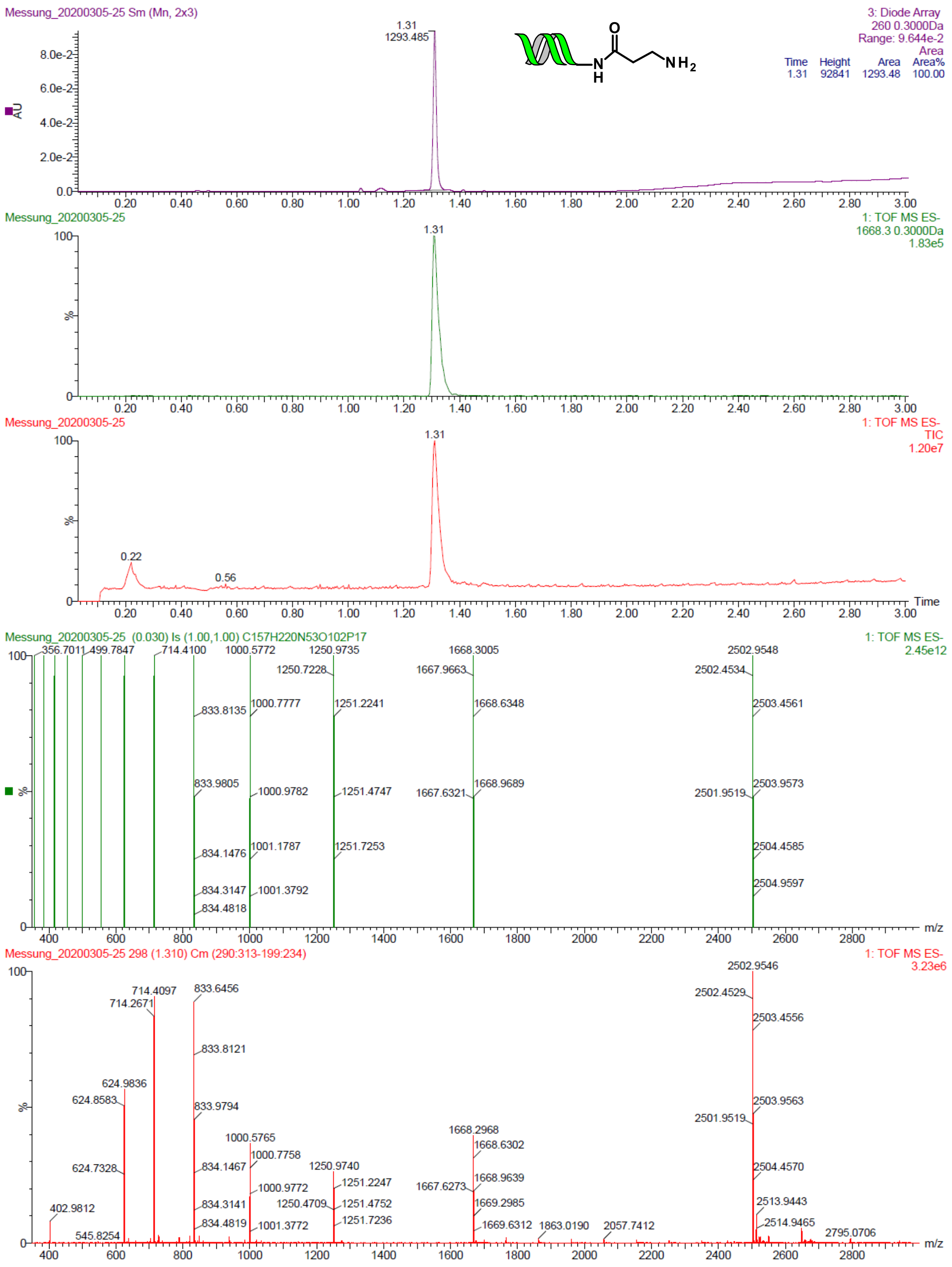

*Figure 3.10: LC-MS chromatogram of compound **Table 2 Entry 3 amine** t_R=1,31min TOF-MS-ESI$^-$ m/z= 1668,297(100%) [M-3H]$^{3-}$ (calc. 1668,301 for $C_{157}H_{220}N_{53}O_{102}P_{17}$)*

Azido-β-Ala-OH conjugated with HP-280 (Table 2 Entry 3):

The reaction was carried out by using the general method **DTR 1** (1 µl, 10 mM of **(Table 2 Entry 3 amine)**, 10nmol scale)-

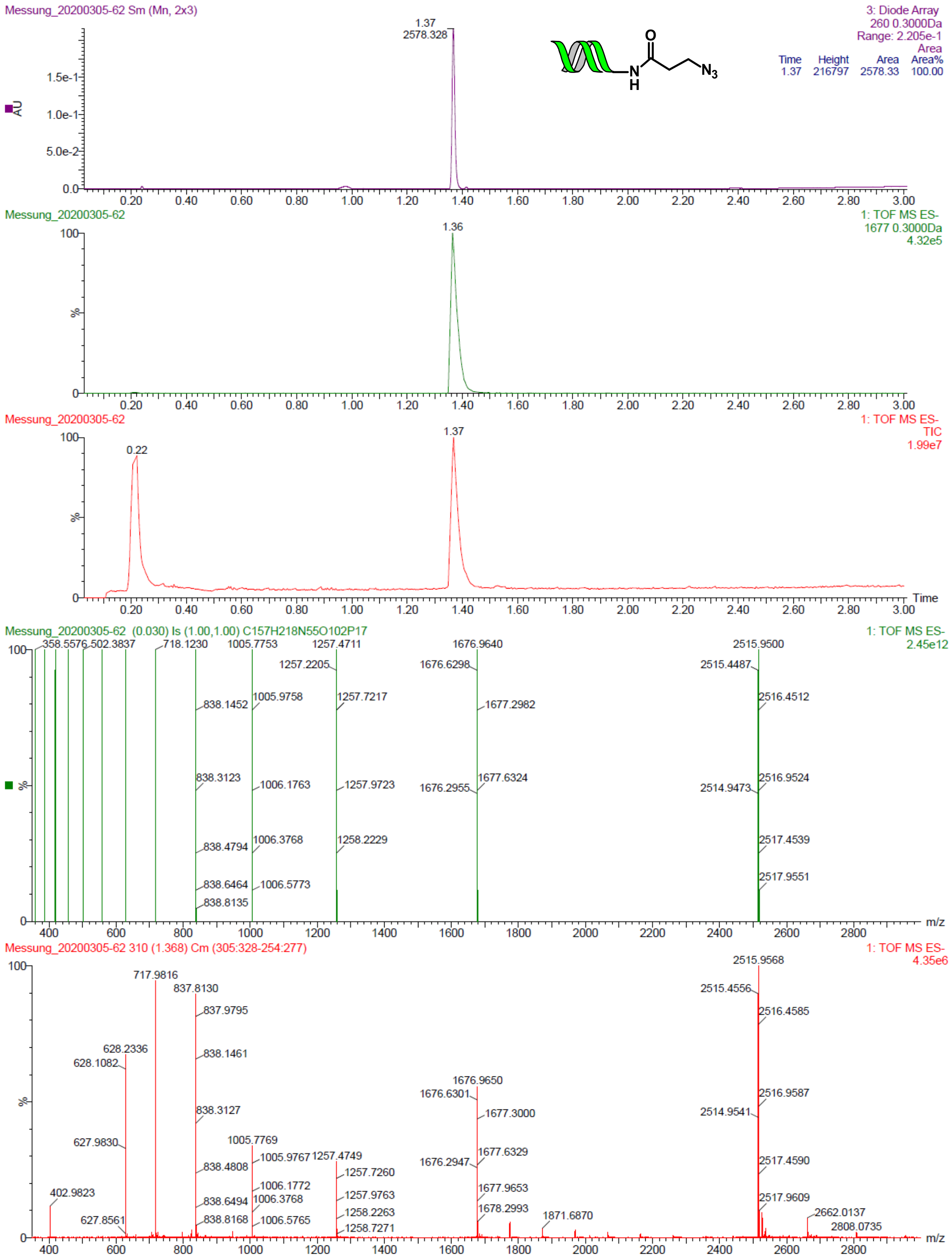

Figure 3.11: LC-MS chromatogram of compound ***Table 2 Entry 1*** *t_R = 1,37 min TOF-MS-ESI$^-$ m/z = 1676,965(100%) [M-3H]$^{3-}$ (calc. 1676,964 for $C_{157}H_{218}N_{55}O_{102}P_{17}$)*

3.3.6 Azido-γ-Abu-OH conjugated with HP-280 (Table 2 Entry 4)

H-γ-Abu-OH conjugated with HP-280:

Fmoc-γ-Abu-OH (CAS 135112-7-5) was conjugated using general method **ABF 1** (15 µl, 10 mM HP-280, 150 nmol scale) and purified with general purification method **GP1**. The Fmoc deprotection was carried out using general method for Fmoc deprotection and was purified using the methods **GP1** and **GP2.**

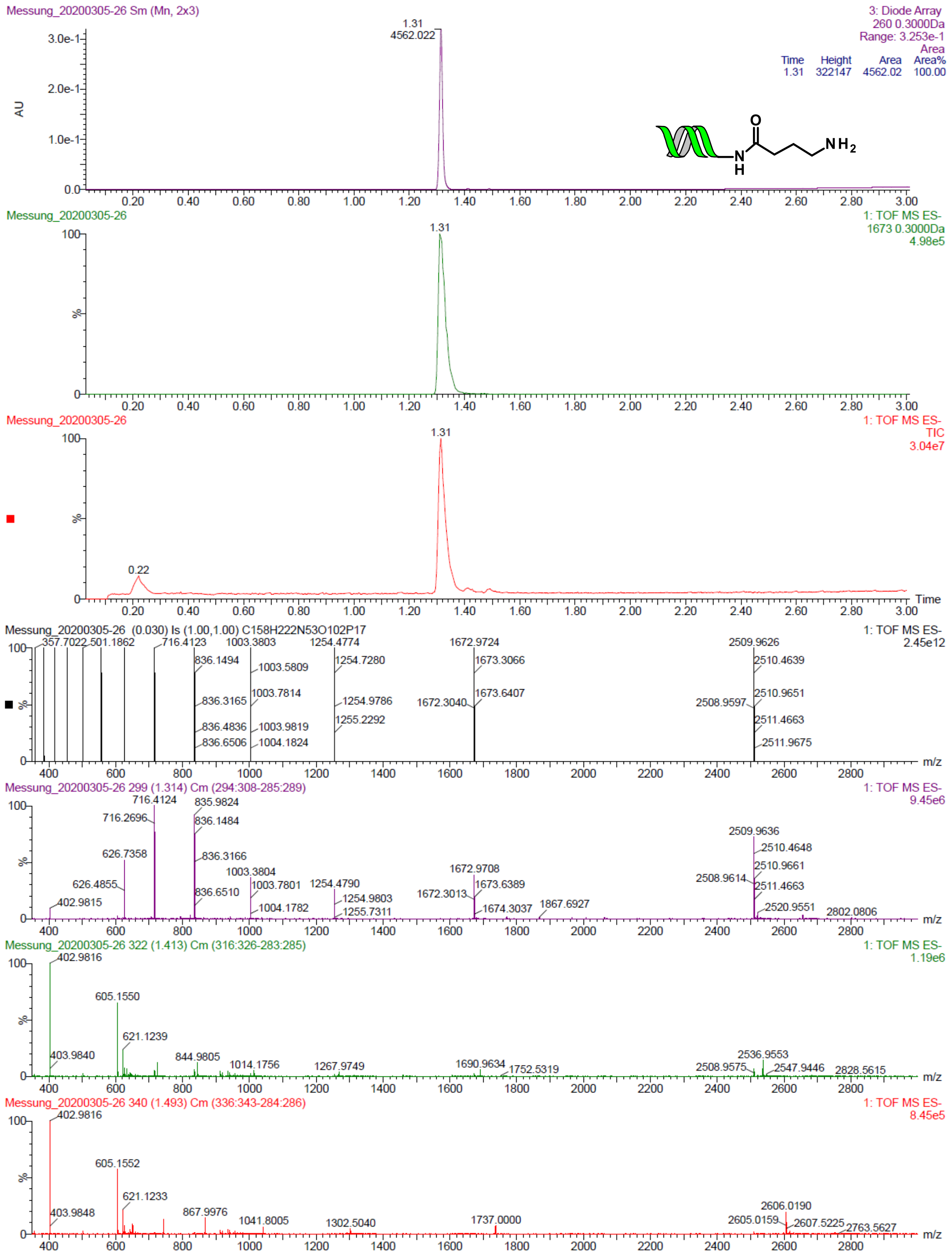

*Figure 3.12: LC-MS chromatogram of compound **Table 2 Entry 4 amine** t_R = 1,31min TOF-MS-ESI$^-$ m/z = 1672,971(100%) [M-3H]$^{3-}$ (calc. 1672,972 for $C_{159}H_{224}N_{53}O_{102}P_{17}$)*

Azido-γ-Abu-OH conjugated with HP-280 (Table 2 Entry 4):

The reaction was carried out by using the general method **DTR 1** (1 µl, 10 mM of **(Table 2 Entry 4 amine)**, 10nmol scale)

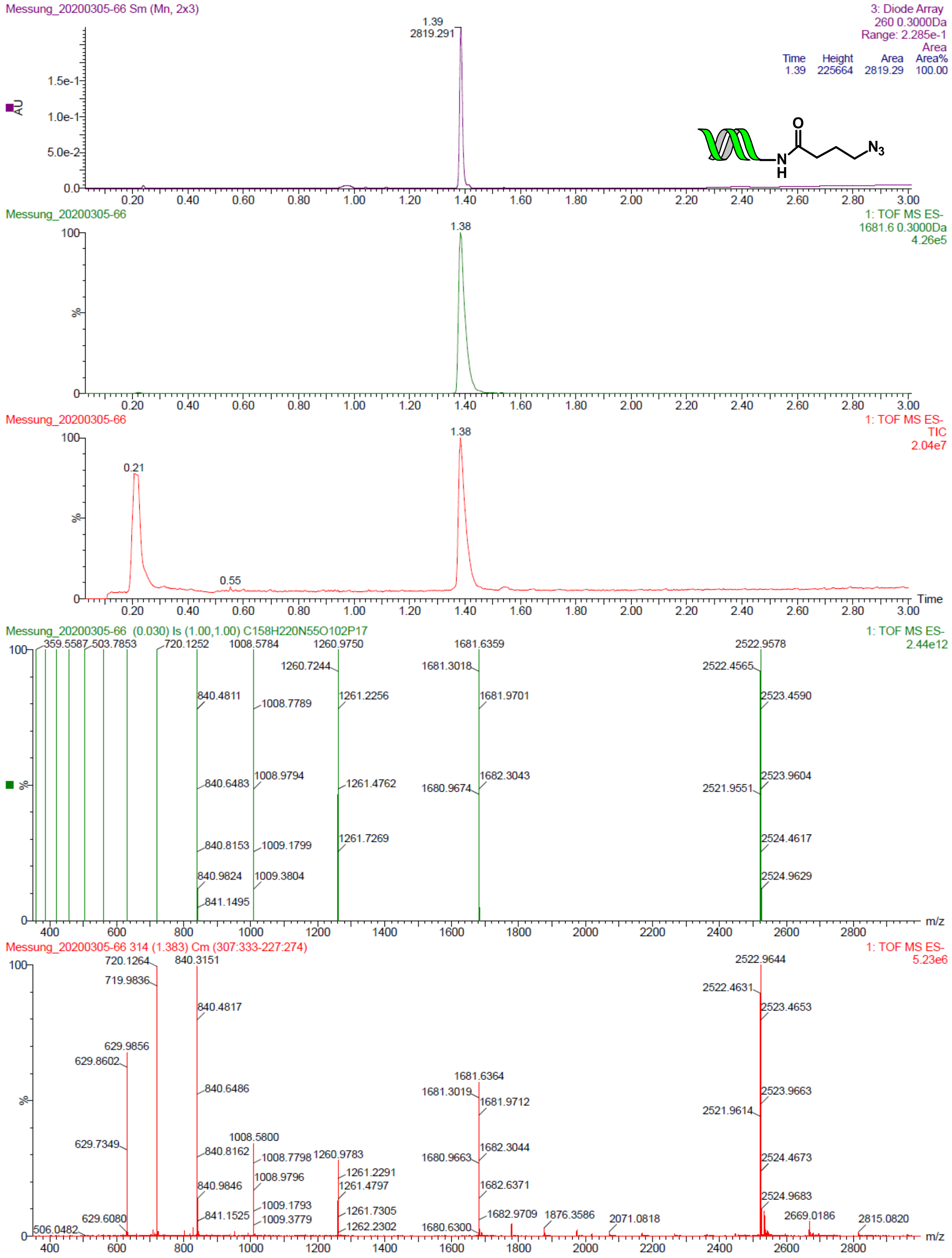

*Figure 3.13: LC-MS chromatogram of compound **Table 2 Entry 4** t_R = 1,39 min TOF-MS-ESI$^-$ m/z = 1681,636(100%) [M-3H]$^{3-}$ (calc. 1681,636 for $C_{158}H_{220}N_{55}O_{102}P_{17}$)*

3.3.7 Azido-5-Ava-OH conjugated with HP-280 (Table 2 Entry 5)

H-5-Ava-OH conjugated with HP-280:

Fmoc-5-Ava-OH (CAS 123622-48-0) was conjugated using general method **ABF 1** (15 µl, 10 mM HP-280, 150 nmol scale) and purified with general purification method **GP1**. The Fmoc deprotection was carried out using general method for Fmoc deprotection and was purified using the methods **GP1** and **GP2**.

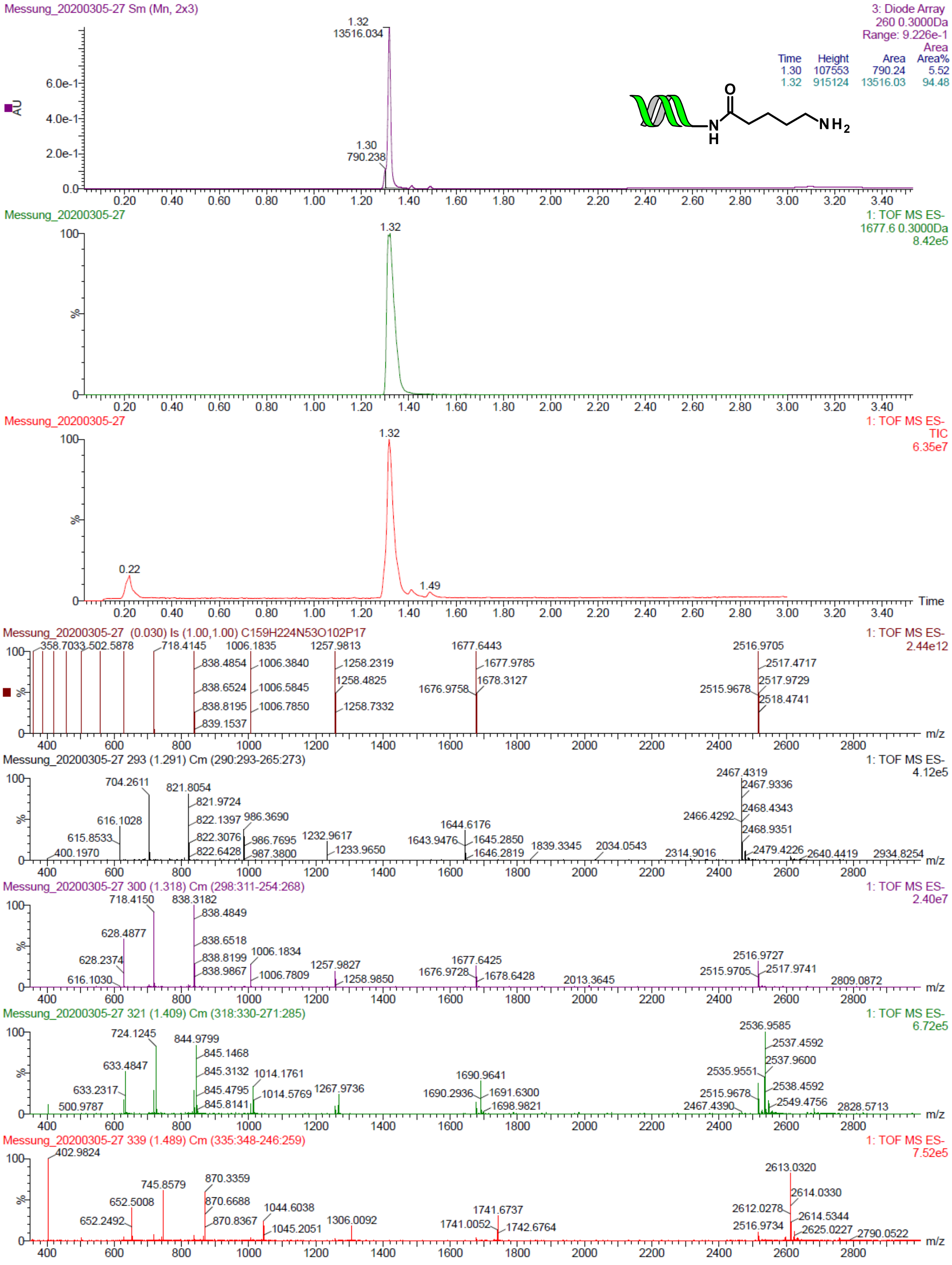

Time	Height	Area	Area%
1.30	107553	790.24	5.52
1.32	915124	13516.03	94.48

Figure 3.14: LC-MS chromatogram of compound ***Table 2 Entry 5 amine*** *t_R = 1,43 min TOF-MS-ESI⁻ m/z = 1677,643(100%) $[M-3H]^{3-}$ (calc. 1677,644 for $C_{159}H_{224}N_{53}O_{102}P_{17}$)*

Azido-5-Ava-OH conjugated with HP-280 (Table 2 Entry 5):

The reaction was carried out by using the general method **DTR 1** (1 µl, 10 mM of **(Table 2 Entry 5 amine)**, 10nmol scale)

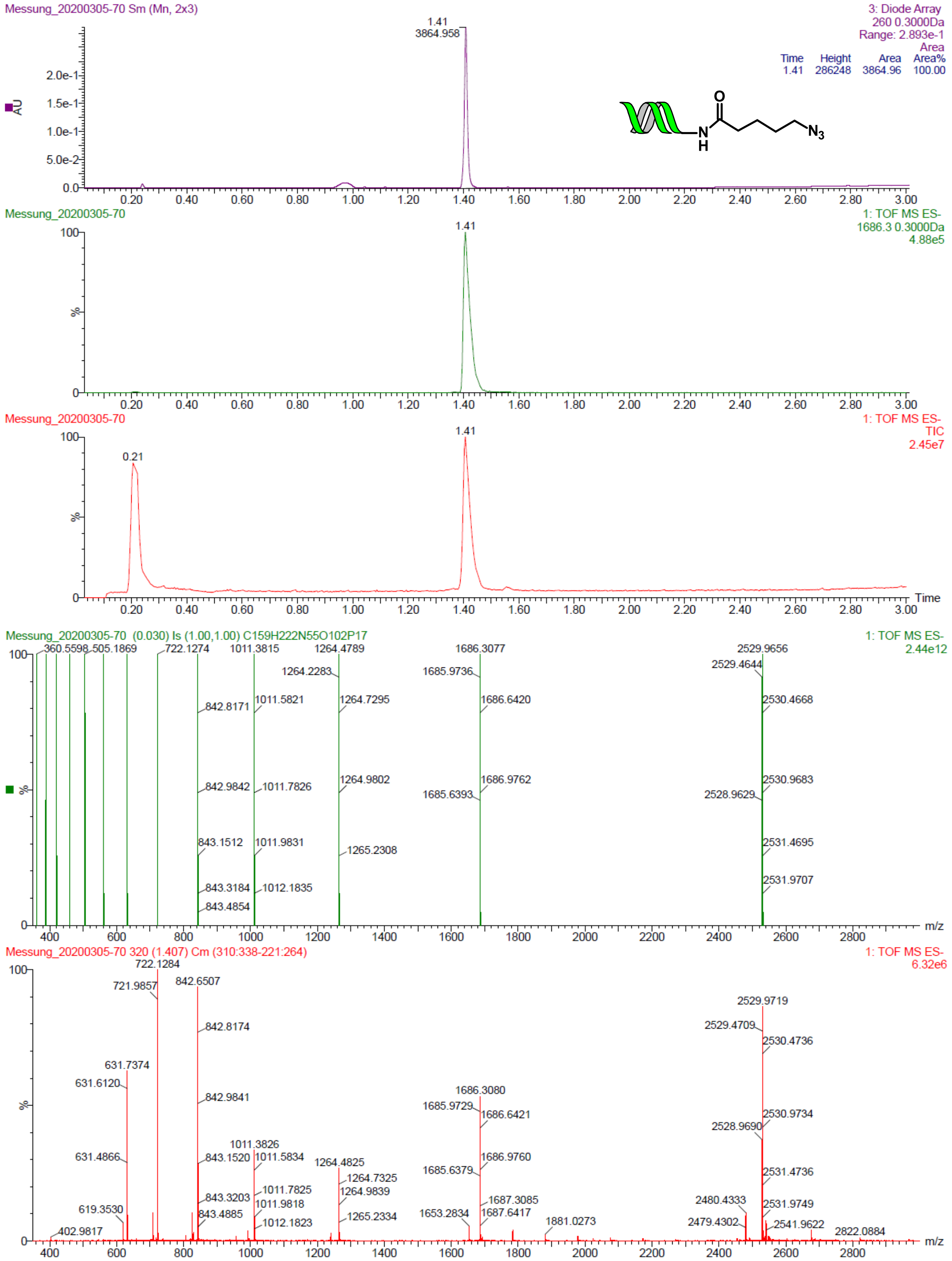

Figure 3.15: LC-MS Chromatogram of compound ***Table 2 Entry 5*** *t_R = 1,41 min TOF-MS-ESI⁻ m/z = 1686,308(100%) $[M-3H]^{3-}$ (calc. 1686,308 for $C_{159}H_{222}N_{55}O_{102}P_{17}$)*

3.3.8 *Azido-Sar*-OH conjugated with HP-280 (Table 2 Entry 7)

H-Sar-OH conjugated with HP-280:

Fmoc-Sar-OH (CAS 77128-70-2) was conjugated using general method **ABF 1** (15 µl, 10 mM HP-280, 150 nmol scale) and purified with general purification method **GP1**. The Fmoc deprotection was carried out using general method for Fmoc deprotection and was purified using the methods **GP1** and **GP2**.

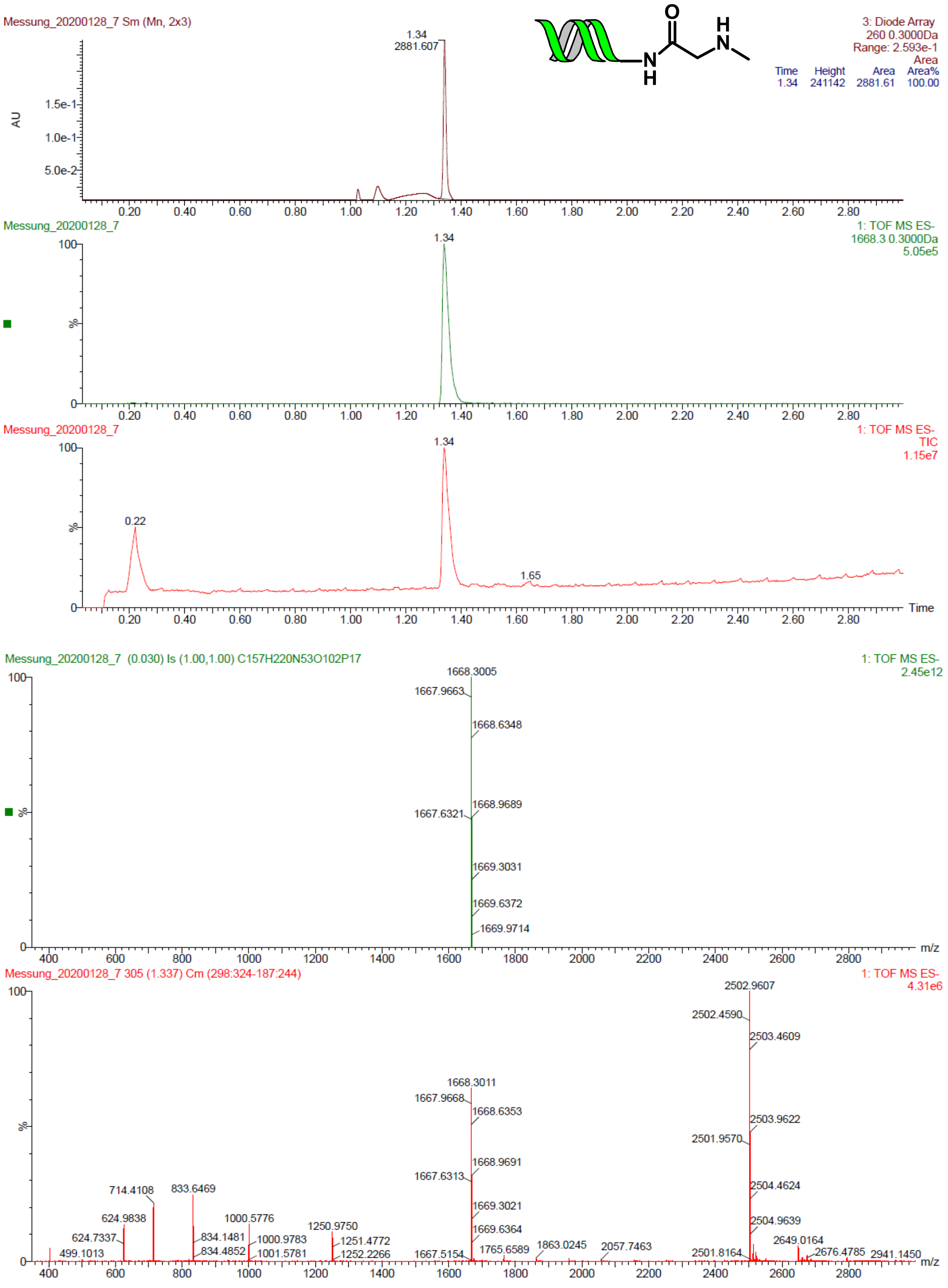

*Figure 3.16: LC-MS chromatogram of compound **Table 2 Entry 7 amine** t_R = 1,34 min TOF-MS-ESI$^-$ m/z = 1668,301(100%) [M-3H]$^{3-}$ (calc. 1668,301 for $C_{161}H_{218}N_{55}O_{102}P_{17}$)*

H-Sar-OH conjugated with HP-280 after Diazo-Transfer Reaction:

The reaction was carried out by using the general method DTR 1 (1 µl, 10 mM of **(Table 2 Entry 7 amine)**, 10nmol scale)

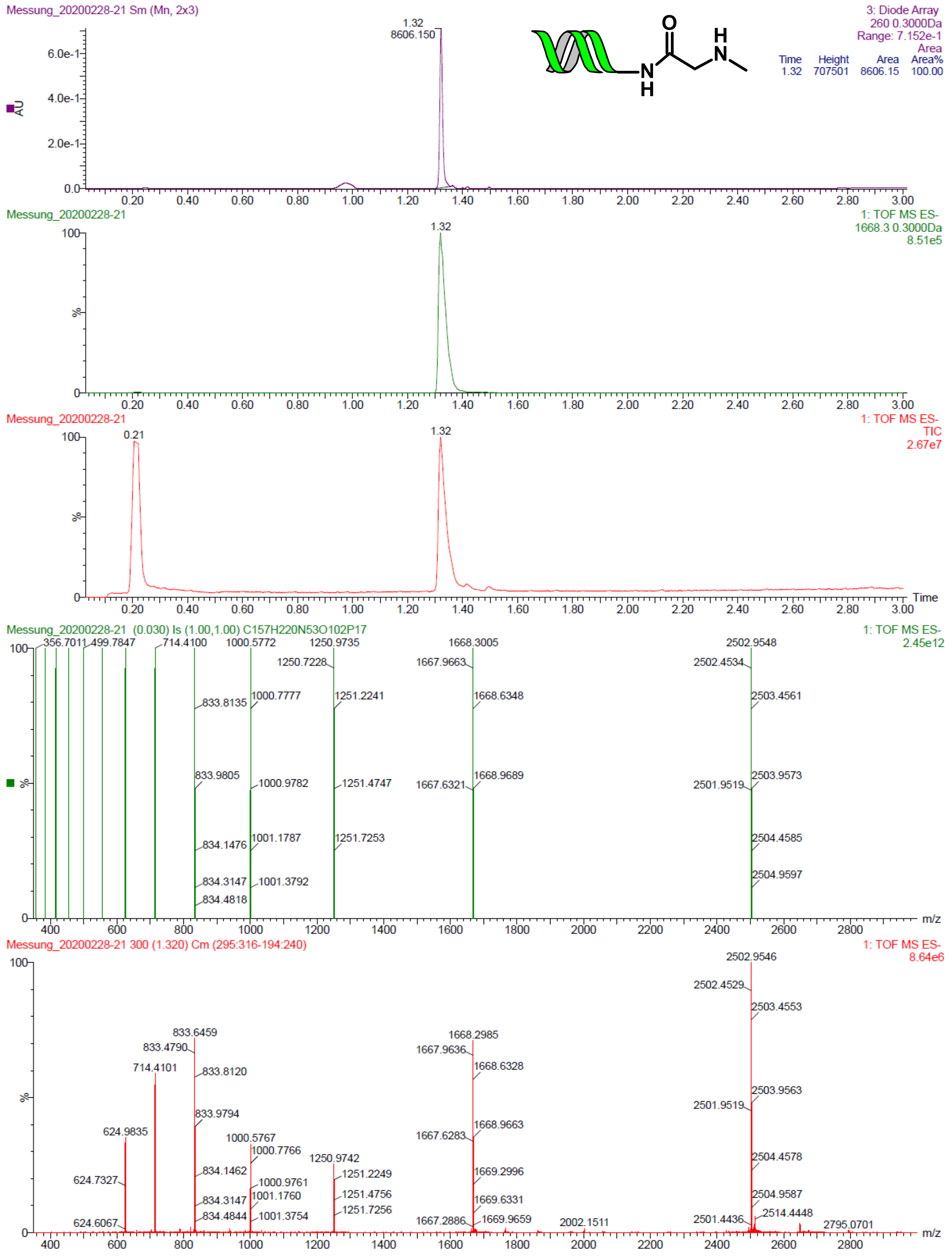

*Figure 3.17: LC-MS Chromatogram of compound **Table 2 Entry 7 amine** after Diazo-Transfer Reaktion*
t_R = 1,34 min TOF-MS-ESI$^-$ m/z = 1668,299 (100%) $[M-3H]^{3-}$ (calc. 1668,301 for $C_{161}H_{218}N_{55}O_{102}P_{17}$)

3.3.9 Azido-Ala-OH conjugated with HP-280 (Table 2 Entry 8)

H-Ala-OH conjugated with HP-280:

Fmoc-Ala-OH (CAS 35661-39-3) was conjugated using general method **ABF 1** (15 µl, 10 mM HP-280, 150 nmol scale) and purified with general purification method **GP1**. The Fmoc deprotection was carried out using general method for Fmoc deprotection and was purified using the methods **GP1** and **GP2**.

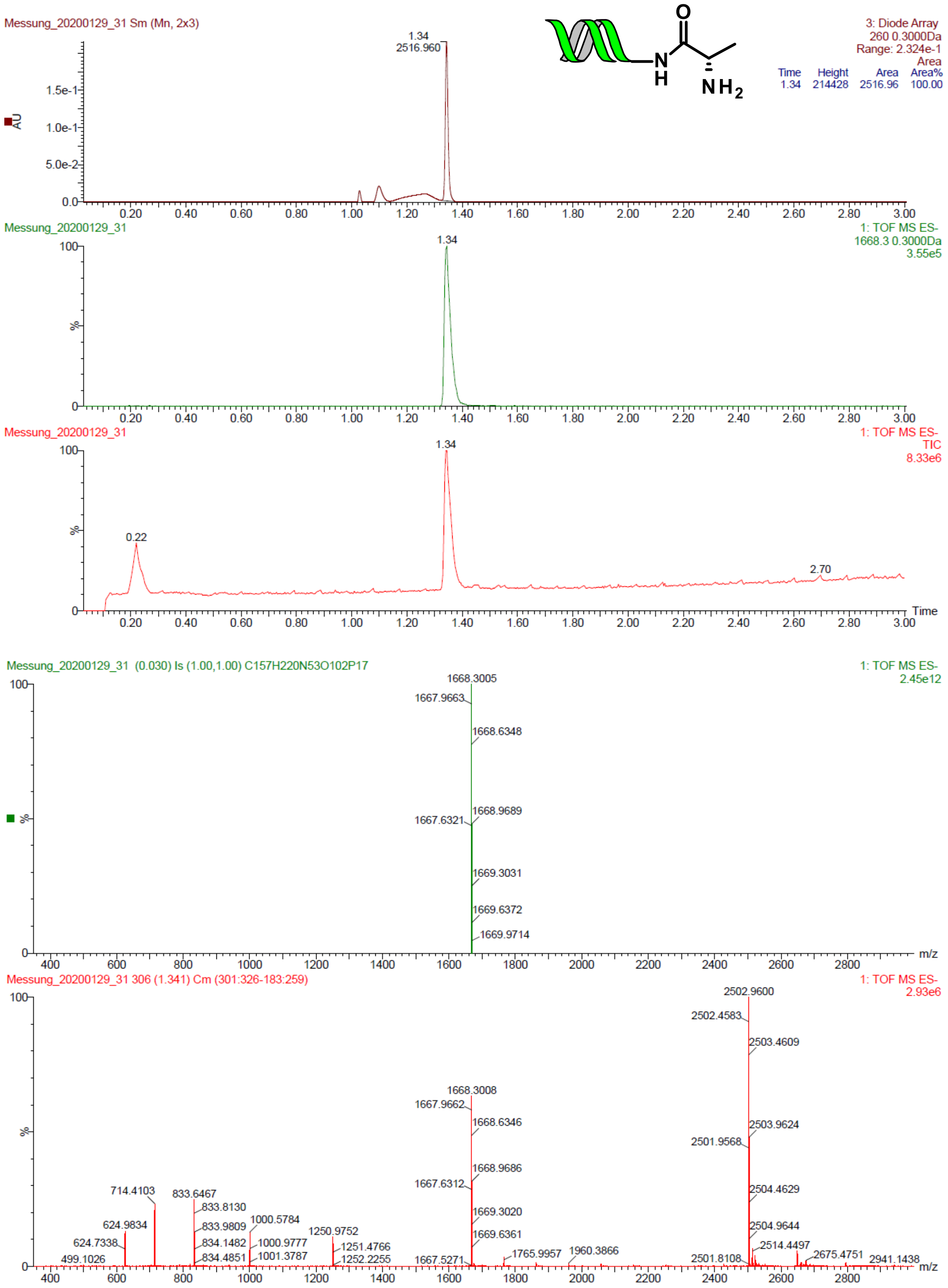

*Figure 3.18: LC-MS chromatogram of compound **Table 2 Entry 8 amine** t_R = 1,34 min TOF-MS-ESI$^-$ m/z = 1668,301(100%) $[M-3H]^{3-}$ (calc. 1668,301 for $C_{157}H_{220}N_{53}O_{102}P_{17}$)*

Azido-Ala-OH conjugated with HP-280 (Table 2 Entry 8):

The reaction was carried out by using the general method DTR 1 (1 µl, 10 mM of **(Table 2 Entry 8 amine)**, 10nmol scale)

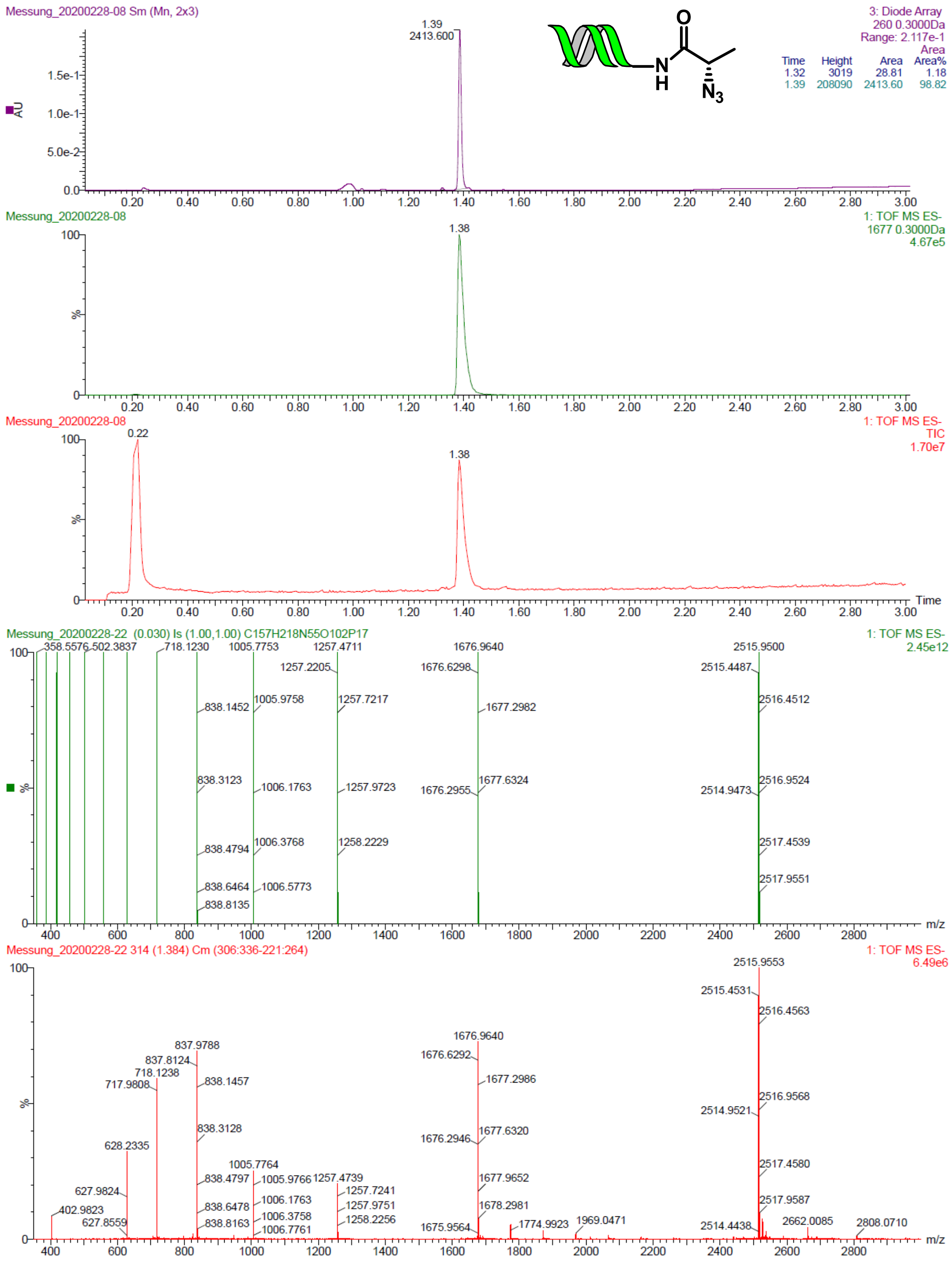

Figure 3.19: LC-MS chromatogram of compound ***Table 2 Entry 8*** *t_R = 1,38 min TOF-MS-ESI$^-$ m/z = 1676,964(100%) [M-3H]$^{3-}$ (calc. 1674,964 for $C_{157}H_{218}N_{55}O_{102}P_{17}$)*

3.3.10 Azido-α-methylalanine conjugated with HP-280 (Table 2 Entry 9)

H-α-Methylalanine conjugated with HP-280:

Fmoc-α-methylalanine (CAS 94744-50-0) was conjugated using general method **ABF 1** (15 µl, 10 mM HP-280, 150 nmol scale) and purified with general purification method **GP1**. The Fmoc deprotection was carried out using general method for Fmoc deprotection and was purified using the methods **GP1** and **GP2.**

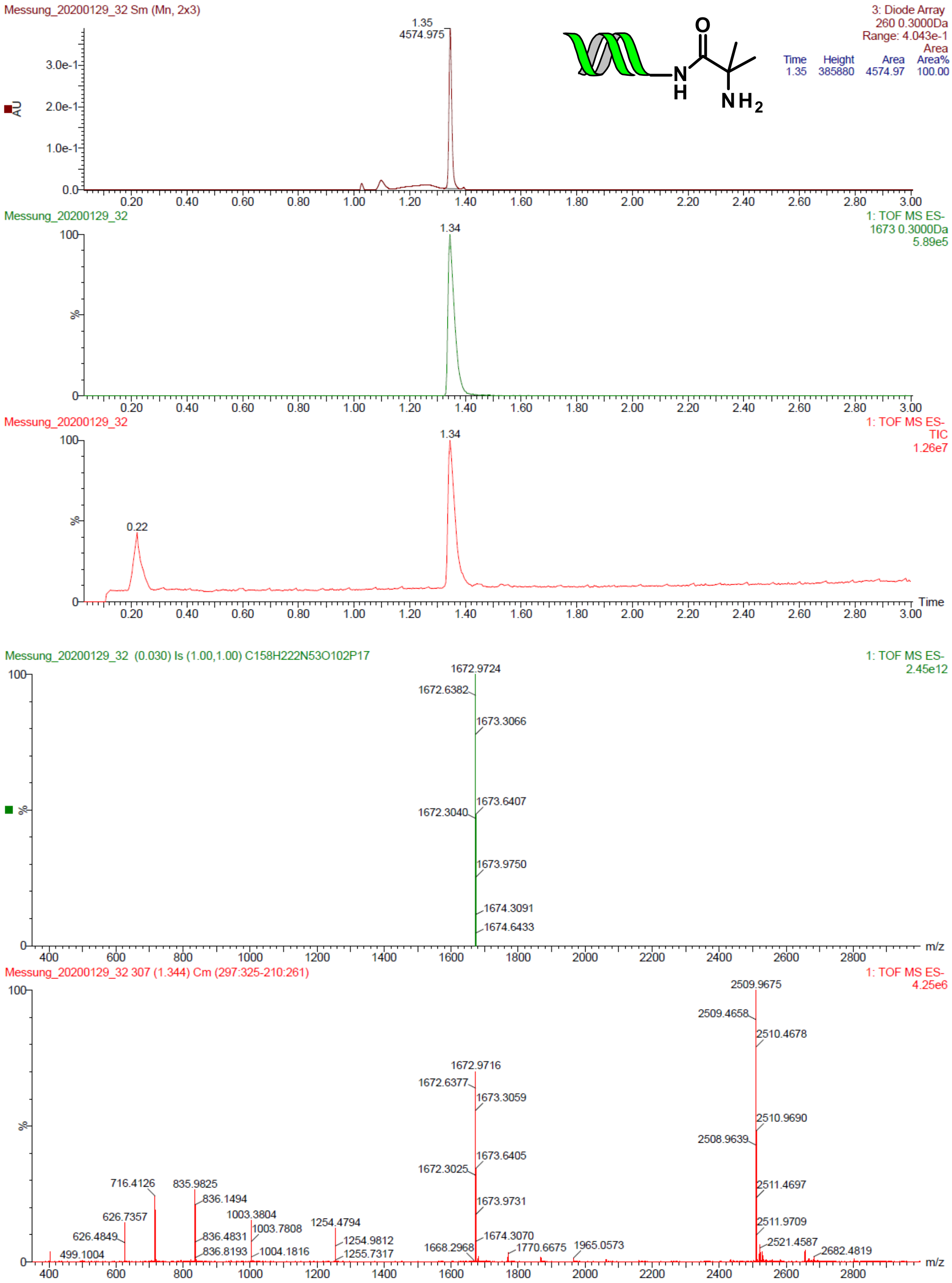

*Figure 3.20: LC-MS chromatogram of compound **Table 2 Entry 9 amine** t_R = 1,35 min TOF-MS-ESI⁻ m/z = 1692,964(100%) [M-3H]$^{3-}$ (calc. 1692,964 for $C_{158}H_{222}N_{53}O_{102}P_{17}$)*

Azido-α-methylalanine conjugated with HP-280 (Table 2 Entry 9):

The reaction was carried out by using the general method DTR 1 (1 µl, 10 mM of **(Table 2 Entry 9 amine)**, 10nmol scale).

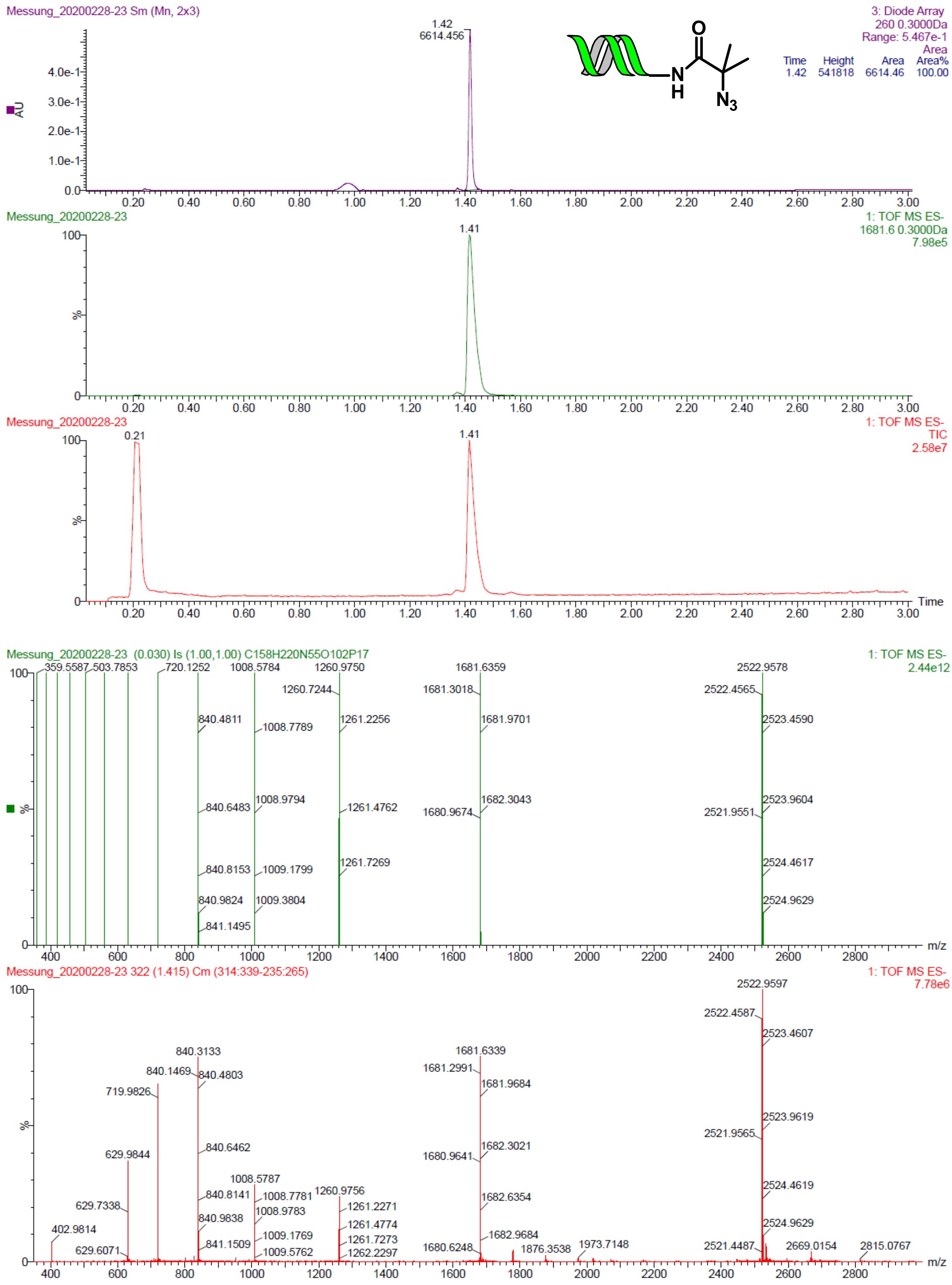

*Figure 3.21: LC-MS chromatogram of compound **Table 2 Entry 9** t_R = 1,41 min TOF-MS-ESI⁻ m/z = 1681,634(100%) $[M-3H]^{3-}$ (calc. 1681,636 for $C_{158}H_{220}N_{55}O_{102}P_{17}$)*

3.3.11 Azidocycloleucine conjugated with HP-280 (Table 2 Entry 10)

Cycloleucine conjugated with HP-280:

Fmoc-cycloleucine (CAS 52-52-8) was conjugated using general method **ABF 1** (15 µl, 10 mM HP-280, 150 nmol scale) and purified with general purification method **GP1**. The Fmoc deprotection was carried out using general method for Fmoc deprotection and was purified using the methods **GP1** and **GP2**.

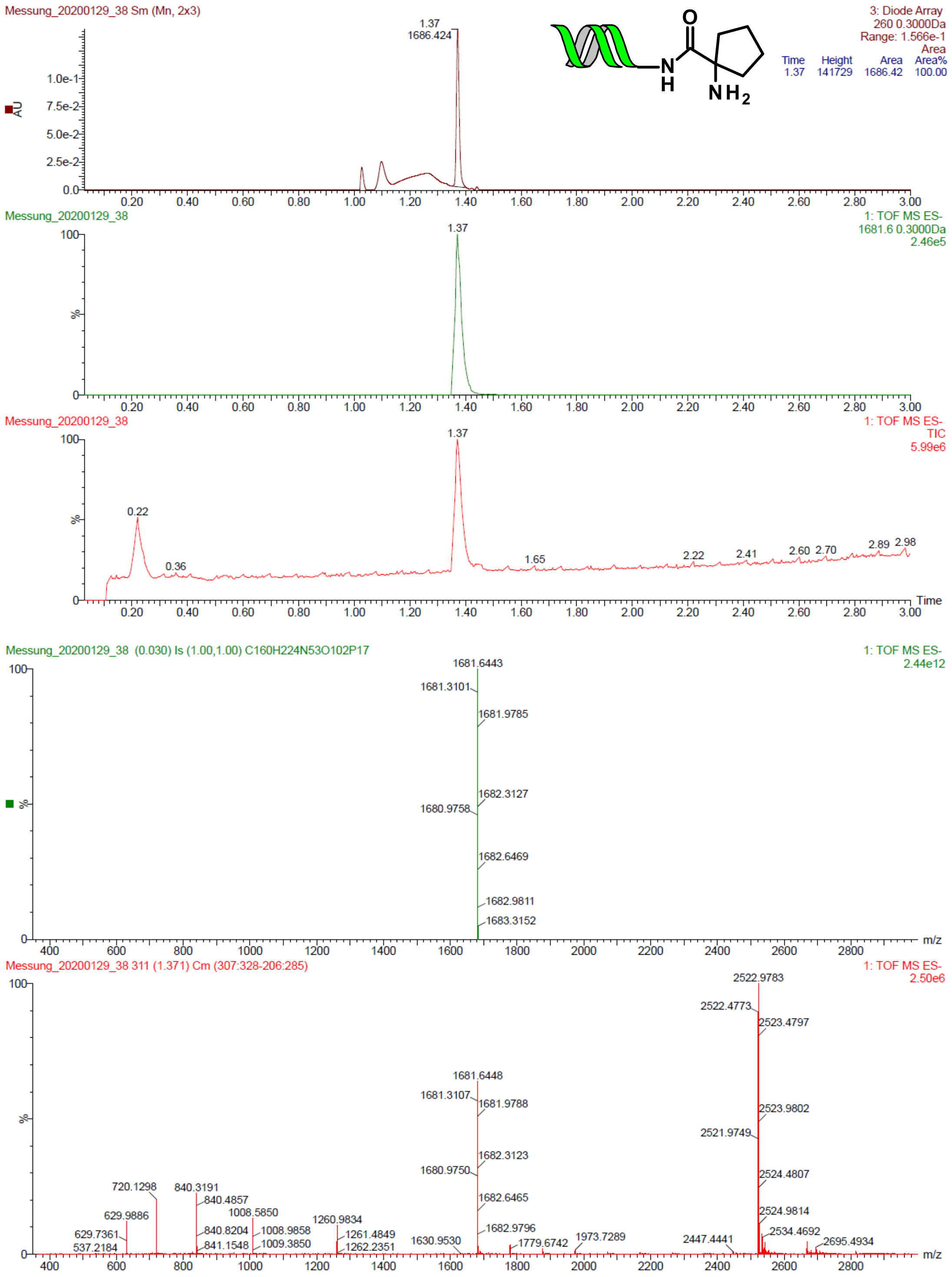

*Figure 3.22: LC-MS chromatogram of compound **Table 2 Entry 10 amine** t_R = 1,37 min TOF-MS-ESI$^-$ m/z = 1681,644(100%) [M-3H]$^{3-}$ (calc. 1681,644 for $C_{161}H_{218}N_{55}O_{102}P_{17}$)*

Azidocycloleucine conjugated with HP-280 (Table 2 Entry 10):

The reaction was carried out by using the general method DTR 1 (1 µl, 10 mM of **(Table 2 Entry 10 amine)**, 10nmol scale).

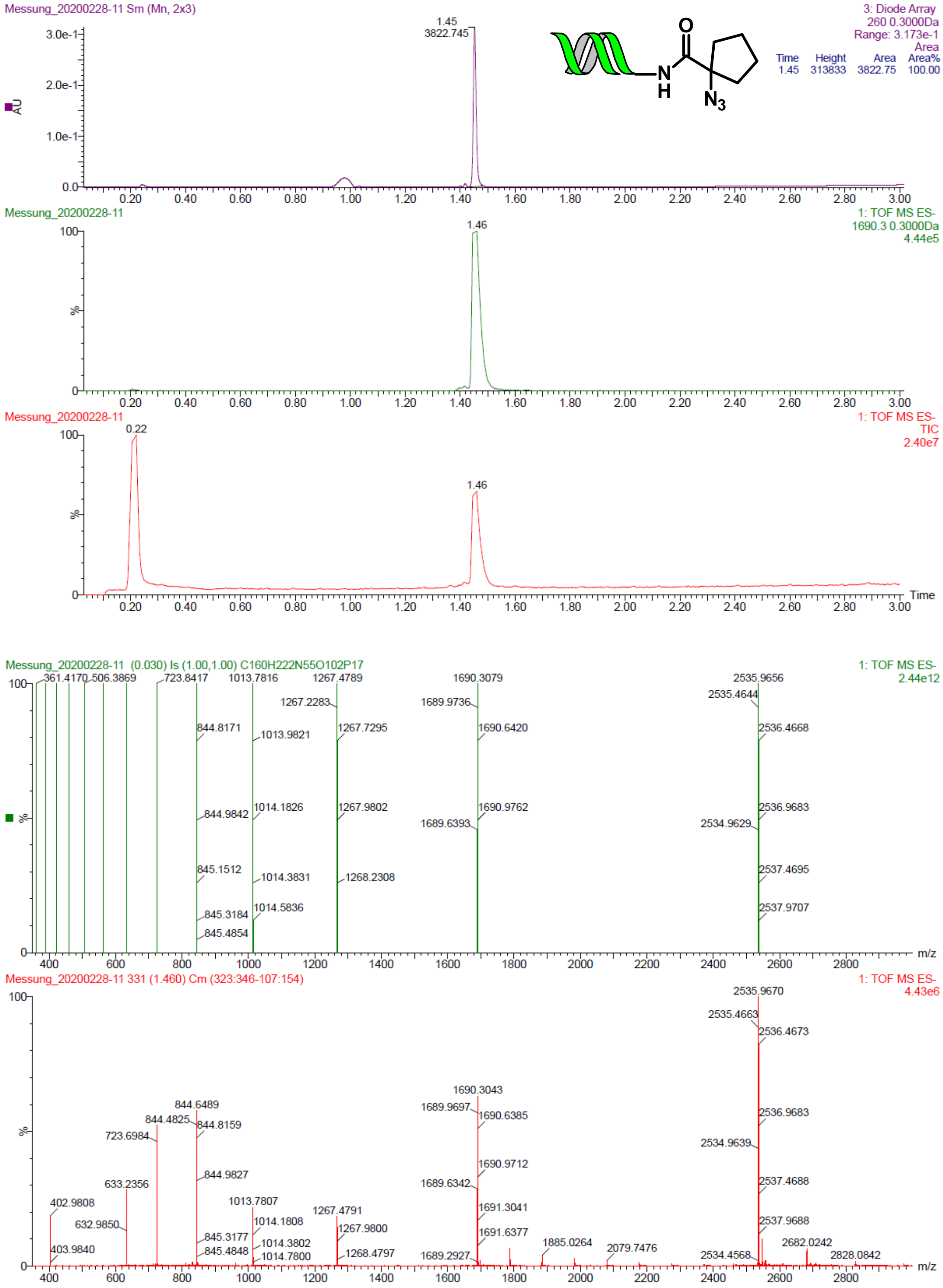

*Figure 3.23: LC-MS chromatogram of compound **Table 2 Entry 10** t_R = 1,45 min TOF-MS-ESI$^-$ m/z = 1690,304(100%) $[M-3H]^{3-}$ (calc. 1690,308 for $C_{161}H_{218}N_{55}O_{102}P_{17}$)*

3.3.12 Azido-propargylglycine conjugated with HP-280 (Table 2 Entry 11)

Propargylglycine conjugated with HP-280:

Fmoc-propargylglycine (CAS 198561-07-8) was conjugated using general method **ABF 1** (15 µl, 10 mM HP-280, 150 nmol scale) and purified with general purification method **GP1**. The Fmoc deprotection was carried out using general method for Fmoc deprotection and was purified using the methods **GP1** and **GP2**.

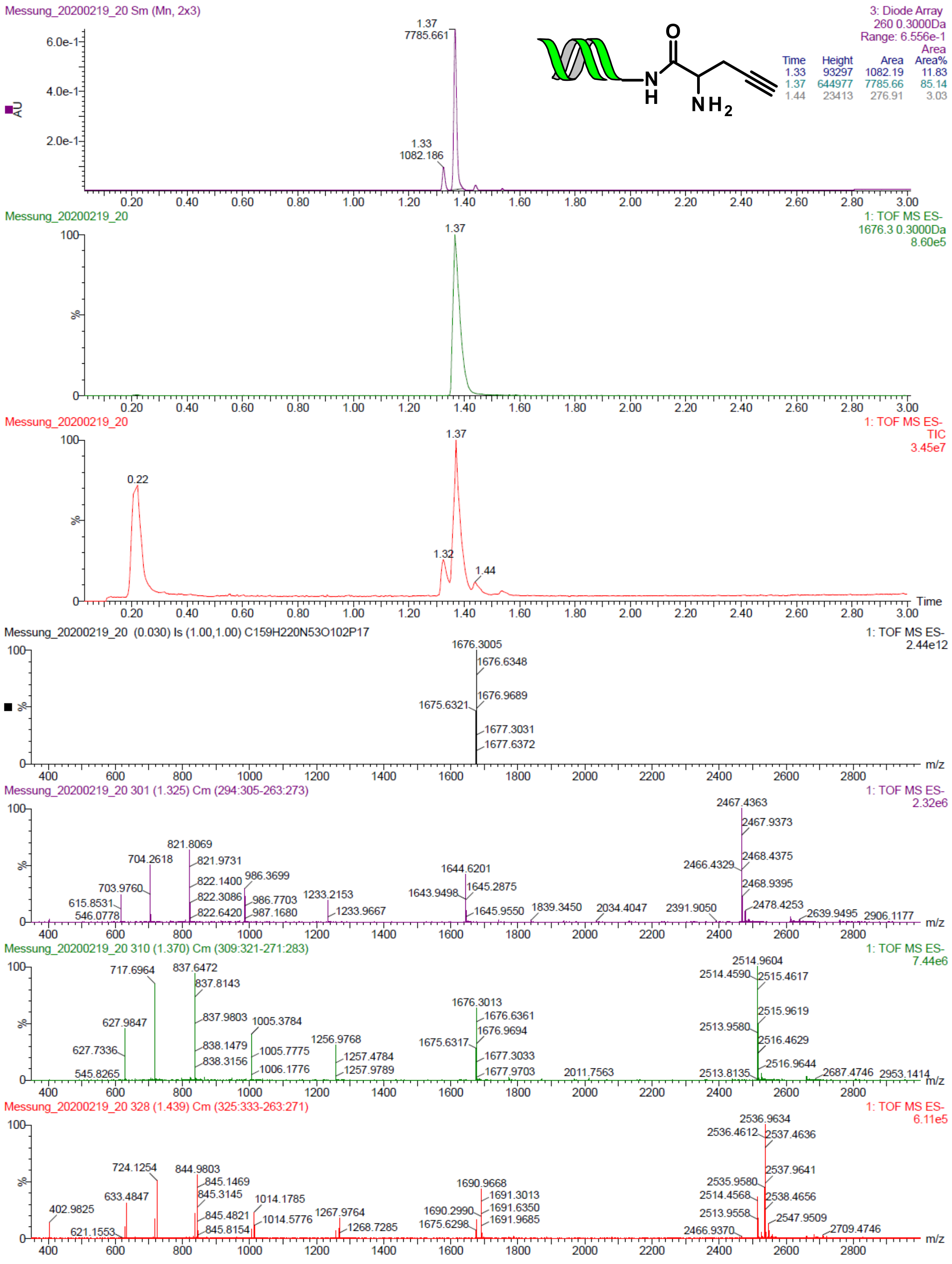

*Figure 3.24: LC-MS chromatogram of compound **Table 2 Entry 11 amine** t_R = 1,37 min TOF-MS-ESI$^-$ m/z = 1676,301(100%) [M-3H]$^{3-}$ (calc. 1676,301 for $C_{159}H_{220}N_{53}O_{102}P_{17}$)*

Azido-propargylglycine conjugated with HP-280 (Table 2 Entry 11):

The reaction was carried out by using the general method DTR 1 (1 µl, 10 mM of **(Table 2 Entry 11 amine)**, 10nmol scale).

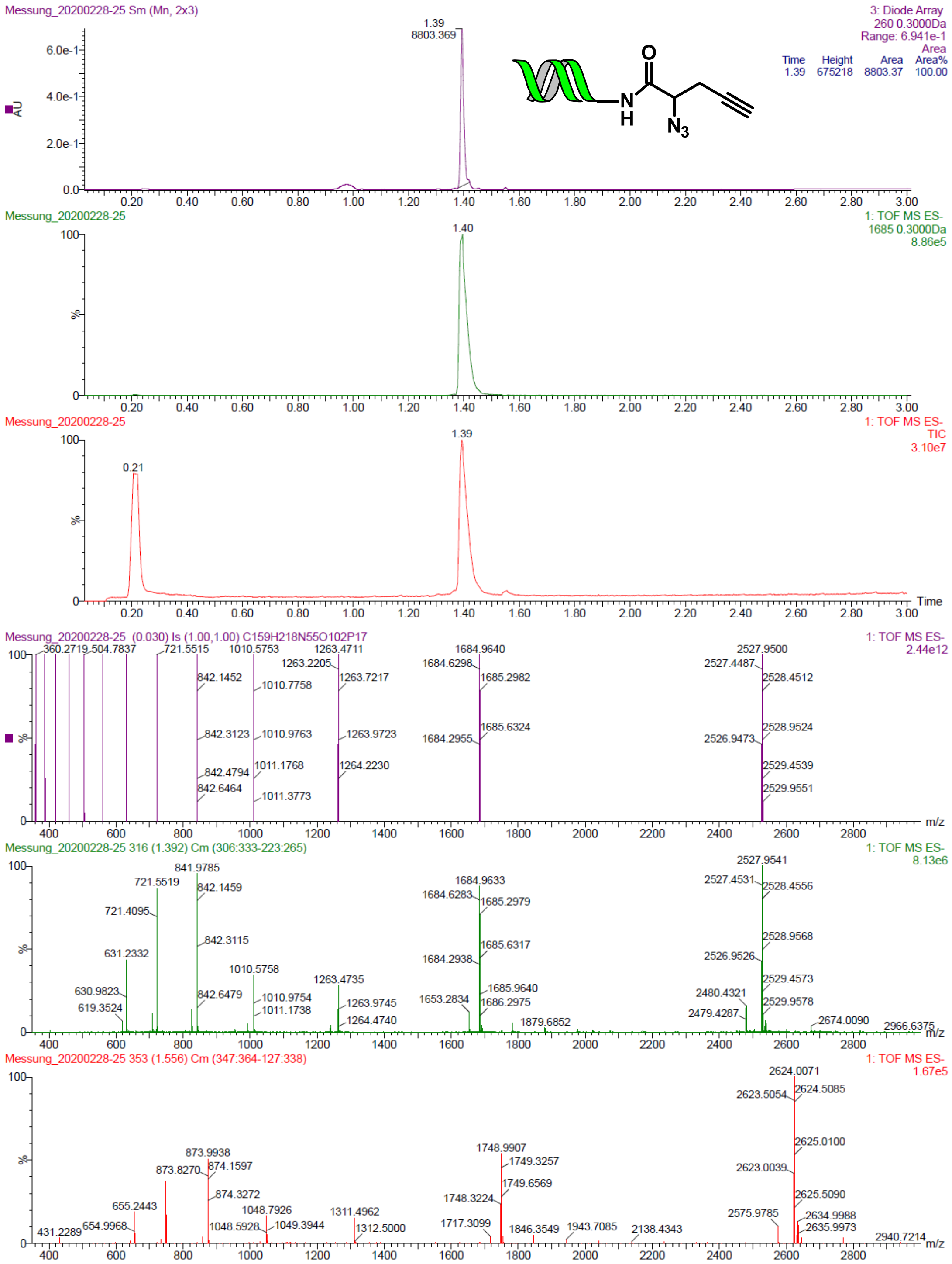

Figure 3.25: LC-MS chromatogram of compound ***Table 2 Entry 11*** *t_R = 1,39 min TOF-MS-ESI$^-$ m/z = 1684,963(100%) [M-3H]$^{3-}$ (calc. 1684,964 for $C_{159}H_{218}N_{55}O_{102}P_{17}$)*

3.3.13 Azido-Lys(Alloc)-OH conjugated with HP-280 (Table 2 Entry 12)

H-Lys(Alloc)-OH conjugated with HP-280:

Fmoc-Lys(Alloc)-OH (CAS 146982-27-6) was conjugated using general method **ABF 1** (15 µl, 10 mM HP-280, 150 nmol scale) and purified with general purification method **GP1**. The Fmoc deprotection was carried out using general method for Fmoc deprotection and was purified using the methods **GP1** and **GP2**.

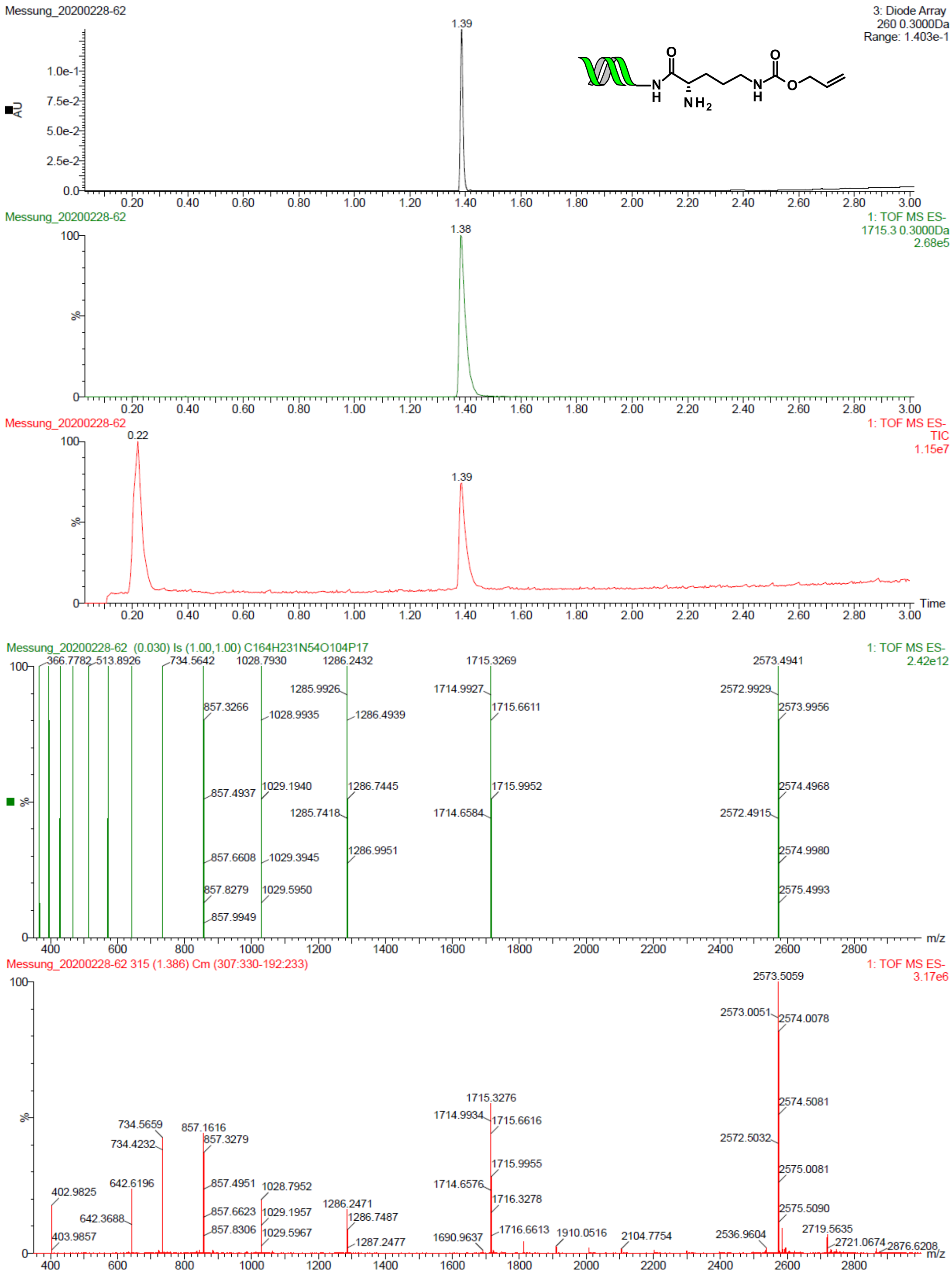

Figure 3.26: LC-MS chromatogram of compound ***Table 2 Entry 14*** *t_R = 1,39 min TOF-MS-ESI$^-$ m/z = 1715,327(100%) $[M-3H]^{3-}$ (calc. 1715,327 for $C_{164}H_{231}N_{54}O_{104}P_{17}$)*

Azido-Lys(Alloc)-OH conjugated with HP-280 (Table 2 Entry 12):

The reaction was carried out by using the general method DTR 1 (1 µl, 10 mM of **(Table 2 Entry 12 amine)**, 10nmol scale).

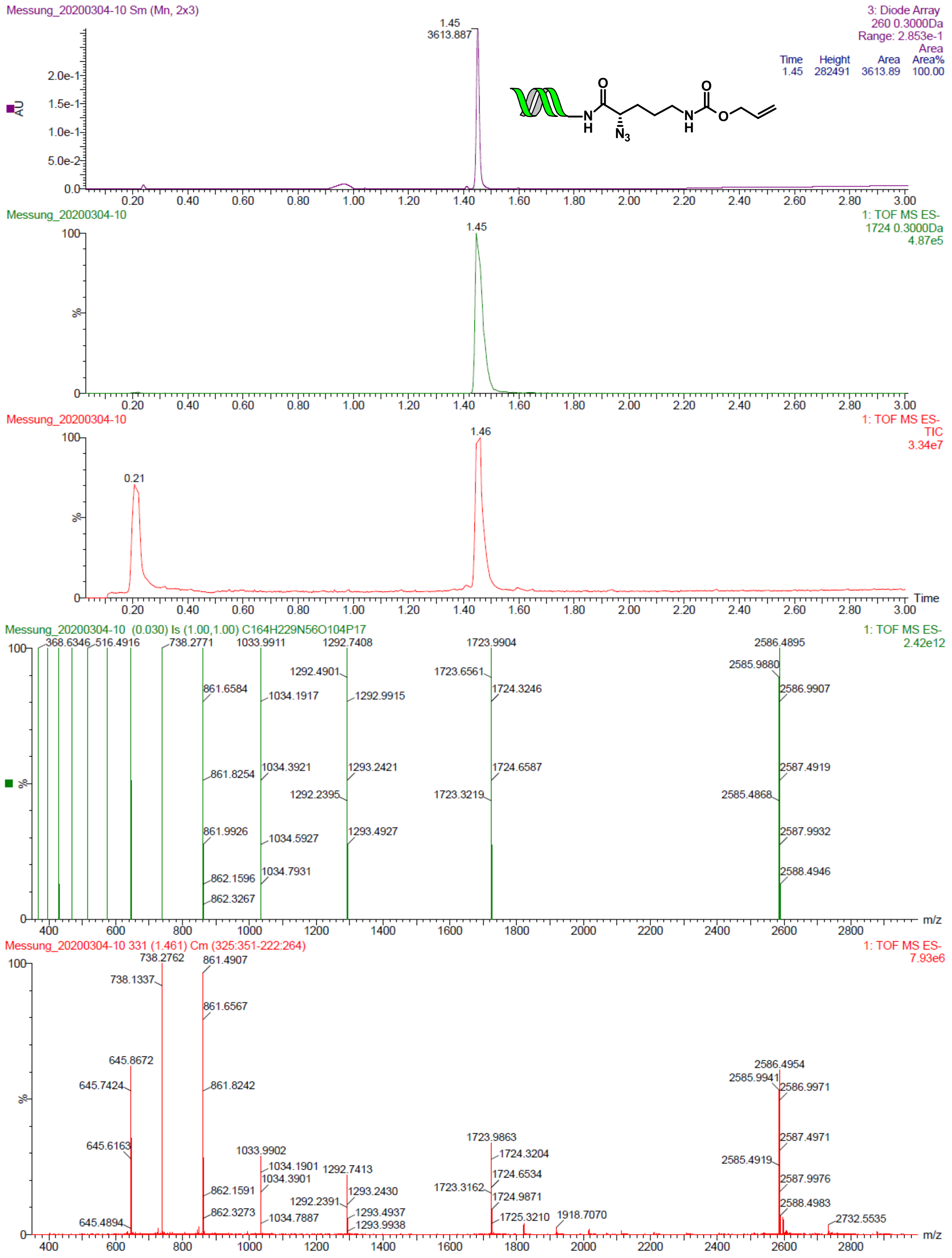

*Figure 3.27: LC-MS chromatogram of compound **Table 2 Entry 12 amine** t_R = 1,45 min TOF-MS-ESI$^-$ m/z = 1723,986 (100%) [M-3H]$^{3-}$ (calc. 1723,990 for $C_{164}H_{229}N_{56}O_{104}P_{17}$)*

3.3.14 Azido-Gln-OH conjugated with HP-280 (Table 2 Entry 13)

H-Gln-OH conjugated with HP-280:

Fmoc-Gln-OH (CAS 71989-20-3) was conjugated using general method **ABF 1** (15 µl, 10 mM HP-280, 150 nmol scale) and purified with general purification method **GP1**. The Fmoc deprotection was carried out using general method for Fmoc deprotection and was purified using the methods **GP1** and **GP2**.

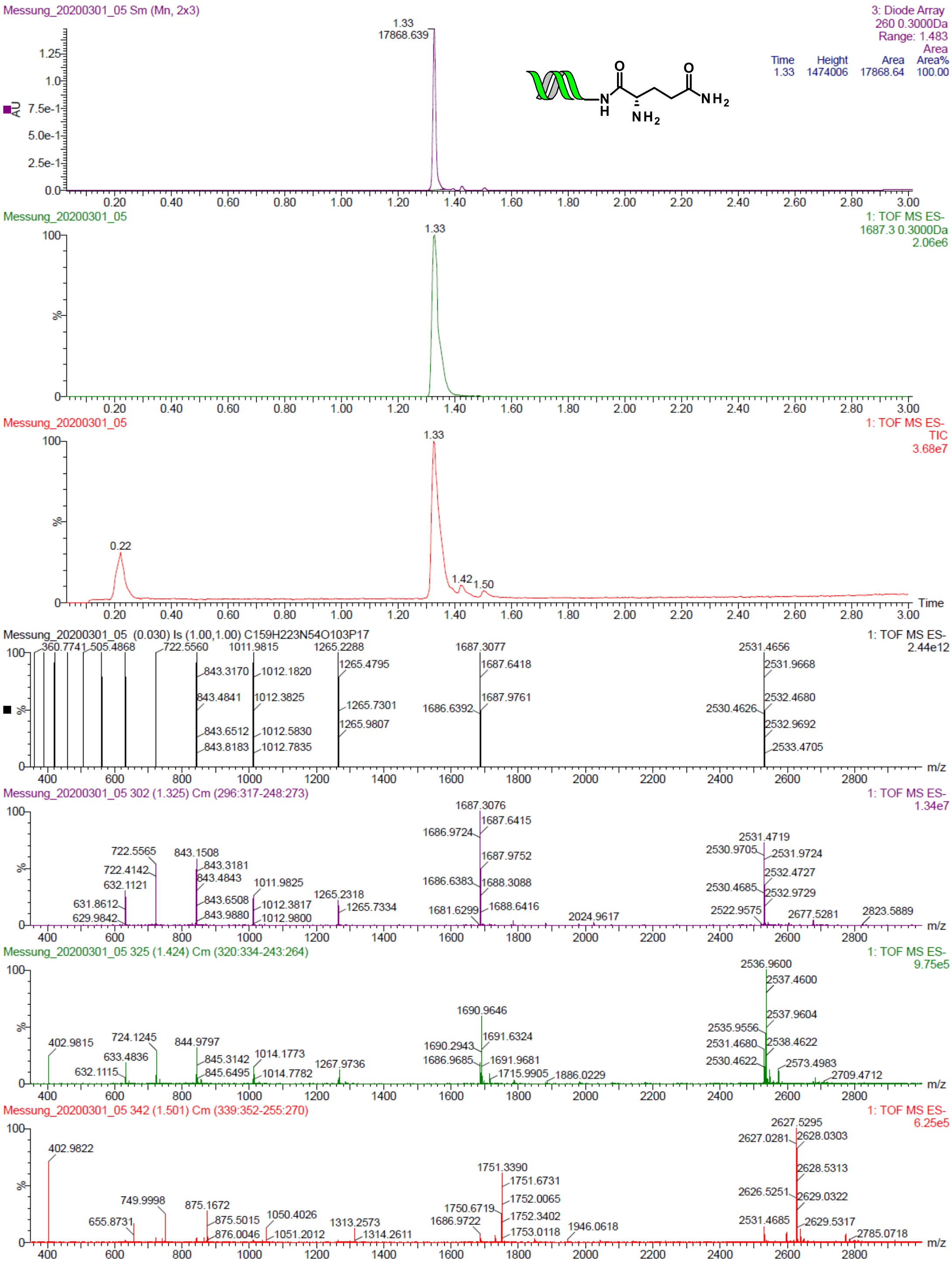

Figure 3.28: LC-MS chromatogram of compound ***Table 2 Entry 12*** *t_R = 1,33 min TOF-MS-ESI$^-$ m/z = 1723,986 (100%) $[M-3H]^{3-}$ (calc. 1723,990 for $C_{164}H_{229}N_{56}O_{104}P_{17}$)*

Azido-Gln-OH conjugated with HP-280 (Table 2 Entry 13):

The reaction was carried out by using the general method DTR 1 (1 µl, 10 mM of **(Table 2 Entry 13 amine)**, 10nmol scale).

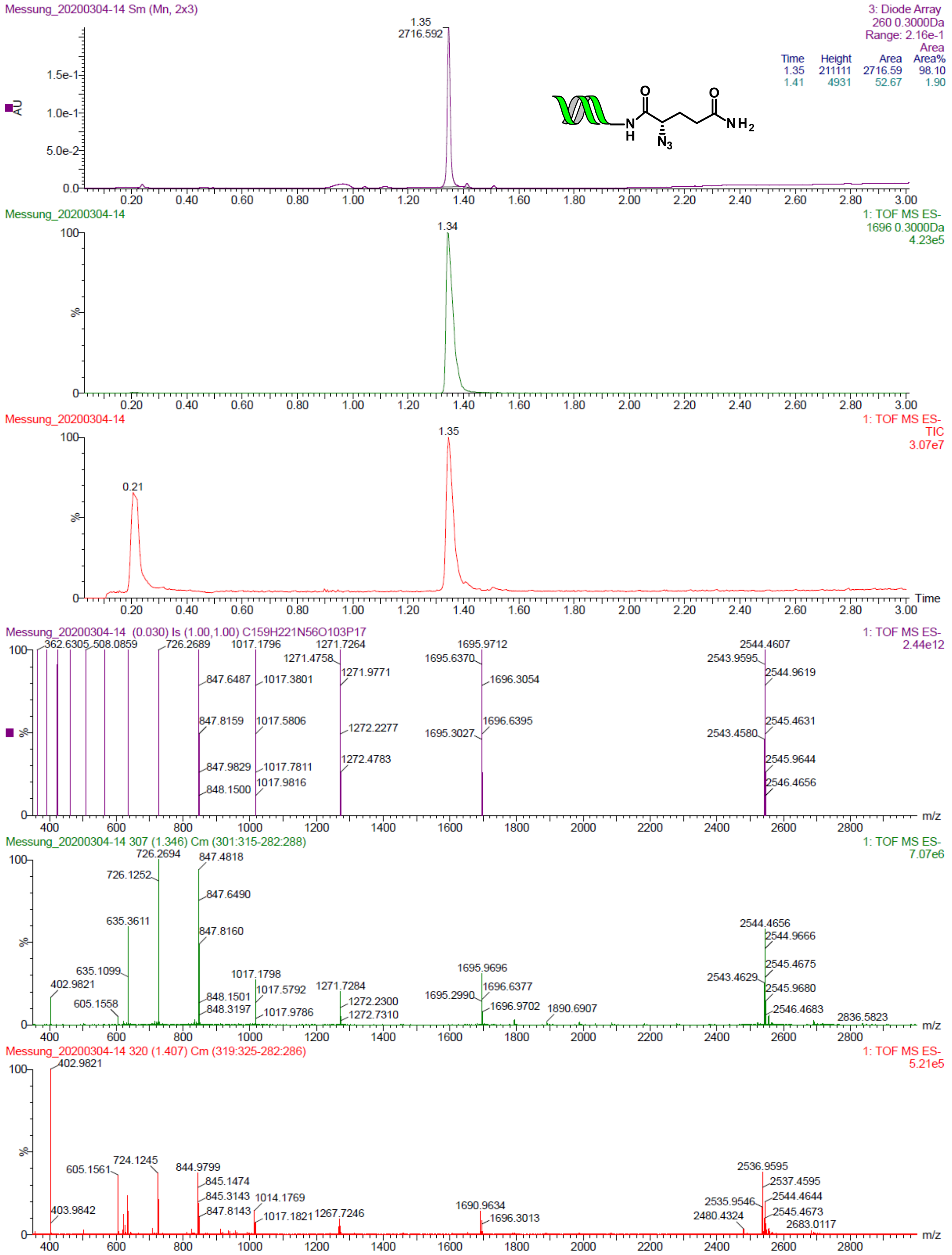

Figure 3.29: LC-MS chromatogram of compound ***Table 2 Entry 13 amine*** *t_R = 1,33 min TOF-MS-ESI$^-$ m/z = 1695,970 (100%) [M-3H]$^{3-}$ (calc. 1695,971 for $C_{159}H_{223}N_{56}O_{103}P_{17}$)*

3.3.15 Azido-Phe(4-Br)-OH conjugated with HP-280 (Table 2 Entry 14)

H-Phe(4-Br)-OH conjugated with HP-280:

Fmoc-Phe(4-Br)-OH (CAS 198561-04-5) was conjugated using general method **ABF 1** (15 µl, 10 mM HP-280, 150 nmol scale) and purified with general purification method **GP1**. The Fmoc deprotection was carried out using general method for Fmoc deprotection and was purified using the methods **GP1** and **GP2**.

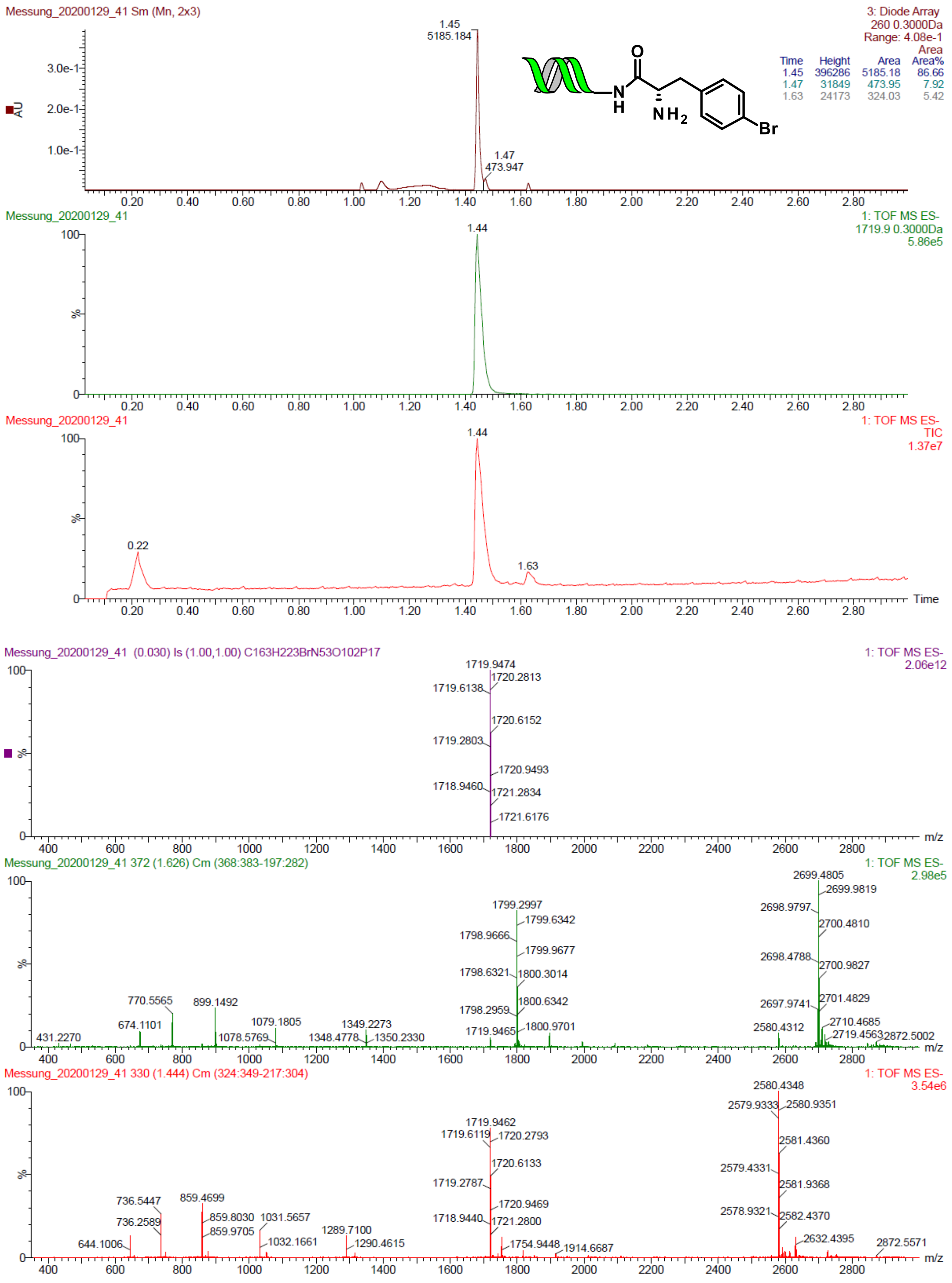

*Figure 3.30: LC-MS chromatogram of compound **Table 2 Entry 14 amine** t_R = 1,45 min TOF-MS-ESI$^-$ m/z = 1719,946(100%) $[M\text{-}3H]^{3-}$ (calc. 1719,947 for $C_{163}H_{223}BrN_{53}O_{102}P_{17}$)*

Azido-Phe(4-Br)-OH conjugated with HP-280 (Table 2 Entry 14):

The reaction was carried out by using the general method DTR 1 (1 µl, 10 mM of **(Table 2 Entry 14 amine)**, 10nmol scale).

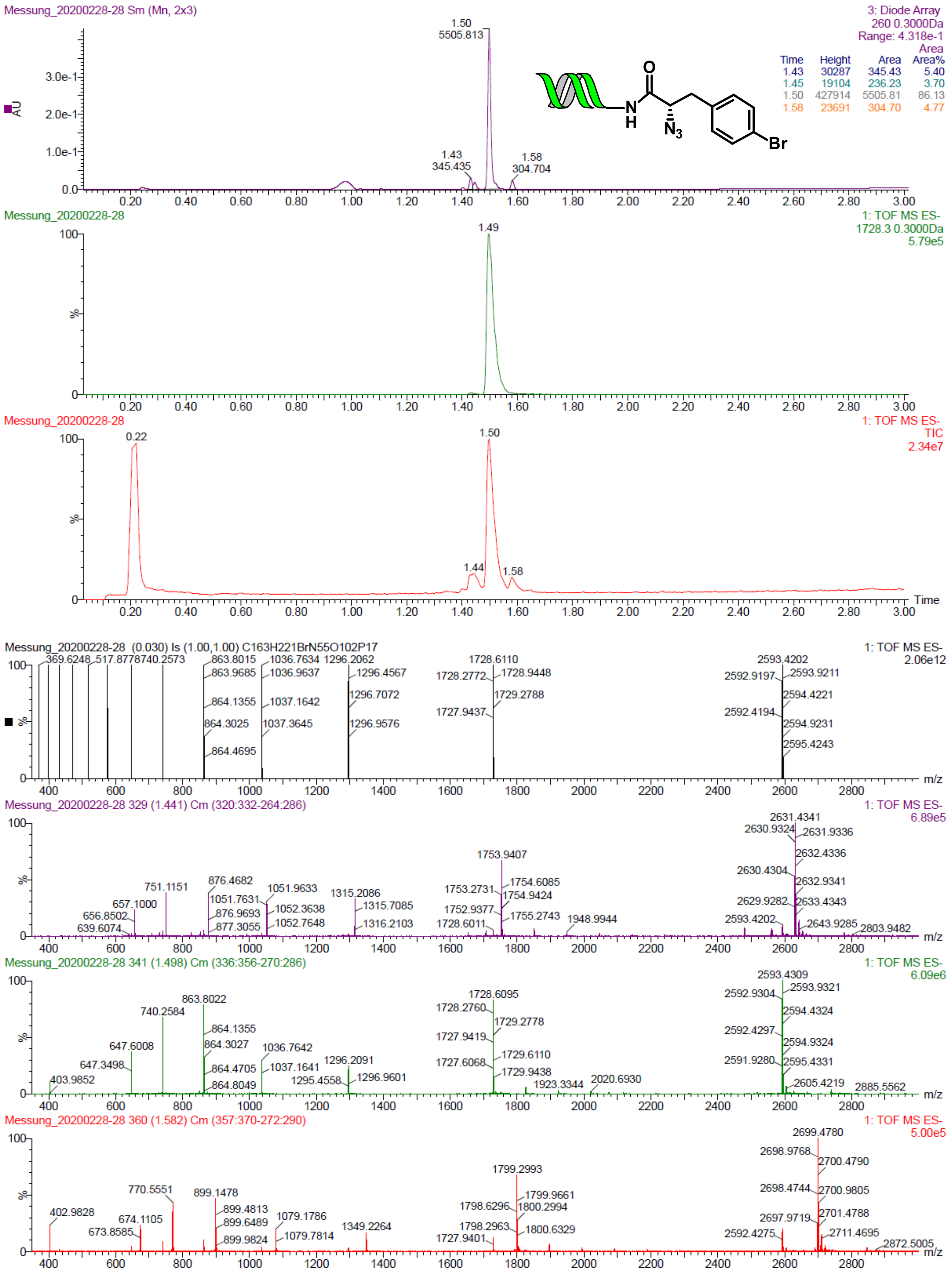

Figure 3.31: LC-MS chromatogram of compound ***Table 2 Entry 14*** *t_R = 1,50min TOF-MS-ESI$^-$ m/z = 1728,610(100%) [M-3H]$^{3-}$ (calc. 1728,611 for $C_{163}H_{221}BrN_{55}O_{102}P_{17}$)*

3.3.16 Azido-Tyr-OH conjugated with HP-280 (Table 2 Entry 15)

H-Tyr-OH conjugated with HP-280:

Fmoc-Tyr-OH (CAS 92954-90-0) was conjugated using general method **ABF 1** (15 µl, 10 mM HP-280, 150 nmol scale) and purified with general purification method **GP1**. The Fmoc deprotection was carried out using general method for Fmoc deprotection and was purified using the methods **GP1** and **GP2**.

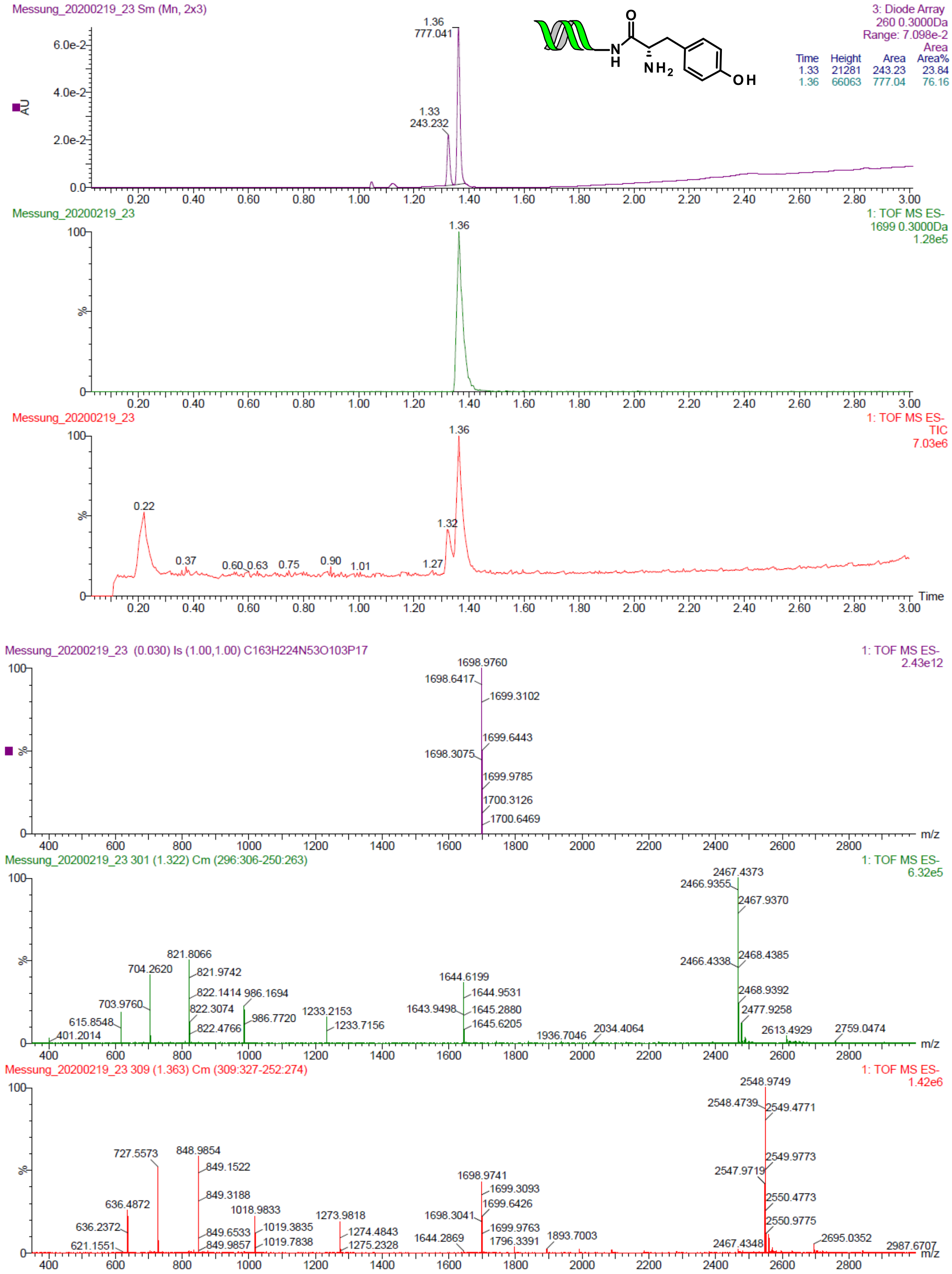

Figure 3.32: LC-MS chromatogram of compound ***Table 2 Entry 15 amine*** *t_R = 1,46 min TOF-MS-ESI$^-$ m/z = 1692,964(100%) [M-3H]$^{3-}$ (calc. 1692,964 for $C_{161}H_{218}N_{55}O_{102}P_{17}$)*

Azido-Tyr-OH conjugated with HP-280 (Table 2 Entry 15):

The reaction was carried out by using the general method DTR 1 (1 µl, 10 mM of **(Table 2 Entry 15 amine)**, 10nmol scale).

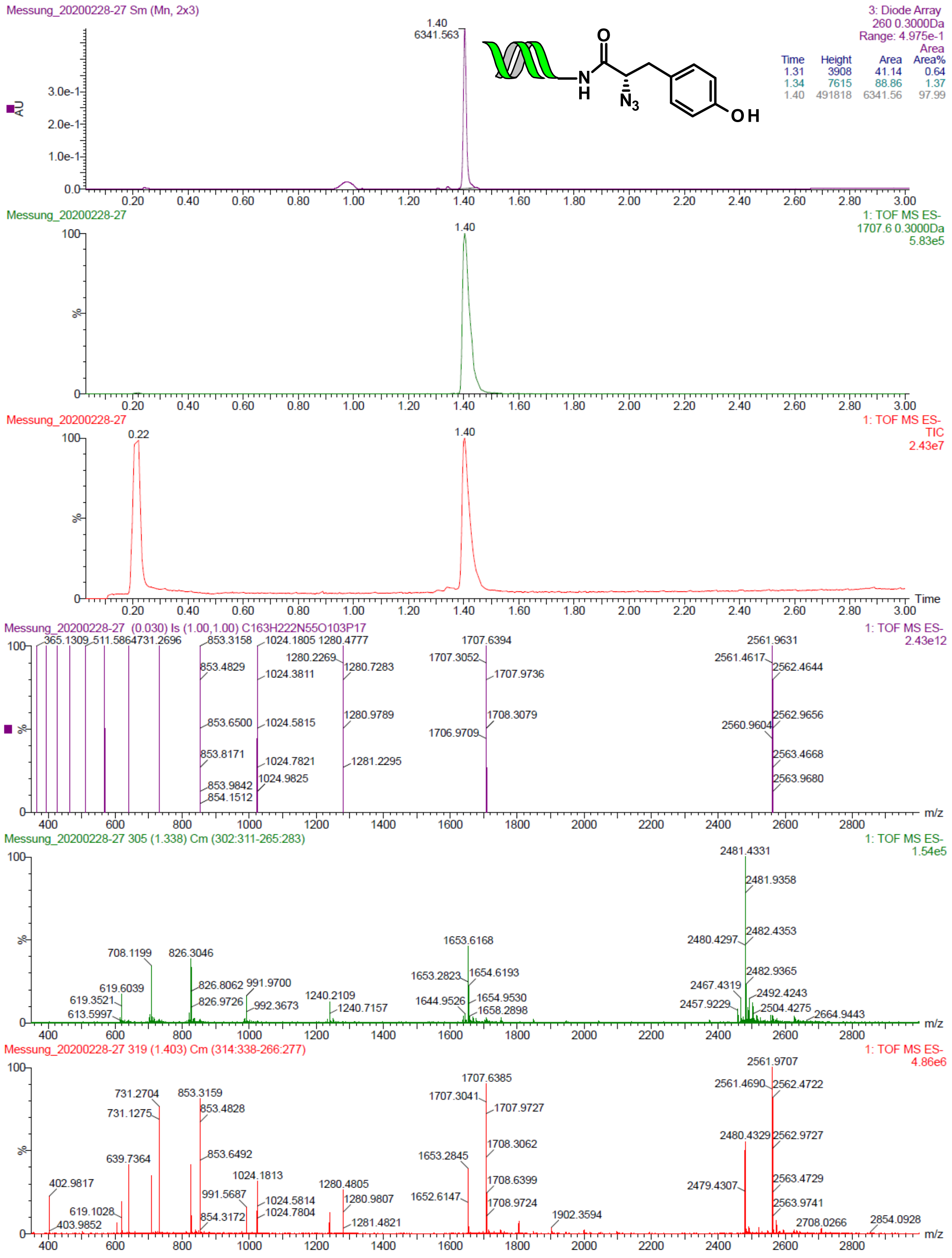

*Figure 3.33: LC-MS chromatogram of compound **Table 2 Entry 15** t_R = 1,40 min TOF-MS-ESI⁻ m/z = 1707,639(100%) $[M-3H]^{3-}$ (calc. 1707,639 for $C_{161}H_{218}N_{55}O_{102}P_{17}$)*

3.3.17 Azido-Phe(4-N-Boc)-OH conjugated with HP-280 (Table 2 Entry 16)

H-Phe(4-N-Boc)-OH conjugated with HP-280

Fmoc-Phe(4-N-Boc)-OH (CAS 174132-31-1) was conjugated using general method **ABF 1** (15 µl, 10 mM HP-280, 150 nmol scale) and purified with general purification method **GP1**. The Fmoc deprotection was carried out using general method for Fmoc deprotection and was purified using the methods **GP1** and **GP2**.

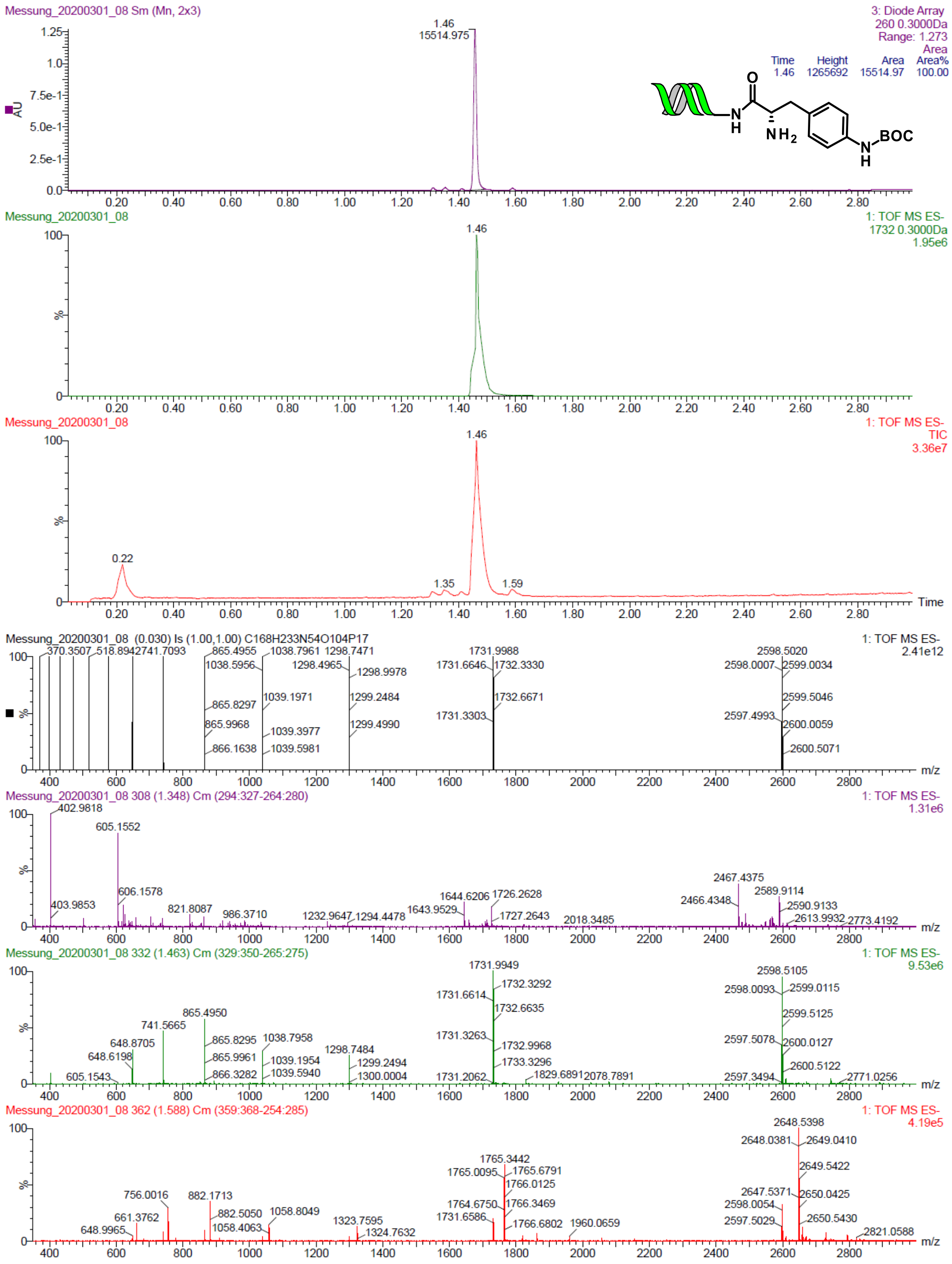

Figure 3.34: LC-MS chromatogram of compound ***Table 2 Entry 16 amine*** *t_R = 1.46 min TOF-MS-ESI$^-$ m/z = 1731.995(100%) [M-3H]$^{3-}$ (calc. 1731.999 for $C_{168}H_{233}N_{54}O_{104}P_{17}$)*

Azido-Phe(4-N-Boc)-OH conjugated with HP-280 (Table 2 Entry 16)

The reaction was carried out by using the general method DTR 1 (1 µl, 10 mM of **(Table 2 Entry 16 amine)**, 10nmol scale).

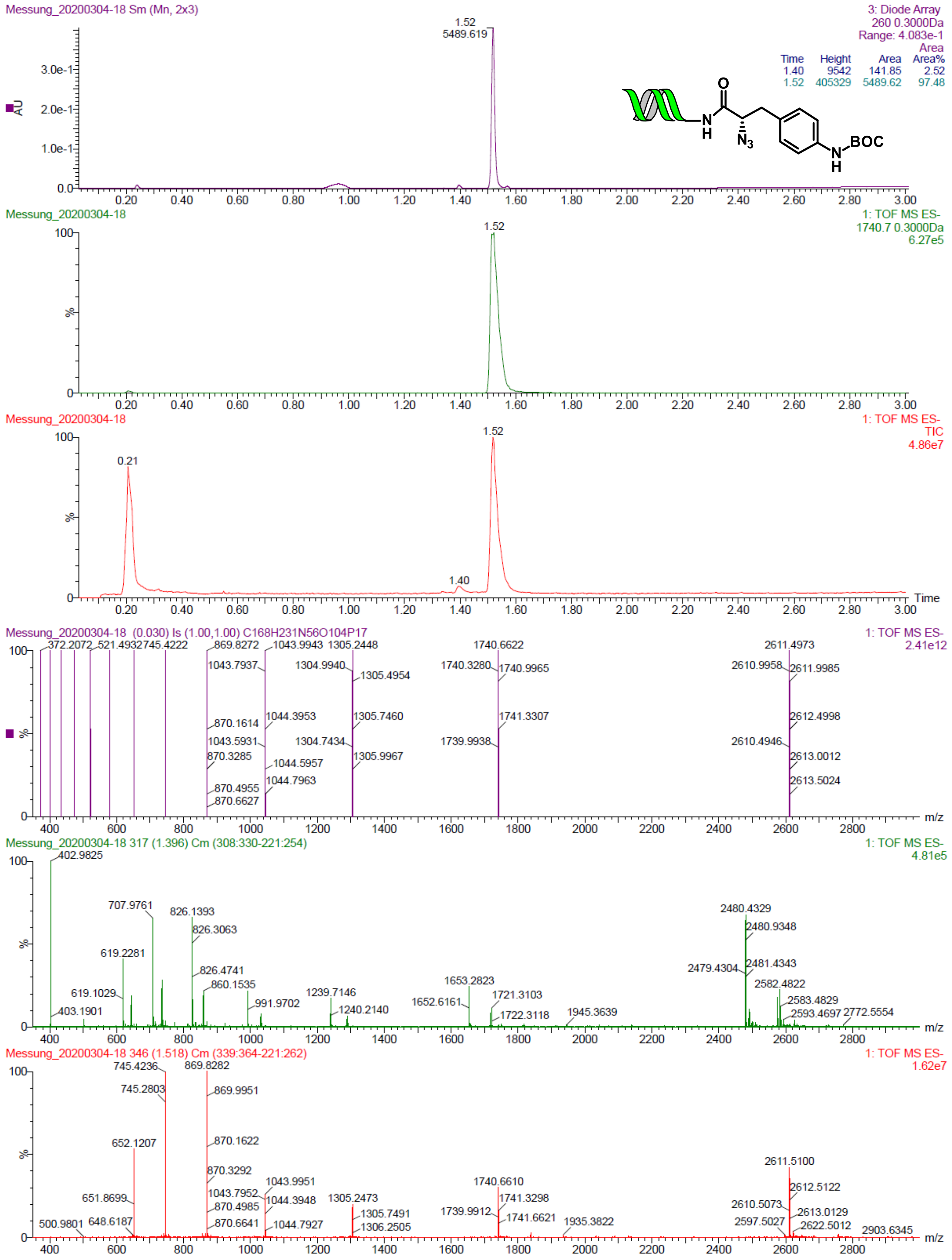

*Figure 3.35: LC-MS chromatogram of compound **Table 2 Entry 16** t_R = 1.52 min TOF-MS-ESI$^-$ m/z = 1740.661(100%) $[M-3H]^{3-}$ (calc. 1740.662 for $C_{168}H_{231}N_{56}O_{104}P_{17}$)*

3.3.18 Boc-Phe(4-azido)-OH conjugated with HP-280 (Table 2 Entry 17)

Boc-Phe(4-NH_2)-OH conjugated with HP-280

Boc-Phe(Fmoc)-OH (CAS 114346-31-5) was conjugated using general method **ABF 1** (15 µl, 10 mM HP-280, 150 nmol scale) and purified with general purification method **GP1**. The Fmoc deprotection was carried out using general method for Fmoc deprotection and was purified using the methods **GP1** and **GP2**.

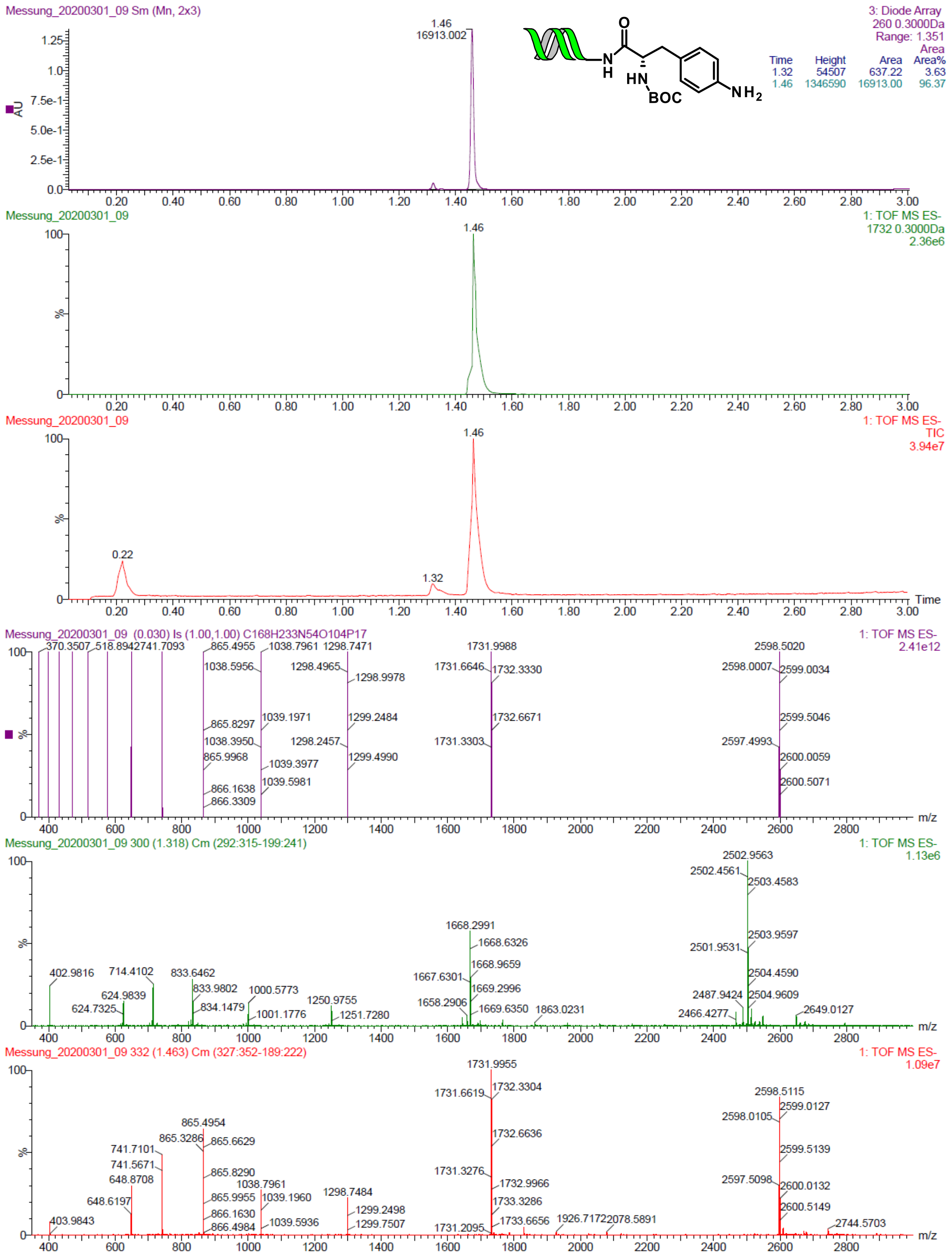

Figure 3.36: LC-MS chromatogram of compound ***Table 2 Entry 17 amine*** *t_R = 1.70 min TOF-MS-ESI$^-$ m/z = 1806.019(100%) $[M-3H]^{3-}$ (calc. 1806.022 for $C_{183}H_{243}N_{54}O_{106}P_{17}$)*

Boc-Phe(4-Azido)-OH conjugated with HP-280 (Table 2 Entry 17)

The reaction was carried out by using the general method DTR 1 (1 µl, 10 mM of **(Table 2 Entry 17 amine)**, 10nmol scale).

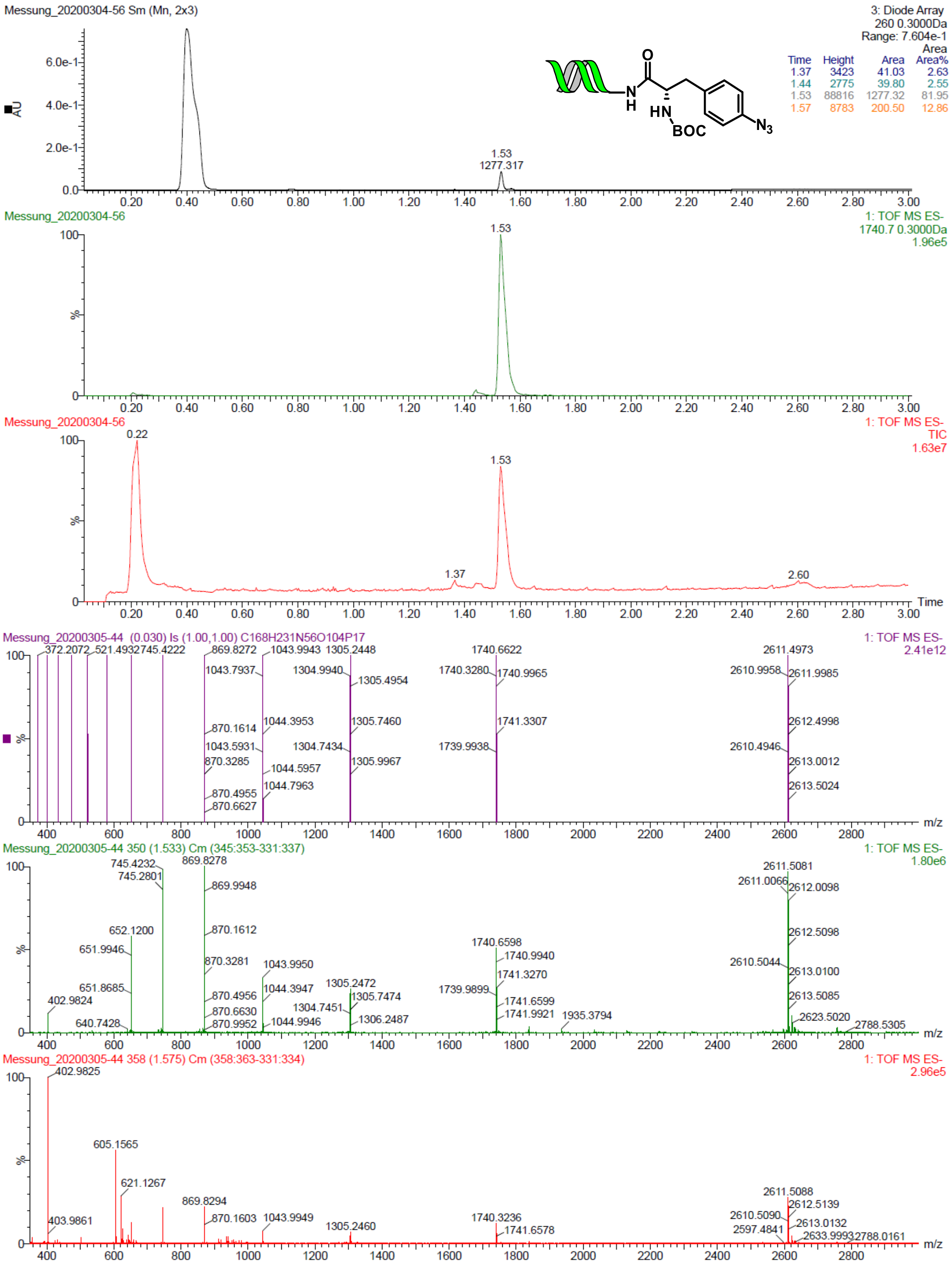

Figure 3.37: LC-MS chromatogram of compound ***Table 2 Entry 17*** *t_R = 1.53 min TOF-MS-ESI$^-$ m/z = 1740.660(100%) $[M-3H]^{3-}$ (calc. 1740.662 for $C_{168}H_{231}N_{56}O_{104}P_{17}$)*

3.3.19 Azido-Trp-OH conjugated with HP-280 (Table 2 Entry 18)

H-Trp-OH conjugated with HP-280:

Fmoc-Trp-OH (CAS 65737-15-6) was conjugated using general method **ABF 1** (15 µl, 10 mM HP-280, 150 nmol scale) and purified with general purification method **GP1**. The Fmoc deprotection was carried out using general method for Fmoc deprotection and was purified using the methods **GP1** and **GP2**.

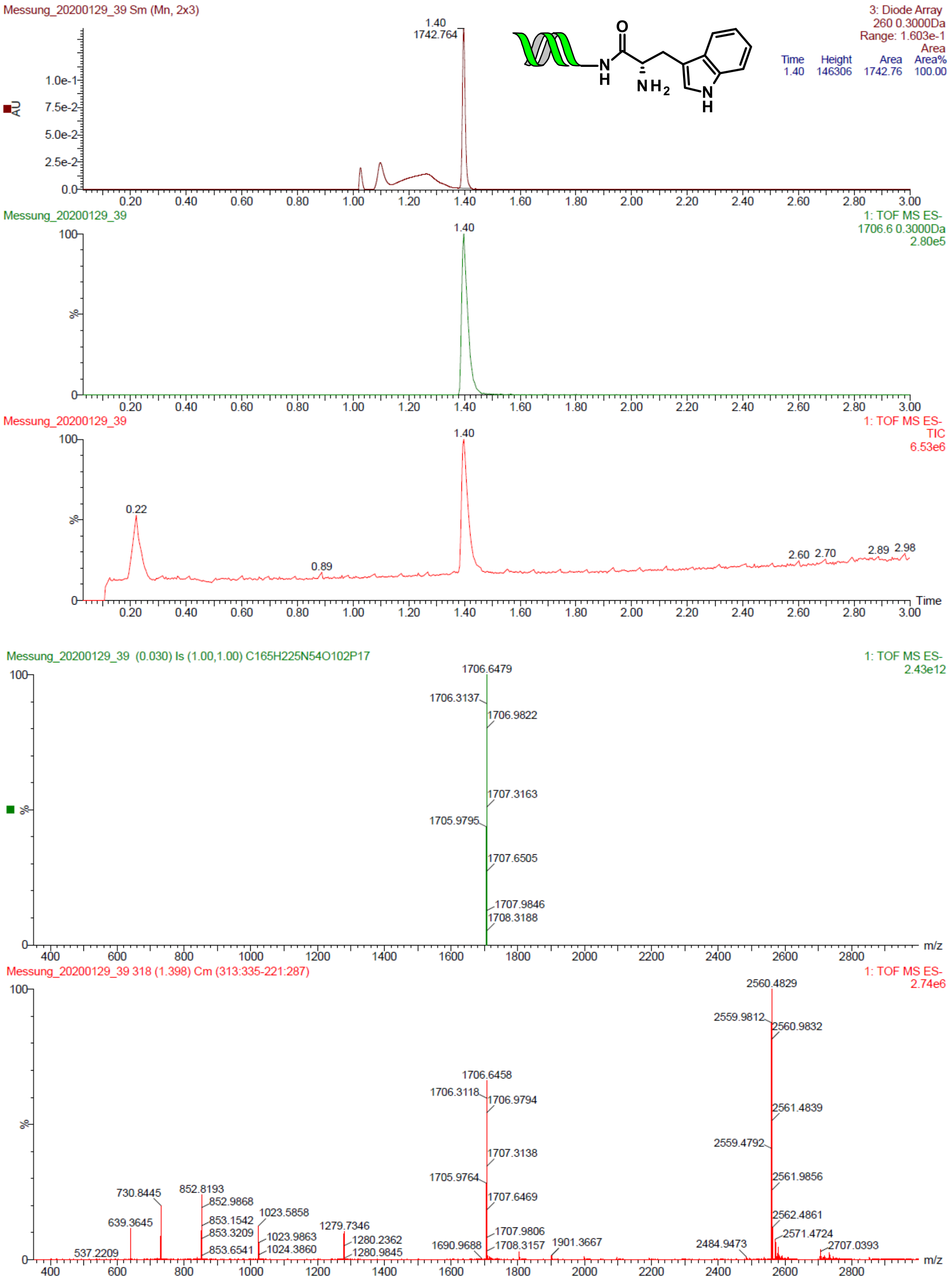

Figure 3.38. LC-MS chromatogram of compound **Table 2 Entry 18 amine** t_R = 1,40 min TOF-MS-ESI$^-$ m/z = 1706,646(100%) [M-3H]$^{3-}$ (calc. 1706,648 for $C_{165}H_{225}N_{54}O_{102}P_{17}$)

Azido-Trp-OH conjugated with HP-280 (Table 2 Entry 18):

The reaction was carried out by using the general method DTR 1 (1 µl, 10 mM of **(Table 2 Entry 18 amine)**, 10nmol scale).

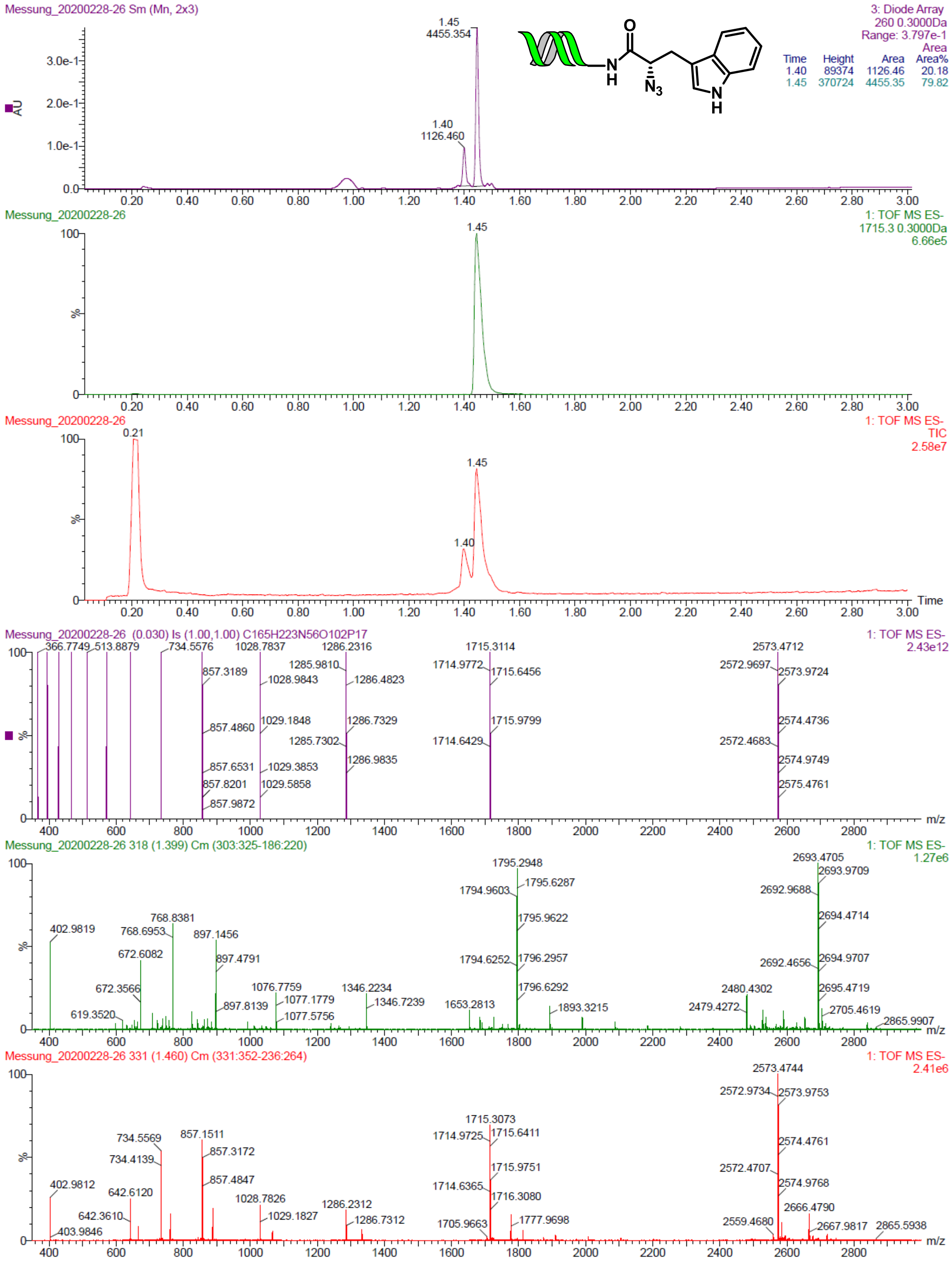

*Figure 3.39: LC-MS chromatogram of compound **Table 2 Entry 18** t_R = 1,45 min TOF-MS-ESI$^-$ m/z = 1715,307(100%) $[M-3H]^{3-}$ (calc. 1715,311 for $C_{165}H_{223}N_{56}O_{102}P_{17}$)*

3.3.20 3-(Azidomethyl)benzoic acid conjugated with HP-280 (Table 2 Entry 19)

3-(Aminomethyl)benzoic acid conjugated with HP-280:

Fmoc-3-(Aminomethyl)benzoic acid (CAS155369-11-2) was conjugated using general method **ABF 2** (15 µl, 10 mM HP-280, 150 nmol scale) and purified with general purification method **GP1**. The Fmoc deprotection was carried out using general method for Fmoc deprotection and was purified using the methods **GP1** and **GP2**.

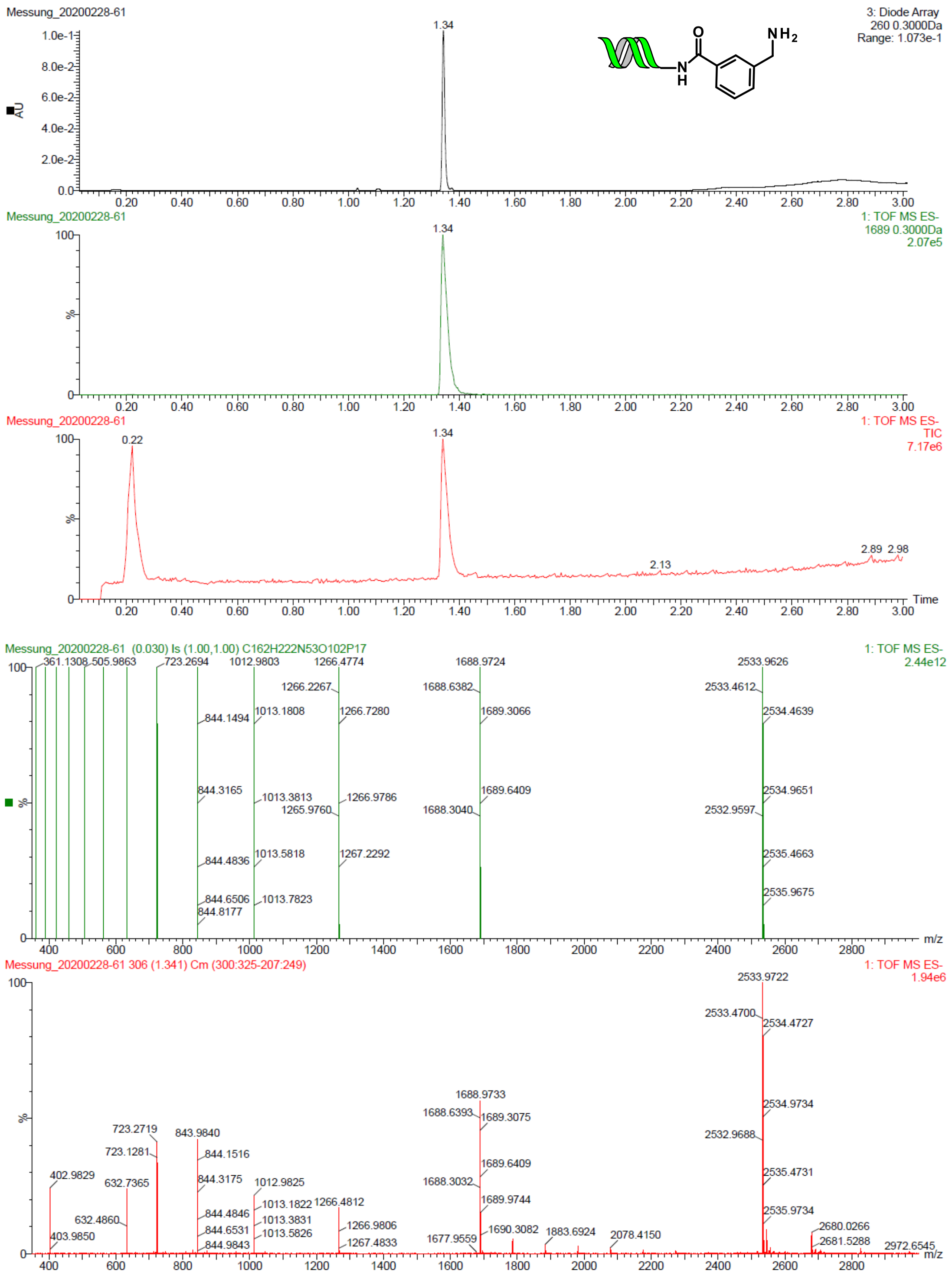

*Figure 3.40. LC-MS chromatogram of compound **Table 2 Entry 19 amine** t_R = 1,46 min TOF-MS-ESI$^-$ m/z = 1688,973(100%) [M-3H]$^{3-}$ (calc. 1688,972 for $C_{162}H_{222}N_{53}O_{102}P_{17}$)*

3-(Azidomethyl)benzoic acid conjugated with HP-280 (Table 2 Entry 19):

The reaction was carried out by using the general method DTR 1 (1 µl, 10 mM of **(Table 2 Entry 19 amine)**, 10nmol scale).

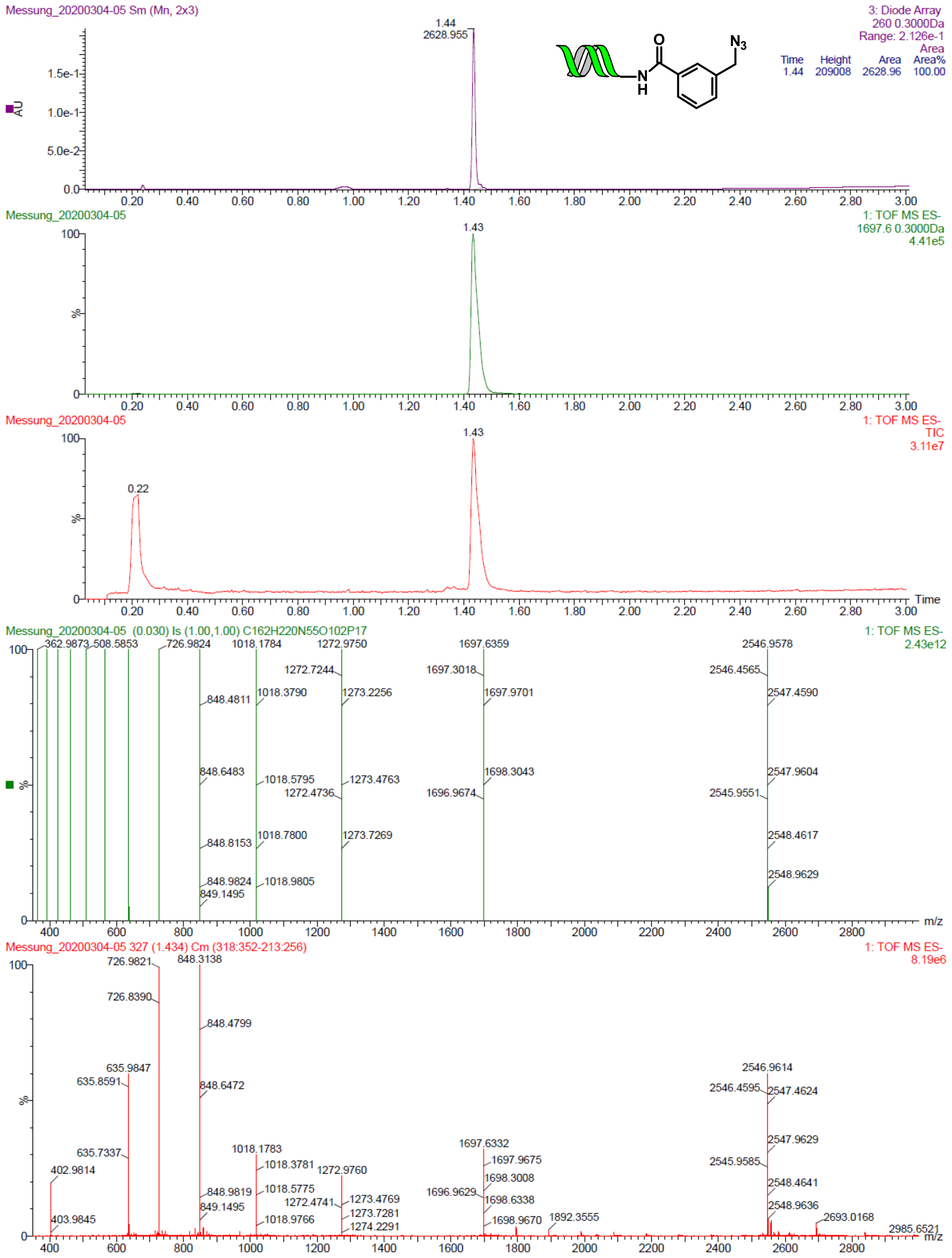

Figure 3.41: LC-MS chromatogram of compound ***Table 2 Entry 19*** *t_R = 1,46 min TOF-MS-ESI$^-$ m/z = 1697,633(100%) [M-3H]$^{3-}$ (calc. 1697,636for $C_{162}H_{220}N_{55}O_{102}P_{17}$)*

3.3.21 Azido-3-Abz-OH conjugated with HP-280 (Table 2 Entry 21)

H-3-Abz-OH conjugated with HP-280:

Fmoc-3-Abz-OH (CAS 185116-42-1) was conjugated using general method **ABF 2** (15 µl, 10 mM HP-280, 150 nmol scale) and purified with general purification method **GP1**. The Fmoc deprotection was carried out using general method for Fmoc deprotection and was purified using the methods **GP1** and **GP2**.

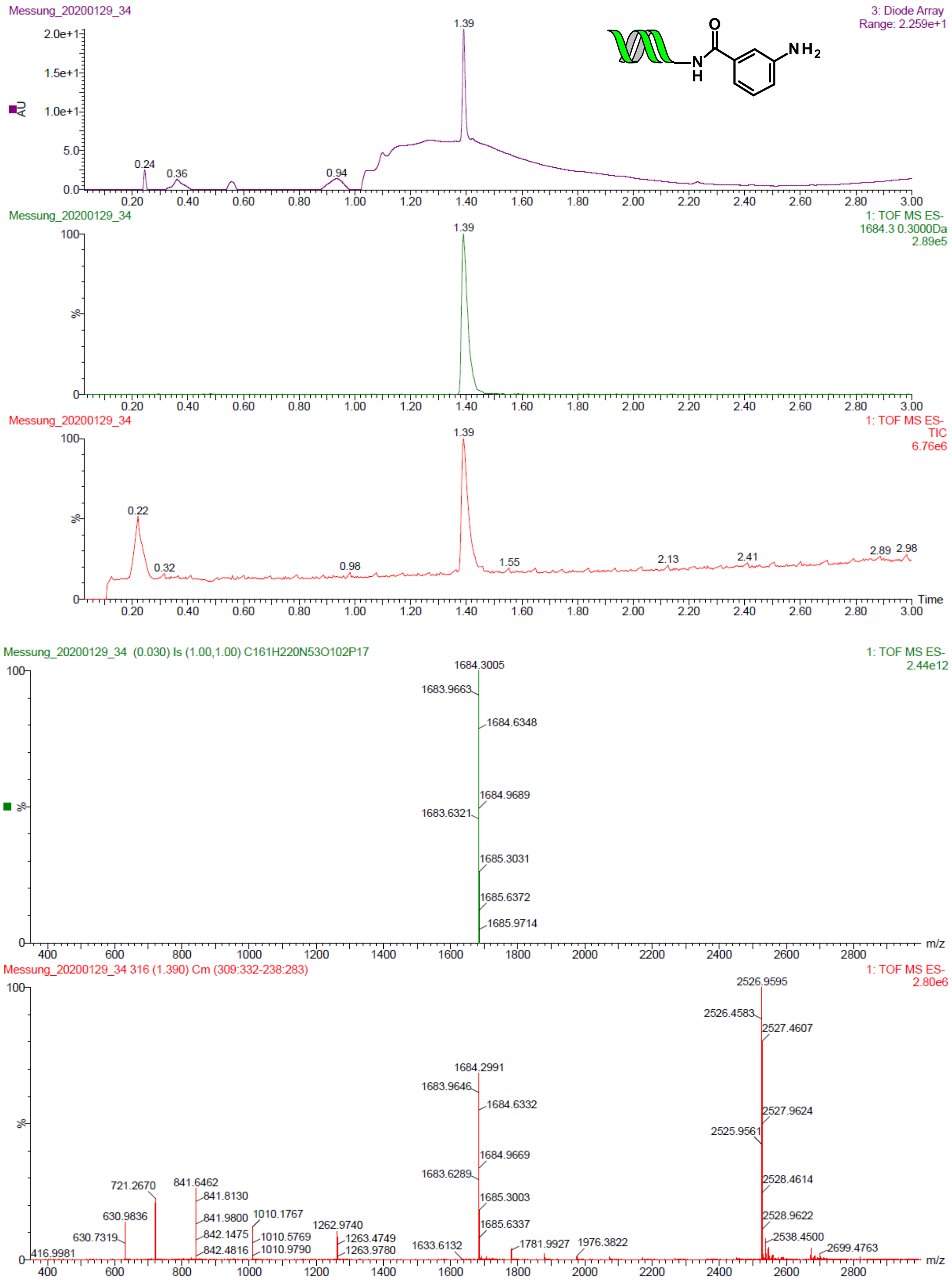

Figure 3.42: LC-MS chromatogram of compound ***Table 2 Entry 21 amine*** *t_R = 1,39 min TOF-MS-ESI$^-$ m/z = 1684,299(100%) [M-3H]$^{3-}$ (calc. 1684,301 for $C_{161}H_{220}N_{53}O_{102}P_{17}$)*

Azido-3-Abz-OH conjugated with HP-280 (Table 2 Entry 21):

The reaction was carried out by using the general method DTR 2(1 µl, 10 mM of **(Table 2 Entry 21 amine)**, 10nmol scale).

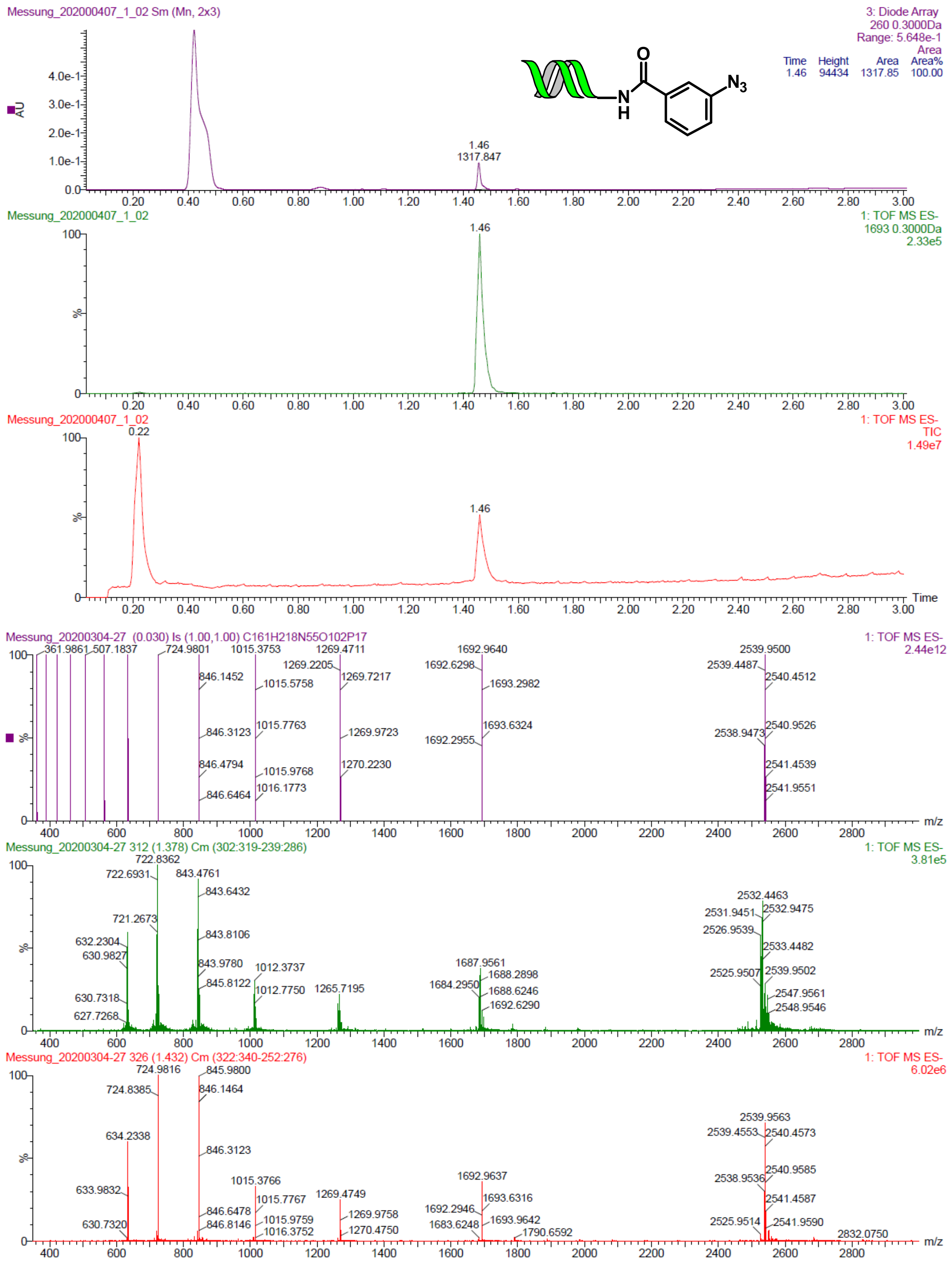

Figure 3.43: LC-MS chromatogram of compound ***Table 2 Entry 21*** *t_R = 1,46 min TOF-MS-ESI$^-$ m/z = 1692,964 (100%) [M-3H]$^{3-}$ (calc. 1692,964 for $C_{161}H_{218}N_{55}O_{102}P_{17}$)*

3.3.22 Azido-2-Abz-OH conjugated with HP-280 (Table 2 Entry 22)

H-2-Abz-OH conjugated with HP-280:

Fmoc-2-Abz-OH (CAS 150256-42-1) was conjugated using general method **ABF 2** (15 µl, 10 mM HP-280, 150 nmol scale) and purified with general purification method **GP1**. The Fmoc deprotection was carried out using general method for Fmoc deprotection and was purified using the methods **GP1** and **GP2**.

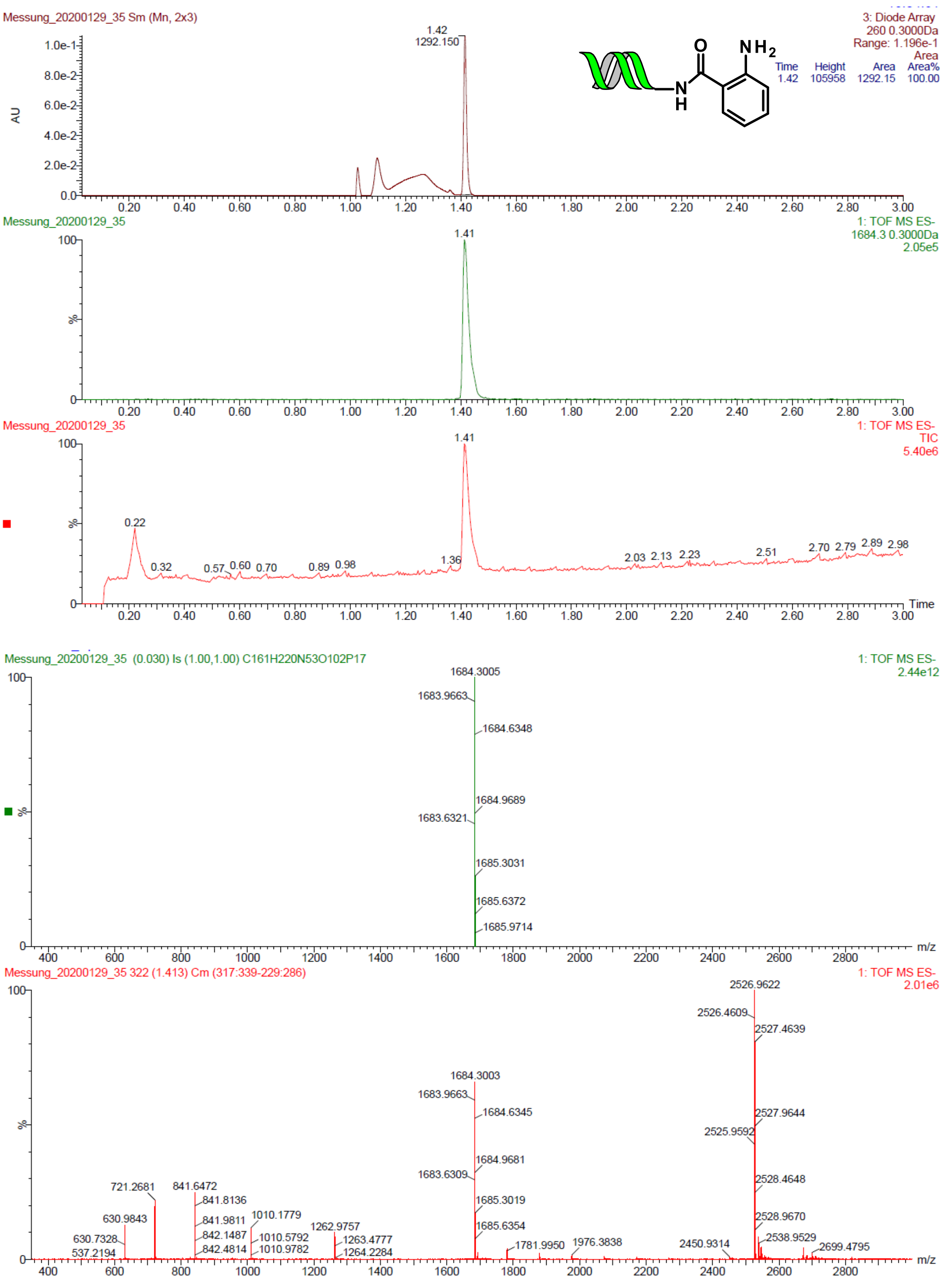

Figure 3.44: LC-MS chromatogram of compound ***Table 2 Entry 22 amine*** *t_R = 1,41 min TOF-MS-ESI$^-$ m/z = 1684,300(100%) [M-3H]$^{3-}$ (calc. 1684,301 for $C_{159}H_{220}N_{53}O_{102}P_{17}$)*

Azido-2-Abz-OH conjugated with HP-280 (Table 2 Entry 22):

The reaction was carried out by using the general method DTR 2(1 µl, 10 mM of **(Table 2 Entry 22 amine)**, 10nmol scale).

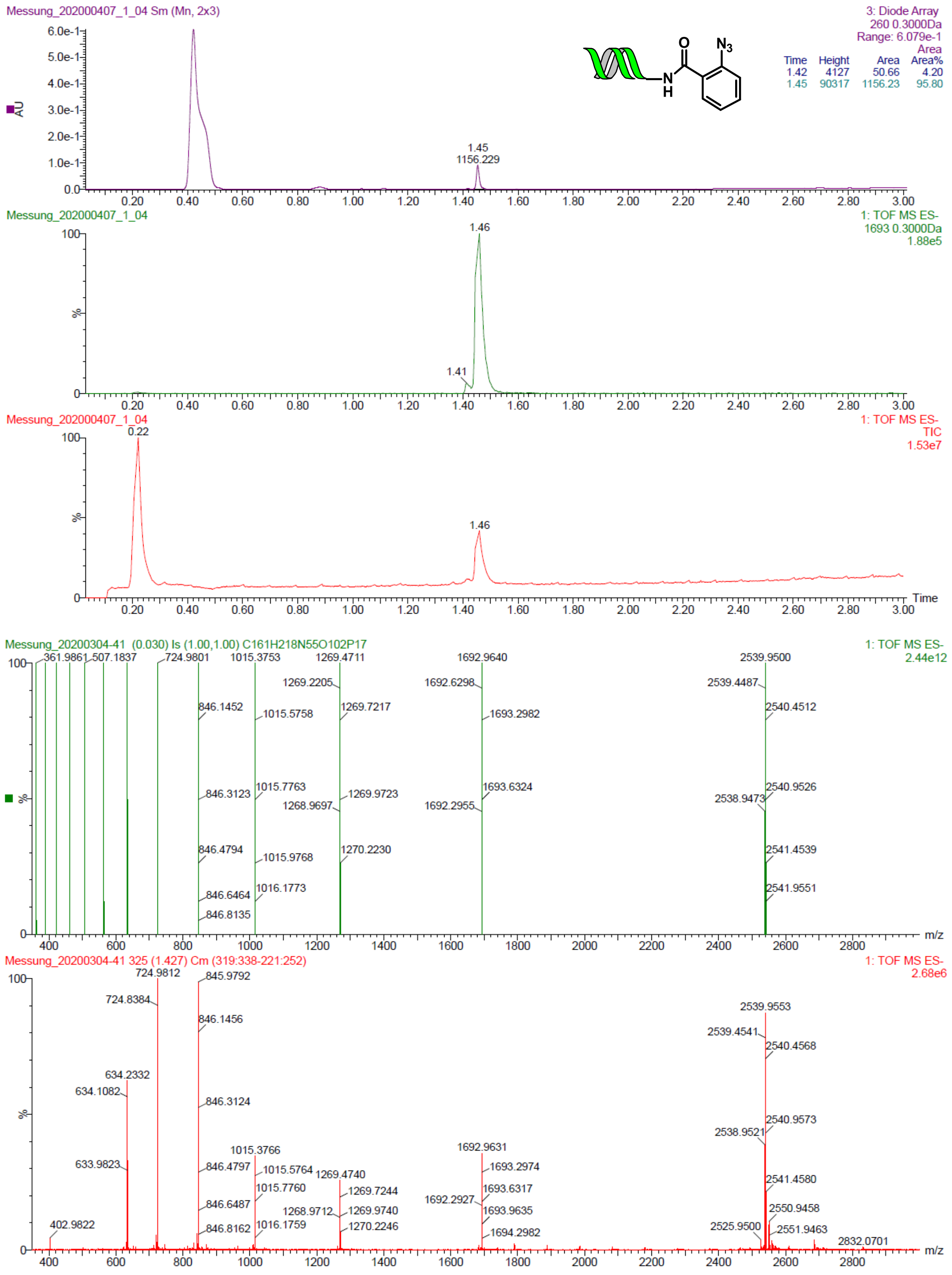

Figure 3.45: LC-MS chromatogram of compound ***Table 2 Entry 22*** *t_R = 1,45 min TOF-MS-ESI$^-$ m/z = 1692,963(100%) [M-3H]$^{3-}$ (calc. 1692,964 for $C_{161}H_{218}N_{55}O_{102}P_{17}$)*

3.3.23 Azido-3-Abz(4-F)-OH conjugated with HP-280 (Table 2 Entry 23)

3-Abz(4-F)-OH conjugated with HP-280:

Fmoc-3-Abz(4F)-OH (CAS 1339407-92-9) was conjugated using general method **ABF 2** (15 µl, 10 mM HP-280, 150 nmol scale) and purified with general purification method **GP1**. The Fmoc deprotection was carried out using general method for Fmoc deprotection and was purified using the methods **GP1** and **GP2**.

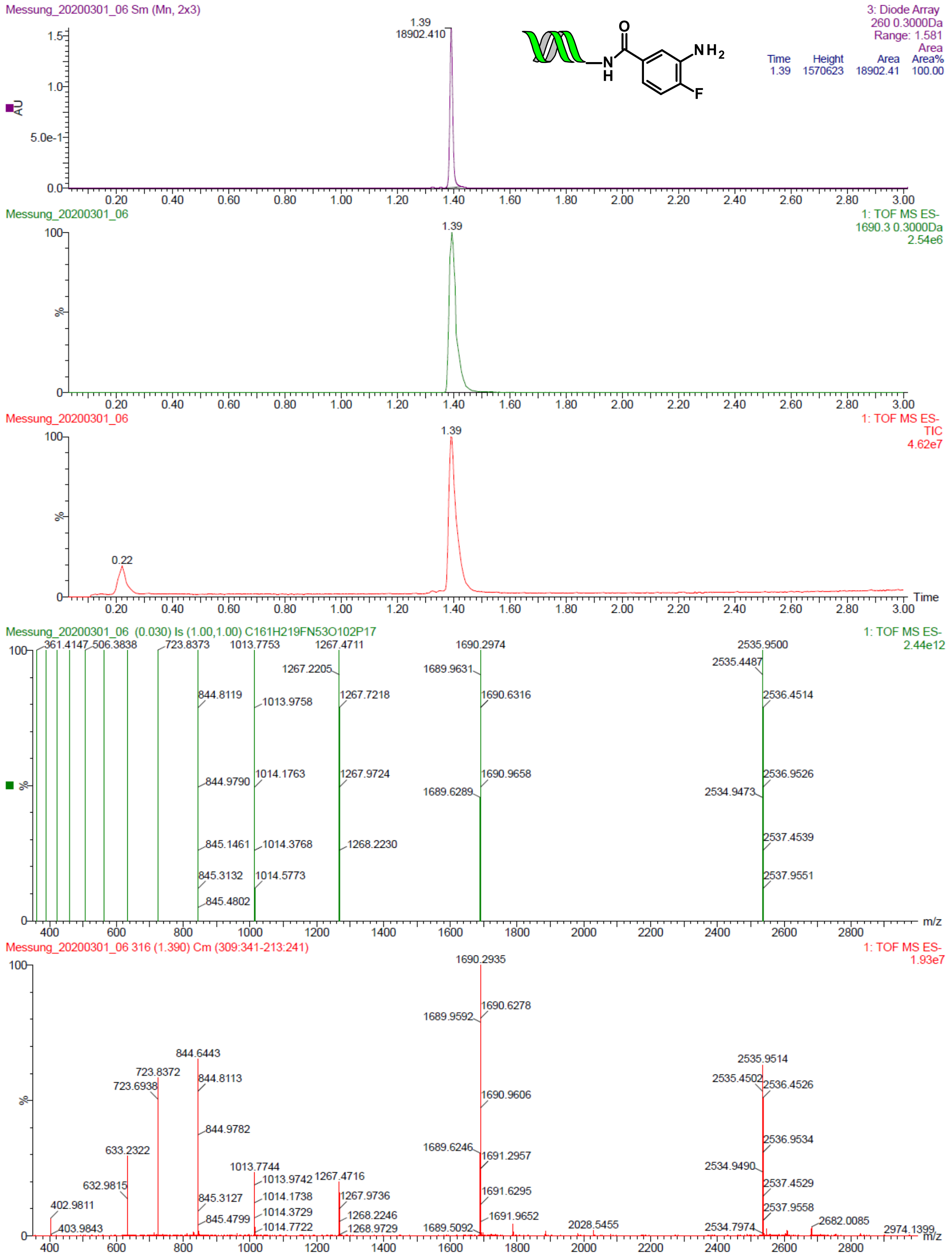

*Figure 3.46: LC-MS chromatogram of compound **Table 2 Entry 23 amine** t_R = 1,39 min TOF-MS-ESI$^-$ m/z = 1690,294(100%) [M-3H]$^{3-}$ (calc. 1690,297 for $C_{161}H_{219}FN_{53}O_{102}P_{17}$)*

Azido-3-Abz(4-F)-OH conjugated with HP-280 (Table 2 Entry 23):

The reaction was carried out by using the general method DTR 2(1 µl, 10 mM of **(Table 2 Entry 23 amine)**, 10nmol scale).

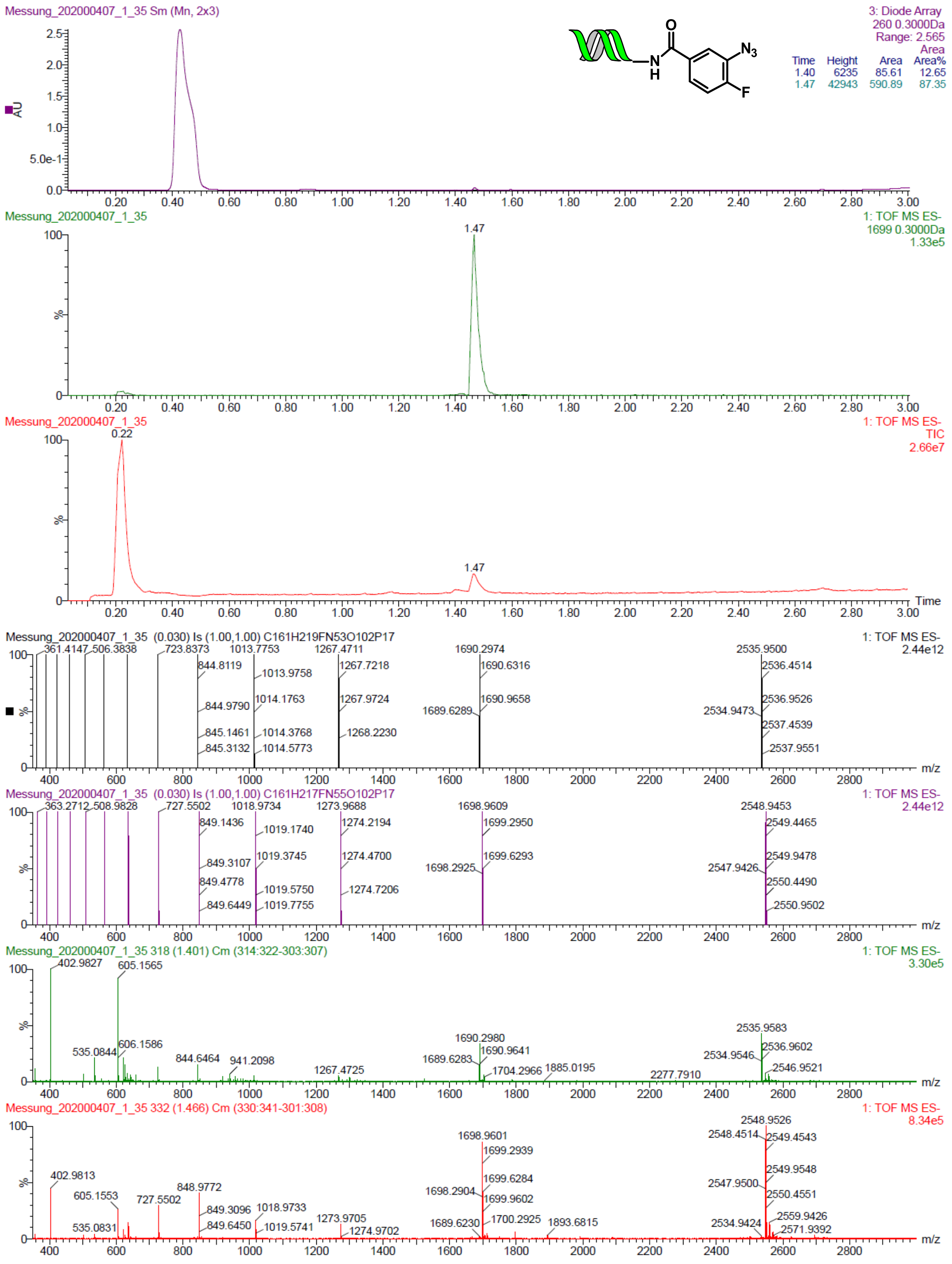

Figure 3.47: LC-MS chromatogram of compound ***Table 2 Entry 23*** *t_R = 1,47 min TOF-MS-ESI$^-$ m/z = 1698,960(100%) [M-3H]$^{3-}$calc. 1698,961 for $C_{161}H_{217}FN_{55}O_{102}P_{17}$)*

3.3.24 Azido-3-Abz(6-Cl)-OH conjugated with HP-280 (Table 2 Entry 24)

H-3-Abz(6-Cl)-OH conjugated with HP-280:

Fmoc-3-Abz(6-Cl)-OH (CAS 186320-11-6) was conjugated using general method **ABF 2** (15 µl, 10 mM HP-280, 150 nmol scale) and purified with general purification method **GP1**. The Fmoc deprotection was carried out using general method for Fmoc deprotection and was purified using the methods **GP1** and **GP2**.

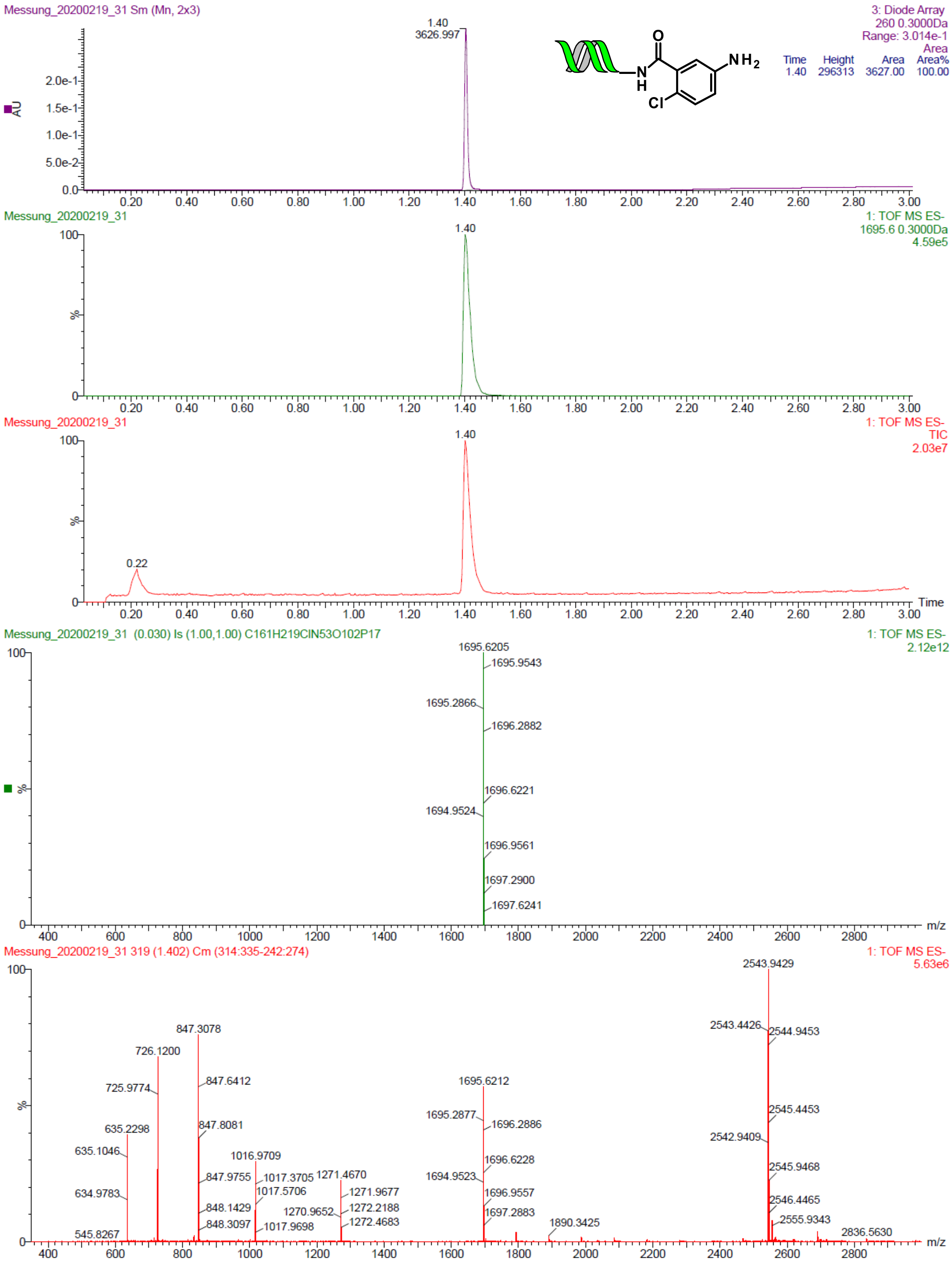

*Figure 3.48: LC-MS chromatogram of compound **Table 2 Entry 24 amine** t_R = 1,43 min TOF-MS-ESI⁻ m/z = 1695,621(100%) [M-3H]$^{3-}$ (calc. 1695,621 for $C_{161}H_{219}ClN_{53}O_{102}P_{17}$)*

Azido-3-Abz(6-Cl)-OH conjugated with HP-280 (Table 2 Entry 24)

The reaction was carried out by using the general method DTR 2(1 µl, 10 mM of **(Table 2 Entry 24 amine)**, 10nmol scale).

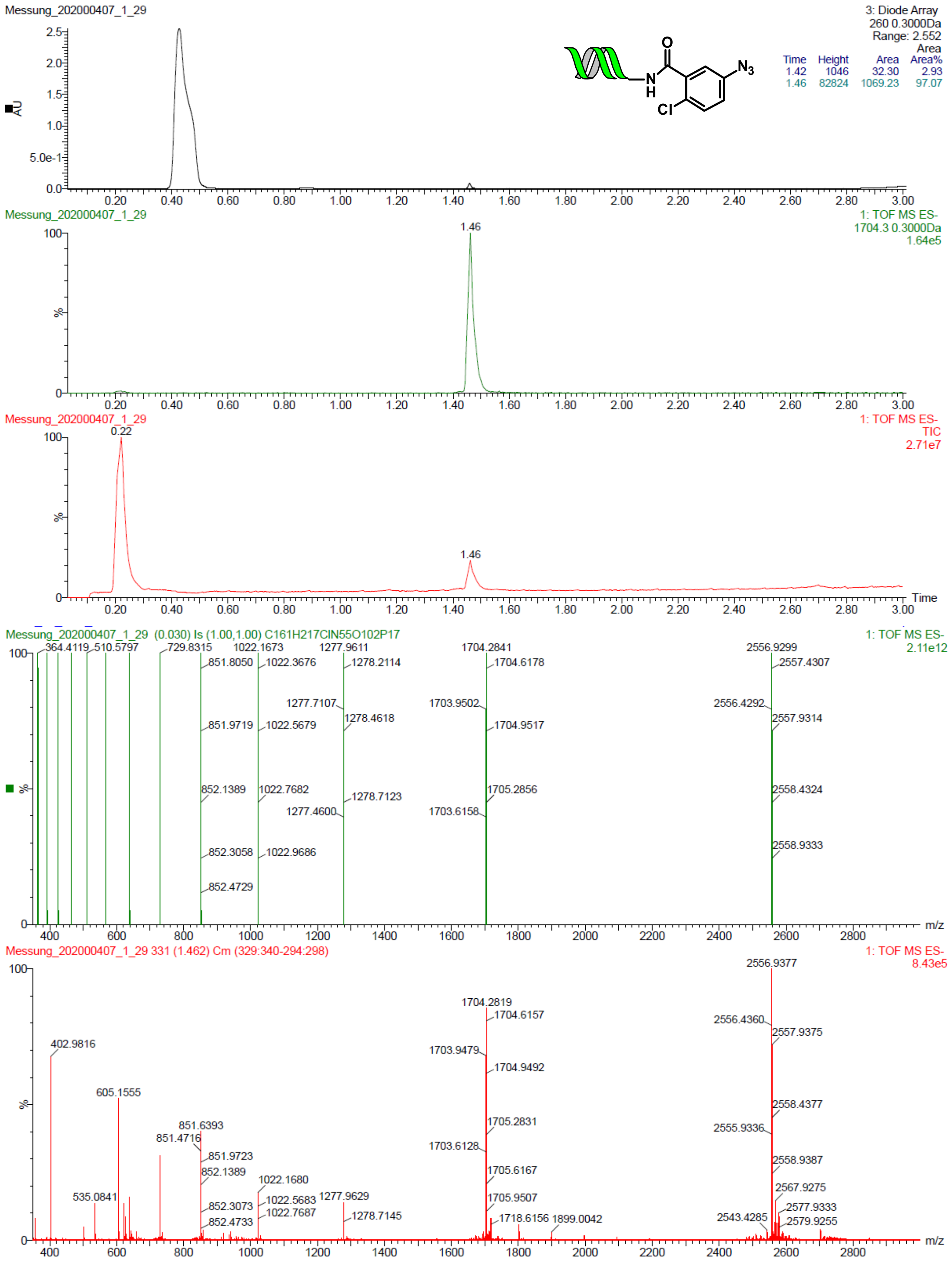

Figure 3.49: LC-MS chromatogram of compound ***Table 2 Entry 24*** *t_R = 1,46 min TOF-MS-ESI$^-$ m/z =1704,282(100%) [M-3H]$^{3-}$ (calc. 1704,284for $C_{161}H_{217}ClN_{55}O_{102}P_{17}$)*

3.3.25 Azido-2-Abz(6-Br)-OH conjugated with HP-280 (Table 2 Entry 25)

H-2-Abz(6-Br)-OH conjugated with HP-280:

Fmoc-2-Abz(6-Br)-OH (CAS 20776-48-1 free amine) was conjugated using general method **ABF 2** (15 µl, 10 mM HP-280, 150 nmol scale) and purified with general purification method **GP1**. The Fmoc deprotection was carried out using general method for Fmoc deprotection and was purified using the methods **GP1** and **GP2.**

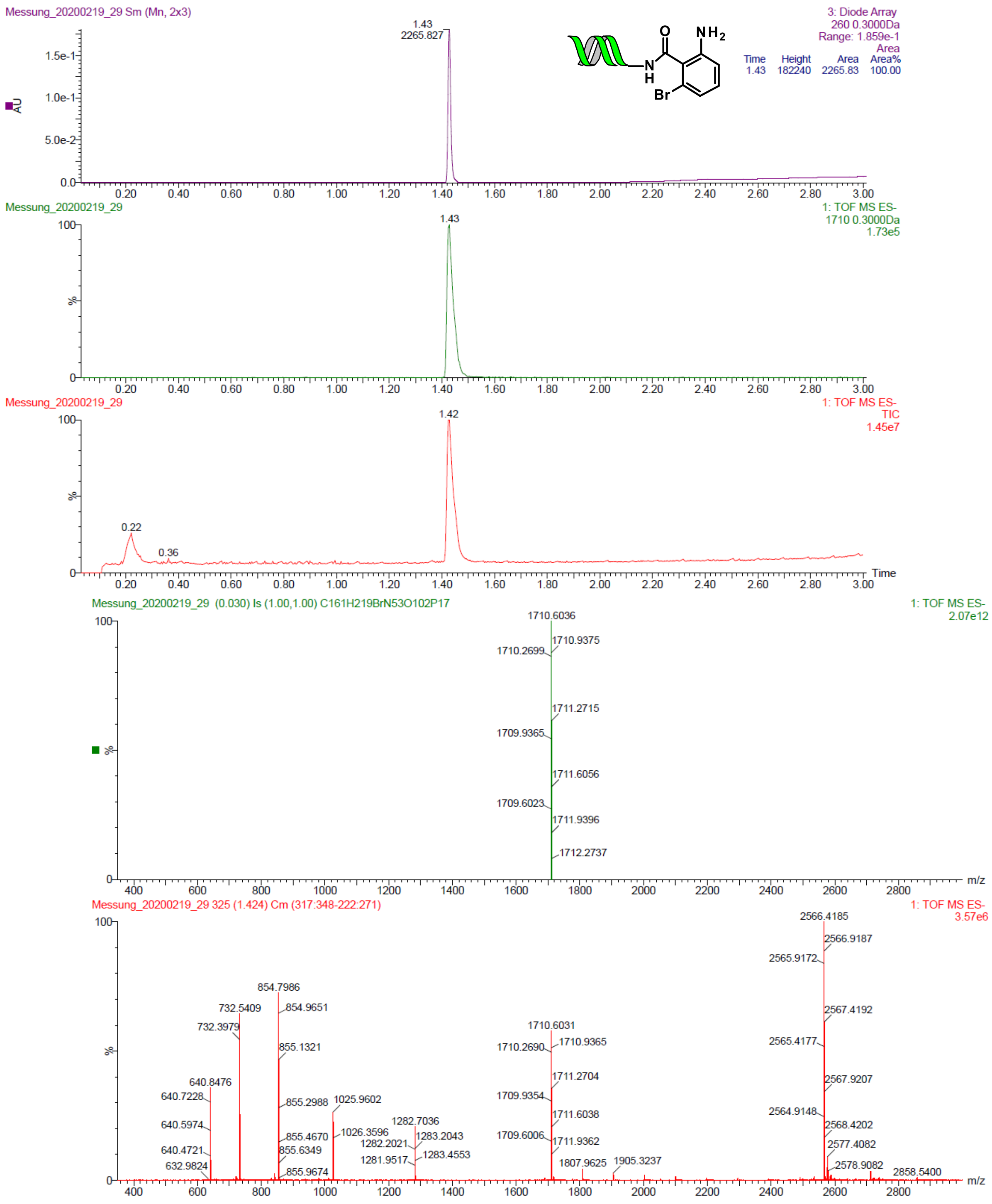

Figure 3.50: LC-MS chromatogram of compound ***Table 2 Entry 25 amine*** *t_R = 1,43 min TOF-MS-ESI$^-$ m/z = 1710,603(100%) $[M-3H]^{3-}$ (calc. 1710,604 for $C_{161}H_{219}BrN_{53}O_{102}P_{17}$)*

Azido-2-Abz(6-Br)-OH conjugated with HP-280 (Table 2 Entry 25):

The reaction was carried out by using the general method DTR 2(1 µl, 10 mM of **(Table 2 Entry 25 amine)**, 10nmol scale).

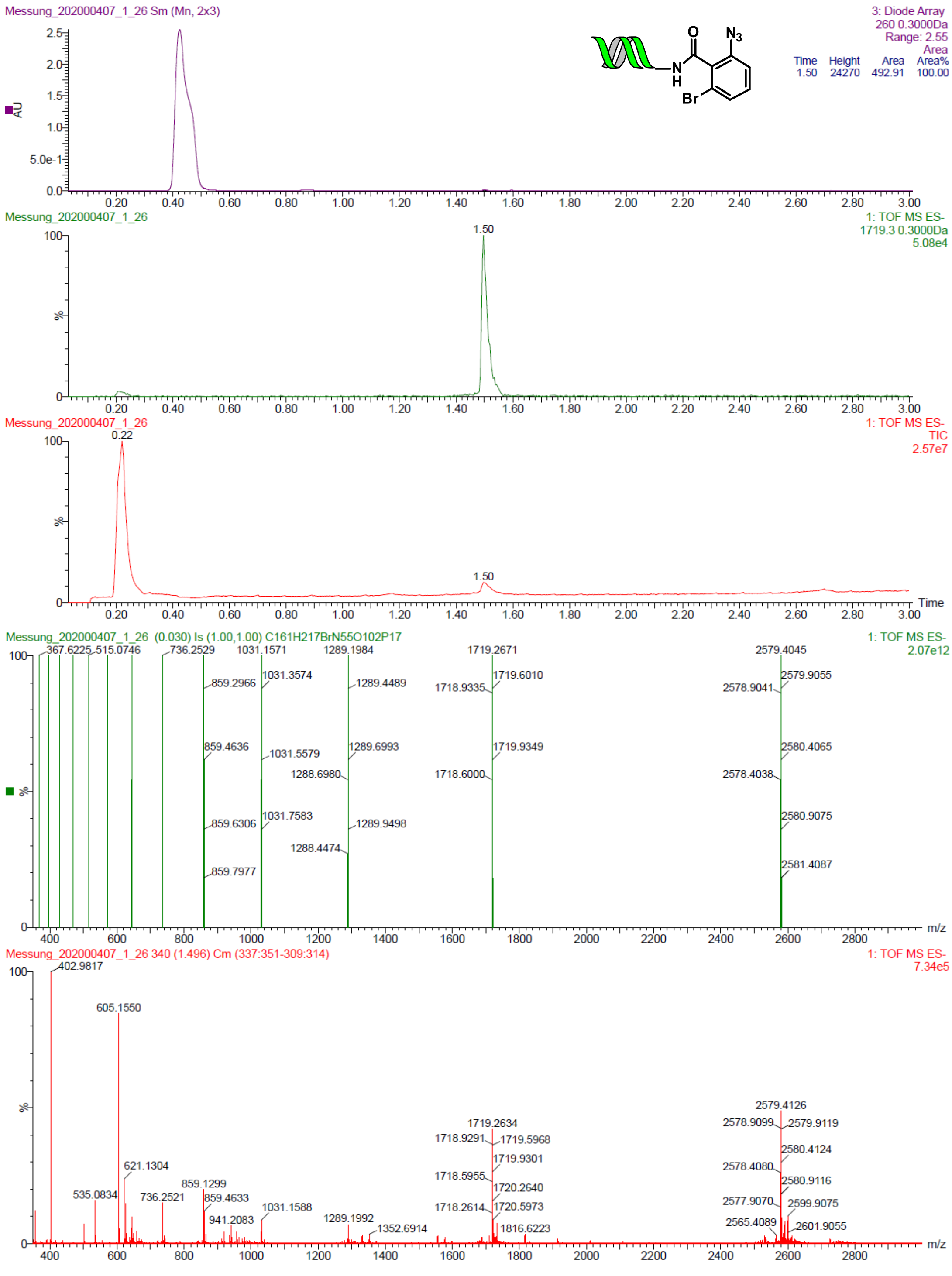

Figure 3.51: LC-MS chromatogram of compound ***Table 2 Entry 25*** *t_R = 1,50 min TOF-MS-ESI⁻ m/z = 1719,363(100%) [M-3H]$^{3-}$ (calc. 1719,367 for $C_{161}H_{217}BrN_{55}O_{102}P_{17}$)*

3.3.26 H-2-Abz(4-Br)-OH conjugated with HP-280 (Table 2 Entry 26)

H-2-Abz(4-Br)-OH conjugated with HP-280

Fmoc-2-Abz(4-Br)-OH (CAS 1340472-73-2) was conjugated using general method **ABF 2** (15 µl, 10 mM HP-280, 150 nmol scale) and purified with general purification method **GP1**. The Fmoc deprotection was carried out using general method for Fmoc deprotection and was purified using the methods **GP1** and **GP2.**

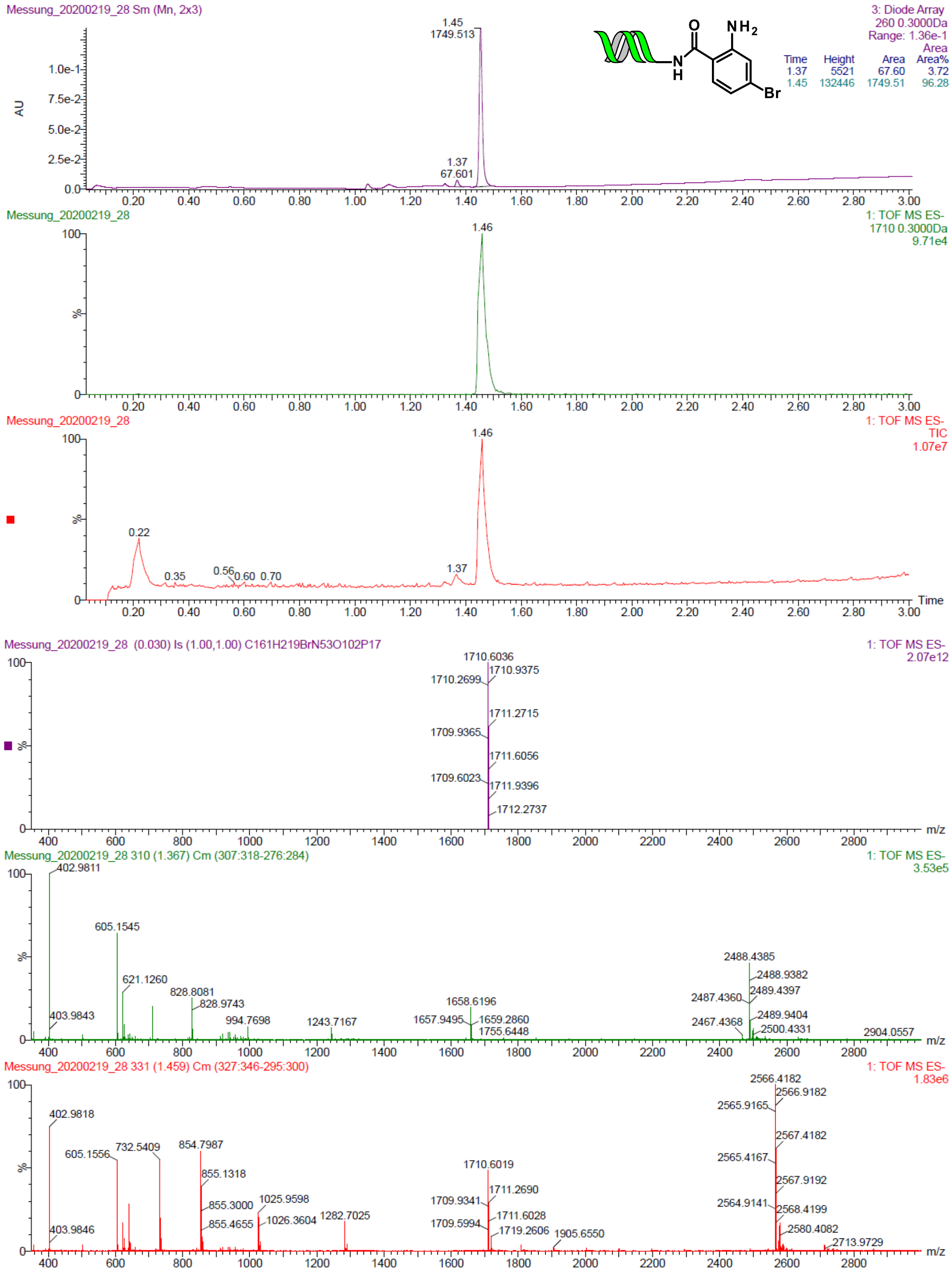

*Figure 3.52: LC-MS chromatogram of compound **Table 2 Entry 26 amine** t_R = 1.46 min TOF-MS-ESI$^-$ m/z = 1710.602(100%) [M-3H]$^{3-}$ (calc. 1710.604 for $C_{161}H_{219}BrN_{53}O_{102}P_{17}$)*

Azido-2-Abz(4-Br)-OH conjugated with HP-280 (Table 2 Entry 26)

The reaction was carried out by using the general method DTR 2(1 µl, 10 mM of **(Table 2 Entry 25 amine)**, 10nmol scale).

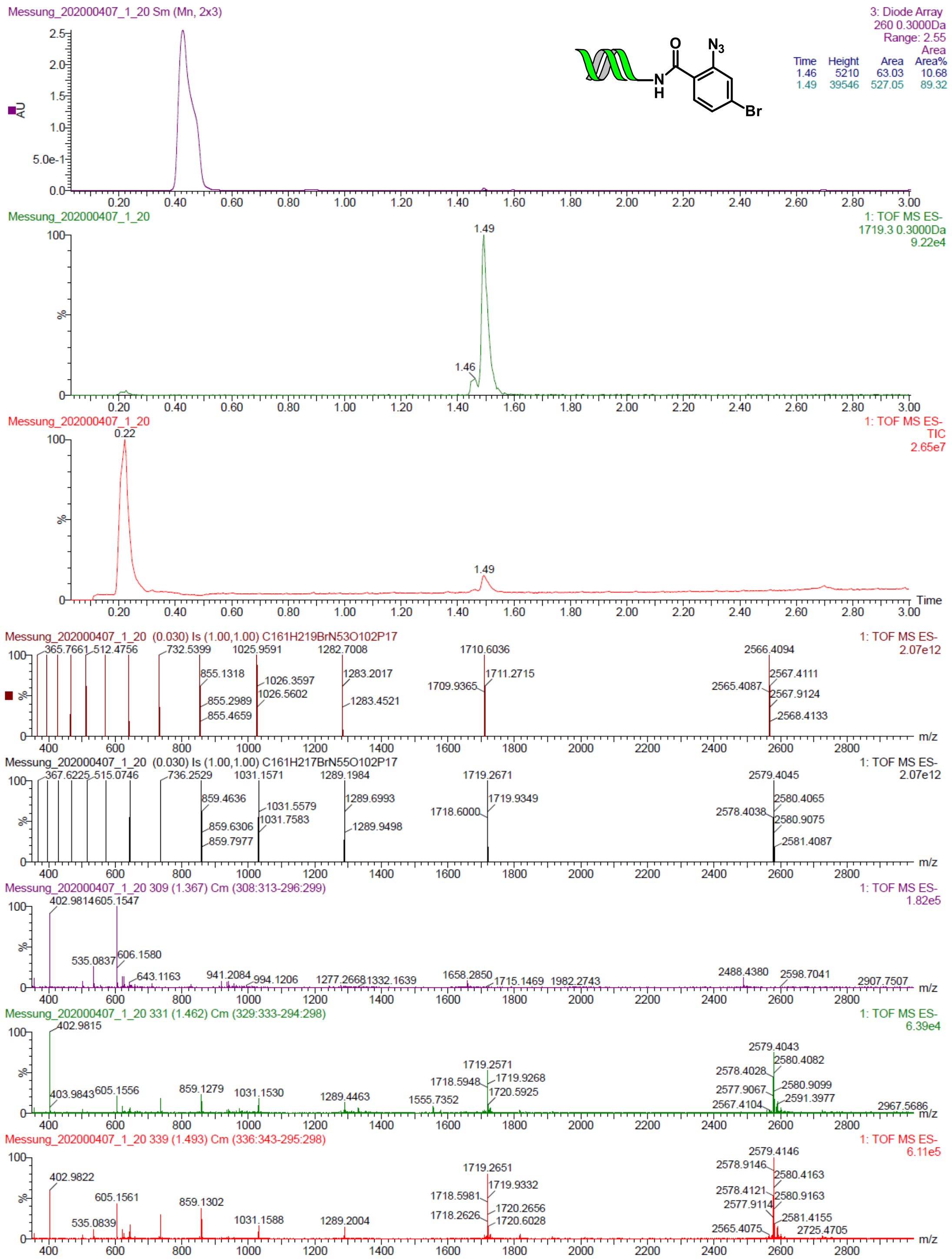

*Figure 3.53: LC-MS chromatogram of compound **Table 2 Entry 25** t_R = 1.49 min TOF-MS-ESI$^-$ m/z = 1719.265(100%) [M-3H]$^{3-}$ (calc. 1719.267 for $C_{161}H_{217}BrN_{55}O_{102}P_{17}$)*

3.3.27 H-3-Abz(3-Br)-OH conjugated with HP-280 (Table 2 Entry 27)

H-3-Abz(3-Br)-OH conjugated with HP-280

Fmoc-3-Abz(3-Br)-OH (CAS 1696643-67-0) was conjugated using general method **ABF 2** (15 µl, 10 mM HP-280, 150 nmol scale) and purified with general purification method **GP1**. The Fmoc deprotection was carried out using general method for Fmoc deprotection and was purified using the methods **GP1** and **GP2.**

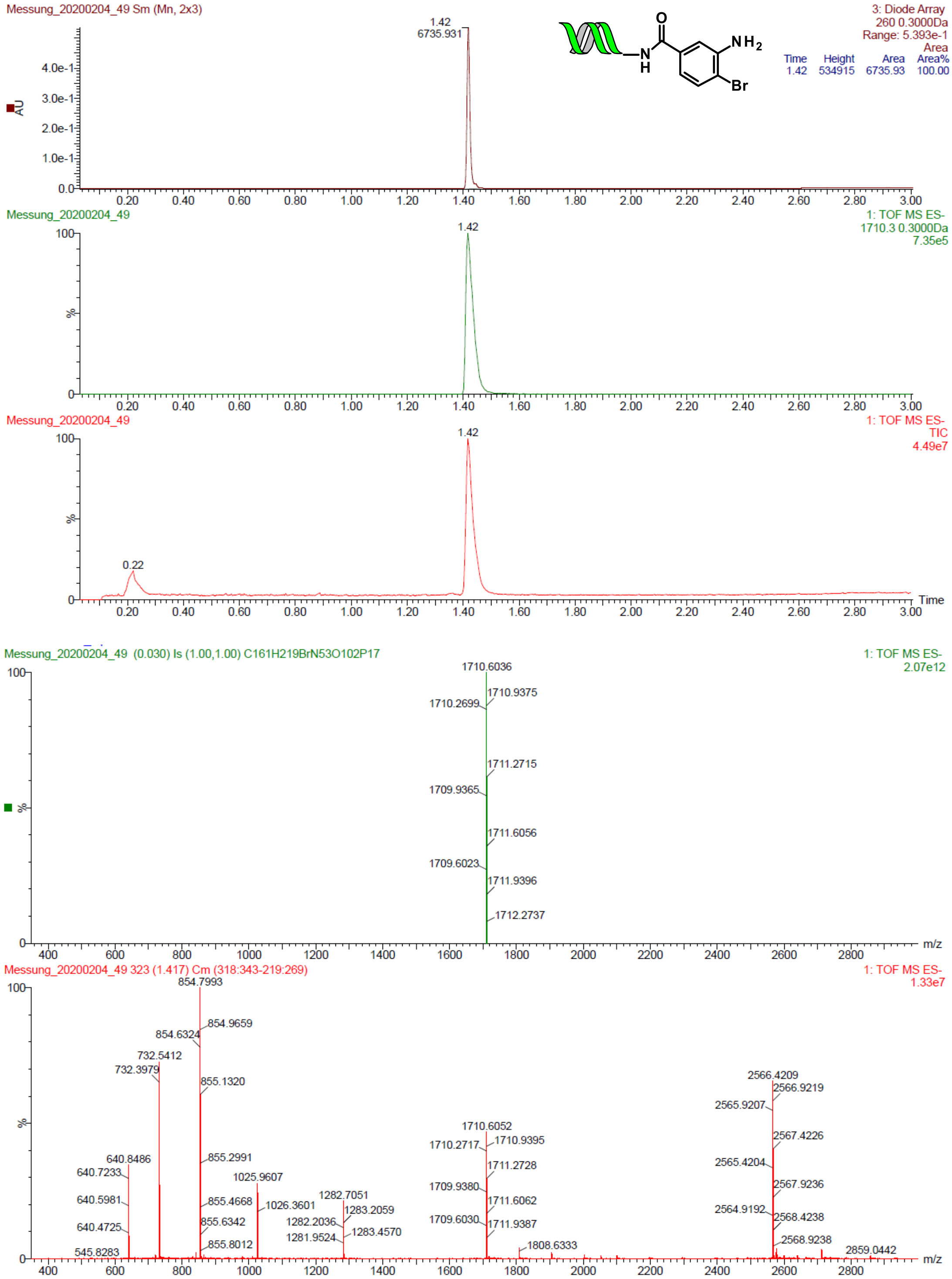

Figure 3.54: LC-MS chromatogram of compound ***Table 2 Entry 27 amine*** *t_R = 1.42 min TOF-MS-ESI$^-$ m/z = 1710.605(100%) $[M-3H]^{3-}$ (calc. 1710.604 for $C_{161}H_{219}BrN_{53}O_{102}P_{17}$)*

Azido-3-Abz(3-Br)-OH conjugated with HP-280 (Table 2 Entry 27)

The reaction was carried out by using the general method DTR 2(1 µl, 10 mM of **(Table 2 Entry 25 amine)**, 10nmol scale).

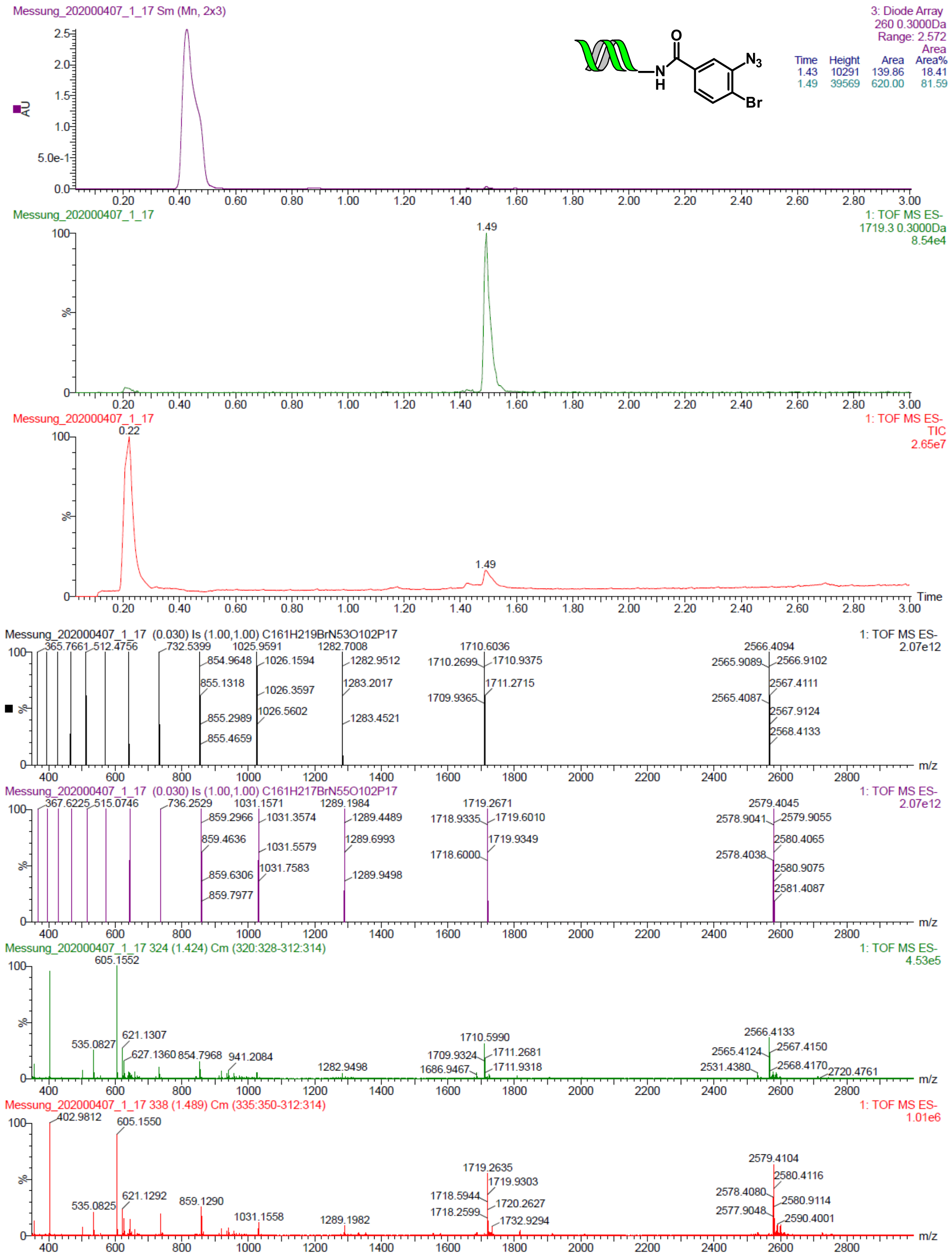

*Figure 3.55: LC-MS chromatogram of compound **Table 2 Entry 27** t_R = 1.49 min TOF-MS-ESI$^-$ m/z = 1719.264(100%) [M-3H]$^{3-}$ (calc. 1719.267 for $C_{161}H_{217}BrN_{55}O_{102}P_{17}$)*

3.3.28 Azido-3-Abz(2-Br)-OH conjugated with HP-280 (Table 2 Entry 28)

H-3-Abz(2-Br)-OH conjugated

Fmoc-3-Abz(2-Br)-OH (CAS 16889-61-4 free amine) was conjugated using general method **ABF 2** (15 µl, 10 mM HP-280, 150 nmol scale) and purified with general purification method **GP1**. The Fmoc deprotection was carried out using general method for Fmoc deprotection and was purified using the methods **GP1** and **GP2.**

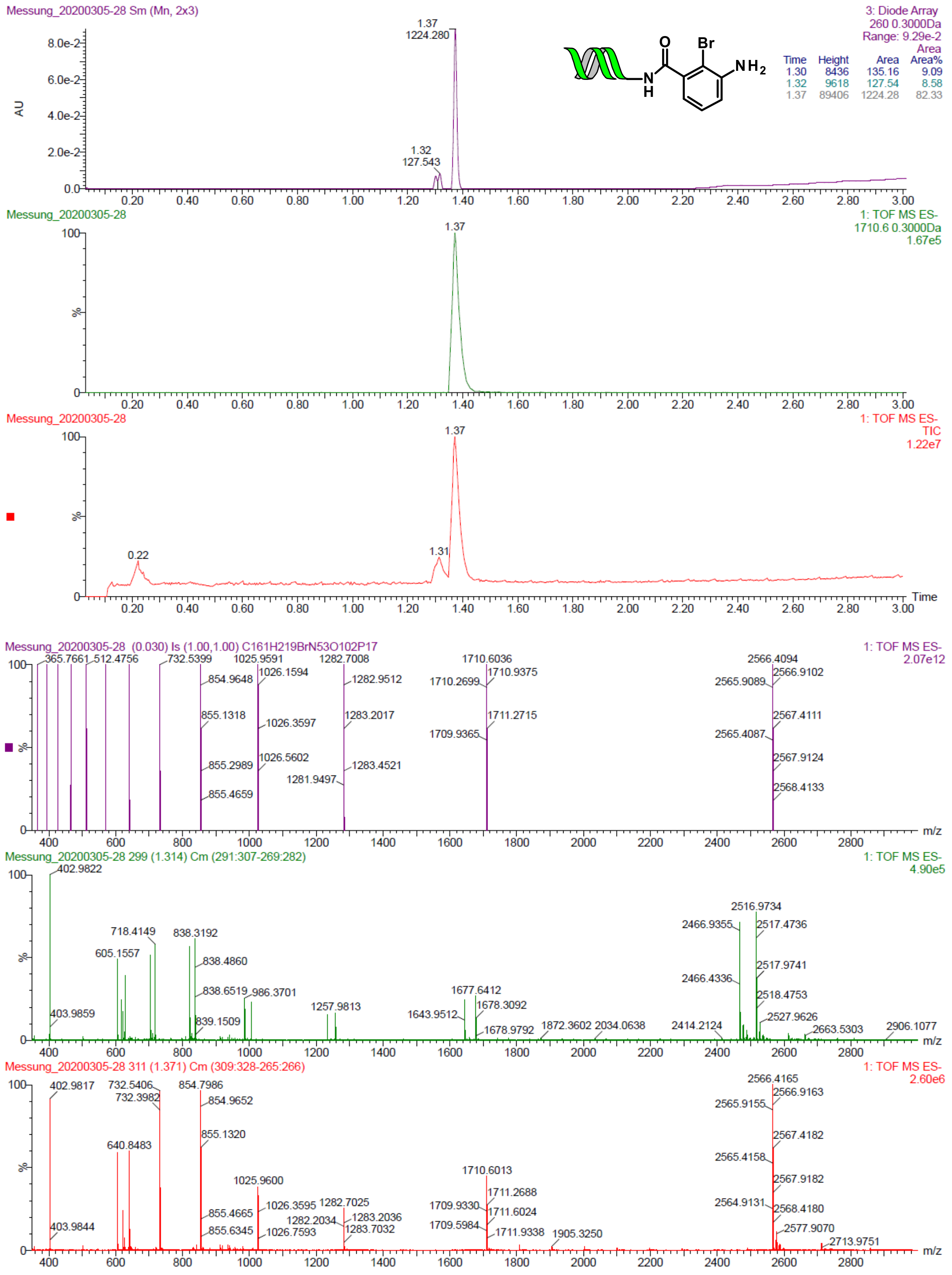

Figure 3.56: LC-MS chromatogram of compound **Table 2 Entry 28 amine** t_R = 1.37 min TOF-MS-ESI$^-$ m/z = 1710.601(100%) $[M-3H]^{3-}$ (calc. 1710.604 for $C_{161}H_{219}BrN_{53}O_{102}P_{17}$)

Azido-3-Abz(2-Br)-OH conjugated with HP-280 (Table 2 Entry 28)

The reaction was carried out by using the general method DTR 2(1 µl, 10 mM of **(Table 2 Entry 25 amine)**, 10nmol scale).

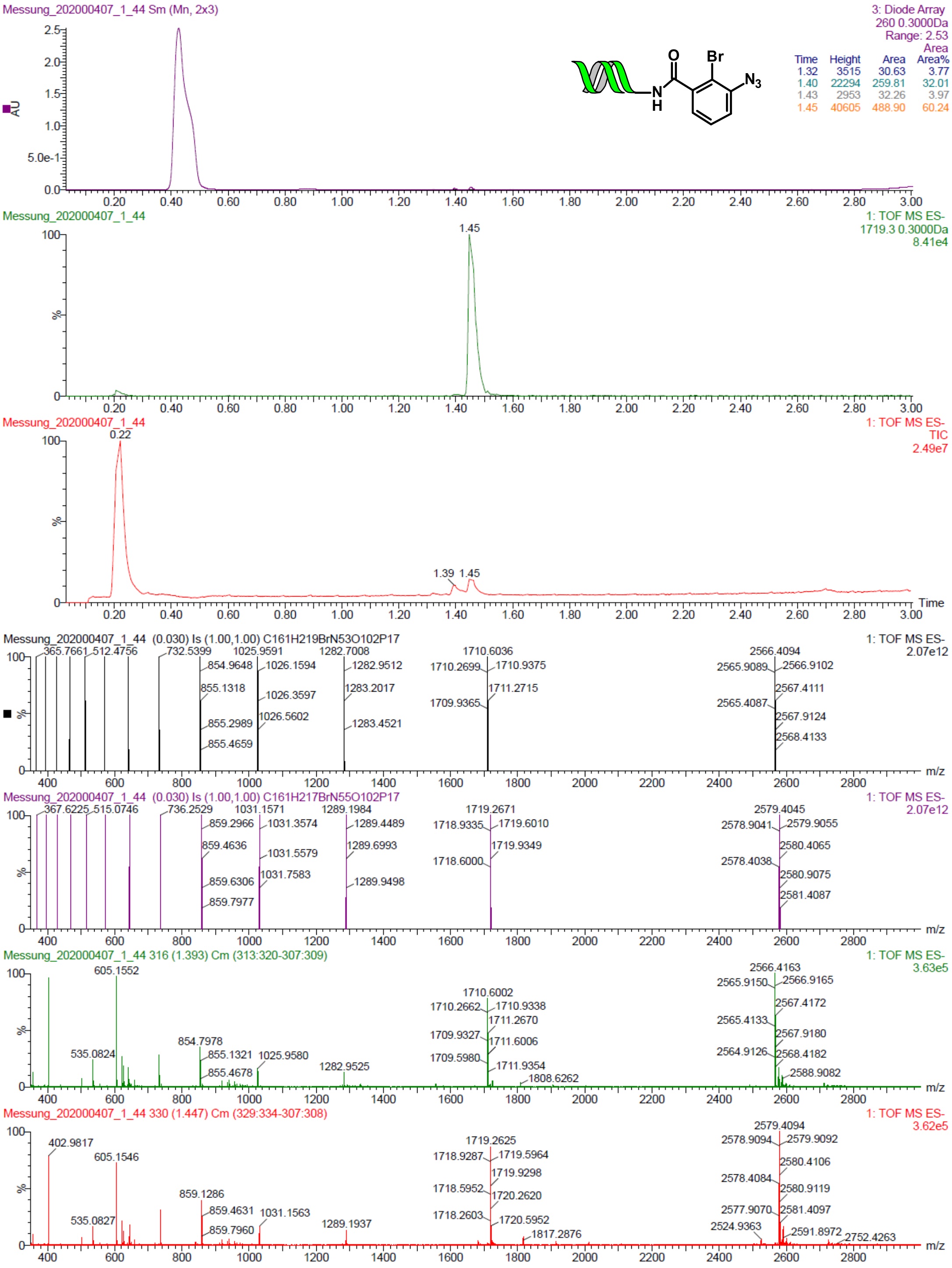

*Figure 3.57: LC-MS chromatogram of compound **Table 2 Entry 28** t_R = 1.45 min TOF-MS-ESI⁻ m/z = 1719.263(100%) $[M-3H]^{3-}$ (calc. 1719.267 for $C_{161}H_{217}BrN_{55}O_{102}P_{17}$)*

3.3.29 Azido-4-Abz(2-Br)-OH conjugated with HP-280 (Table 2 Entry 29)

H-4-Abz(2-Br)-OH conjugated with HP-280

Fmoc-4-Abz(2-Br)-OH (CAS 2486-52-4 free amine) was conjugated using general method **ABF 2** (15 µl, 10 mM HP-280, 150 nmol scale) and purified with general purification method **GP1**. The Fmoc deprotection was carried out using general method for Fmoc deprotection and was purified using the methods **GP1** and **GP2.**

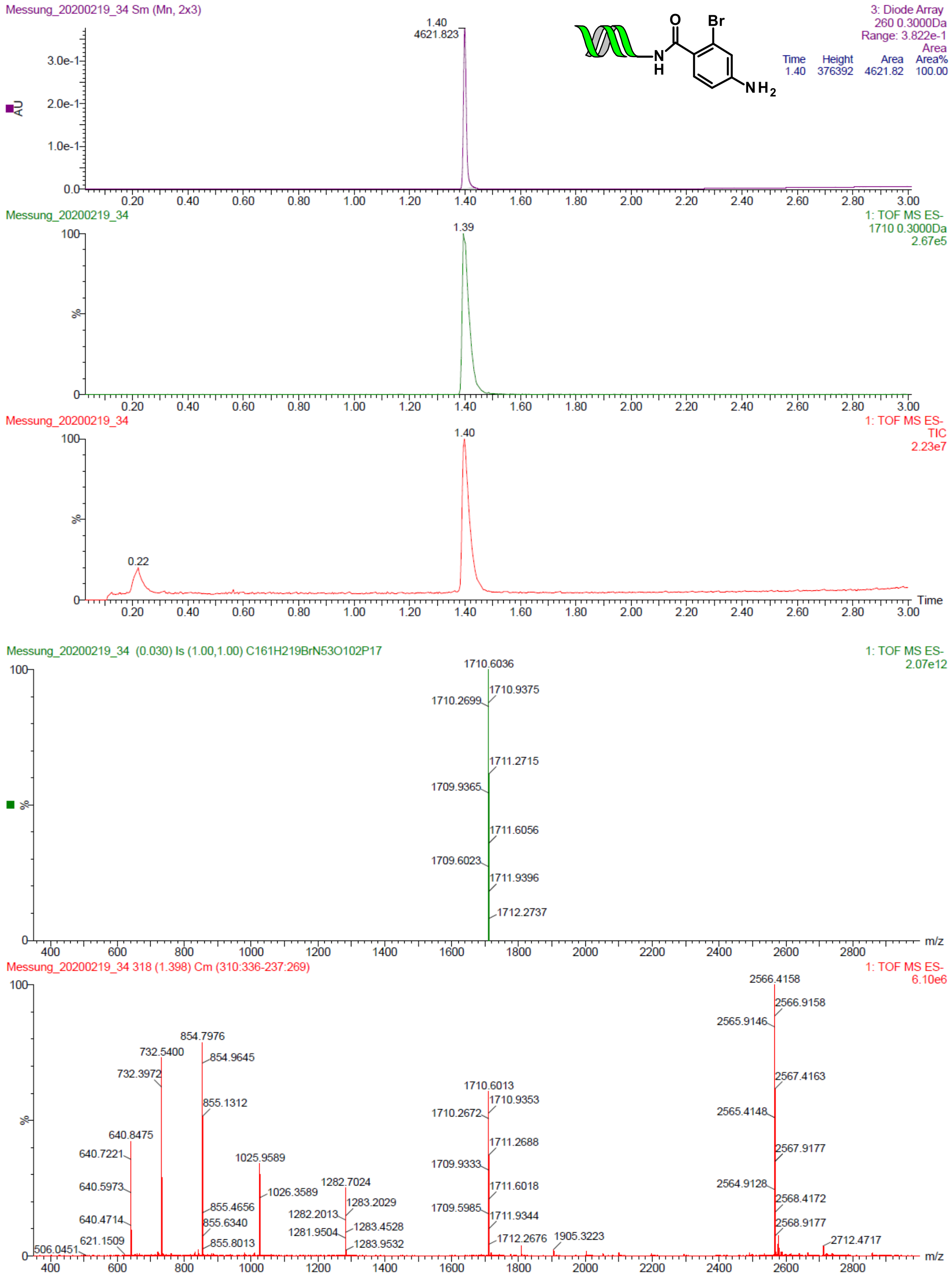

*Figure 3.58: LC-MS chromatogram of compound **Table 2 Entry 29 amine** t_R = 1.40 min TOF-MS-ESI$^-$ m/z = 1710.6015(100%) [M-3H]$^{3-}$ (calc. 1710.604 for $C_{161}H_{219}BrN_{53}O_{102}P_{17}$)*

Azido-4-Abz(2-Br)-OH conjugated with HP-280 (Table 2 Entry 29)

The reaction was carried out by using the general method DTR 2(1 µl, 10 mM of **(Table 2 Entry 25 amine)**, 10nmol scale).

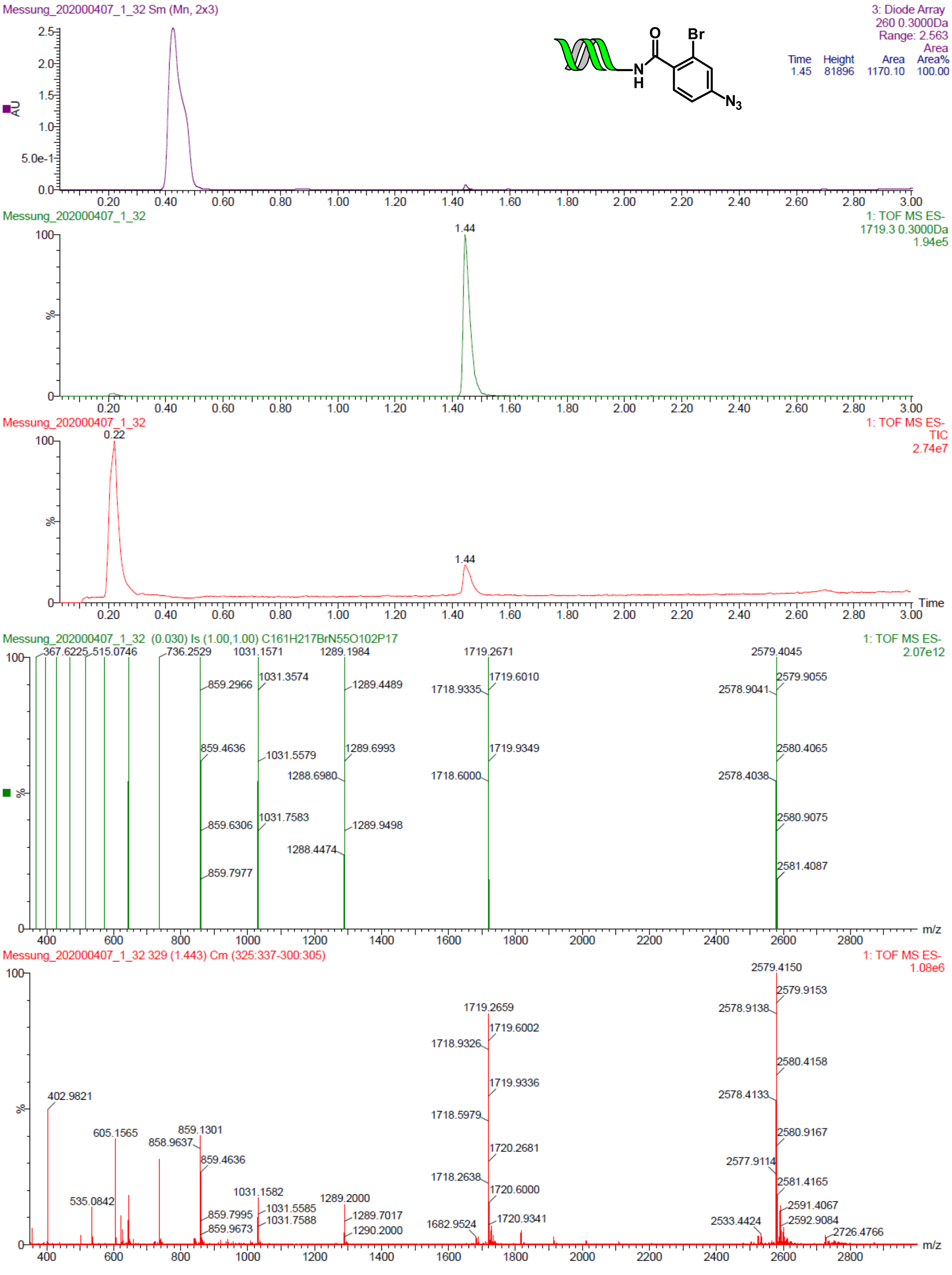

*Figure 3.59: LC-MS chromatogram of compound **Table 2 Entry 29** t_R = 1.45 min TOF-MS-ESI$^-$ m/z = 1719.266(100%) $[M-3H]^{3-}$ (calc. 1719.267 for $C_{161}H_{217}BrN_{55}O_{102}P_{17}$)*

3.3.30 Azido-4-Abz(3-Br)-OH conjugated with HP-280 (Table 2 Entry 30)

H-4-Abz(3-Br)-OH conjugated with HP-280

Fmoc-4-Abz(3-Br)-OH (CAS 1339688-25-3) was conjugated using general method **ABF 2** (15 µl, 10 mM HP-280, 150 nmol scale) and purified with general purification method **GP1**. The Fmoc deprotection was carried out using general method for Fmoc deprotection and was purified using the methods **GP1** and **GP2.**

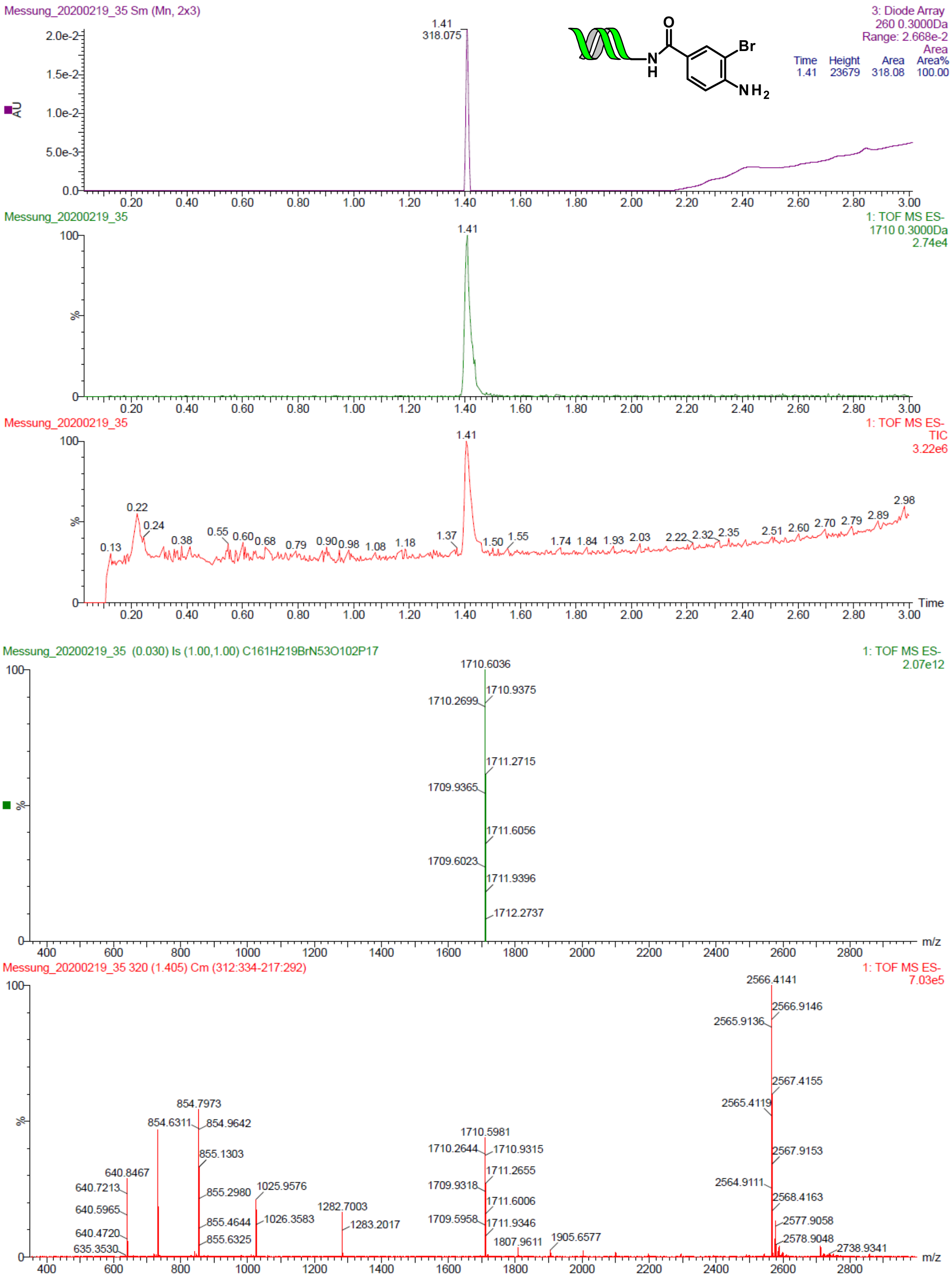

*Figure 3.60: LC-MS chromatogram of compound **Table 2 Entry 30 amine** t_R = 1.41 min TOF-MS-ESI$^-$ m/z = 1710.598(100%) $[M-3H]^{3-}$ calc. 1710.604 for $C_{161}H_{219}BrN_{53}O_{102}P_{17}$)*

Azido-4-Abz(3-Br)-OH conjugated with HP-280 (Table 2 Entry 30)

The reaction was carried out by using the general method DTR 2(1 µl, 10 mM of **(Table 2 Entry 30 amine)**, 10nmol scale).

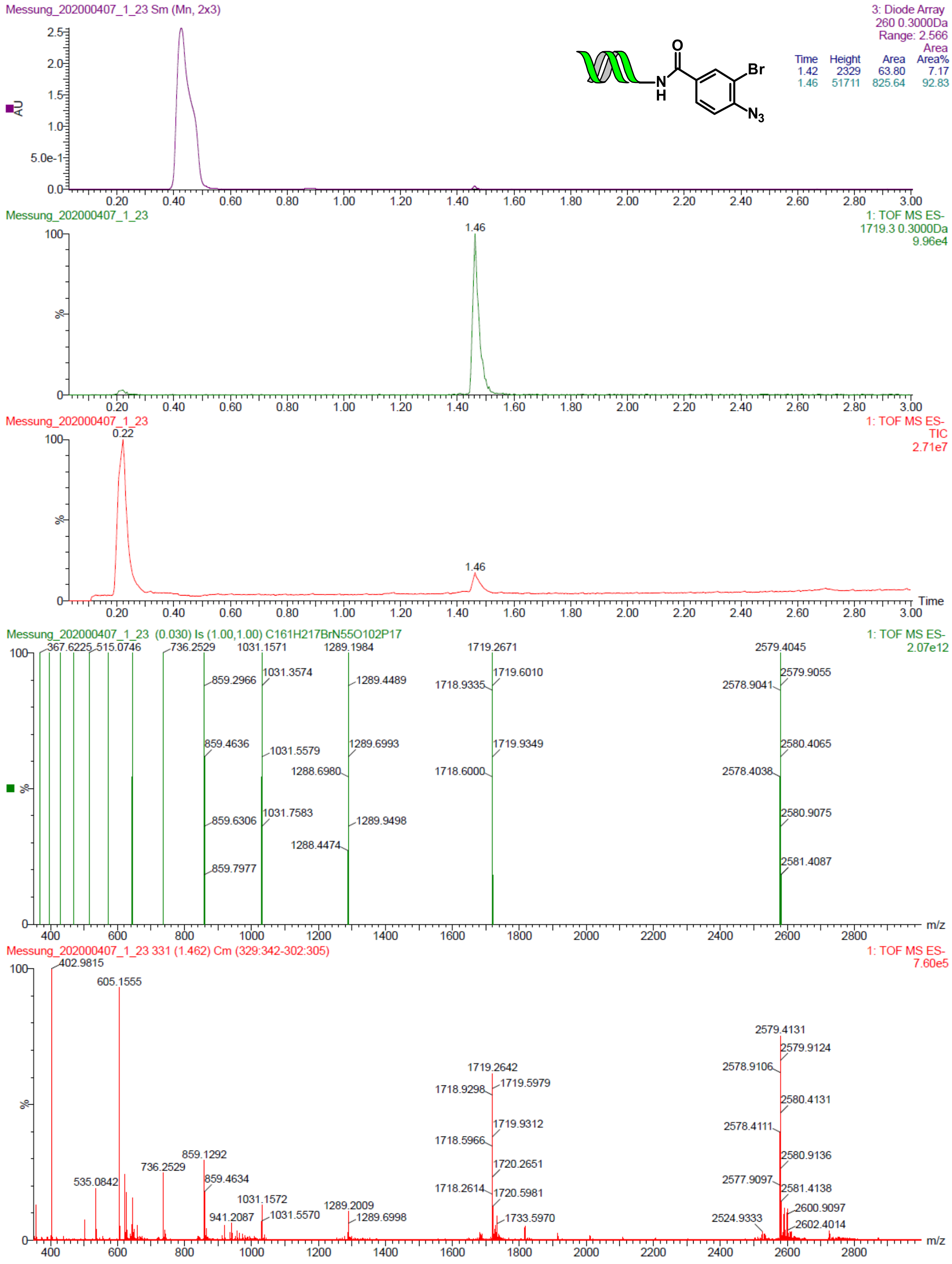

*Figure 3.61: LC-MS chromatogram of compound **Table 2 Entry 30** t_R = 1.42 min TOF-MS-ESI⁻ m/z = 1719.264(100%) $[M-3H]^{3-}$ (calc. 1719.267 for $C_{161}H_{217}BrN_{55}O_{102}P_{17}$)*

3.3.31 4-Azido-Abz(2-I)-OH conjugated with HP-280 (Table 2 Entry 31)

H-4-Abz(2-I)-OH conjugated with HP-280:

Fmoc-4-Abz(2-I)-OH (CAS2122-63-6 free amine) was conjugated using general method **ABF 2** (15 µl, 10 mM HP-280, 150 nmol scale) and purified with general purification method **GP1**. The Fmoc deprotection was carried out using general method for Fmoc deprotection and was purified using the methods **GP1** and **GP2.**

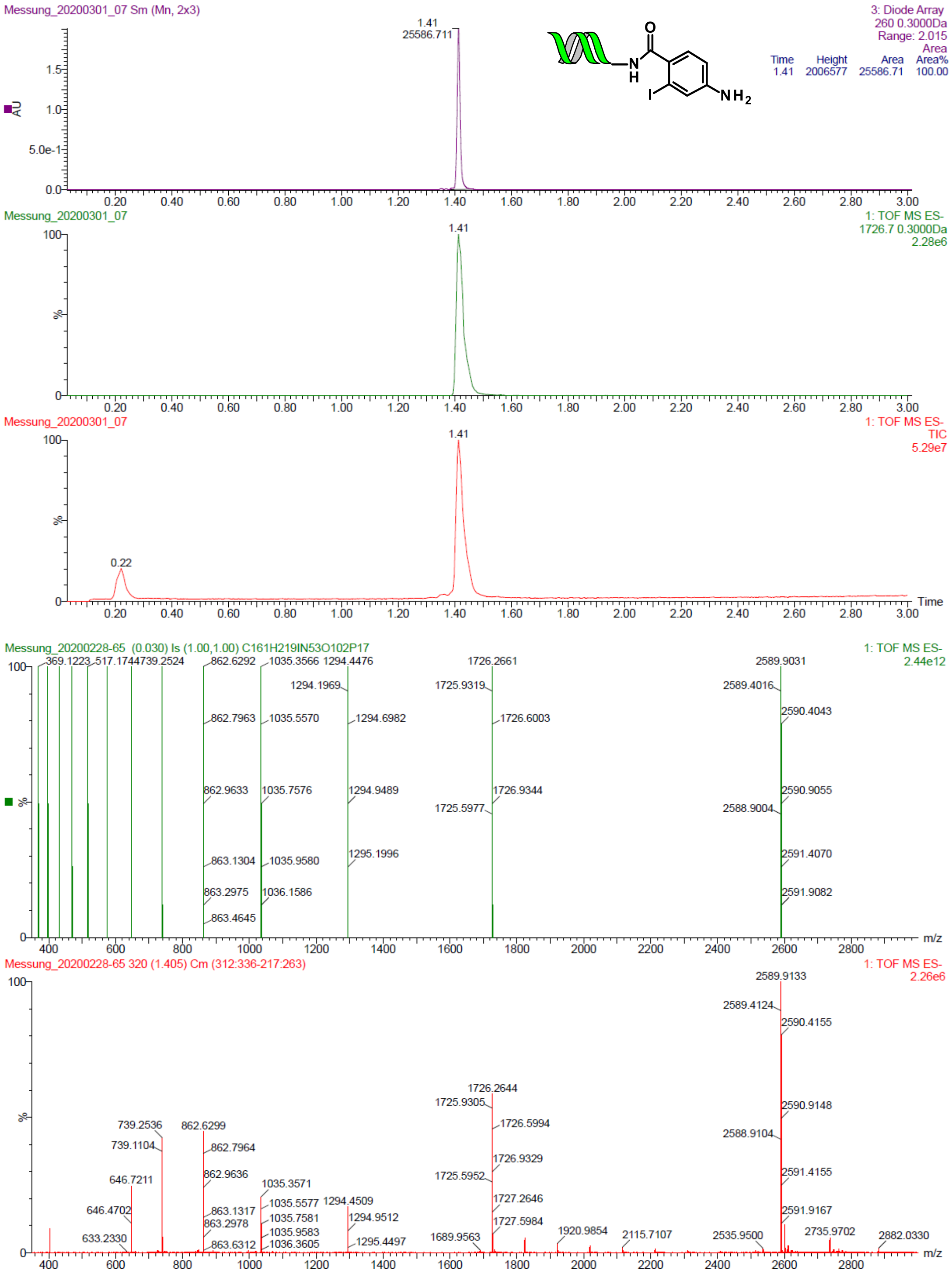

Figure 3.62: LC-MS chromatogram of compound ***Table 2 Entry 31 amine*** *t_R = 1,43 min TOF-MS-ESI$^-$ m/z = 1764,318(100%) $[M-3H]^{3-}$ (calc. 1764,320 for $C_{161}H_{219}IN_{53}O_{102}P_{17}$)*

4-Azido-Abz(2-I)-OH conjugated with HP-280 (Table 2 Entry 31):

The reaction was carried out by using the general method DTR 2(1 µl, 10 mM of **(Table 2 Entry 31 amine)**, 10nmol scale).

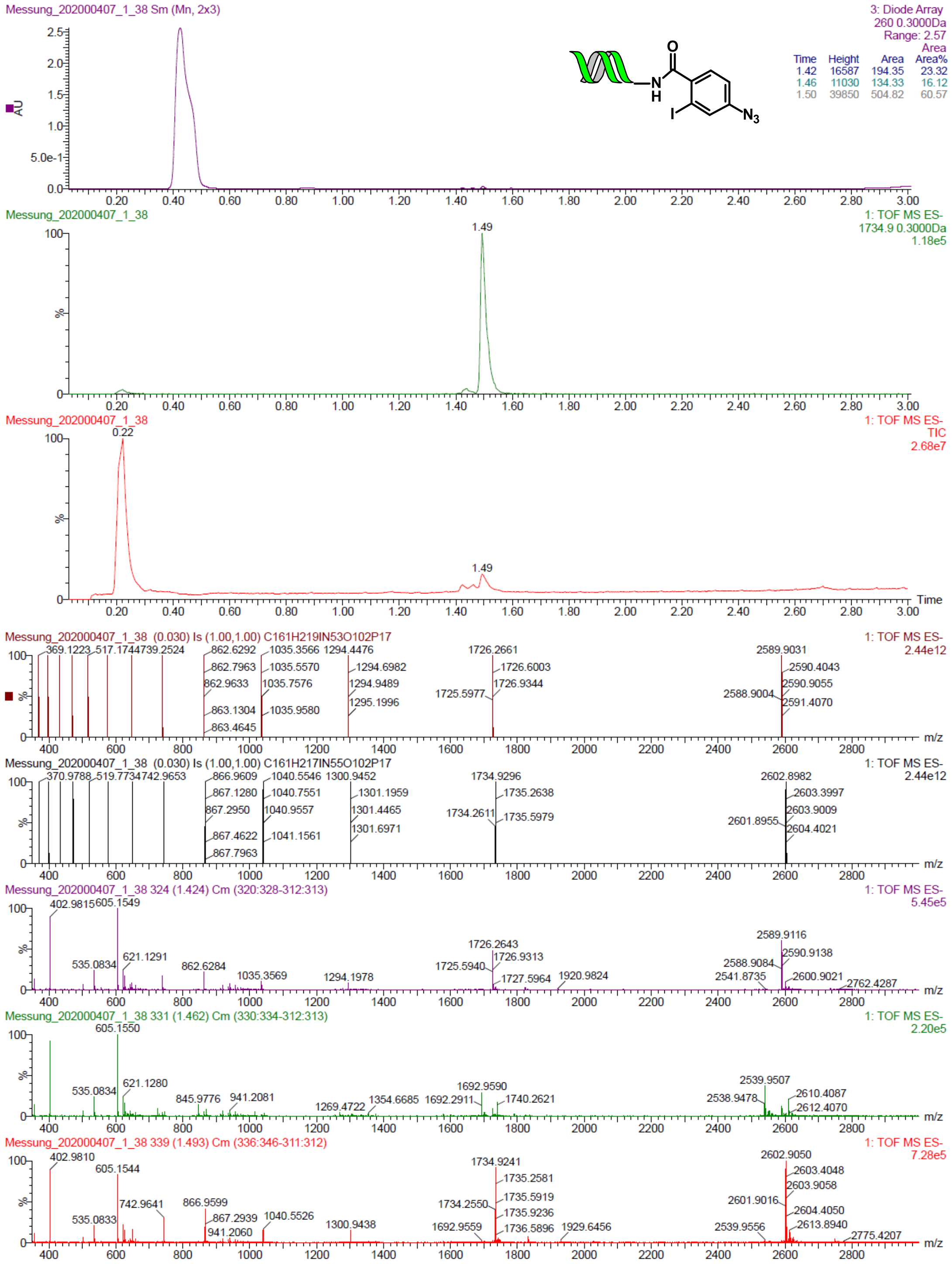

*Figure 3.63: LC-MS chromatogram of compound **Table 2 Entry 31** t_R = 1,50 min TOF-MS-ESI$^-$ m/z = 1734,924 (100%) $[M-3H]^{3-}$ (calc. 1734,930 for $C_{161}H_{217}IN_{55}O_{102}P_{17}$)*

3.3.32 5-Azidonicotinic acid conjugated with HP-280 (Table 2 Entry 32)

5-Aminonicotinic acid conjugated with HP-280:

Fmoc-5-aminonicotinic acid (CAS 457100-77-5) was conjugated using general method **ABF 2** (15 µl, 10 mM HP-280, 150 nmol scale) and purified with general purification method **GP1**. The Fmoc deprotection was carried out using general method for Fmoc deprotection and was purified using the methods **GP1** and **GP2.**

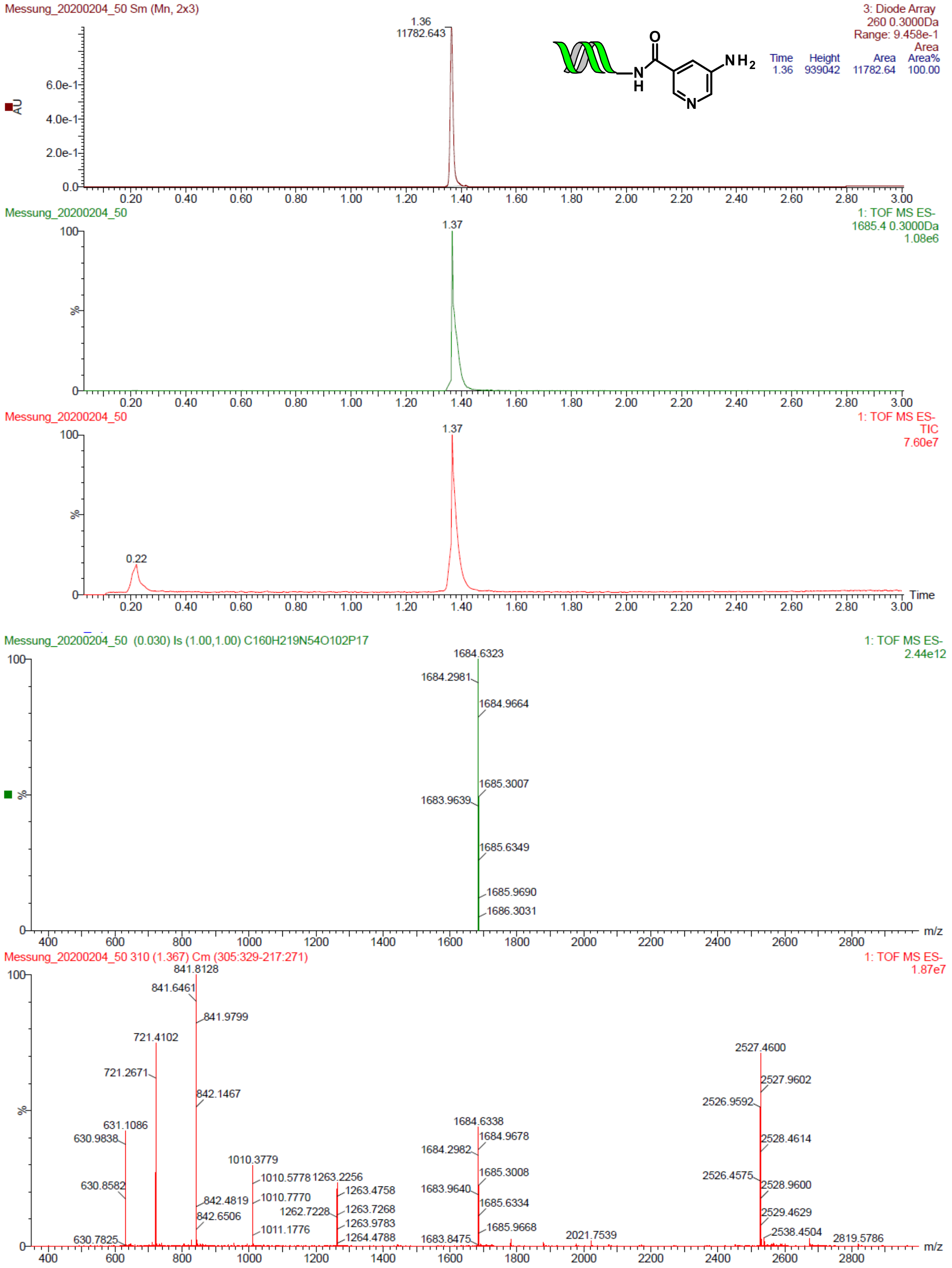

Figure 3.64: : LC-MS chromatogram of compound ***Table 2 Entry 32 amine*** *t_R = 1,36 min TOF-MS-ESI$^-$ m/z = 1684,632(100%) $[M-3H]^{3-}$ (calc. 1684,632 for $C_{160}H_{219}N_{54}O_{102}P_{17}$)*

5-Azidonicotinic acid conjugated with HP-280 (Table 2 Entry 32):

The reaction was carried out by using the general method DTR 2(1 µl, 10 mM of **(Table 2 Entry 32 amine)**, 10nmol scale).

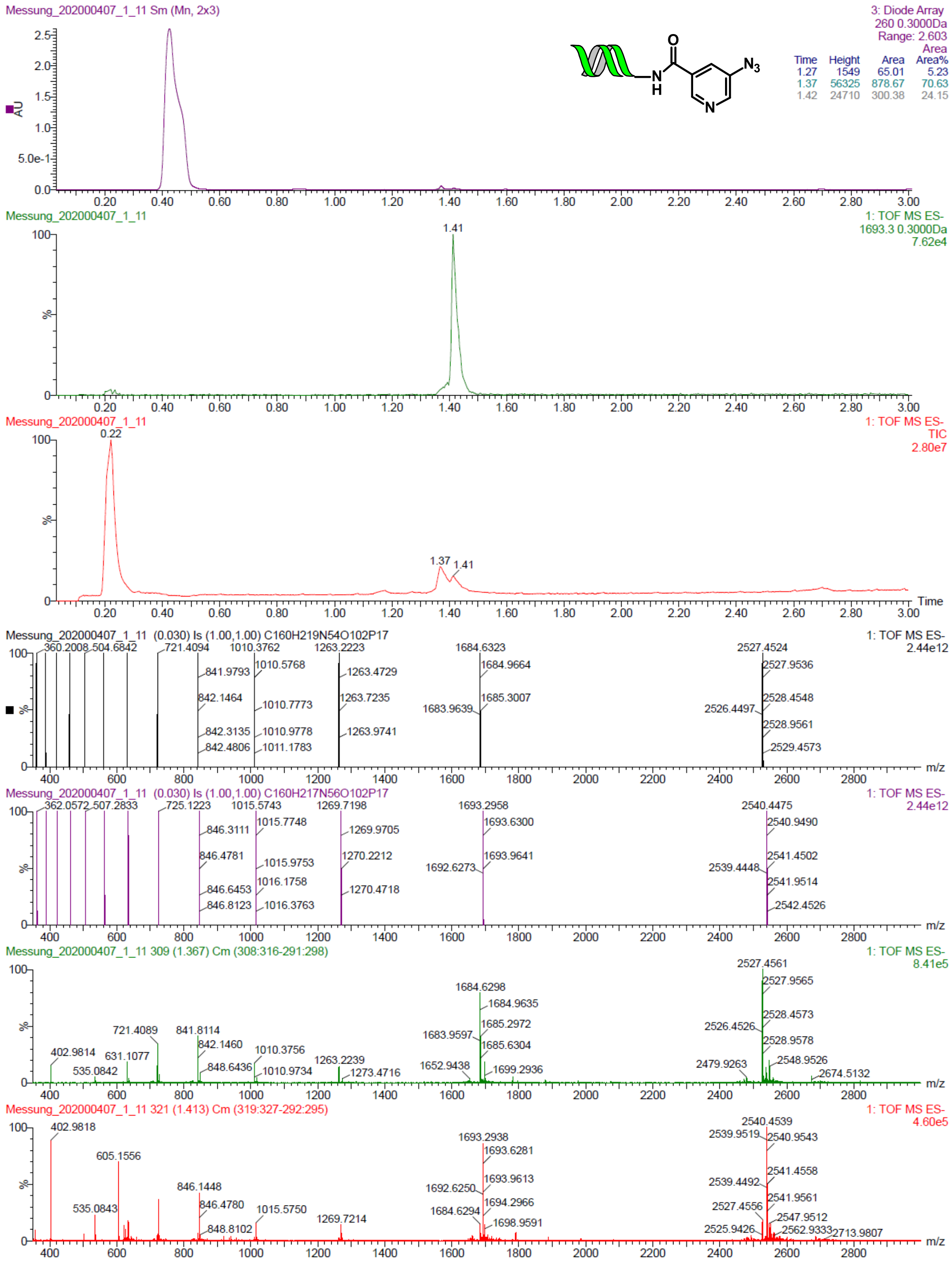

*Figure 3.65: LC-MS chromatogram of compound **Table 2 Entry 32** t_R = 1,42 min TOF-MS-ESI$^-$ m/z = 1693,294(100%) [M-3H]$^{3-}$ (calc. 1693,296 for $C_{160}H_{217}N_{56}O_{104}P_{17}$)*

3.3.33 3-Azidopicolinic acid conjugated with HP-280 (Table 2 Entry 33)

3-Aminopicolinic acid conjugated with HP-280:

Fmoc-3-Aminopicolinic acid (CAS 1567020-33-0) was conjugated using general method **ABF 2** (15 µl, 10 mM HP-280, 150 nmol scale) and purified with general purification method **GP1**. The Fmoc deprotection was carried out using general method for Fmoc deprotection and was purified using the methods **GP1** and **GP2.**

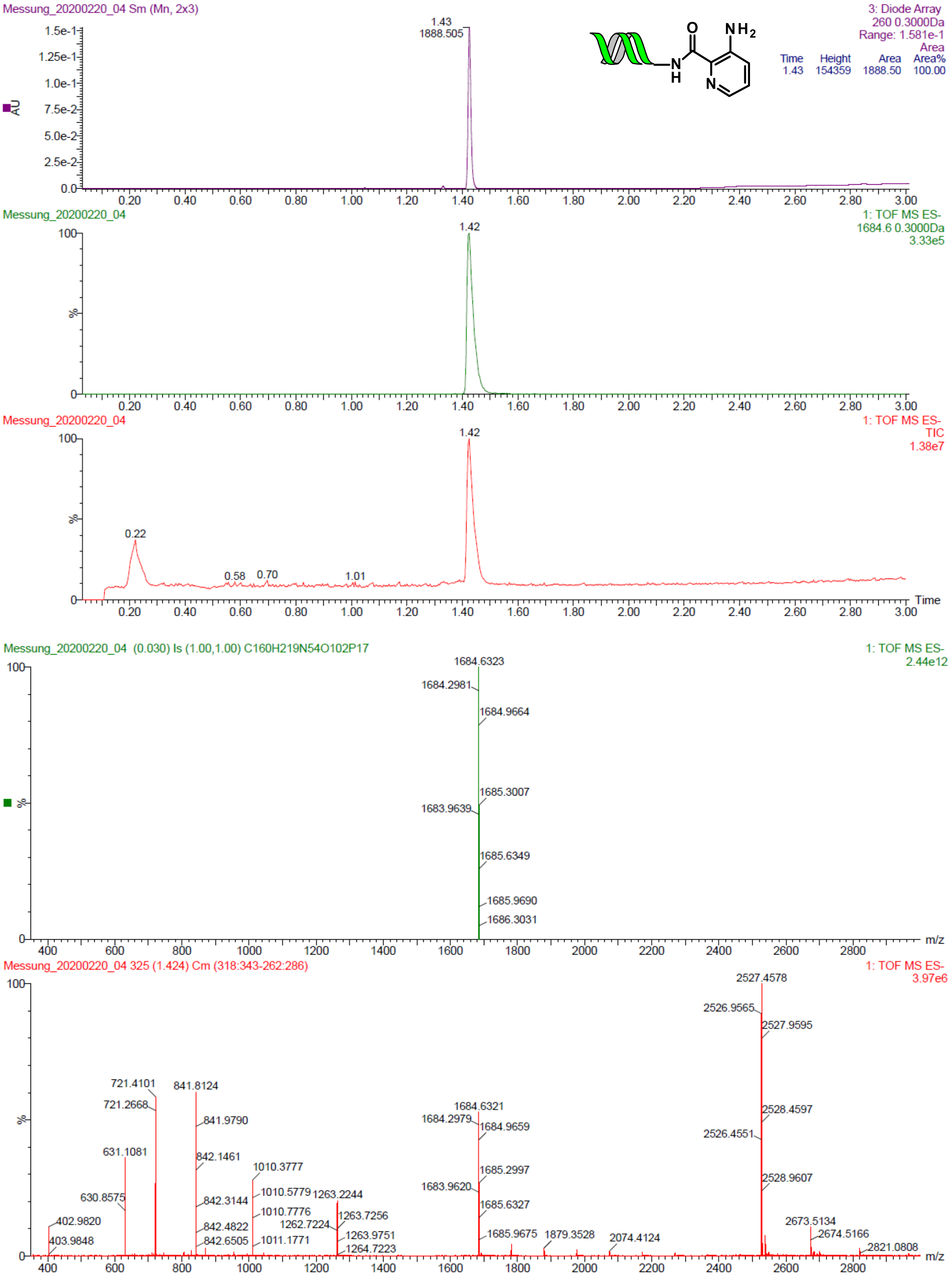

*Figure 3.66: LC-MS chromatogram of compound **Table 2 Entry 33 amine** t_R = 1,36 min TOF-MS-ESI$^-$ m/z = 1684,632(100%) [M-3H]$^{3-}$ (calc. 1684,632 for $C_{160}H_{219}N_{54}O_{102}P_{17}$)*

3-Azidopicolin acid conjugated with HP-280 (Table 2 Entry 33)

The reaction was carried out by using the general method DTR 2(1 µl, 10 mM of **(Table 2 Entry 33 amine)**, 10nmol scale).

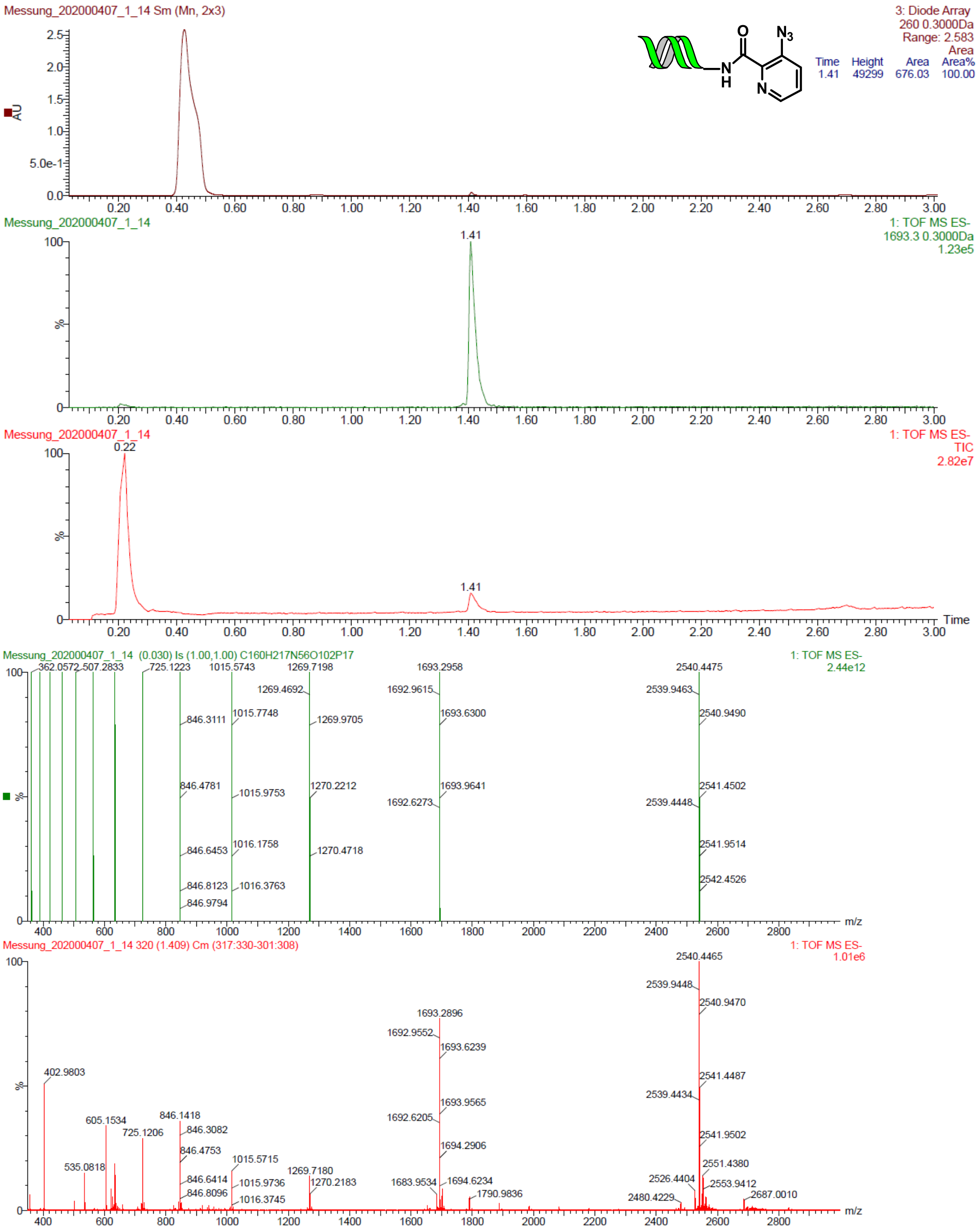

*Figure 3.67: LC-MS chromatogram of compound **Table 2 Entry 33** t_R = 1,41 min TOF-MS-ESI$^-$ m/z = 1693,290(100%) [M-3H]$^{3-}$ (calc. 1693,296 for $C_{160}H_{217}N_{56}O_{104}P_{17}$)*

3.3.34 6-Azidonicotinic acid conjugated with HP-280 (Table 2 Entry 34)

6-Aminonicotinic acid conjugated with HP-280:

Fmoc-6-Aminonicotinic acid (CAS3167-49-5 free amine) was conjugated using general method **ABF 2** (15 µl, 10 mM HP-280, 150 nmol scale) and purified with general purification method **GP1**. The Fmoc deprotection was carried out using general method for Fmoc deprotection and was purified using the methods **GP1** and **GP2.**

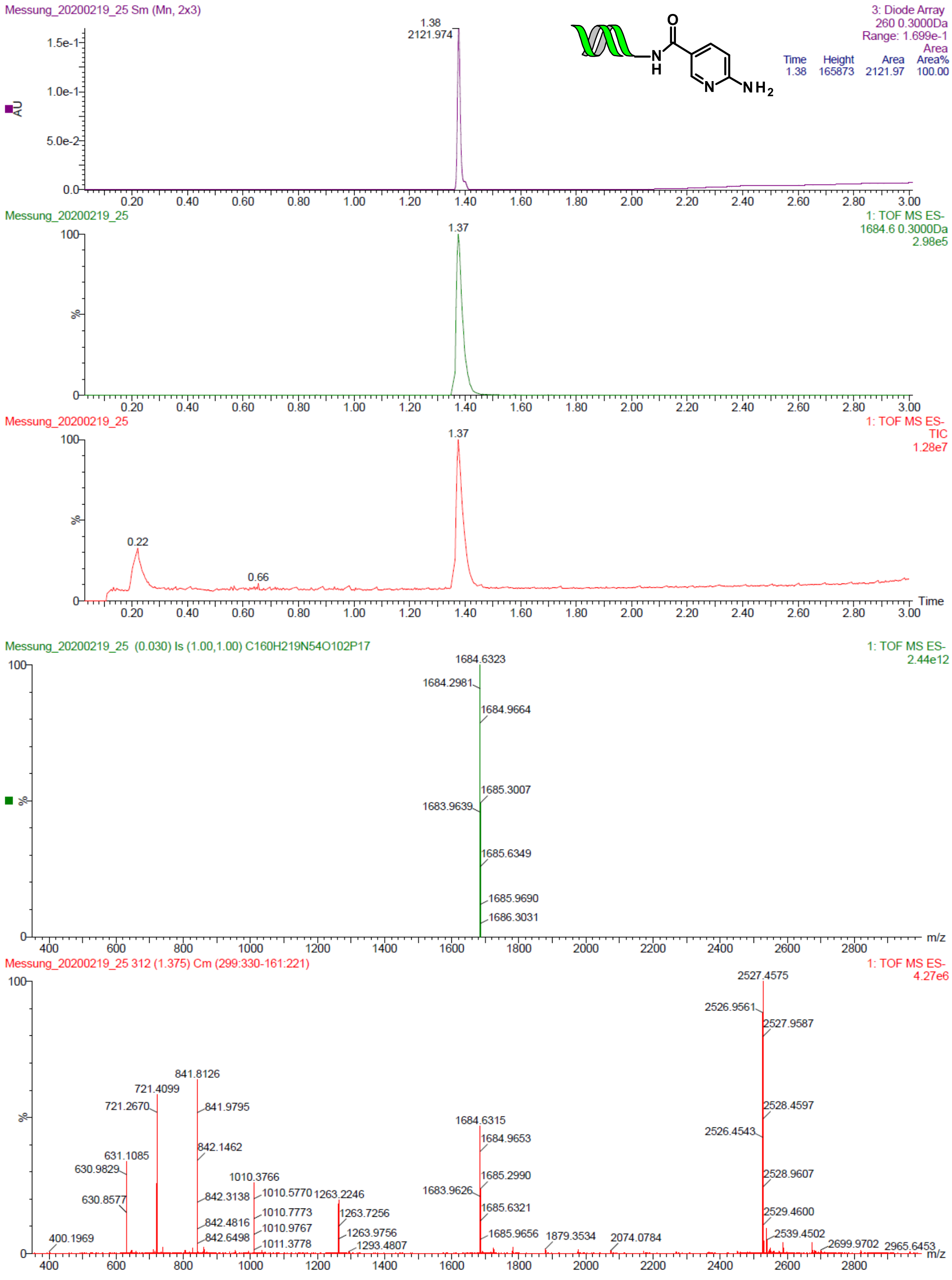

*Figure 3.68: LC-MS chromatogram of compound **Table 2 Entry 34 amine** t_R = 1,38 min TOF-MS-ESI$^-$ m/z = 1684,632(100%) [M-3H]$^{3-}$ (calc. 1684,632 for $C_{160}H_{219}N_{54}O_{102}P_{17}$)*

6-Azidonicotinic acid conjugated with HP-280 (Table 2 Entry 34):

The reaction was carried out by using the general method DTR 2(1 µl, 10 mM of **(Table 2 Entry 34 amine)**, 10nmol scale).

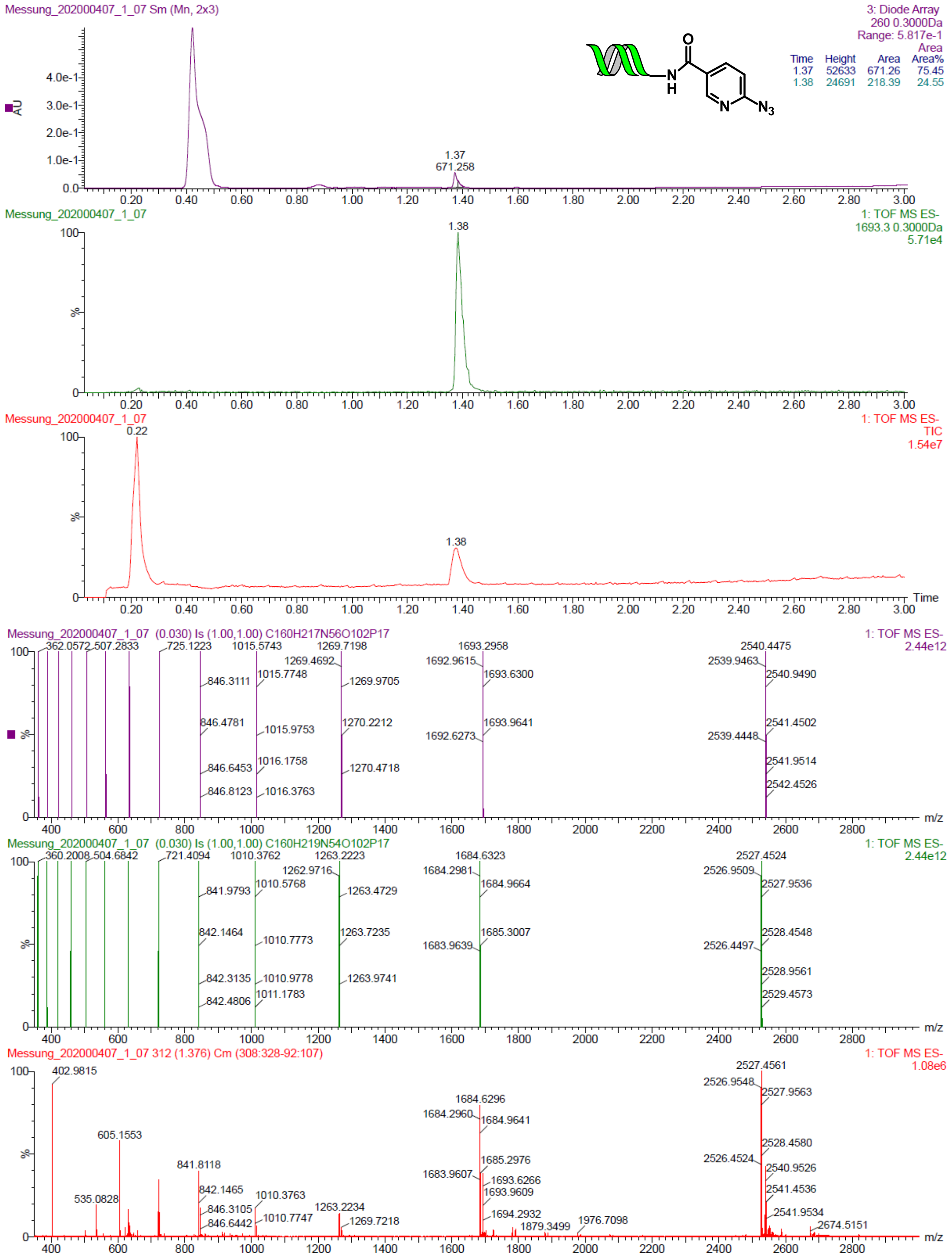

*Figure 3.69: LC-MS chromatogram of compound **Table 2 Entry 34** t_R = 1,38 min TOF-MS-ESI$^-$ m/z = 1693,296(100%) [M-3H]$^{3-}$ (calc. 1693,296 for $C_{160}H_{217}N_{56}O_{104}P_{17}$)*

3.3.35 2-Azidopyrimidine-5-carboxylic acid conjugated with HP-280 (Table 2 Entry 35)

2-Aminopyrimidine-5-carboxylic acid conjugated with HP-280:

2-Aminopyrimidine-5-carboxylic acid (CAS 3167-50-8) was conjugated using general method **ABF 2** (15 µl, 10 mM HP-280, 150 nmol scale) and purified with general purification method **GP1**. The Fmoc deprotection was carried out using general method for Fmoc deprotection and was purified using the methods **GP1** and **GP2.**

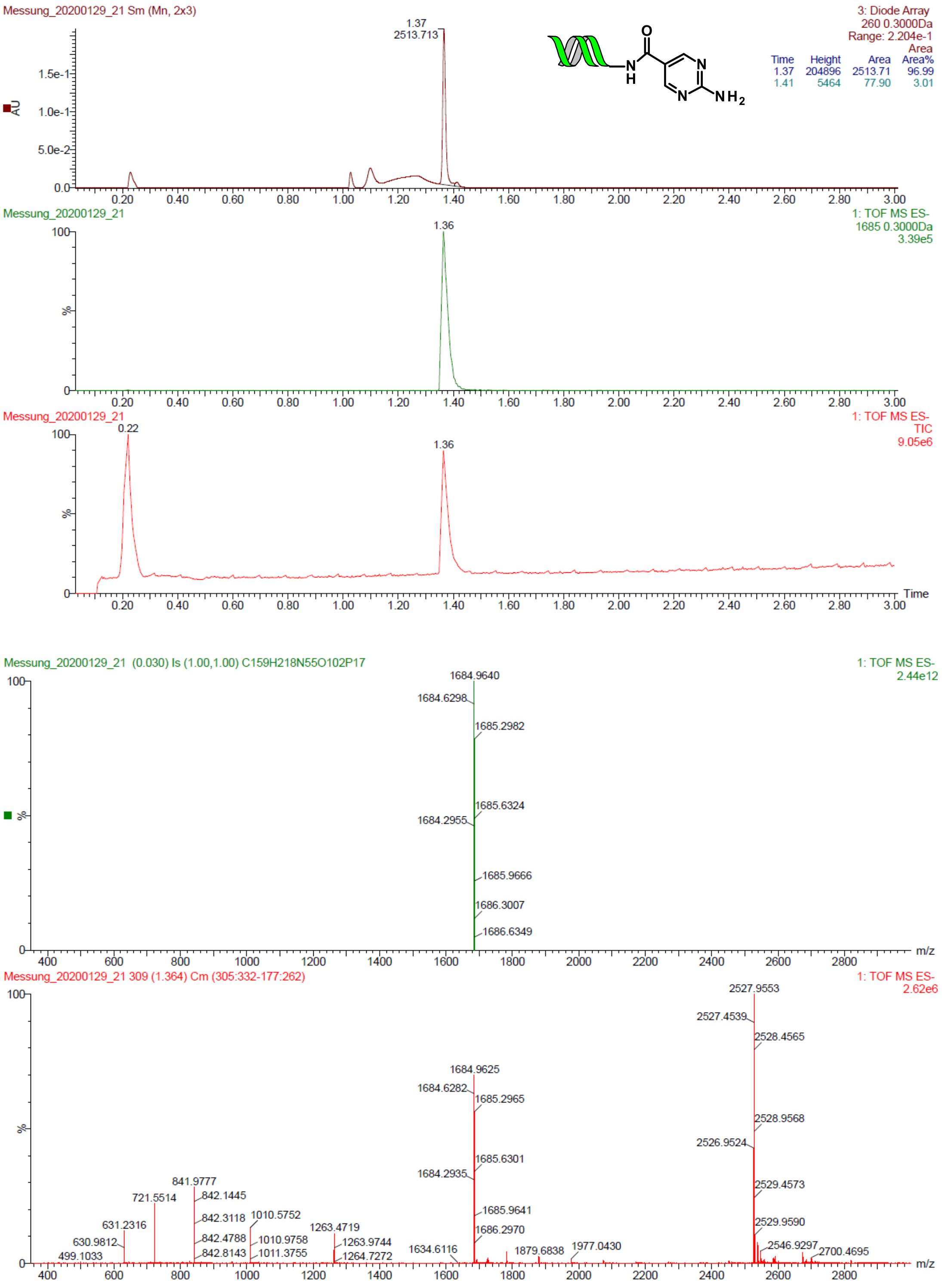

*Figure 3.70: LC-MS chromatogram of compound **Table 2 Entry 35 amine** t_R = 1,37 min TOF-MS-ESI$^-$ m/z = 1684,963(100%) [M-3H]$^{3-}$ (calc. 1684,964 for $C_{159}H_{218}N_{55}O_{102}P_{17}$)*

2-Azidopyrimidine-5-carboxylic acid conjugated with HP-280 (Table 2 Entry 35):

The reaction was carried out by using the general method DTR 2(1 µl, 10 mM of **(Table 2 Entry 35 amine)**, 10nmol scale).

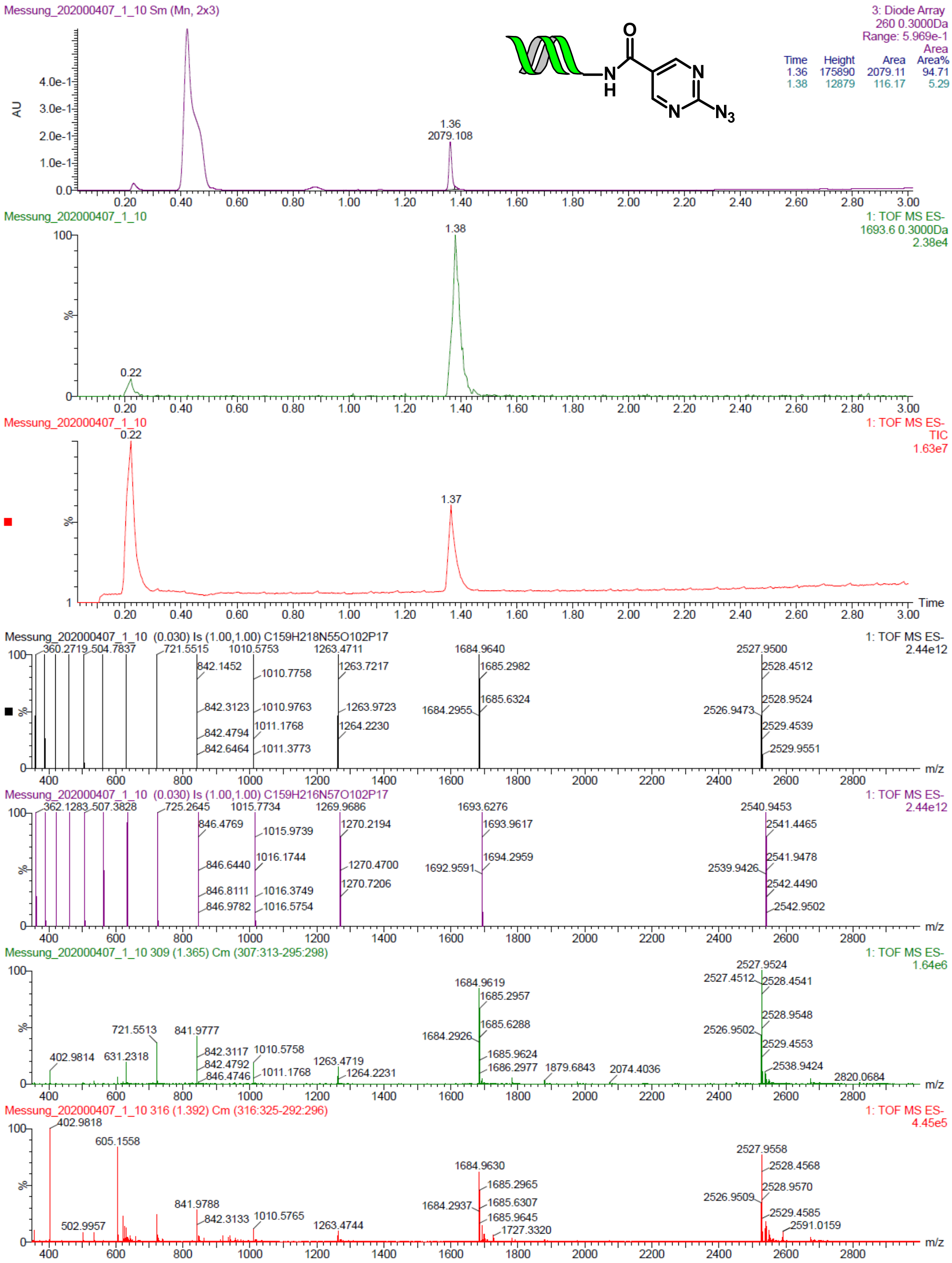

*Figure 3.71: LC-MS chromatogram of compound **Table 2 Entry 35** t_R = 1,34 min TOF-MS-ESI$^-$ m/z = 1684,624(100%) [M-3H]$^{3-}$ (starting material) (calc. 1693,628 for $C_{159}H_{216}N_{57}O_{102}P_{17}$)*

3.3.36 4-Azido-1-*H*-pyrazole-1-acetic acid conjugated with HP-280 (Table 2 Entry 36)

4-Amino-1-*H*-pyrazole-1-acetic acid conjugated with HP-280:

Fmoc-4-Amino-1-H-pyrazole-1-acetic acid (CAS 1341762-24-0) was conjugated using general method **ABF 2** (15 µl, 10 mM HP-280, 150 nmol scale) and purified with general purification method **GP1**. The Fmoc deprotection was carried out using general method for Fmoc deprotection and was purified using the methods **GP1** and **GP2.**

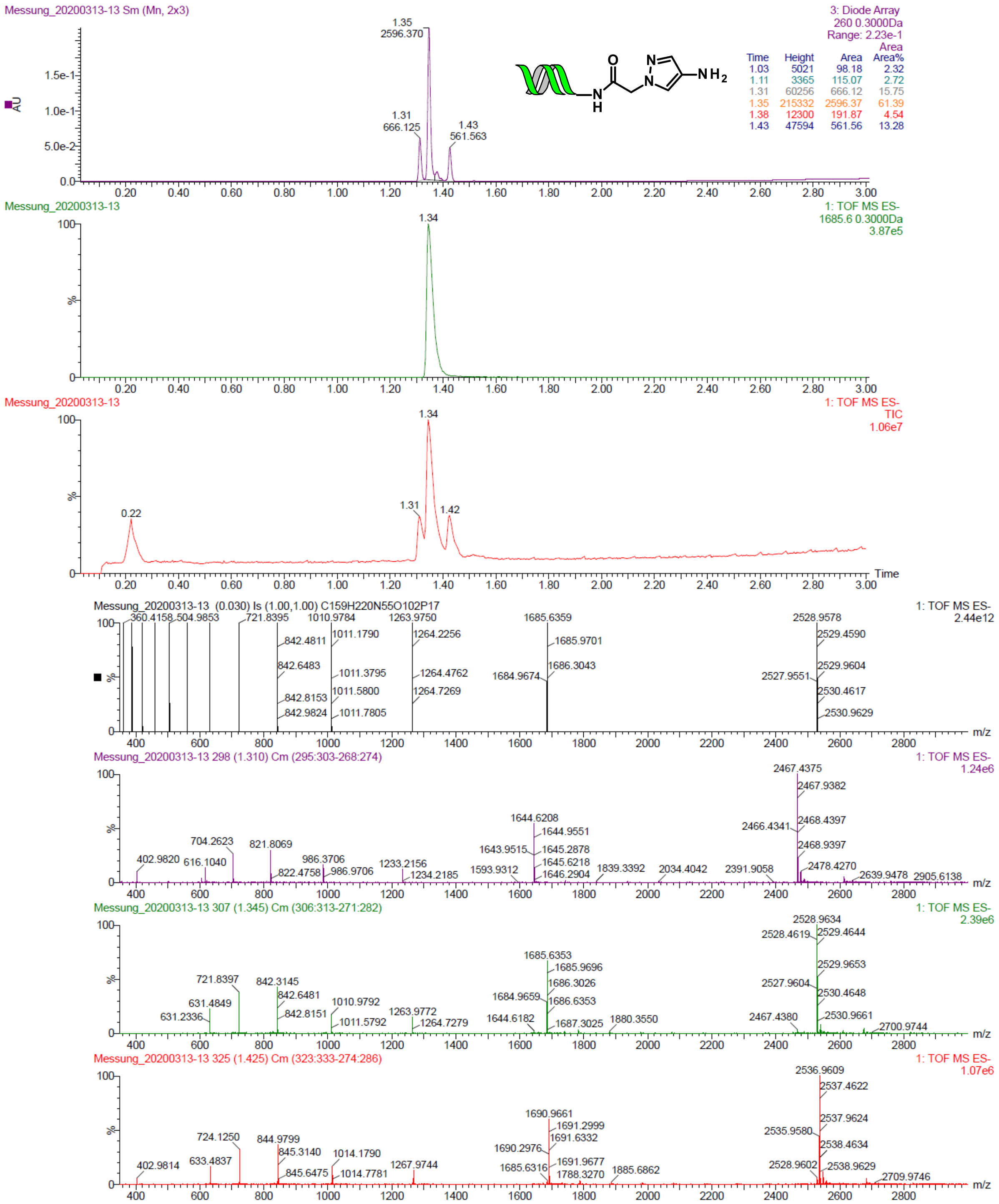

Figure 3.72: LC-MS chromatogram of compound ***Table 2 Entry 36 amine*** *t_R = 1,35 min TOF-MS-ESI$^-$ m/z = 1684,632(100%) [M-3H]$^{3-}$ (calc. 1684,632 for $C_{160}H_{219}N_{54}O_{102}P_{17}$)*

4-Azido-1-*H*-pyrazol-1-acetic acid conjugated with HP-280 (Table 2 Entry 36):

The reaction was carried out by using the general method DTR 2(1 µl, 10 mM of **(Table 2 Entry 36 amine)**, 10nmol scale).

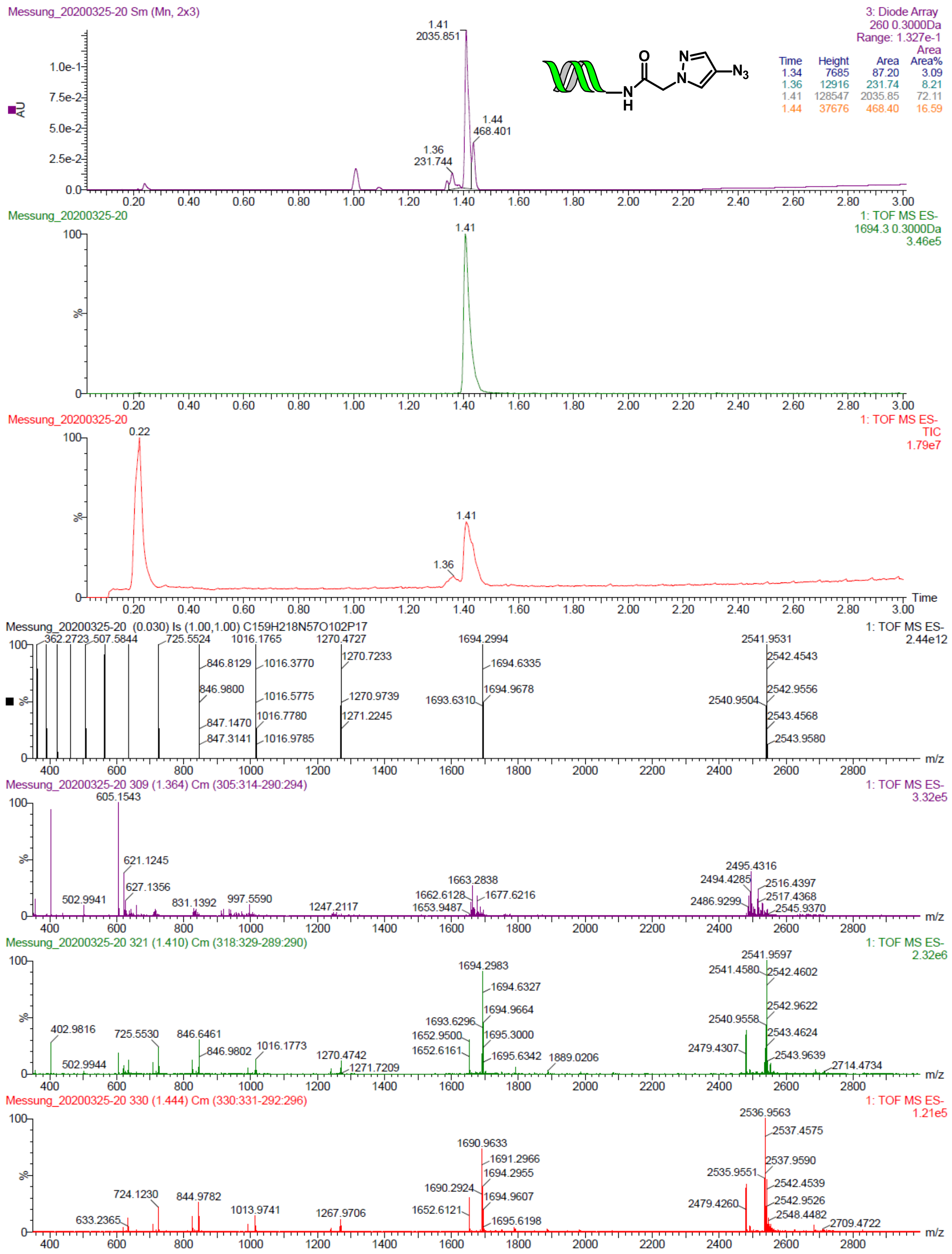

Figure 3.73: LC-MS chromatogram of compound ***Table 2 Entry 36*** *t_R = 1,41min TOF-MS-ESI⁻ m/z = 1694.298(100%) $[M-3H]^{3-}$ (calc. 1694.299 for $C_{159}H_{218}N_{57}O_{102}P_{17}$)*

3.4 Chromatograms of DNA-conjugated Tripeptides and Cyclization

The following tripeptides were built with the following methods:

TABLE 3: Overview of the Macrocycle Syntheses on-DNA

Entry	AA1	AA2	AA3	Ring size of cyclized product 7
1	Pra	L-Phe	3-Abz	n = 13
2	Pra	L-Phe	2-Abz	n = 12
3	Pra	L-Phe	ß-Ala	n = 12
4	Pra	L-Trp	ß-Ala	n = 12
5	Pra	L-Trp	Gly	n = 11
6	Pra	L-Trp	γ-Abu	n = 13
7	Pra	ß-Ala	ß-Ala	n = 13
8	Pra	ß-Ala	γ-Abu	n = 14
9	Pra	ß-Ala	3-Abz	n = 14

The DNA conjugated Pra-OH (**Table 2 Entry 11)** was split into three portions of 114 µl (10mM aq.). The second amino acids (Fmoc-Phe-OH, Fmoc-Trp-OH and Fmoc-β-Ala-OH) were coupled with the general method **ABF 1** and followed by purification method **GP 1**. The Fmoc-groups were deprotected by the general deprotection method and then purified by general methods **GP 1** and **GP 2**.

The three dipeptides described above were each split into three portions of 38 µl (10mM aq.). The third amino acids (Fmoc-3-Abz-OH or Fmoc-2-Abz-OH) was coupled using the **ABF 2** method and (Fmoc-β-Ala-OH, Fmoc-Gly-OH or Fmoc-γ-Abu-OH) was coupled using the general method **ABF 1** followed by general method **GP 1**. The Fmoc-groups were deprotected with the general deprotection method and then purified by general method **GP 1** and **GP 2**.

3.4.1 [cyclo-3-Abz-Phe-Pra]-OH conjugated with HP-280 (Table 3 Entry 1)

H-3-Abz-Phe-Pra-OH conjugated with HP-280 (Table 3 Entry 1 amine):

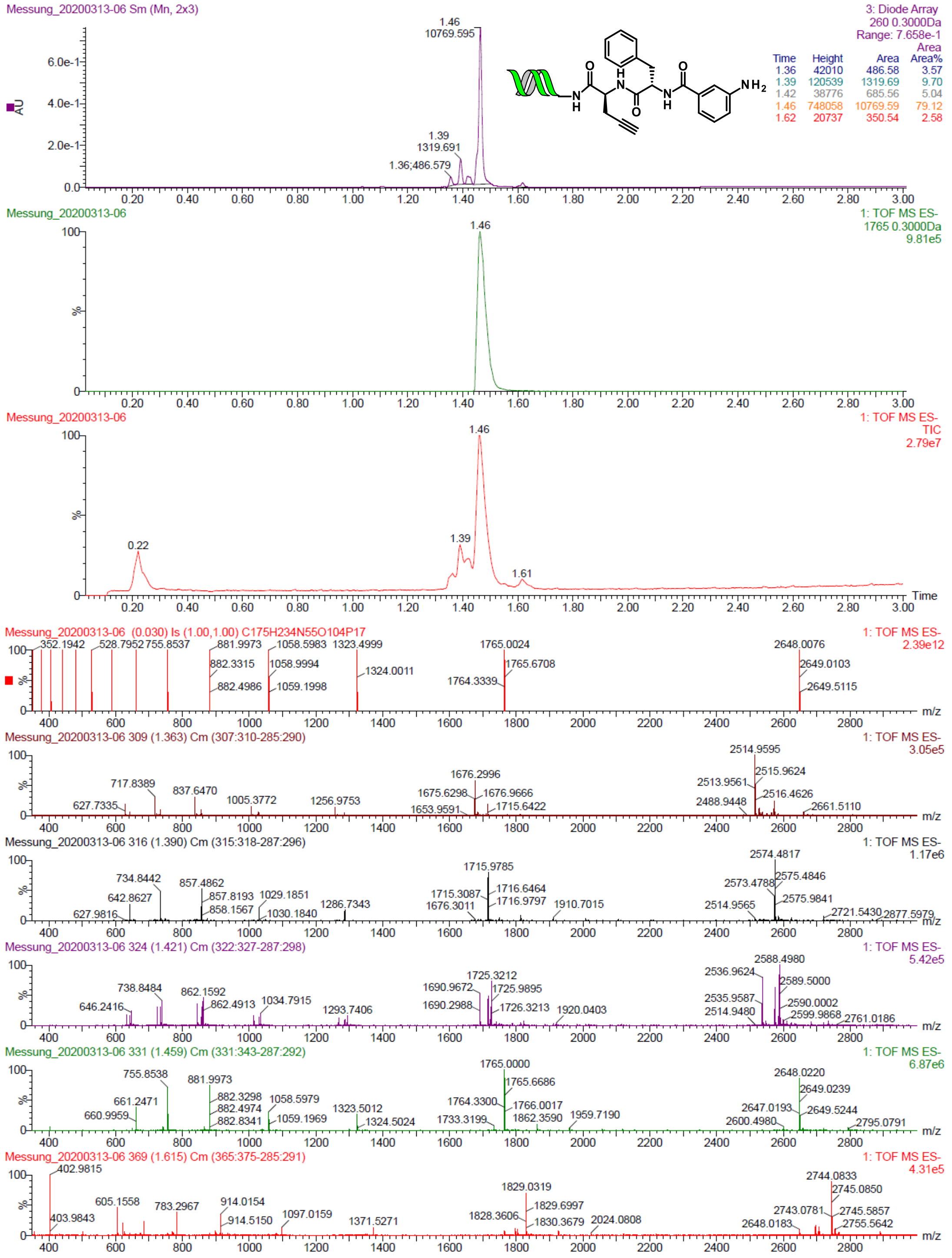

*Figure 3.74: : LC-MS chromatogram of compound **Table 3 Entry 1 amine** t_R = 1,46 min TOF-MS-ESI$^-$ m/z = 1765.000(100%) $[M-3H]^{3-}$ (calc. 1765.002 for $C_{175}H_{234}N_{55}O_{104}P_{17}$)*

Azido-3-Abz-Phe-Pra-OH conjugated with HP-280 (Table 3 Entry 1 Azide):

The reaction was carried out by using the general method DTR 2(15 µl, 10 mM of **(Table 3 Entry 1 amine)**, 150nmol scale) followed by general method **GP 1**.

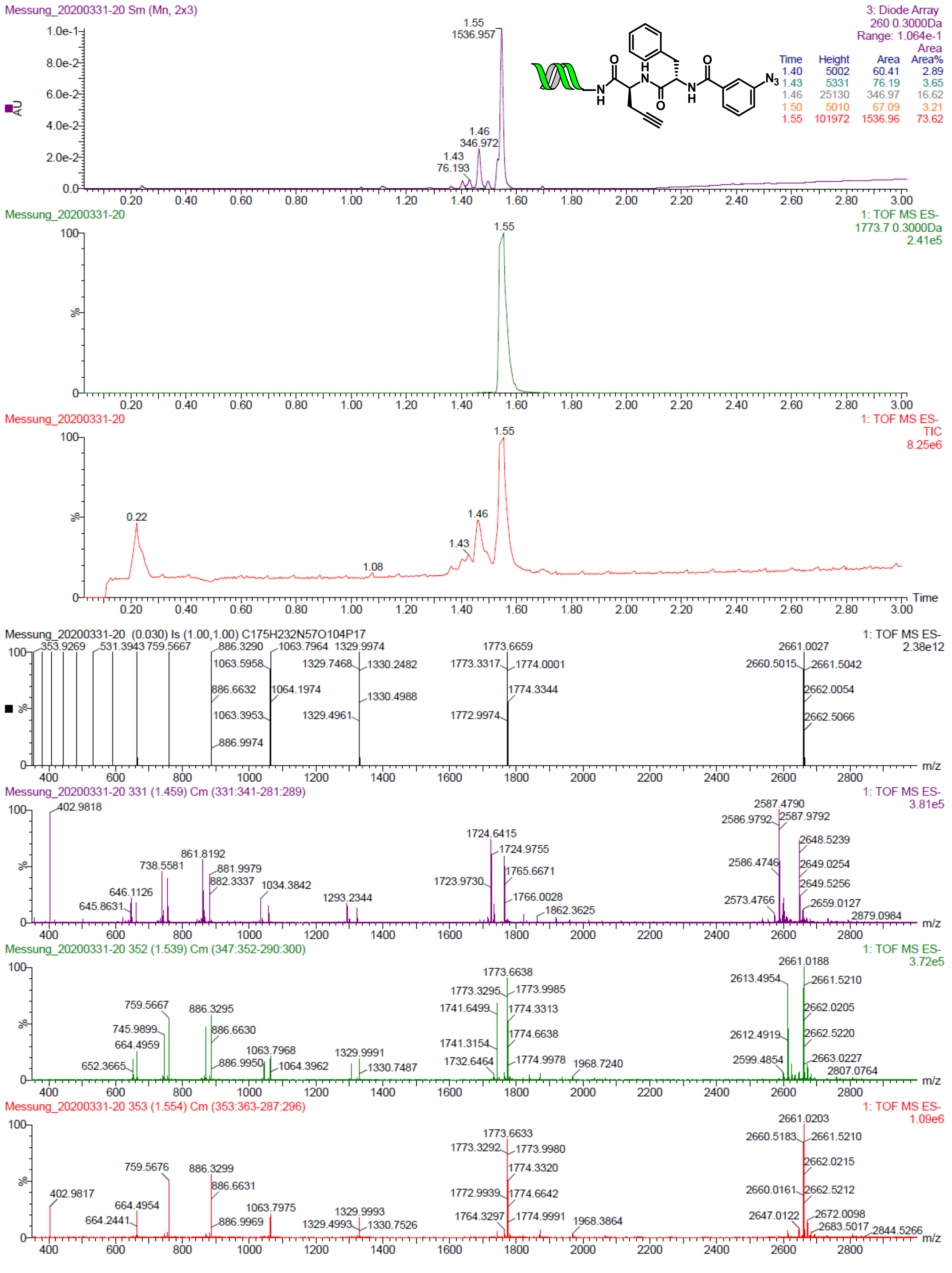

Figure 3.75: LC-MS chromatogram of compound ***Table 3 Entry 1 azide*** *t_R = 1,55 min TOF-MS-ESI$^-$ m/z = 1773.663(100%) [M-3H]$^{3-}$ (calc. 1773.666 for $C_{171}H_{232}N_{57}O_{104}P_{17}$)*

Azido-3-Abz-Phe-Pra-OH conjugated with HP-280 2. Synthesis (Table 3 Entry 1 Azide):

Compound **15** was conjugated using general method **ABF 1** (10 µl, 10 mM of **(HP-280)**, 100nmol scale) followed by general method **GP 1**.

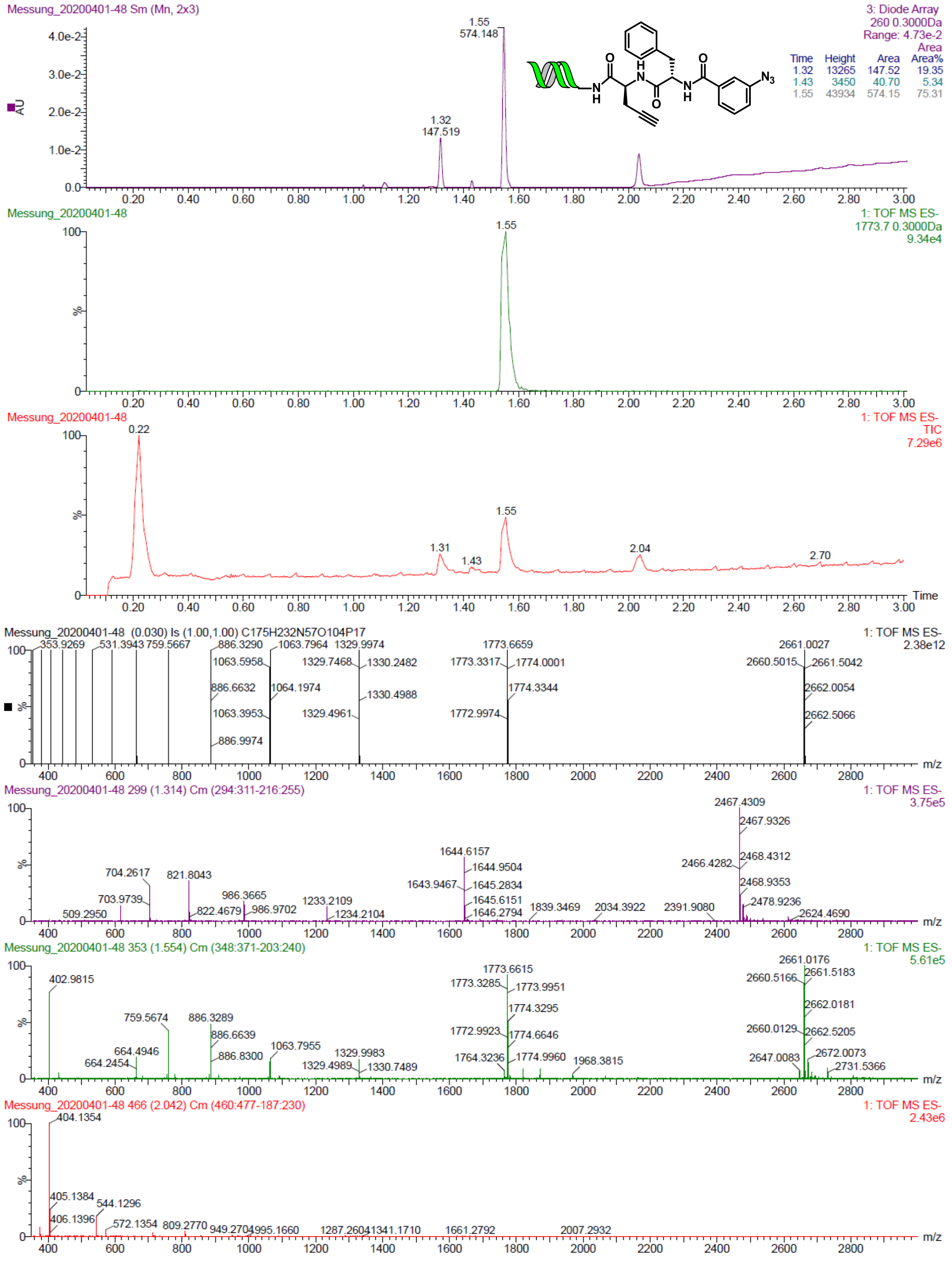

*Figure 3.76: LC-MS chromatogram of compound **Table 3 Entry 1 azide** t_R = 1,55 min TOF-MS-ESI$^-$ m/z = 1773.662(100%) [M-3H]$^{3-}$ (calc. 1773.666 for $C_{171}H_{232}N_{57}O_{104}P_{17}$)*

[cyclo-3-Abz-Phe-Pra]-OH conjugated with HP-280 (Table 3 Entry 1 Cyclo-Triazole):

The reaction was carried out using method **CuAAC 1** method (1 µl, 10 mM of **(Table 3 Entry 1 azide)**, 10nmol scale).

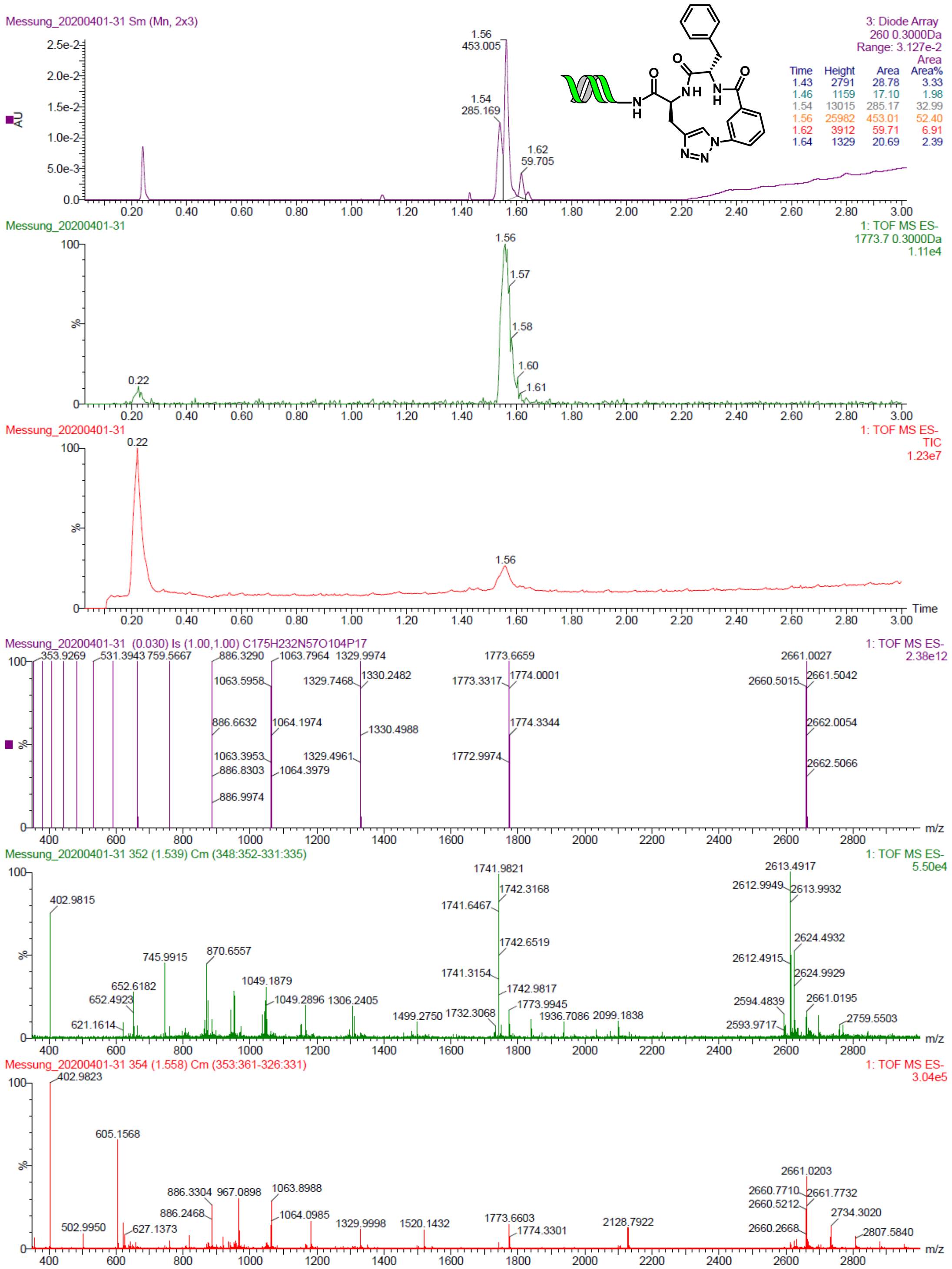

*Figure 3.77: LC-MS chromatogram of compound **Table 3 Entry 1 cyclo-triazole** t_R = 1,54+1.56 min TOF-MS-ESI$^-$ m/z = 1757.660(100%) [M-3H]$^{3-}$ (calc. 1757.666 for $C_{171}H_{232}N_{57}O_{104}P_{17}$)*

Linear Triazole of Azido-3-Abz-Phe-Pra-OH conjugated with HP-280 (Table 3 Entry 1 Linear-Triazole):

The reaction was carried out using method **CuAAC 2** (1 µl, 10 mM of **(Table 3 Entry 1 azide)**, 10nmol scale).

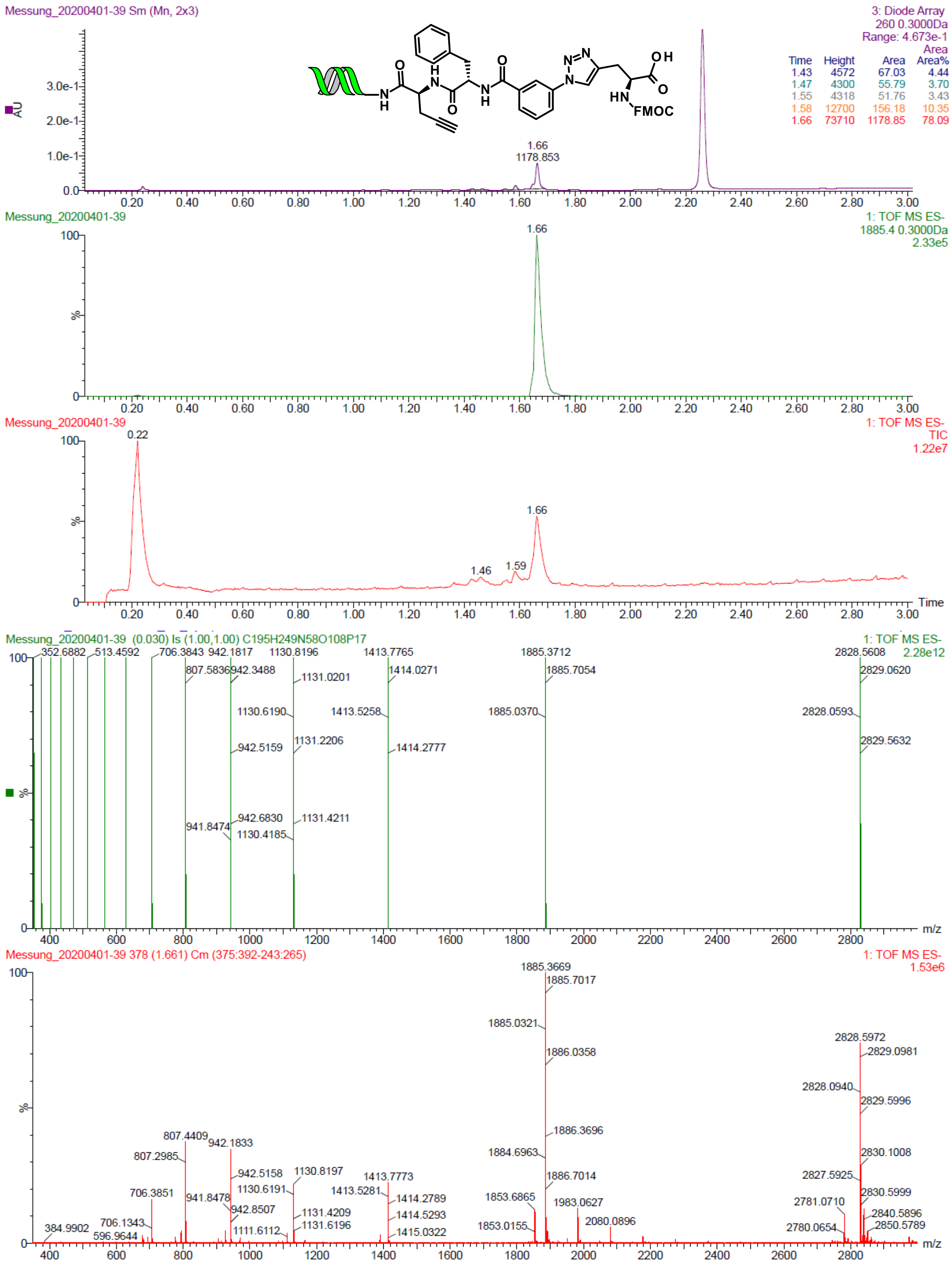

*Figure 3.78: LC-MS Chromatogram of compound **Table 3 Entry 1 linear triazole** t_R = 1,48 min TOF-MS-ESI⁻ m/z = 1885.370(100%) $[M-3H]^{3-}$ (calc. 1885.371 for $C_{195}H_{249}N_{58}O_{108}P_{17}$)*

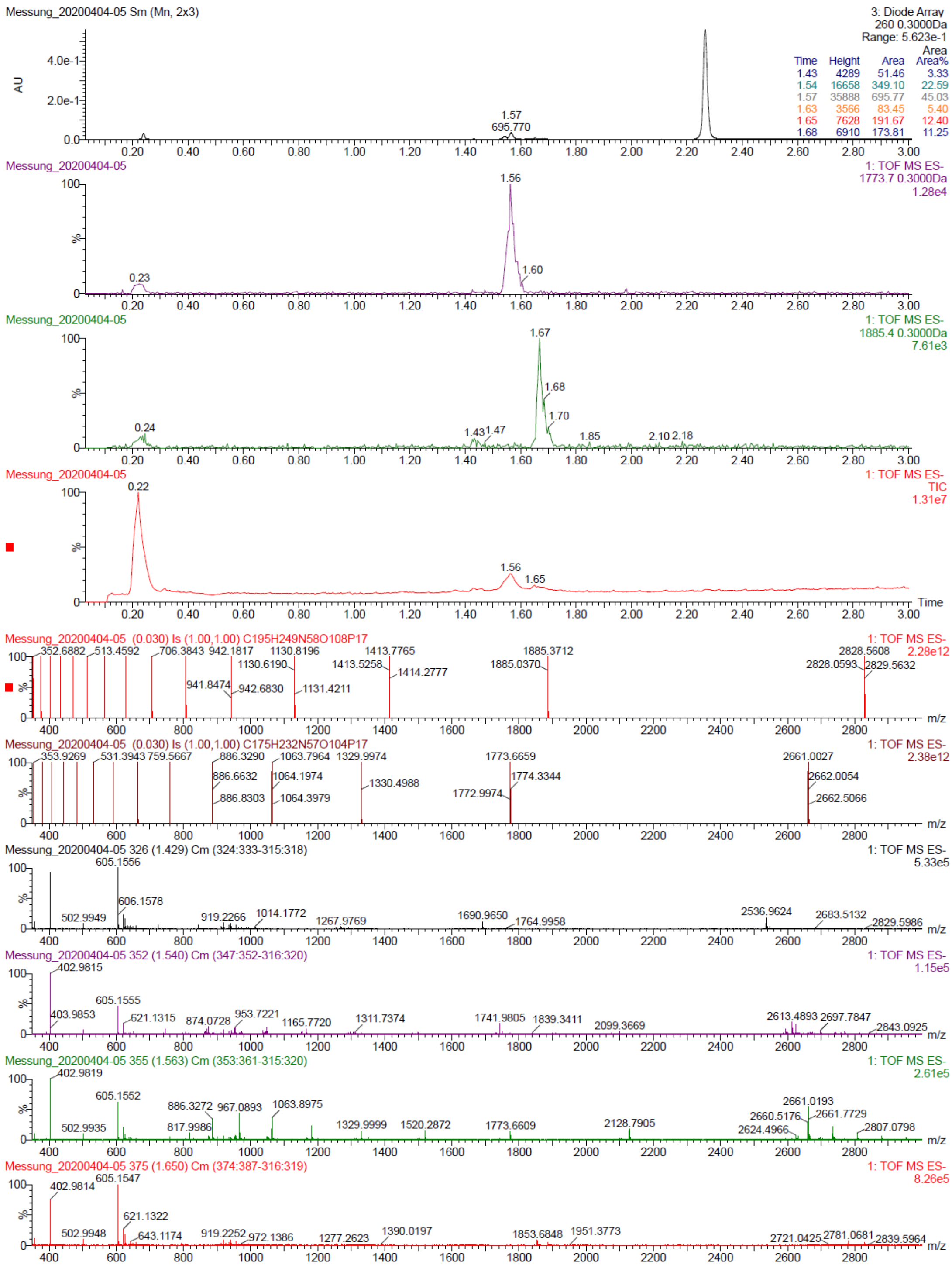

*Figure 3.79: LC-MS chromatogram of compound **Table 3 Entry 1 cyclo-triazole** reaction with Fmoc-Pra-OH*

3.4.2 [cyclo-2-Abz-Phe-Pra]-OH conjugated with HP-280 (Table 3 Entry 2)

Azido-2-Abz-Phe-Pra-OH conjugated with HP-280 (Table 3 Entry 2 Azide):

Compound **14** was coupled with **ABF 1** method (10 µl, 10 mM of **(HP-280)**, 100nmol scale) followed by **GP 1**.

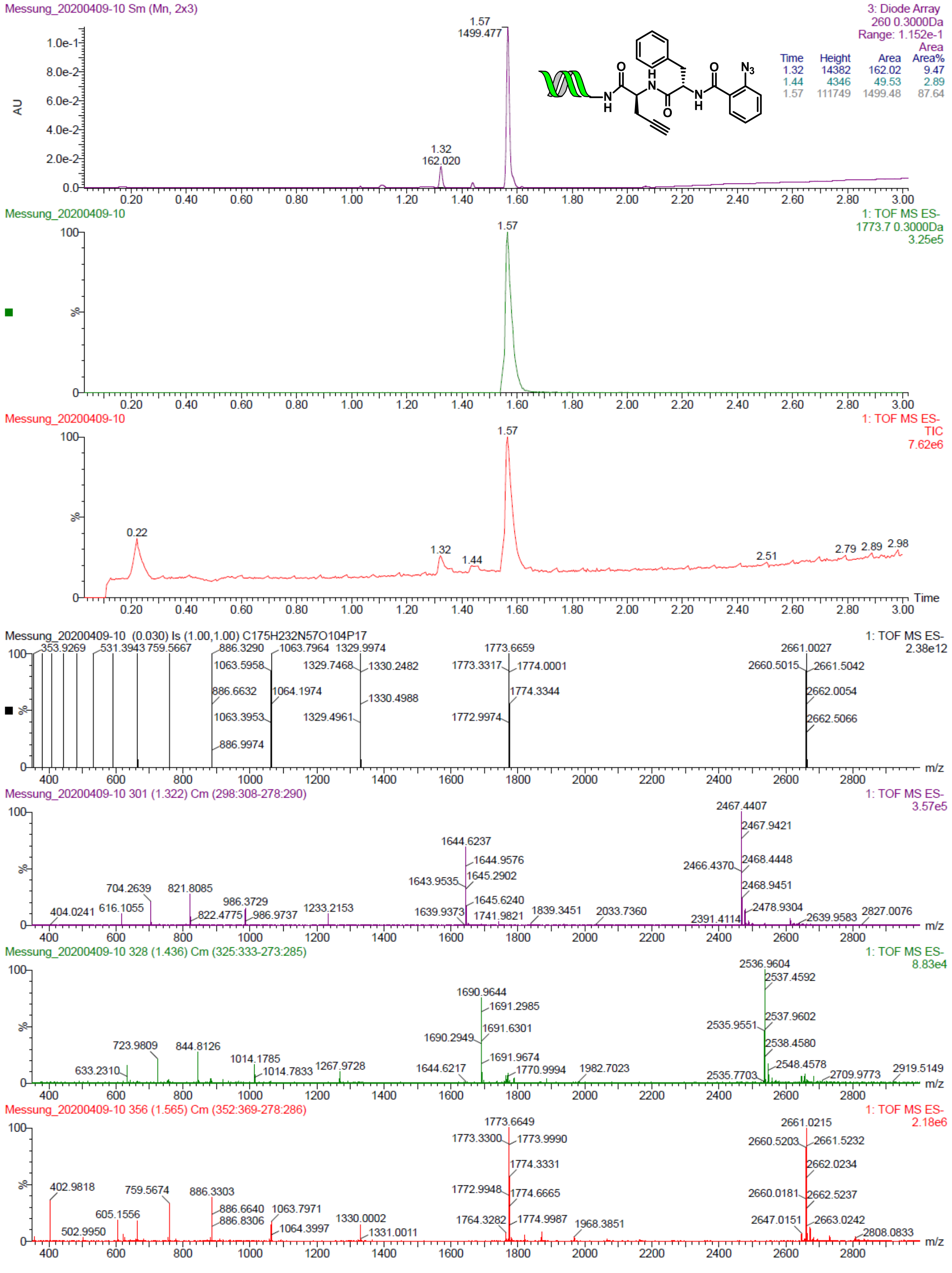

*Figure 3.80: LC-MS chromatogram of compound **Table 3 Entry 2 azide** t_R = 1,57 min TOF-MS-ESI$^-$ m/z = 1773.665(100%) $[M-3H]^{3-}$ (calc. 1773.666 for $C_{175}H_{232}N_{57}O_{104}P_{17}$)*

[cyclo-2-Abz-Phe-Pra]-OH conjugated with HP-280 (Table 3 Entry 1 Cyclo-Triazole):

The reaction was carried out using method **CuAAC 1** method (1 µl, 10 mM of **(Table 3 Entry 2 azide)**, 10nmol scale).

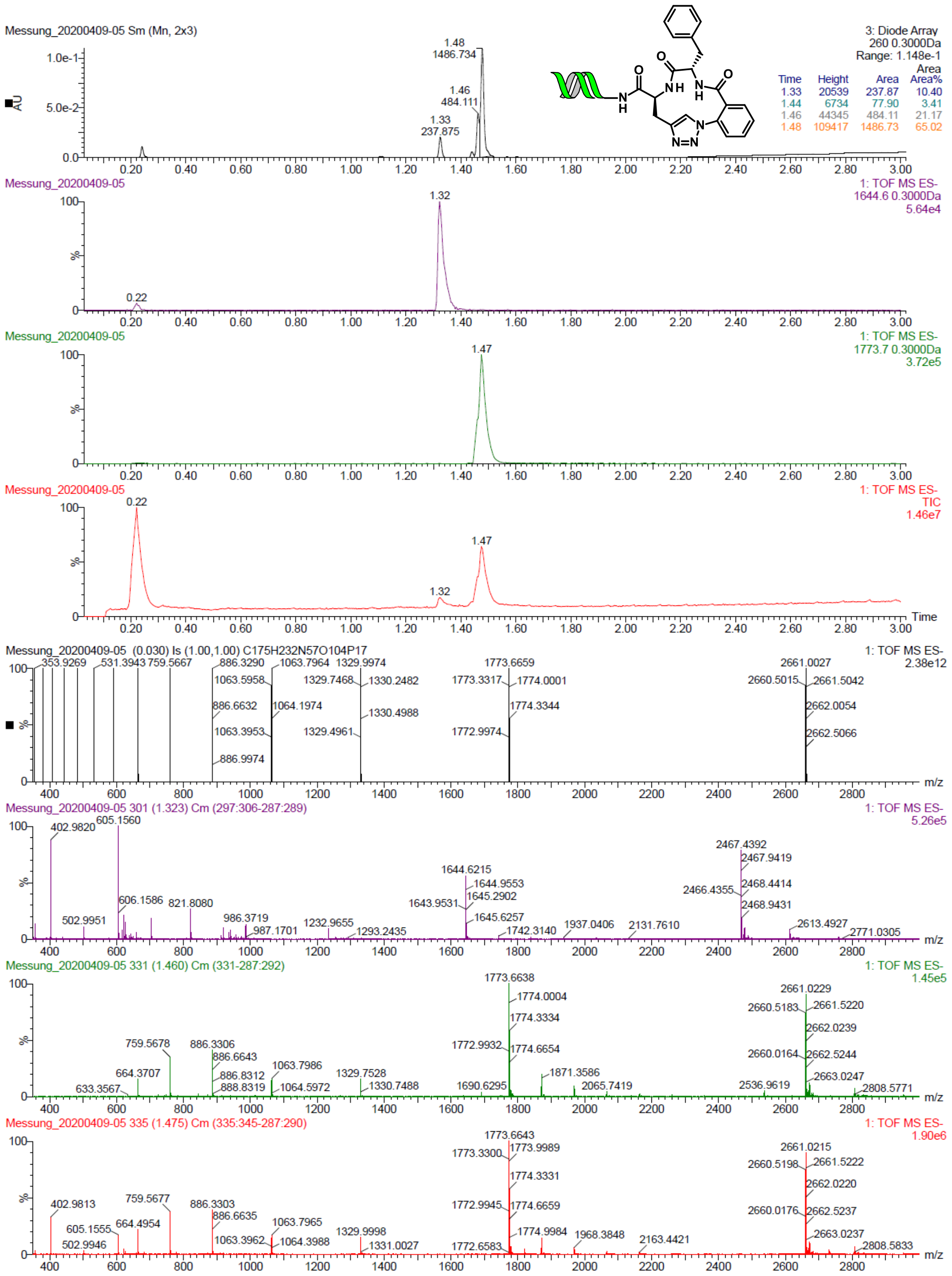

*Figure 3.81: LC-MS chromatogram of compound **Table 3 Entry 2 cyclo-triazole** t_R = 1,48 min TOF-MS-ESI$^-$ m/z = 1773.664(100%) $[M-3H]^{3-}$ (calc. 1773.666 for $C_{175}H_{232}N_{57}O_{104}P_{17}$)*

Linear Triazole of Azido-2-Abz-Phe-Pra-OH conjugated with HP-280 (Table 3 Entry 2 Linear-Triazole):

The reaction was carried out using method **CuAAC 2** (1 µl, 10 mM of **(Table 3 Entry 2 azide)**, 10nmol scale).

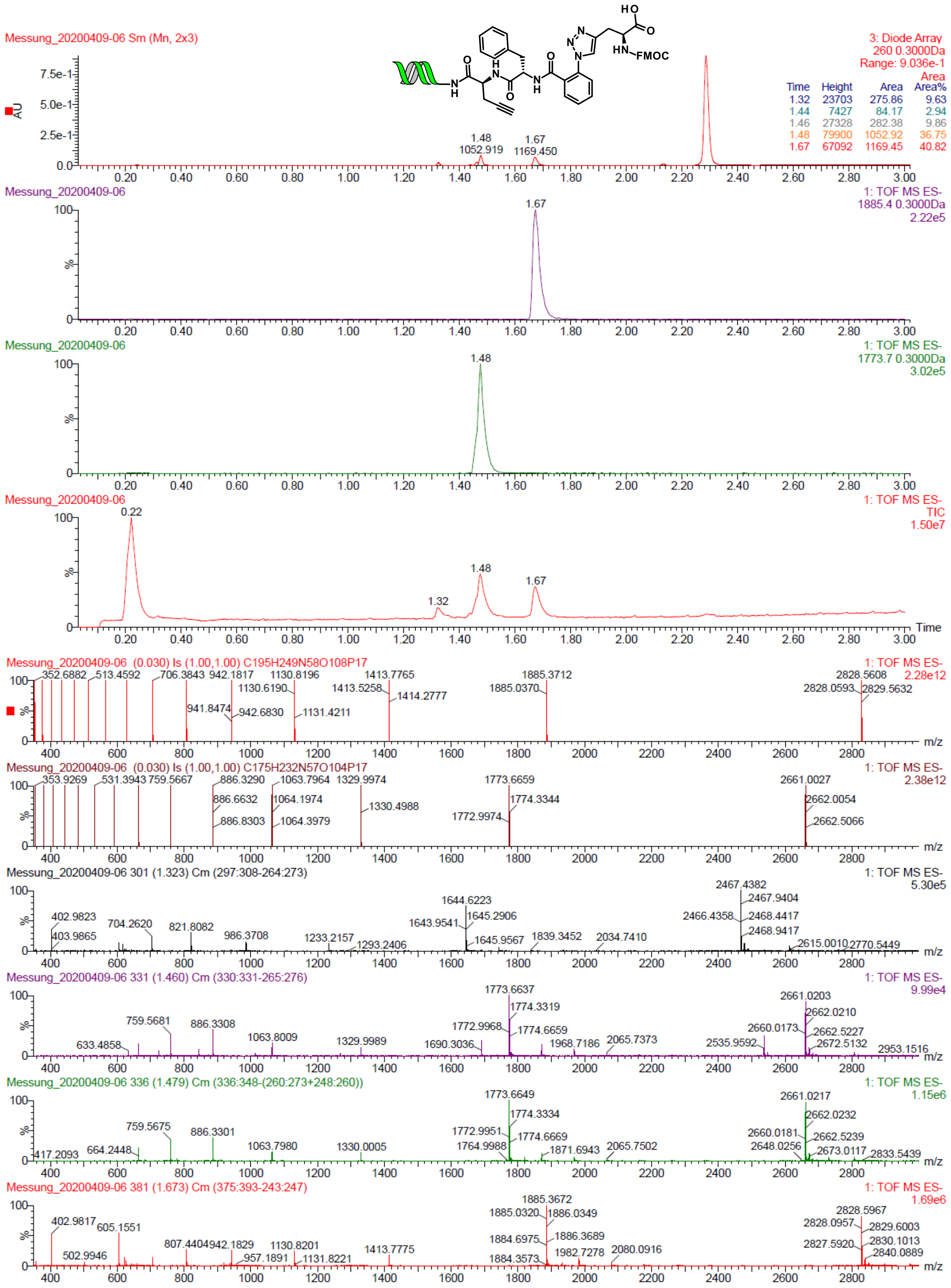

*Figure 3.82: LC-MS Chromatogram of compound **Table 3 Entry 2 linear-triazole** t_R = 1,48 min TOF-MS-ESI$^-$ m/z = 1884.367(100%) $[M-3H]^{3-}$ (calc. 1884.371 for $C_{195}H_{249}N_{58}O_{108}P_{17}$)*

3.4.3 [cyclo-β-Ala-Phe-Pra]-OH conjugated with HP-280 (Table 3 Entry 3)

H-β-Ala-Phe-Pra-OH conjugated with HP-280 (Table 3 Entry 3 Amine)

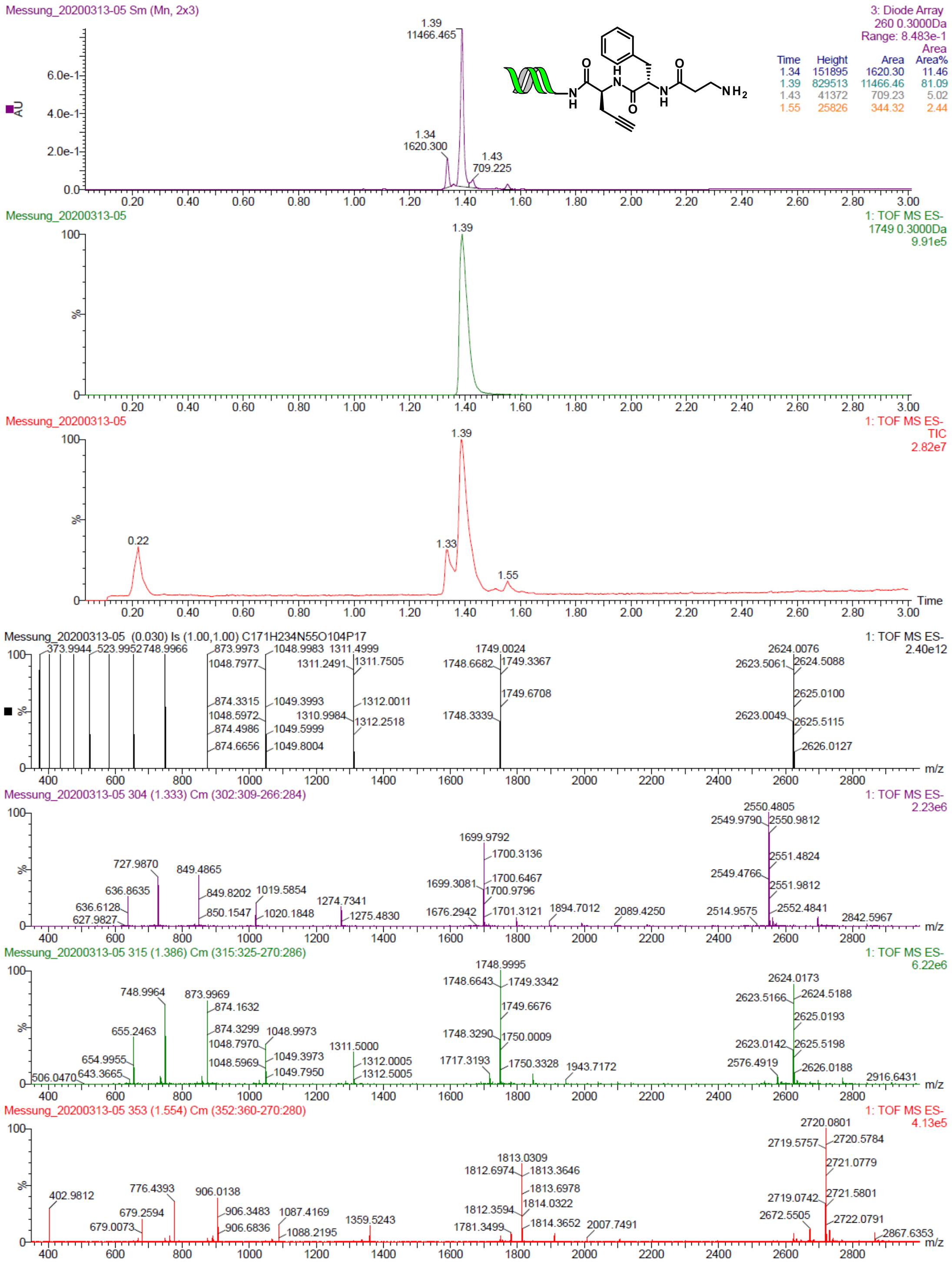

*Figure 3.83: LC-MS chromatogram of compound **Table 3 Entry 3 amine** t_R = 1,63 min TOF-MS-ESI$^-$ m/z = 1823.022(100%) [M-3H]$^{3-}$ (calc. 1823.025 for $C_{186}H_{244}N_{55}O_{106}P_{17}$)*

Azido-β-Ala-Phe-Pra-OH conjugated with HP-280 (Table 3 Entry 3 Azide):

The reaction was carried out by using the general method DTR 1 (15 µl, 10 mM of **(Table 3 Entry 3 amine)**, 150nmol scale) followed by **GP 1**.

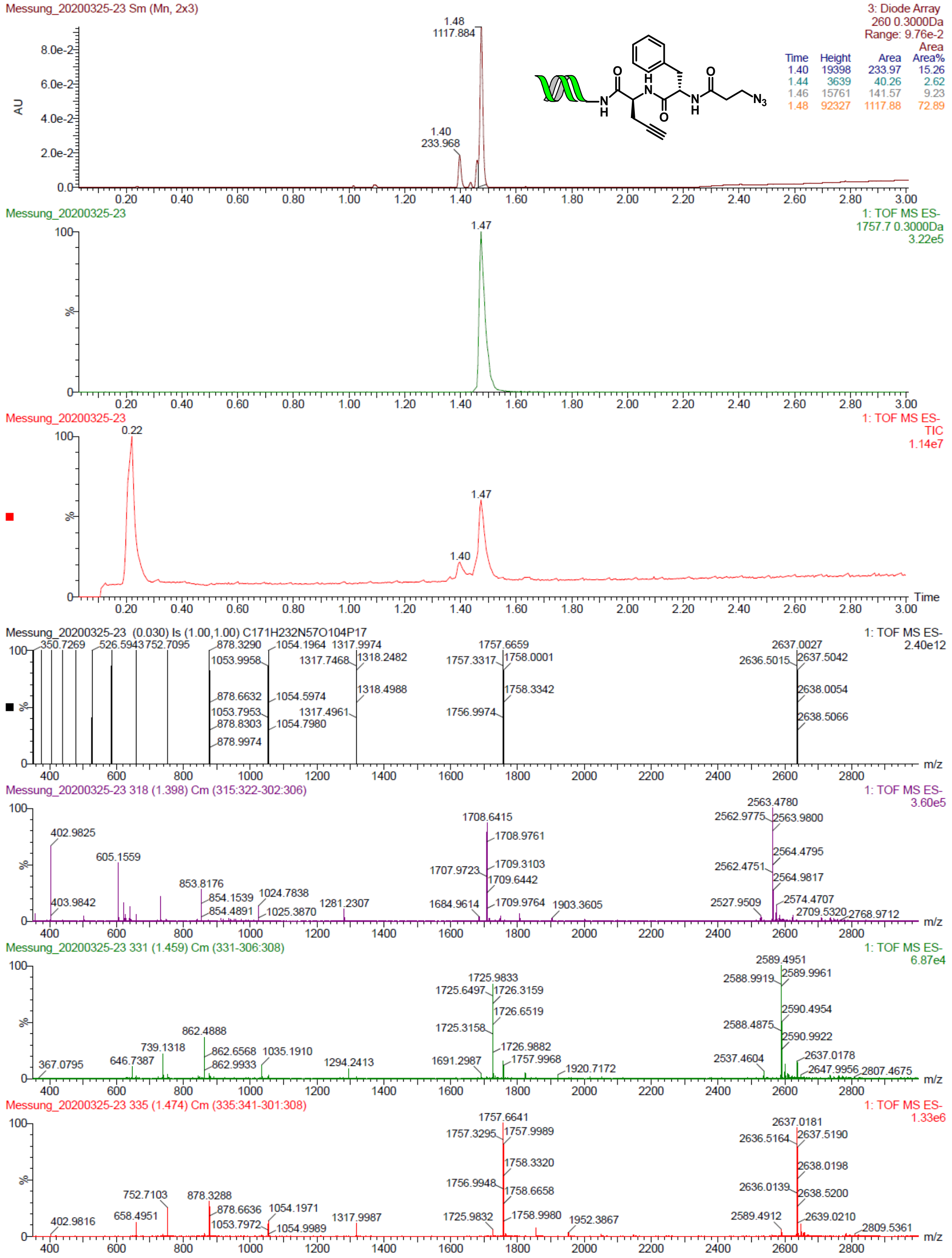

Figure 3.84: LC-MS chromatogram of compound ***Table 3 Entry 3 Azide*** *t_R = 1,48 min TOF-MS-ESI$^-$ m/z = 1757.664(100%) [M-3H]$^{3-}$ (calc. 1757.666 for $C_{171}H_{232}N_{57}O_{104}P_{17}$)*

[cyclo-β-Ala-Phe-Pra]-OH conjugated with HP-280 (Table 3 Entry 3 Cyclo-Triazole):

The reaction was carried out using method **CuAAC 1** (1 µl, 10 mM of **(Table 3 Entry 3 azide)**, 10nmol scale).

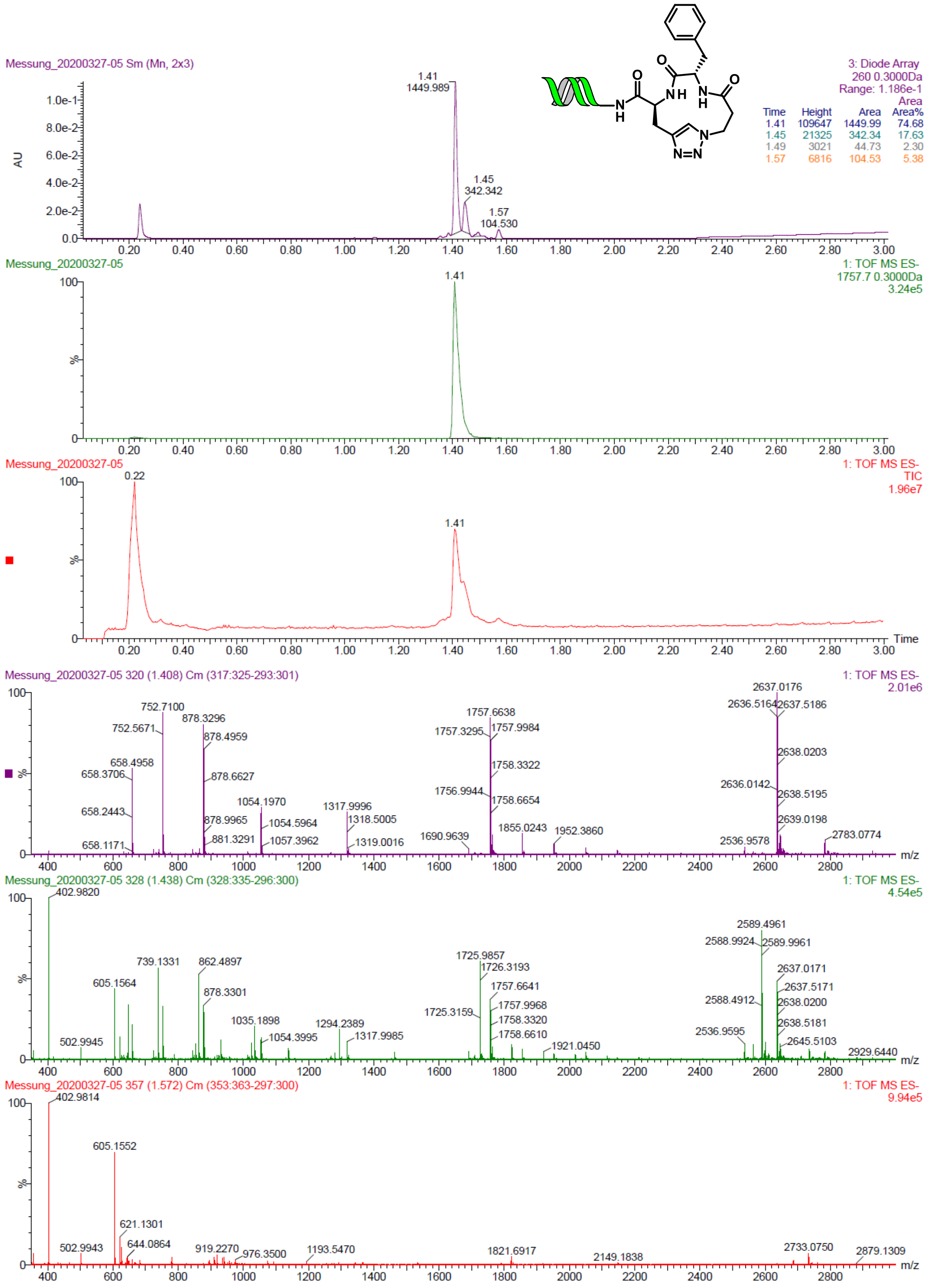

*Figure 3.85: LC-MS chromatogram of compound **Table 3 Entry 3 cyclo-triazole** t_R = 1,41 min TOF-MS-ESI$^-$ m/z = 1757.664(100%) [M-3H]$^{3-}$ (calc. 1757.666 for $C_{171}H_{232}N_{57}O_{104}P_{17}$)*

Linear triazole of Azido-β-Ala-Phe-Pra-OH conjugated with HP-280 (Table 3 Entry 3 Linear-Triazole):

The reaction was carried out using method **CuAAC 2** (1 µl, 10 mM of **(Table 3 Entry 3 azide)**, 10nmol scale).

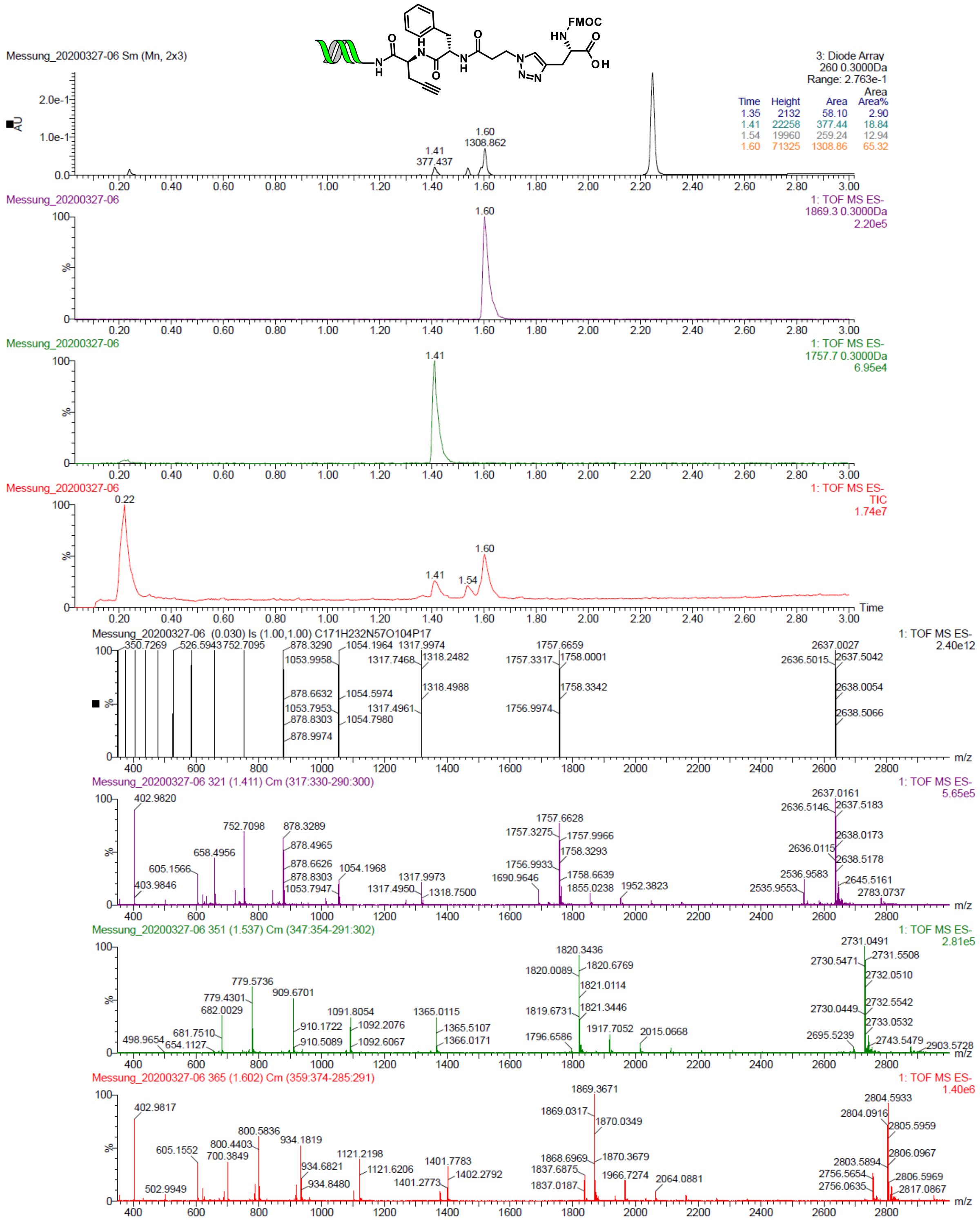

*Figure 3.86: LC-MS chromatogram of compound **Table 3 Entry 3 linear-triazole** t_R = 1,60 min TOF-MS-ESI$^-$ m/z = 1869.367(100%) $[M-3H]^{3-}$ (calc. 1869.371 for $C_{171}H_{232}N_{57}O_{104}P_{17}$)*

3.4.5 [cyclo-β-Ala-Trp-Pra]-OH conjugated with HP-280 (Table 3 Entry 4)

H-β-Ala-Trp-Pra-OH conjugated with HP-280 (Table 3 Entry 4 Amine):

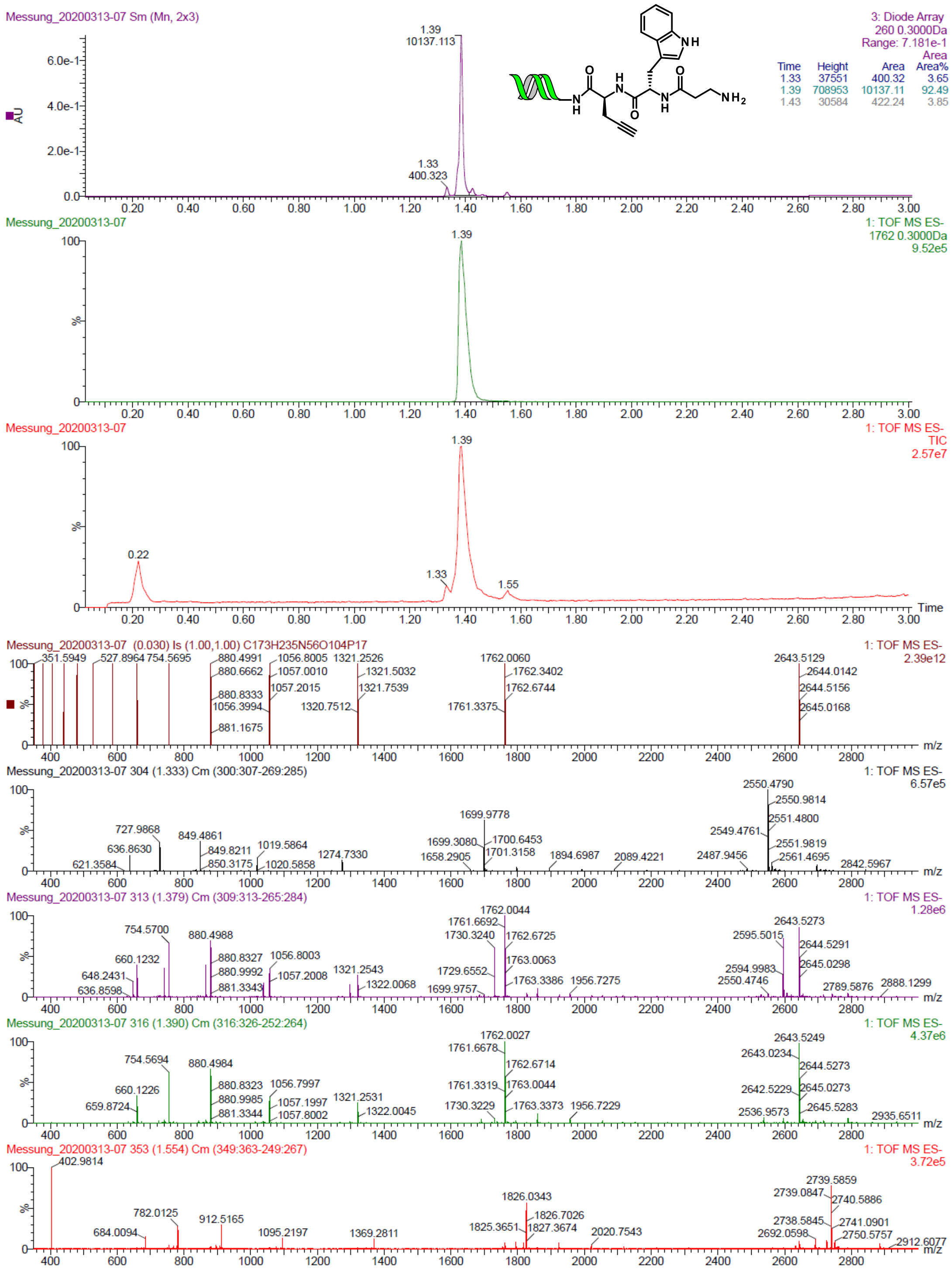

*Figure 3.87: LC-MS chromatogram of compound **Table 3 Entry 4 amine** t_R =1,39 min TOF-MS-ESI$^-$ m/z = 1762.003(100%) $[M-3H]^{3-}$ (calc. 1762.006 for $C_{173}H_{235}N_{56}O_{104}P_{17}$)*

Azido-β-Ala-Trp-Pra-OH conjugated with HP-280 (Table 3 Entry 4 Azide):

The reaction was carried out by using the general method DTR 1 (15 µl, 10 mM of **(Table 3 Entry 4 amine)**, 150nmol scale) followed by general method **GP 1**.

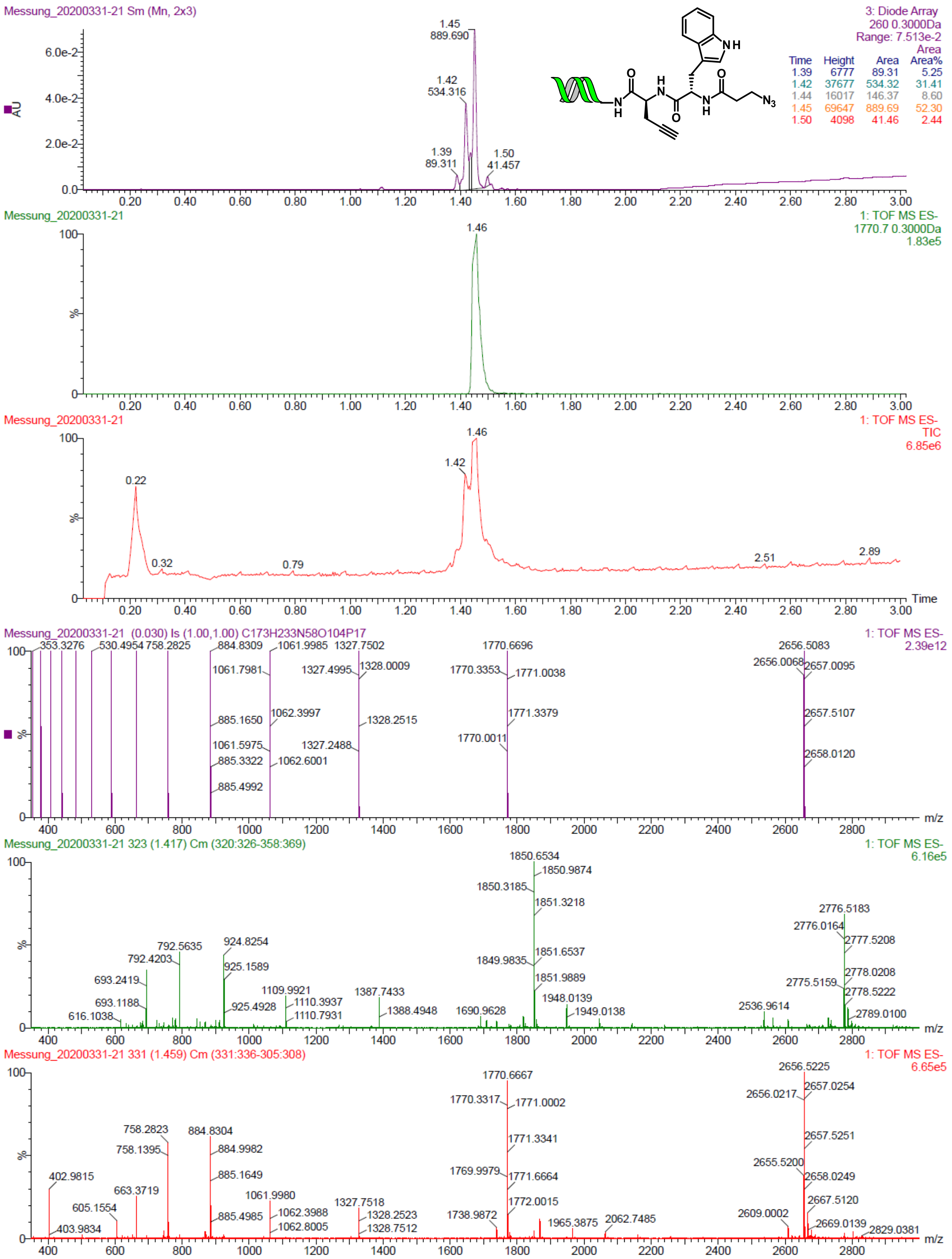

*Figure 3.88: LC-MS chromatogram of compound **Table 3 Entry 4 azide** t_R = 1,48 min TOF-MS-ESI⁻ m/z = 1770.667(100%) [M-3H]$^{3-}$ (calc. 1770.670 for $C_{173}H_{233}N_{58}O_{104}P_{17}$)*

[cyclo-β-Ala-Trp-Pra]-OH conjugated with HP-280 (Table 3 Entry 4 Cyclo-Triazole):

The reaction was carried out using method **CuAAC 1** (1 µl, 10 mM of **(Table 3 Entry 4 azide)**, 10nmol scale).

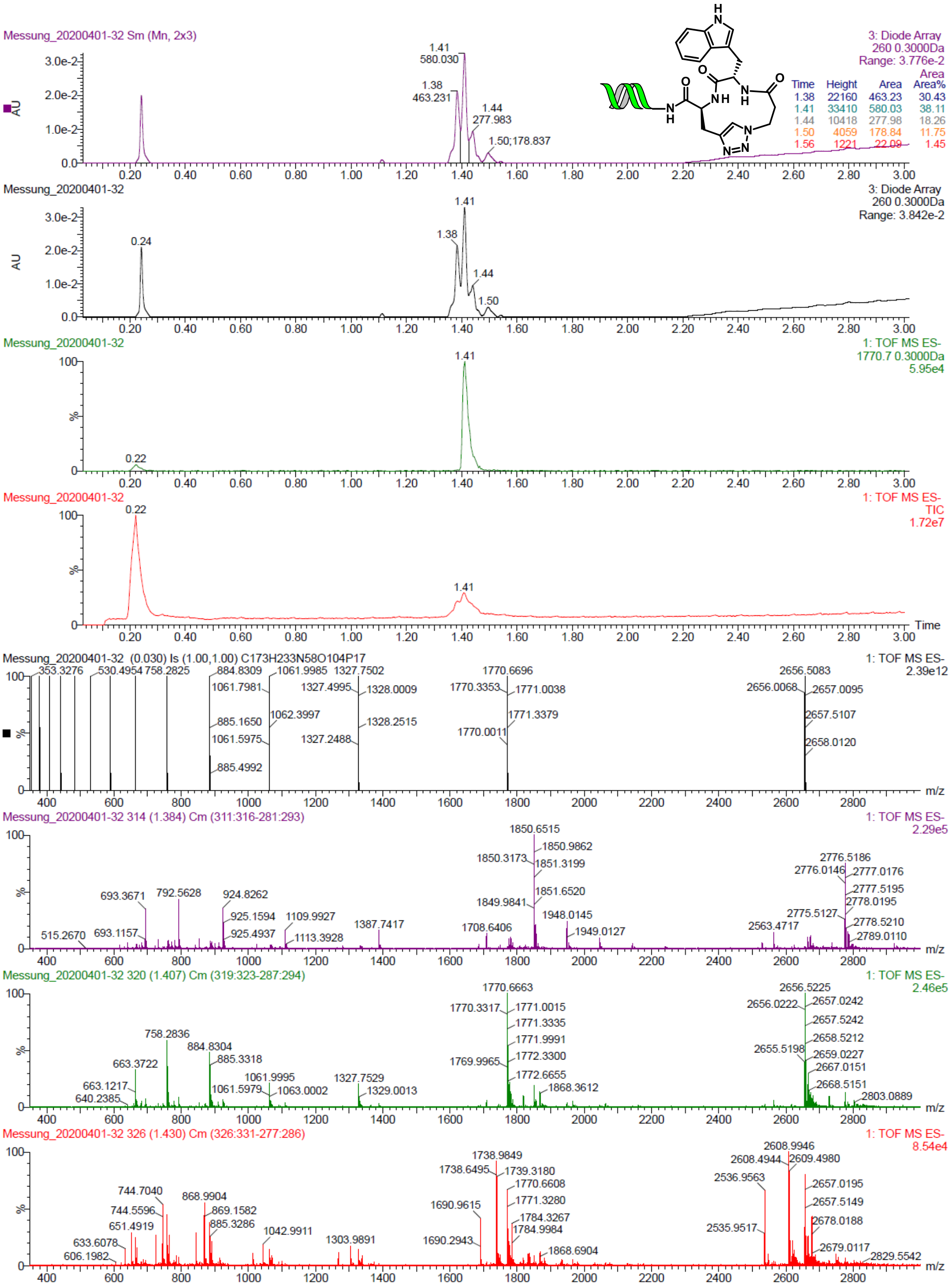

*Figure 3.89: LC-MS chromatogram of compound **Table 3 Entry 4 cyclo-triazole** t_R = 1,48 min TOF-MS-ESI$^-$ m/z = 1770.666(100%) [M-3H]$^{3-}$ (calc. 1770.670 for $C_{173}H_{233}N_{58}O_{104}P_{17}$)*

Linear Triazole of Azido-β-Ala-Trp-Pra-OH conjugated with HP-280 (Table 3 Entry 4 Linear-Triazole):

The reaction was carried out using method **CuAAC 2** (1 µl, 10 mM of **(Table 3 Entry 4 azide)**, 10nmol scale).

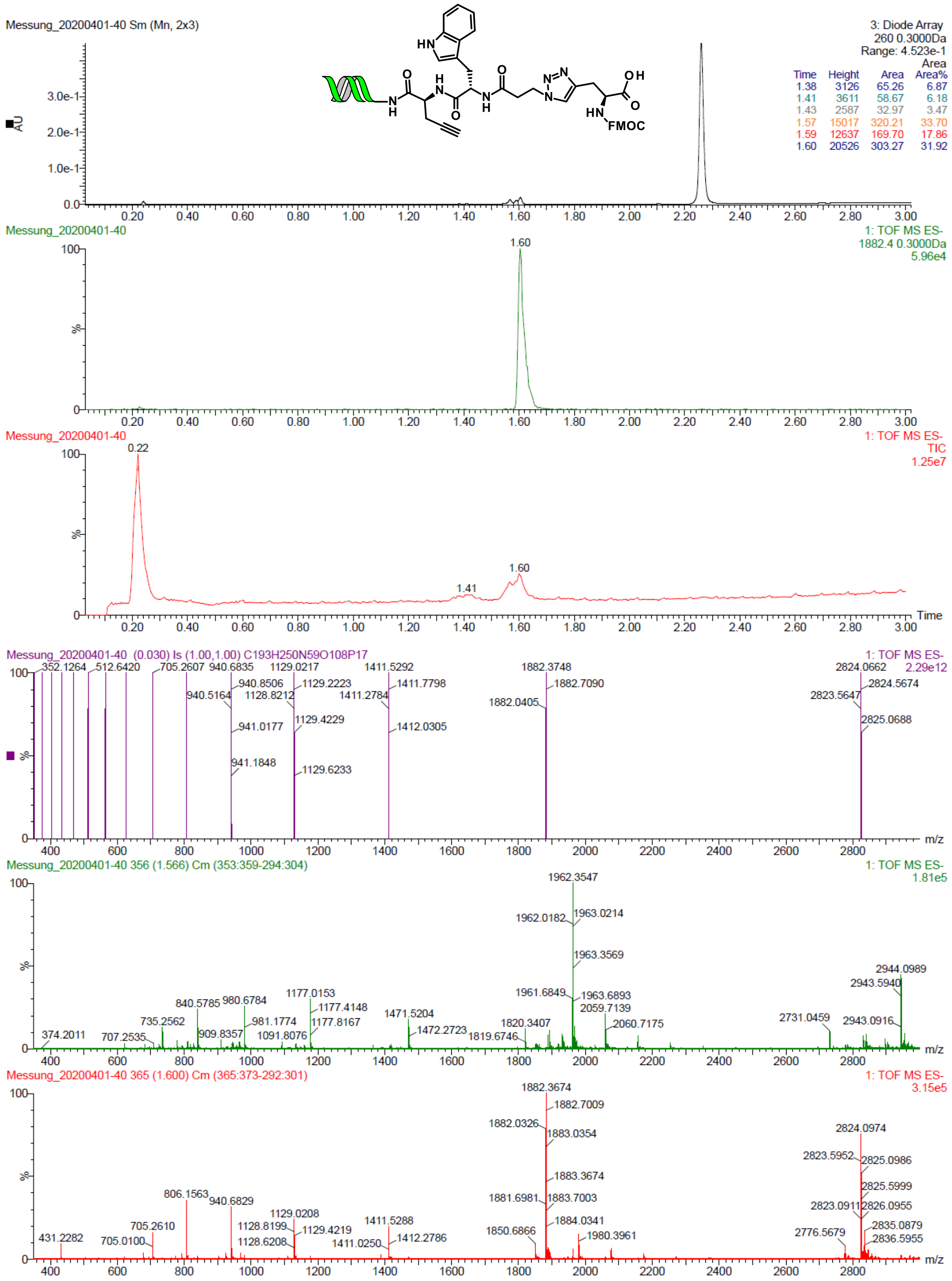

*Figure 3.90: LC-MS chromatogram of compound **Table 3 Entry 4 linear-triazole** t_R = 1,48 min TOF-MS-ESI$^-$ m/z = 1882.367(100%) $[M-3H]^{3-}$(calc. 1882.375 for $C_{193}H_{250}N_{59}O_{108}P_{17}$)*

3.4.6 [cyclo-Gly-Trp-Pra]-OH conjugated with HP-280 (Table 3 Entry 5)

H-Gly-Trp-Pra-OH conjugated with HP-280 (Table 3 Entry 5 Amine):

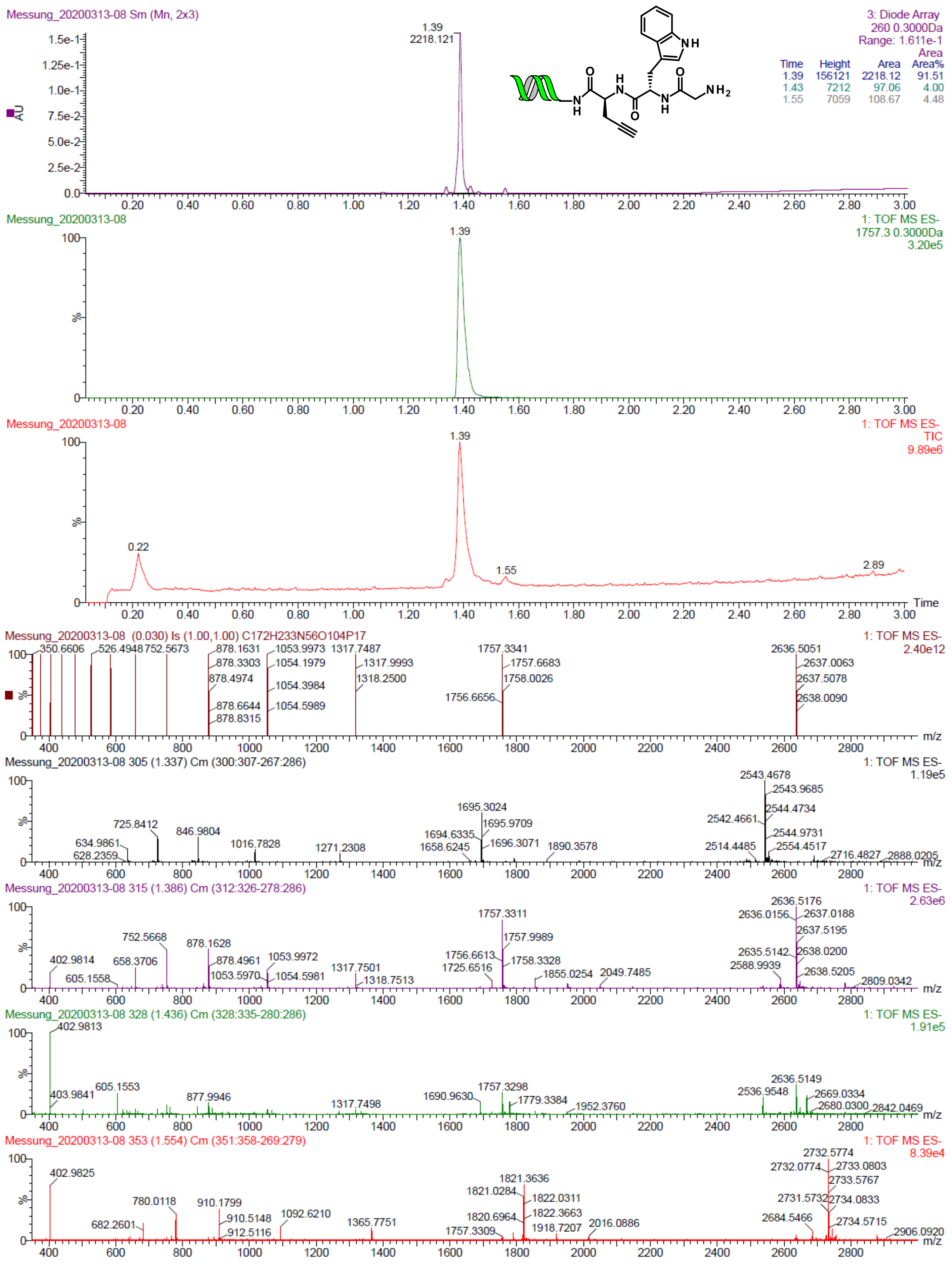

*Figure 3.91: LC-MS chromatogram of compound **Table 3 Entry 5 amine** t_R = 1,39 min TOF-MS-ESI$^-$ m/z = 1757.331(100%) $[M-3H]^{3-}$ (calc. 1757.334 for $C_{172}H_{233}N_{56}O_{104}P_{17}$)*

Azido-Gly-Trp-Pra-OH conjugated with HP-280 (Table 3 Entry 5 Azide):

The reaction was carried out by using the general method DTR 1 (15 µl, 10 mM of **(Table 3 Entry 5 amine)**, 150nmol scale) followed by general method **GP 1**.

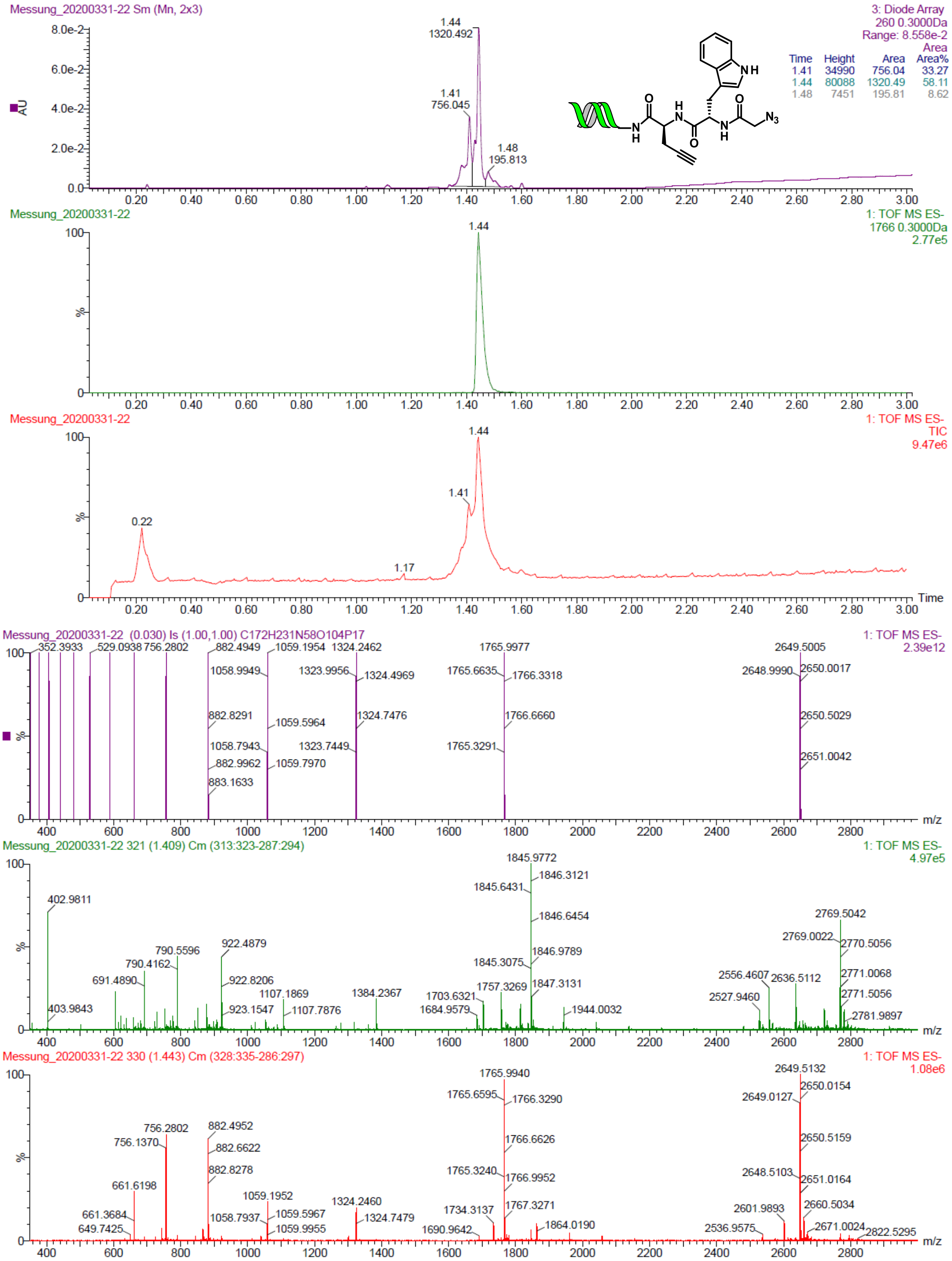

*Figure 3.92: LC-MS chromatogram of compound **Table 3 Entry 5 azide** t_R = 1,44 min TOF-MS-ESI$^-$ m/z = 1765.994(100%) $[M-3H]^{3-}$ (calc. 1765.998 for $C_{171}H_{231}N_{58}O_{104}P_{17}$)*

[cyclo-Gly-Trp-Pra]-OH conjugated with HP-280 (Table 3 Entry 5 Cyclo-Triazole):

The reaction was carried out using method **CuAAC 1** (1 µl, 10 mM of **(Table 3 Entry 5 azide)**, 10nmol scale).

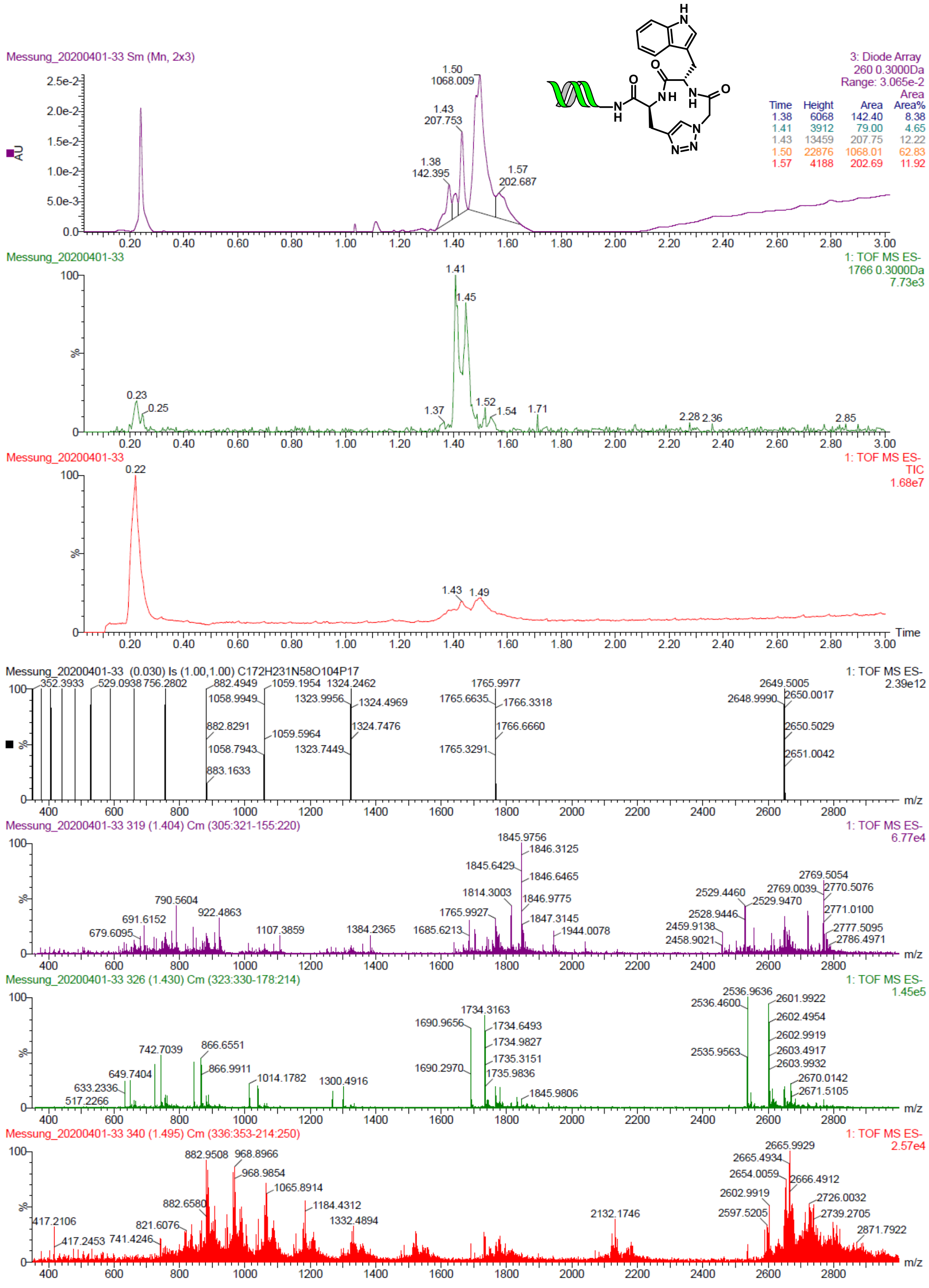

Figure 3.93: LC-MS chromatogram of compound ***Table 3 Entry 5 cyclo-triazole,*** *TOF-MS-ESI*$^-$ *m/z = 1884.367(100%) [M-3H]*$^{3-}$ *(calc. 1884.371 for* $C_{171}H_{232}N_{57}O_{104}P_{17}$*)*

Linear Triazole of Azido-Gly-Trp-Pra-OH conjugated with HP-280 (Table 3 Entry 5 Linear-Triazole):

The reaction was carried out using method **CuAAC 2** (1 µl, 10 mM of **(Table 3 Entry 5 azide)**, 10nmol scale).

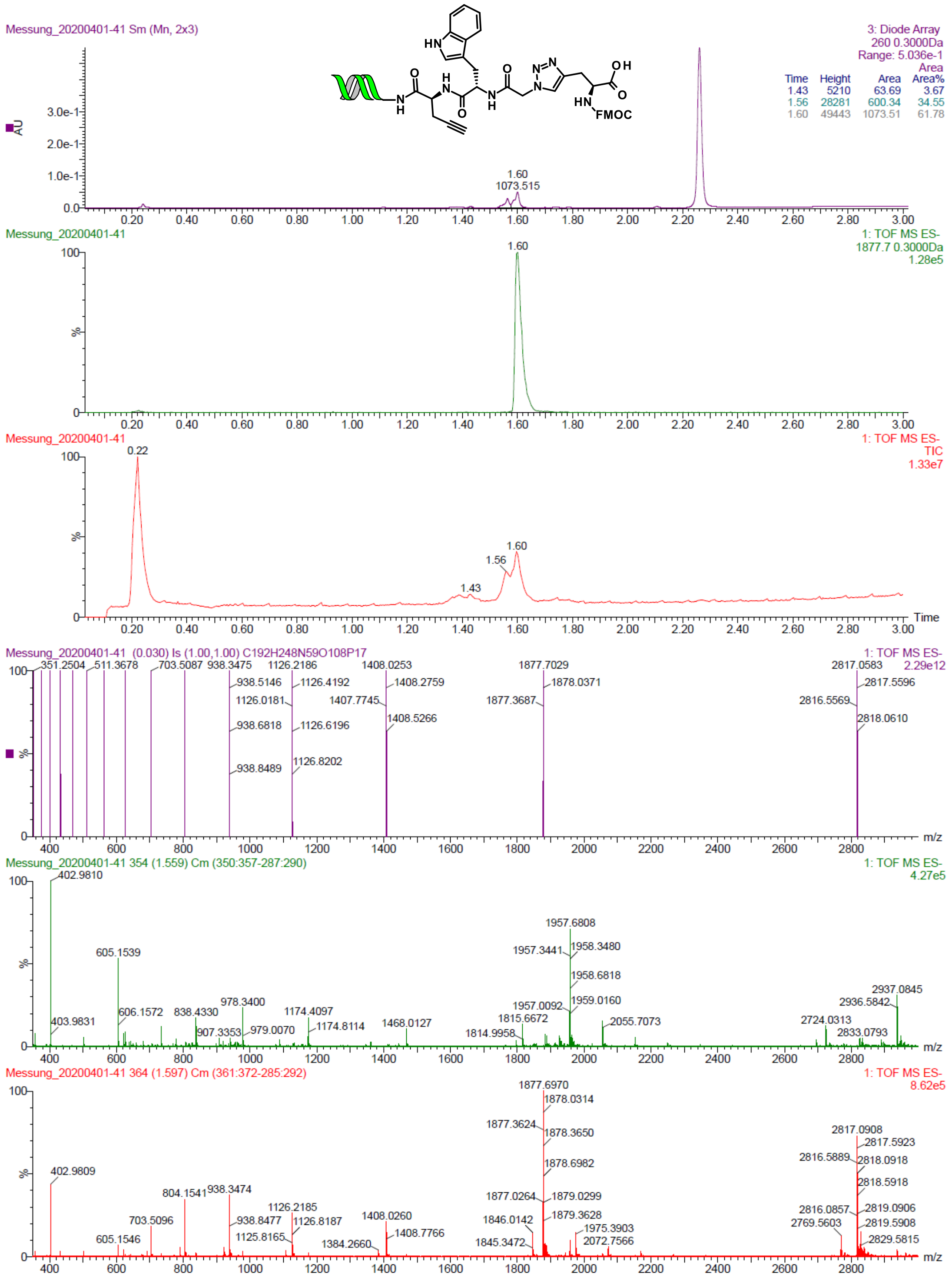

*Figure 3.94: LC-MS chromatogram of compound **Table 3 Entry 5 linear triazole** t_R = 1,48 min TOF-MS-ESI$^-$ m/z = 1877.697(100%) $[M-3H]^{3-}$ (calc. 1877.703 for $C_{192}H_{248}N_{59}O_{108}P_{17}$)*

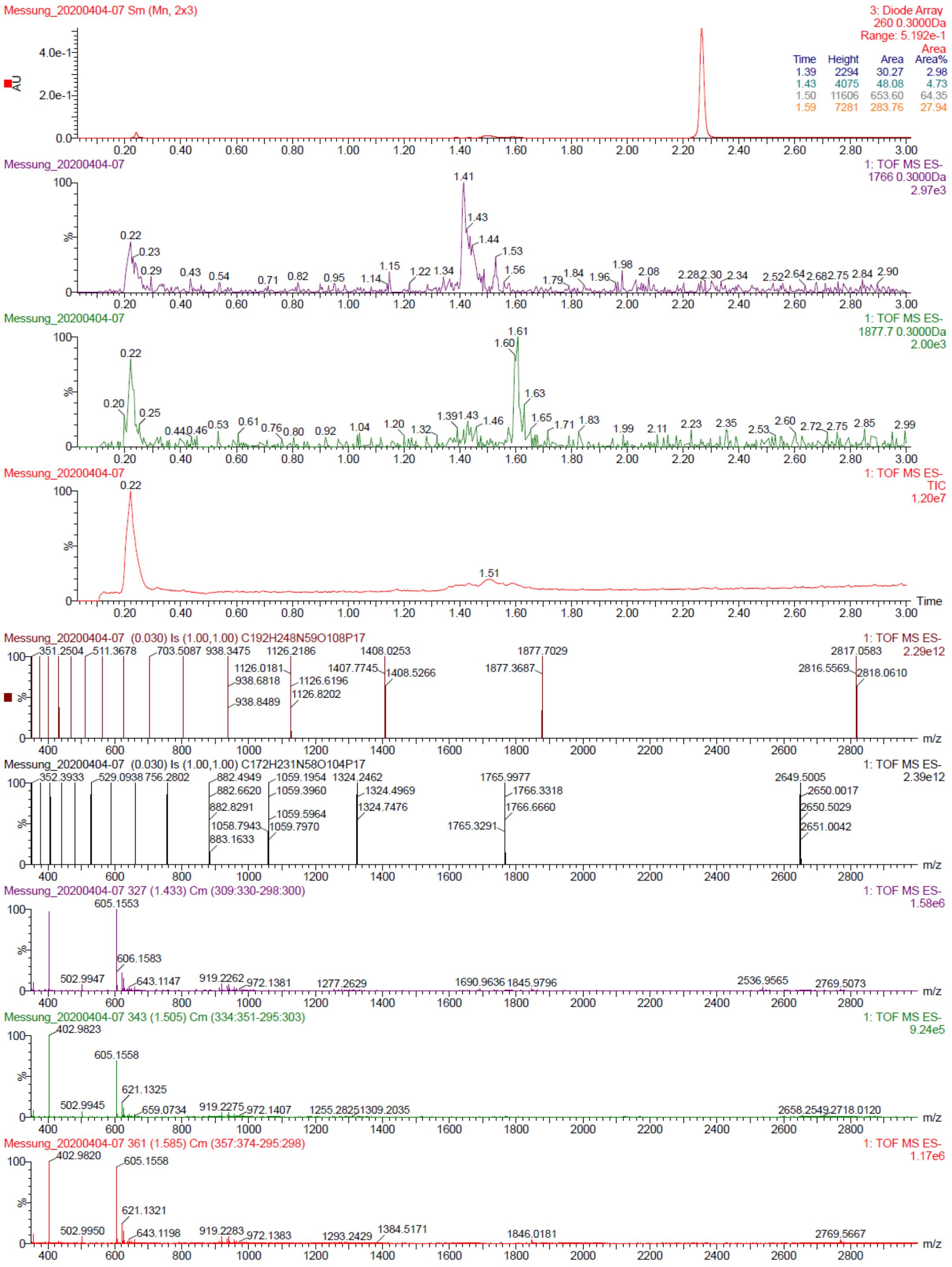

*Figure 3.95: LC-MS chromatogram of compound **Table 3 Entry 5 cyclo-triazole** reaction with Fmoc-Pra-OH*

3.4.7 [cyclo-γ-Abu-Trp-Pra]-OH conjugated with HP-280 (Table 3 Entry 6)

H-γ-Abu-Trp-Pra-OH conjugated with HP-280 (Table 3 Entry 6 Amine):

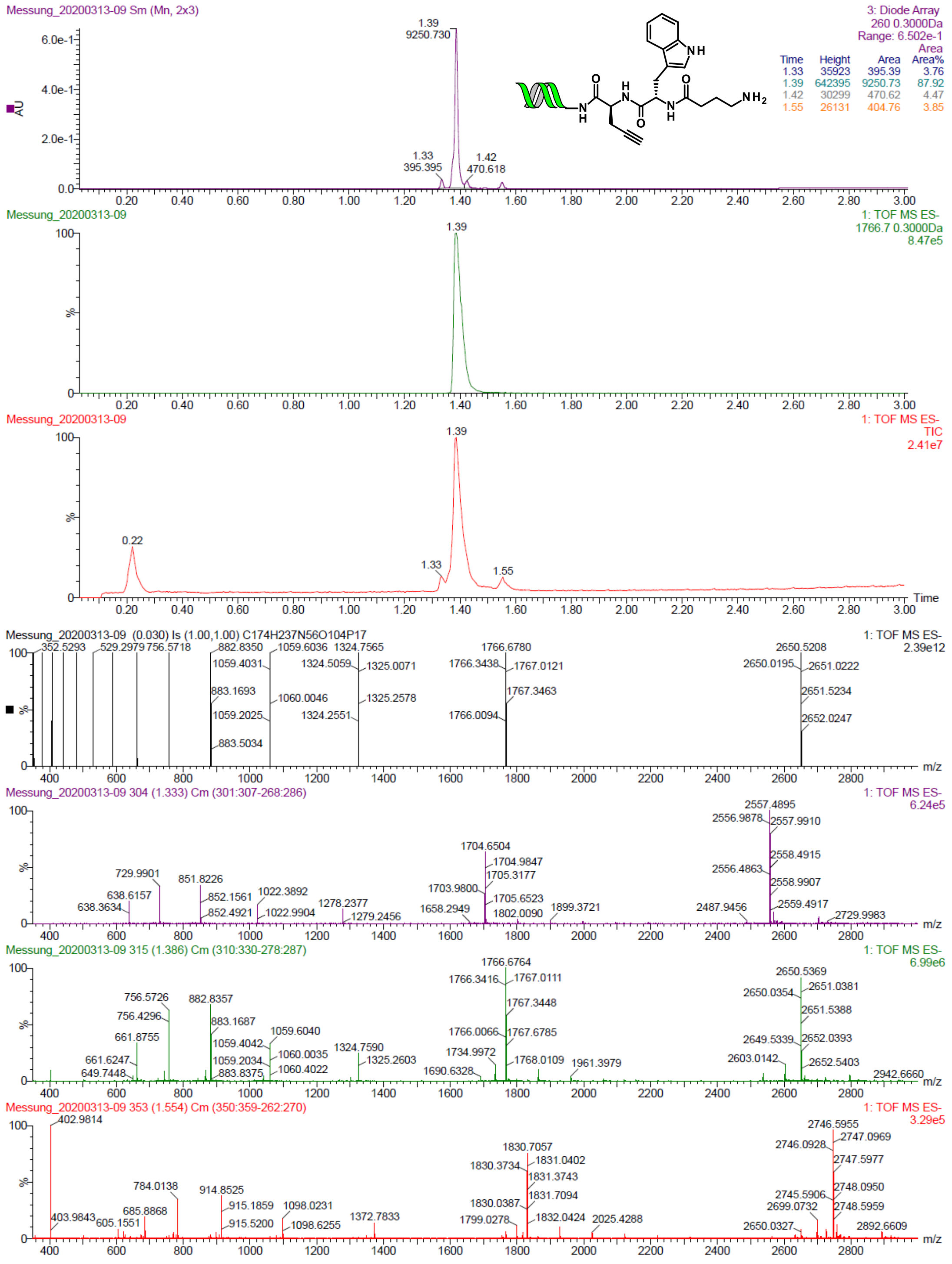

*Figure 3.96: LC-MS chromatogram of compound **Table 3 Entry 6 amine** t_R = 1,39 min TOF-MS-ESI$^-$ m/z = 1766.676(100%) [M-3H]$^{3-}$ (calc. 1766.678 for $C_{174}H_{237}N_{56}O_{104}P_{17}$)*

Azido-γ-Abu-Trp-Pra-OH conjugated with HP-280 (Table 3 Entry 6 Azide):

The reaction was carried out by using the general method DTR 1 (15 µl, 10 mM of **(Table 3 Entry 6 amine)**, 150nmol scale) followed by general method **GP 1**.

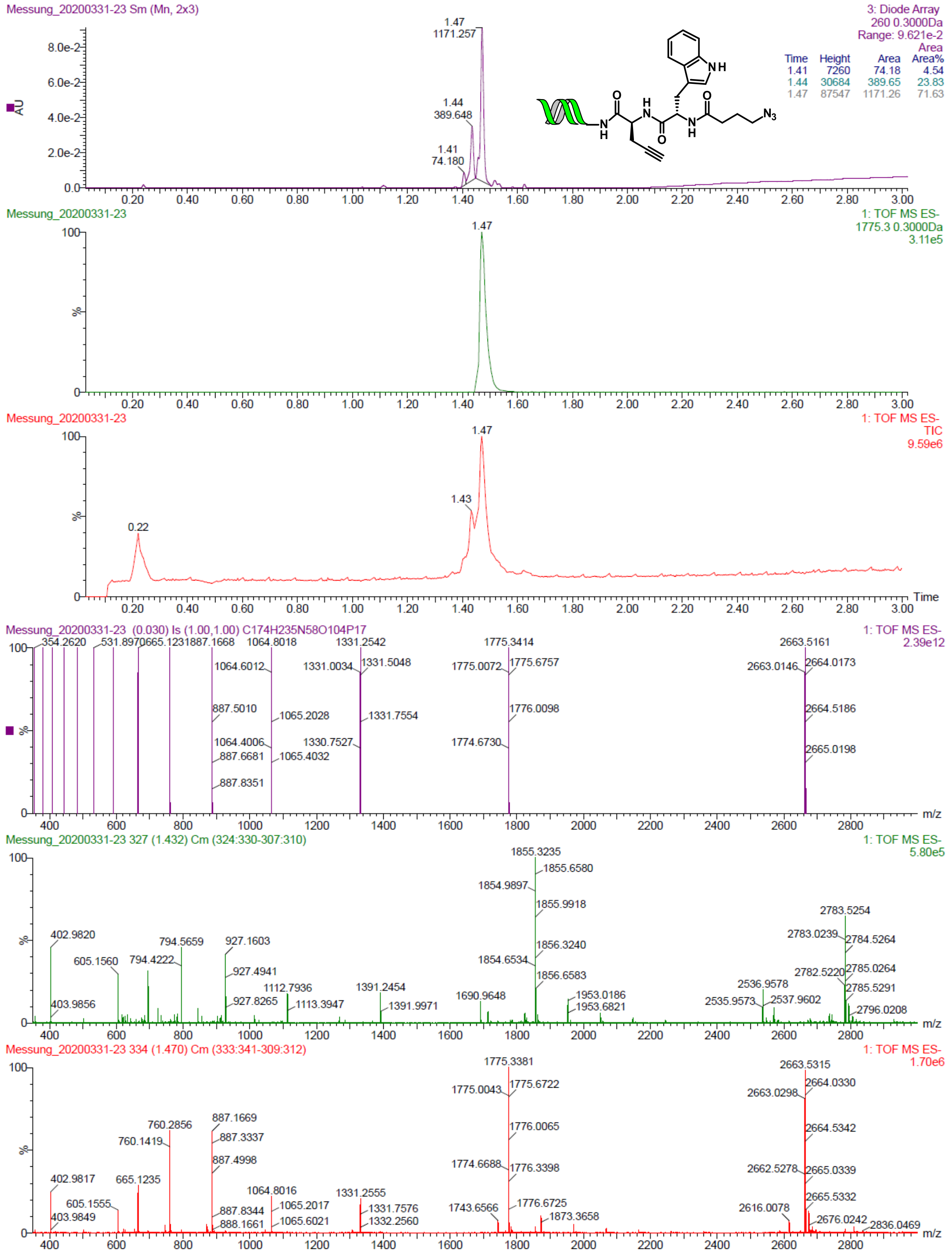

*Figure 3.97: LC-MS chromatogram of compound **Table 3 Entry 6 azide** t_R = 1,47 min TOF-MS-ESI$^-$ m/z = 1775.338(100%) [M-3H]$^{3-}$ (calc. 1775.341 for $C_{174}H_{235}N_{58}O_{104}P_{17}$)*

[cyclo-γ-Abu-Trp-Pra]-OH conjugated with HP-280 (Table 3 Entry 6 Cyclo-Triazole):

The reaction was carried out using method **CuAAC 1** (1 µl, 10 mM of **(Table 3 Entry 6 azide)**, 10nmol scale).

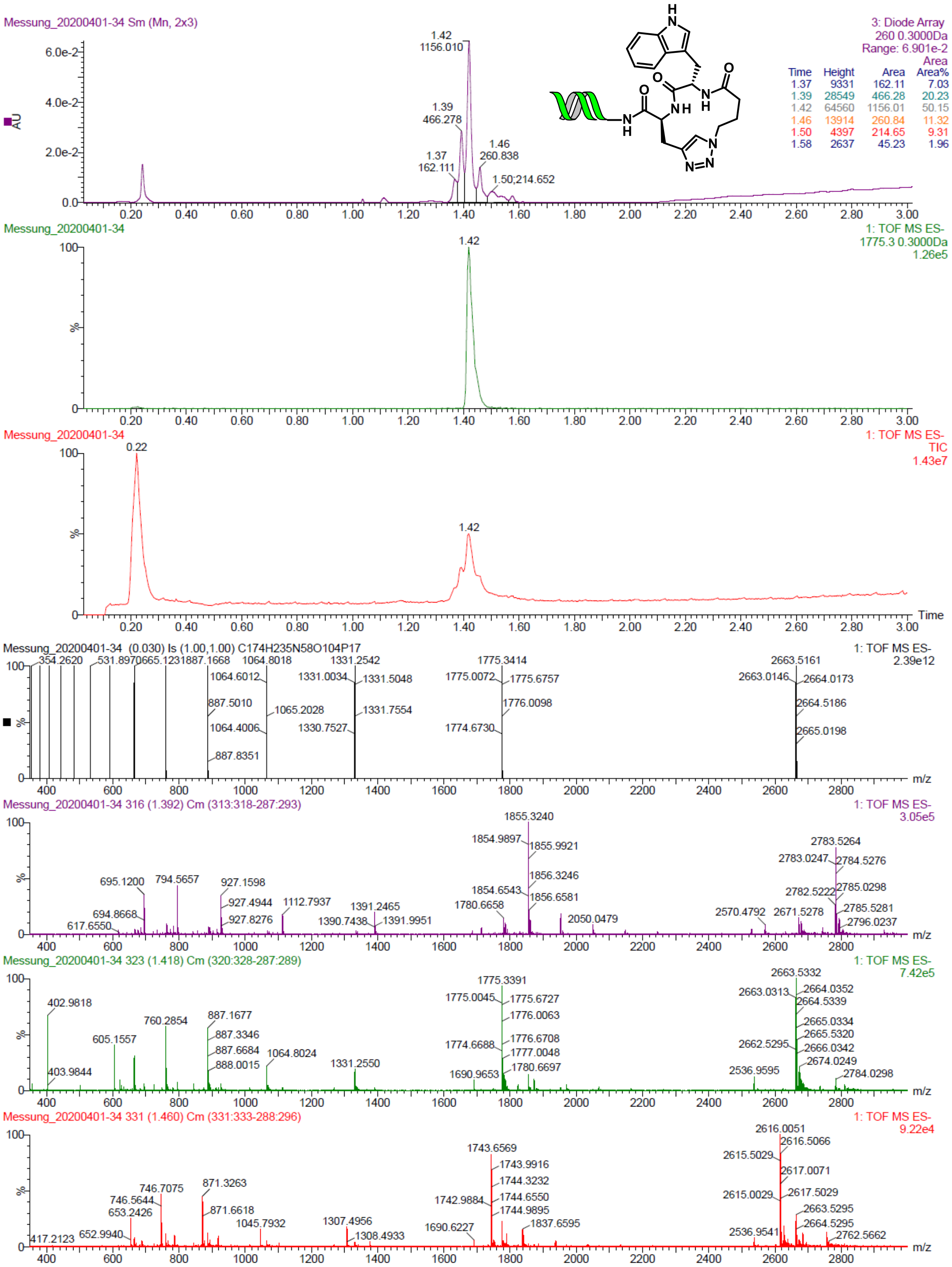

*Figure 3.98: LC-MS chromatogram of compound **Table 3 Entry 6 cyclo-triazole** t_R = 1,42 min TOF-MS-ESI$^-$ m/z = 1775.339(100%) $[M-3H]^{3-}$ (calc. 1775.341 for $C_{174}H_{235}N_{58}O_{104}P_{17}$)*

Linear Triazole of Azido-γ-Abu-Trp-Pra-OH conjugated with HP-280 (Table 3 Entry 6 Linear-Triazole):

The reaction was carried out using method **CuAAC 2** (1 µl, 10 mM of **(Table 3 Entry 6 azide)**, 10nmol scale).

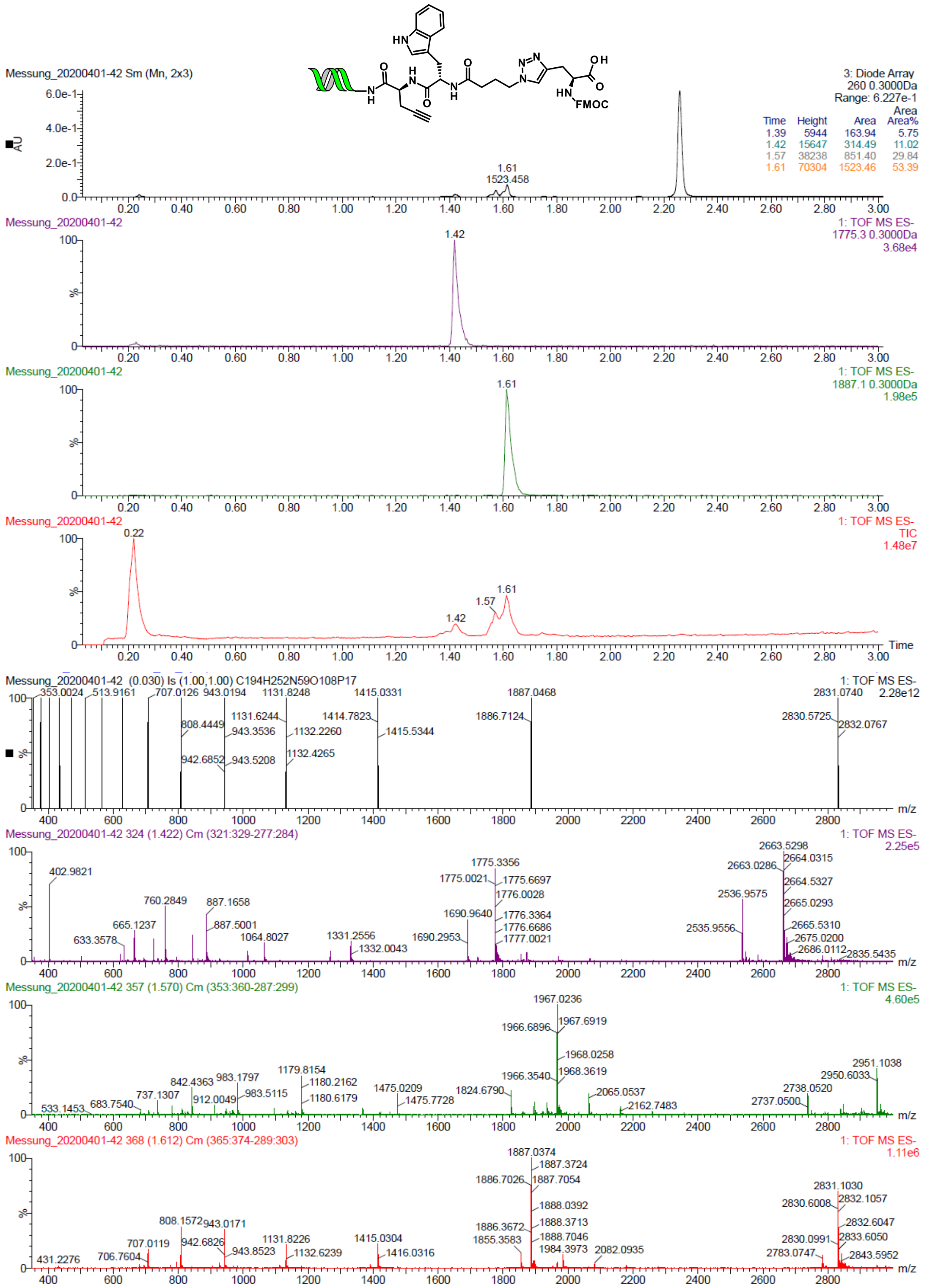

*Figure 3.99: LC-MS chromatogram of compound **Table 3 Entry 6 linear-triazole** t_R = 1,61 min TOF-MS-ESI$^-$ m/z = 1887.037(100%) [M-3H]$^{3-}$ (calc. 1887.047 for $C_{199}H_{252}N_{59}O_{108}P_{17}$)*

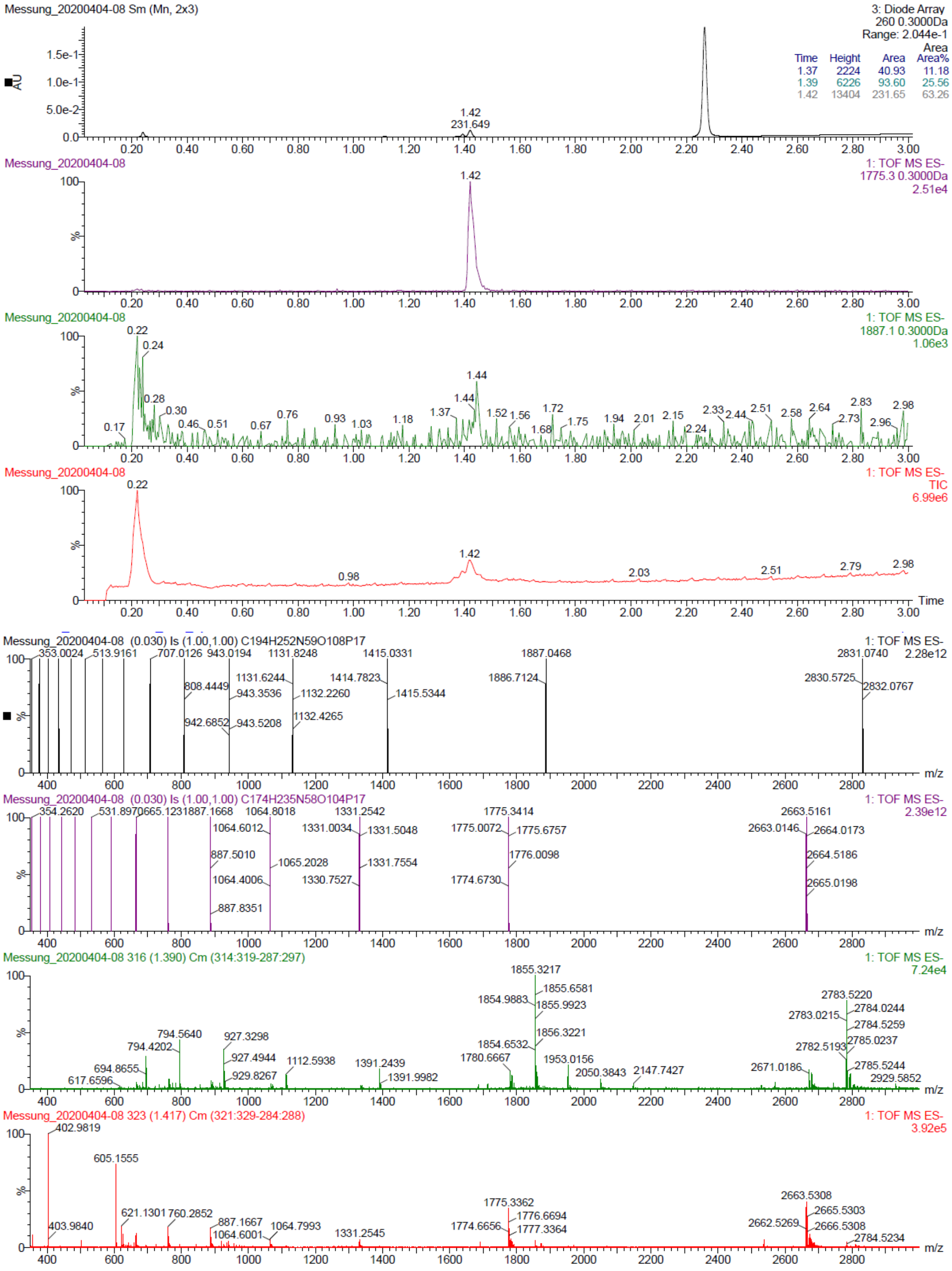

Figure 3.100: LC-MS chromatogram of compound ***Table 3 Entry 6 cyclo-triazole*** *reaction with Fmoc-Pra-OH*

3.4.8 [cyclo-βAla-βAla-Pra]-OH conjugated with HP-280 (Table 3 Entry 7)

H-β-Ala-β-Ala-Pra-OH conjugated with HP-280 (Table 3 Entry 7 Amine):

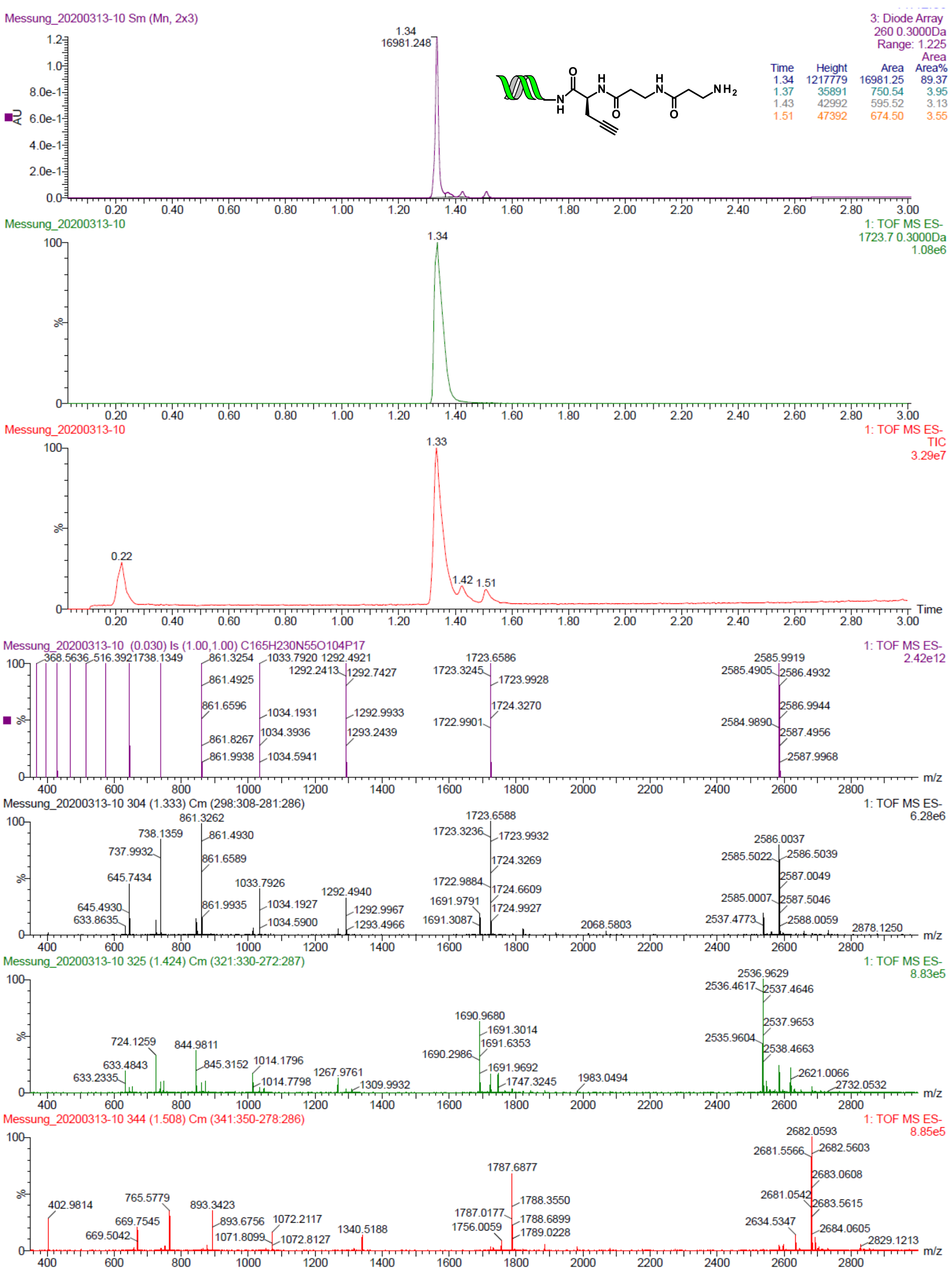

*Figure 3.101: LC-MS chromatogram of compound **Table 3 Entry 7 amine** t_R = 1,34 min TOF-MS-ESI$^-$ m/z = 1723.659(100%) $[M-3H]^{3-}$ (calc. 1723.659 for $C_{165}H_{230}N_{55}O_{104}P_{17}$)*

Azido-β-Ala-β-Ala-Pra-OH conjugated with HP-280 (Table 3 Entry 7 Azide):

The reaction was carried out by using the general method DTR 1 (15 µl, 10 mM of **(Table 3 Entry 7 amine)**, 150nmol scale) followed by general method **GP 1**.

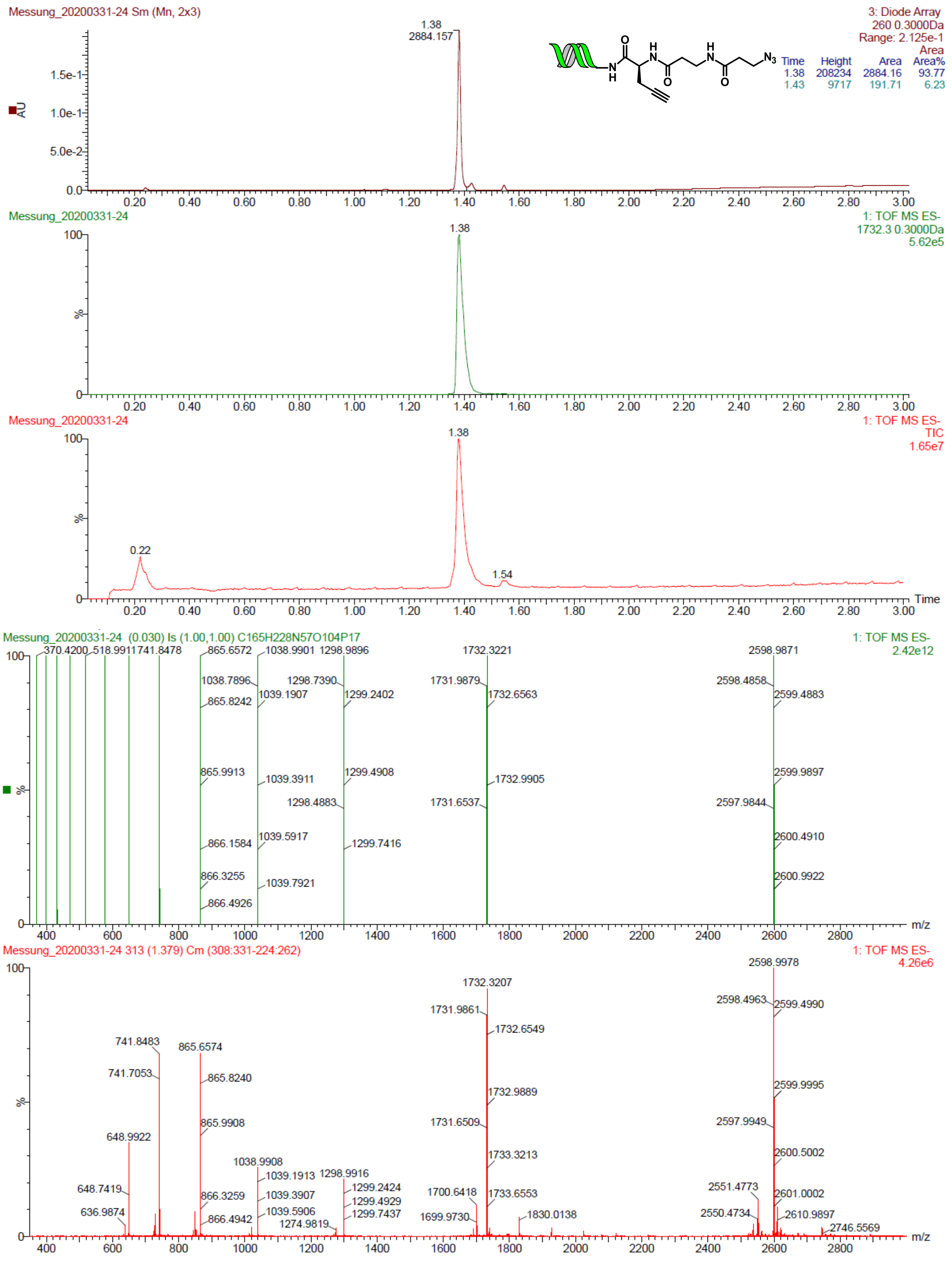

*Figure 3.102: LC-MS chromatogram of compound **Table 3 Entry 7 azide** t_R = 1,38 min TOF-MS-ESI$^-$ m/z = 1732.321(100%) [M-3H]$^{3-}$ (calc. 1732.322 for $C_{165}H_{228}N_{57}O_{104}P_{17}$)*

[cyclo-β-Ala-β-Ala-Pra]-OH conjugated with HP-280 (Table 3 Entry 7 Cyclo-Triazole):

The reaction was carried out using method **CuAAC 1** (1 µl, 10 mM of **(Table 3 Entry 7 azide)**, 10nmol scale).

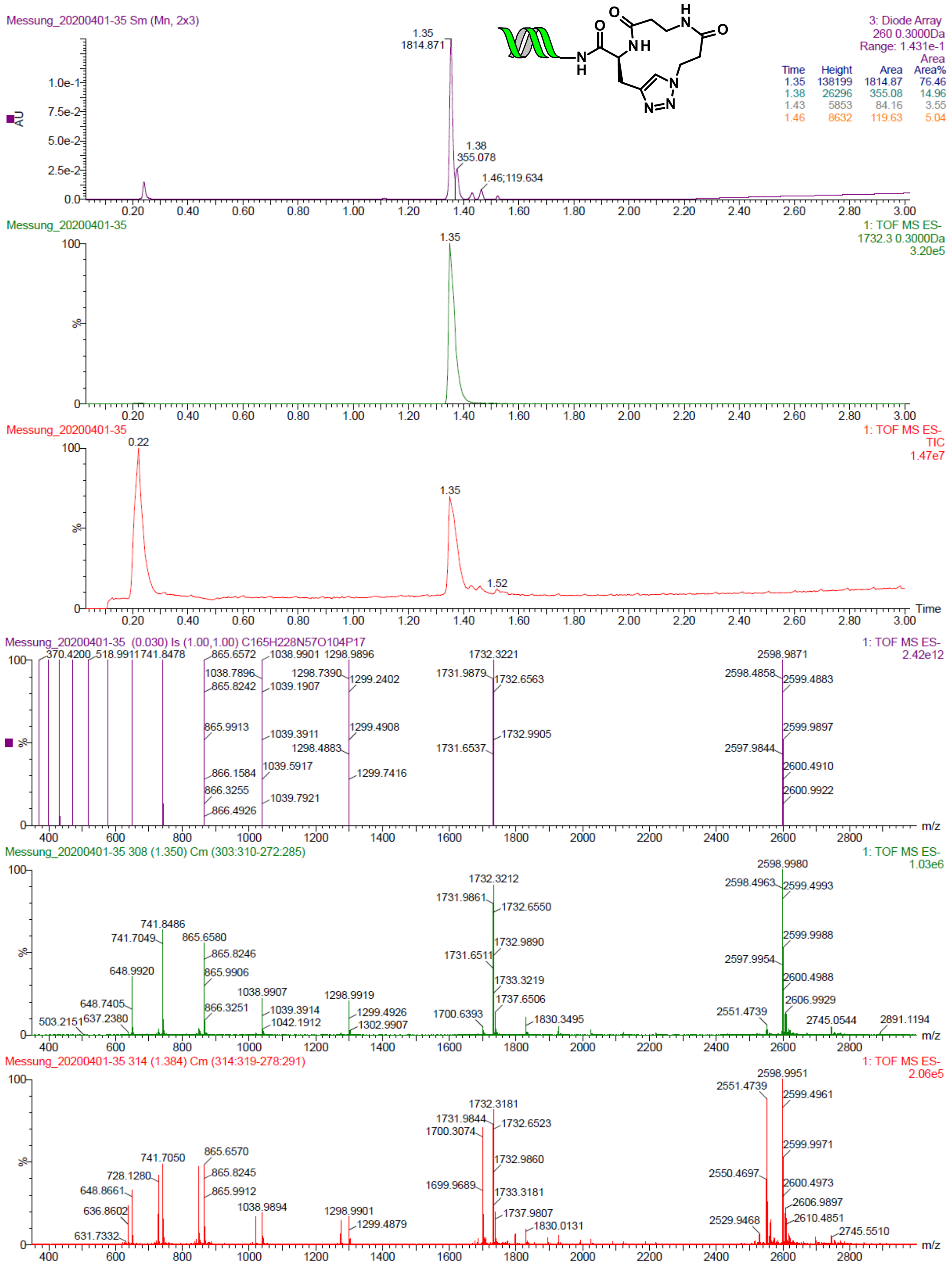

*Figure 3.103: LC-MS chromatogram of compound **Table 3 Entry 7 cyclo-triazole** t_R =1,35 min TOF-MS-ESI$^-$ m/z = 1732.323(100%) [M-3H]$^{3-}$ (calc. 1732.322 for $C_{165}H_{228}N_{57}O_{104}P_{17}$)*

Linear Triazole of Azido-β-Ala-β-Ala-Pra-OH conjugated with HP-280 (Table 3 Entry 7 Linear-Triazole):

The reaction was carried out using method **CuAAC 2** (1 µl, 10 mM of **(Table 3 Entry 7 azide)**, 10nmol scale).

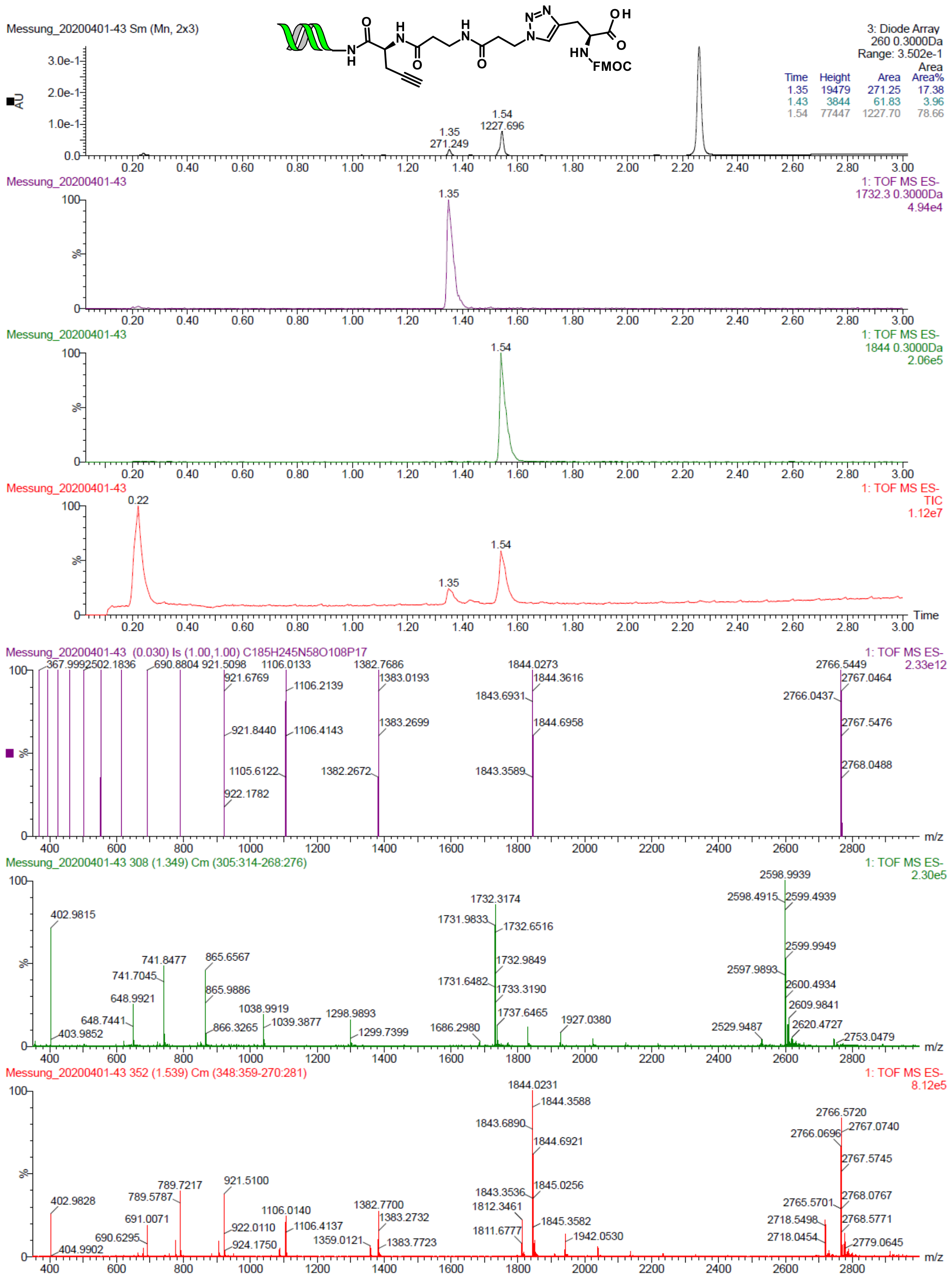

Figure 3.104: LC-MS chromatogram of compound ***Table 3 Entry 7 linear-triazole*** *t_R = 1,54 min TOF-MS-ESI$^-$ m/z = 1884.023(100%) $[M-3H]^{3-}$ (calc. 1884.027 for $C_{185}H_{245}N_{58}O_{108}P_{17}$)*

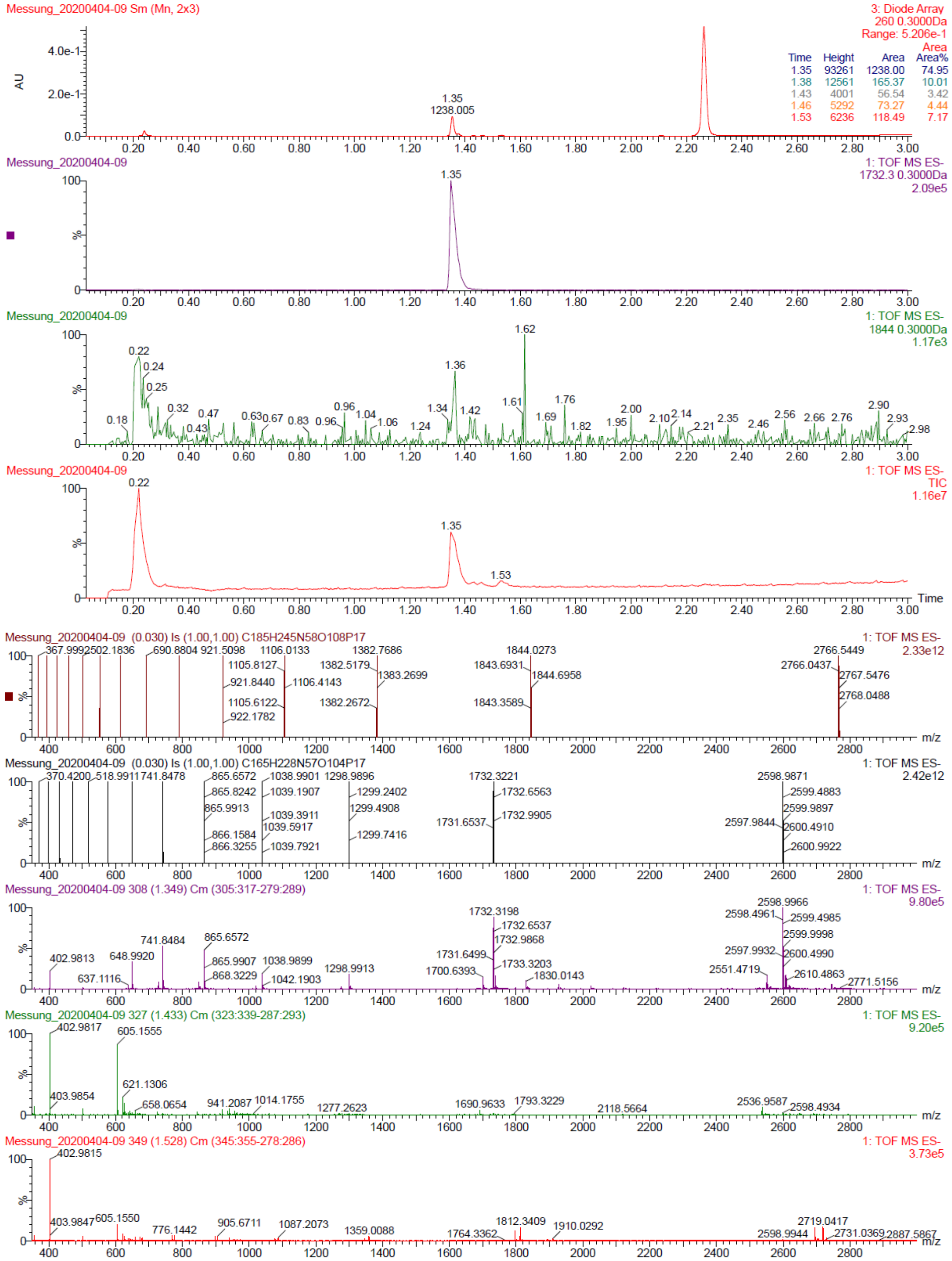

*Figure 3.105: LC-MS chromatogram of compound **Table 3 Entry 7 cyclo-triazole** reaction with Fmoc-Pra-OH*

3.4.9 [cyclo-γ-Abu-β-Ala-Pra]-OH conjugated with HP-280 (Table 3 Entry 8)

H-γ-Abu-β-Ala-Pra-OH conjugated with HP-280 (Table 3 Entry 8 Amine):

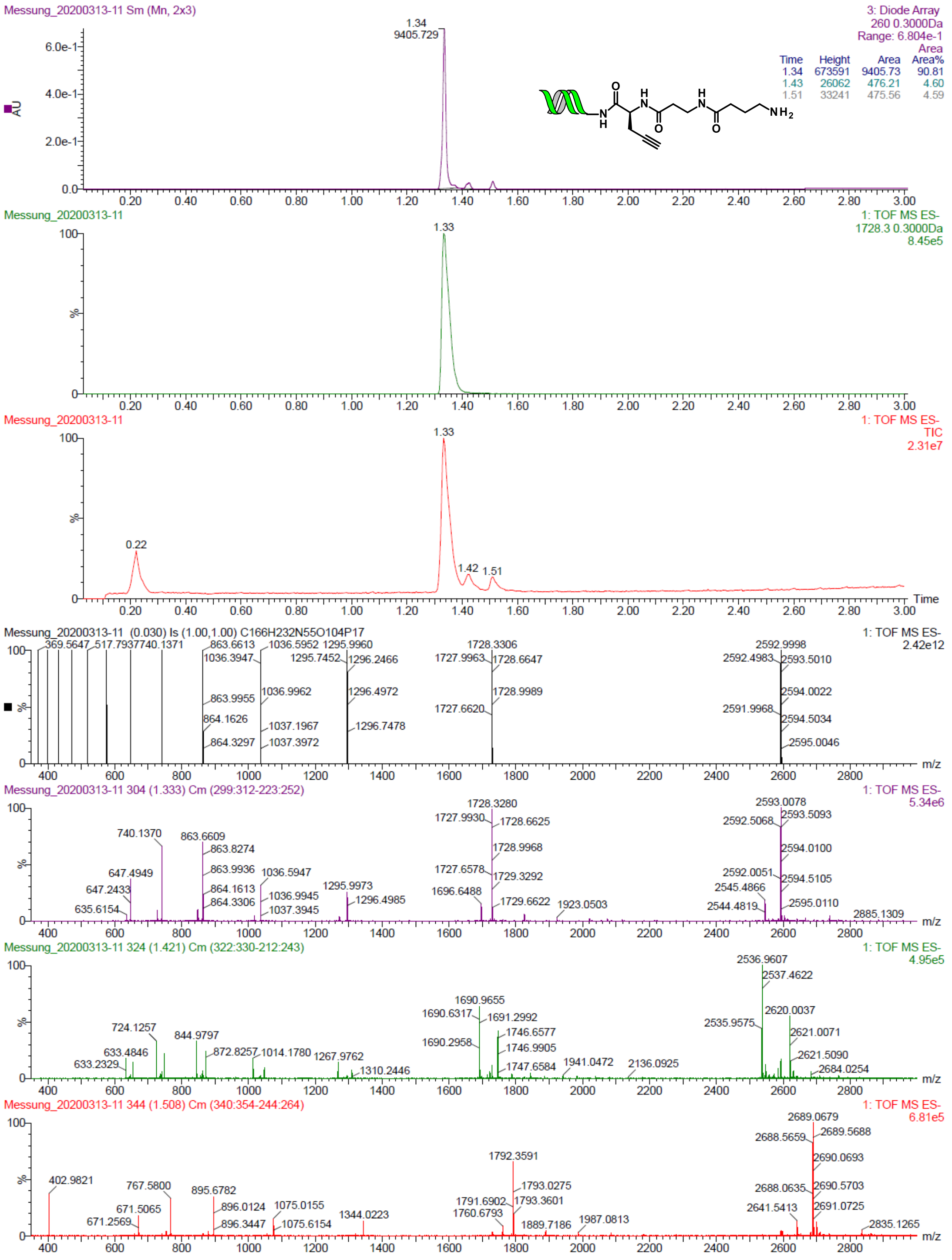

*Figure 3.106: LC-MS chromatogram of compound **Table 3 Entry 8 amine** t_R = 1,34 min TOF-MS-ESI$^-$ m/z = 1728.328(100%) $[M-3H]^{3-}$ (calc. 1728.331 for $C_{166}H_{232}N_{55}O_{104}P_{17}$)*

Azido-γ-Abu-β-Ala-Pra-OH conjugated with HP-280 (Table 3 Entry 8 Azide):

The reaction was carried out by using the general method DTR 1 (15 µl, 10 mM of **(Table 3 Entry 8 amine)**, 150nmol scale) followed by general method **GP 1**.

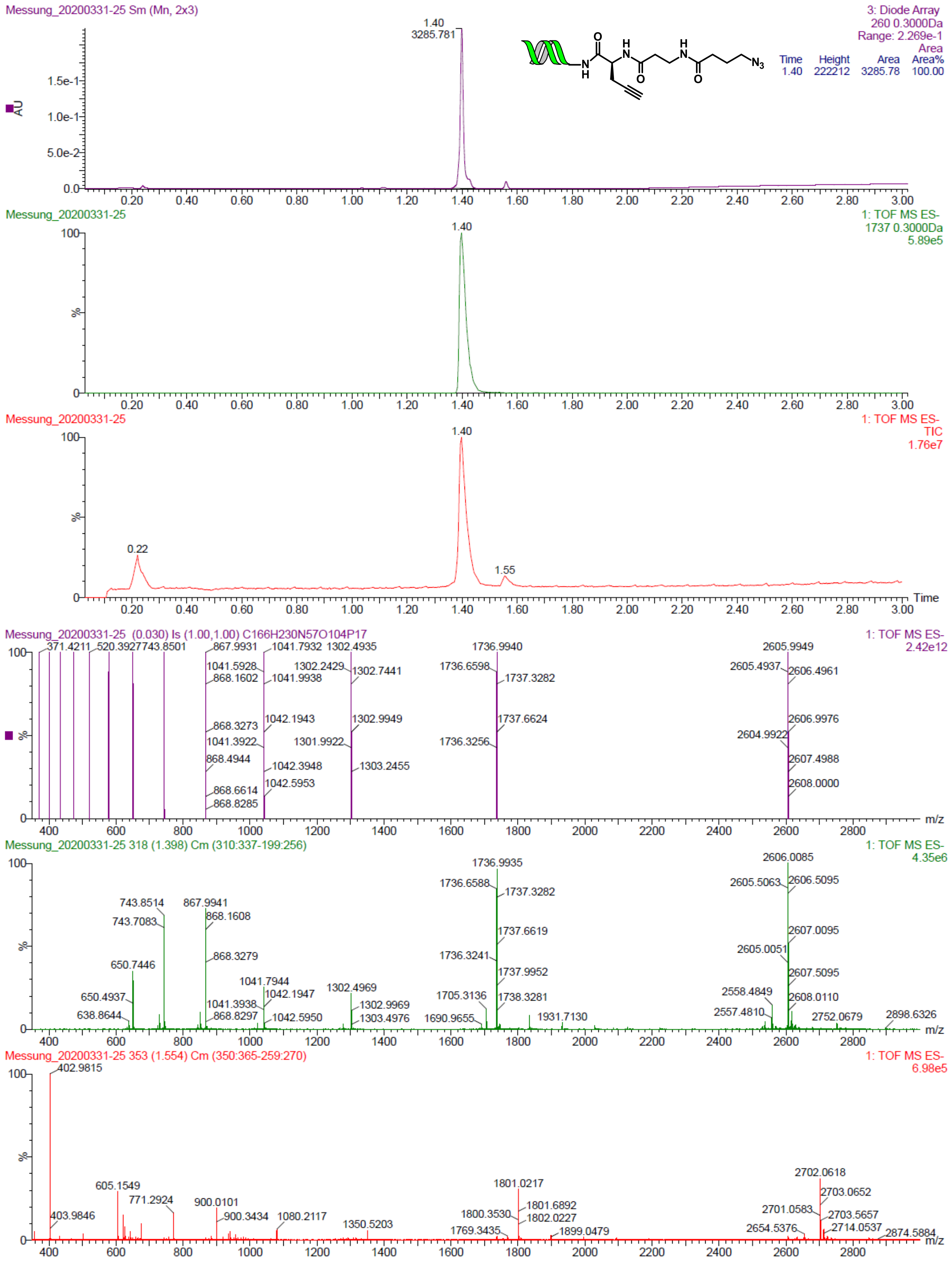

*Figure 3.107: LC-MS chromatogram of compound **Table 3 Entry 8azide** t_R = 1,40 min TOF-MS-ESI$^-$ m/z = 1736.994(100%) $[M-3H]^{3-}$ (calc. 1736.994 for $C_{166}H_{230}N_{57}O_{104}P_{17}$)*

[cyclo-γ-Abu-β-Ala-Pra]-OH conjugated with HP-280 (Table 3 Entry 8 Cyclo-Triazole):

The reaction was carried out using method **CuAAC 1** (1 µl, 10 mM of **(Table 3 Entry 8 azide)**, 10nmol scale).

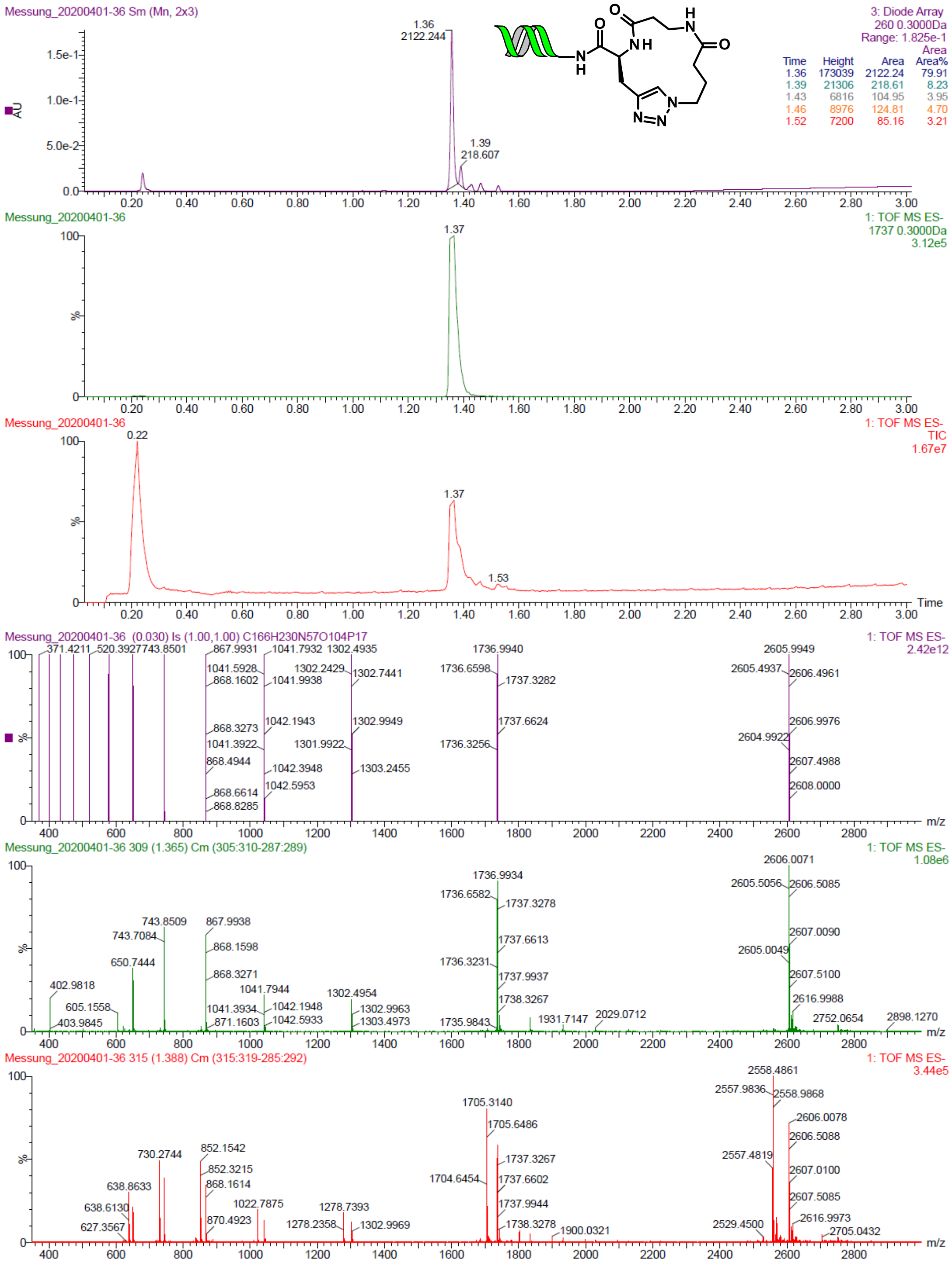

*Figure 3.108: LC-MS chromatogram of compound **Table 3 Entry 8 cyclo-triazole** t_R = 1,36 min TOF-MS-ESI$^-$ m/z = 1736.993(100%) [M-3H]$^{3-}$ (calc. 1736.994 for $C_{166}H_{230}N_{57}O_{104}P_{17}$)*

Linear Triazole of Azido-γ-Abu-β-Ala-Pra-OH conjugated with HP-280 (Table 3 Entry 8 Linear-Triazole):

The reaction was carried out using method **CuAAC 2** (1 µl, 10 mM of **(Table 3 Entry 8 azide)**, 10nmol scale).

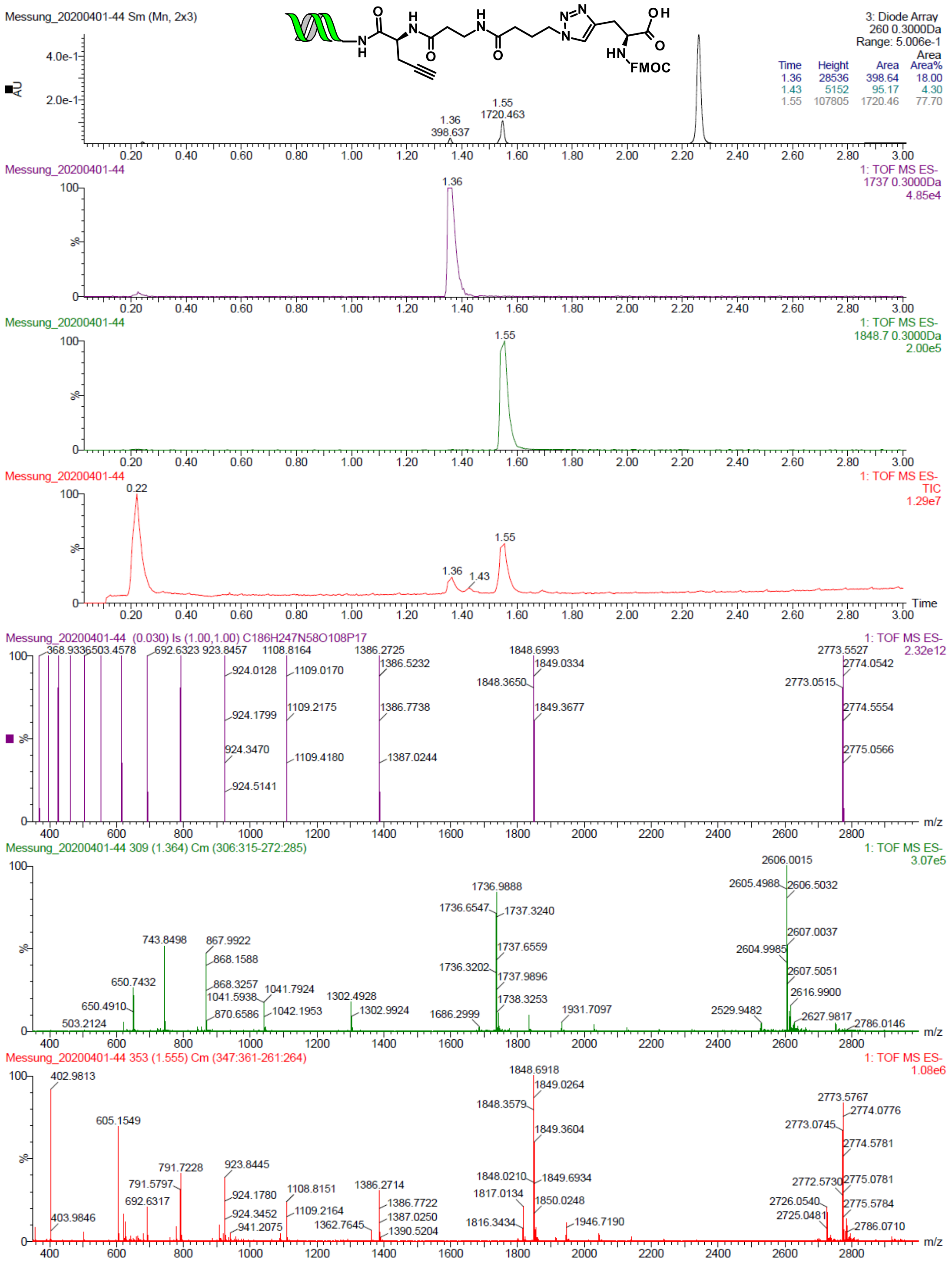

*Figure 3.109: LC-MS chromatogram of compound **Table 3 Entry 8 linear-triazole** t_R = 1,55 min TOF-MS-ESI⁻ m/z = 1848.692(100%) $[M-3H]^{3-}$ (calc. 1848.699 for $C_{186}H_{247}N_{58}O_{108}P_{17}$)*

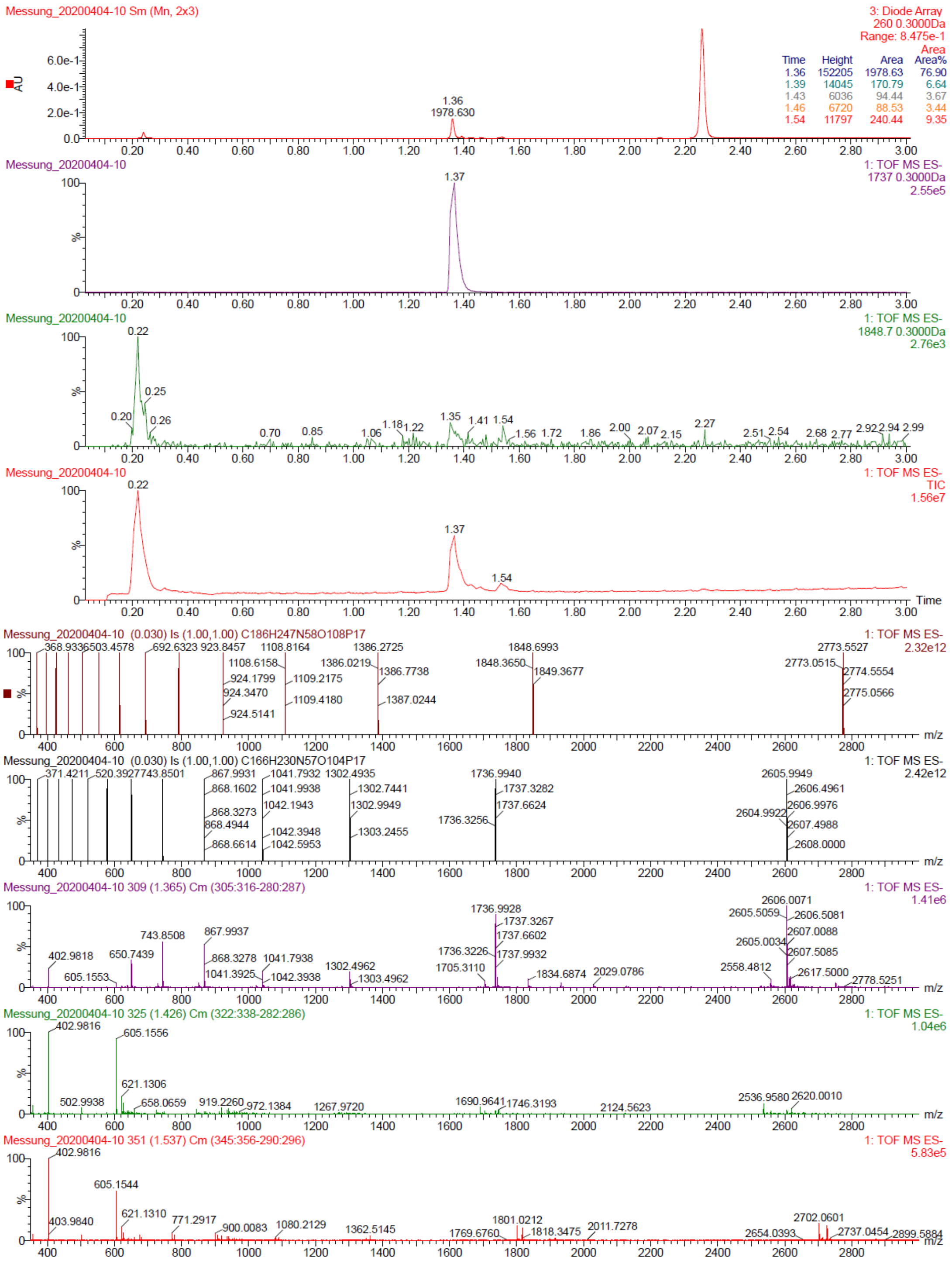

Figure 3.110: LC-MS Chromatogram of compound ***Table 3 Entry 8 cyclo-triazole reaction*** *with Fmoc-Pra-OH*

3.4.10 [cyclo-3-Abz-β-Ala-Pra]-OH conjugated with HP-280 (Table 3 Entry 9)

H-3-Abz-β-Ala-Pra-OH conjugated with HP-280 (Table 3 Entry 9 Amine):

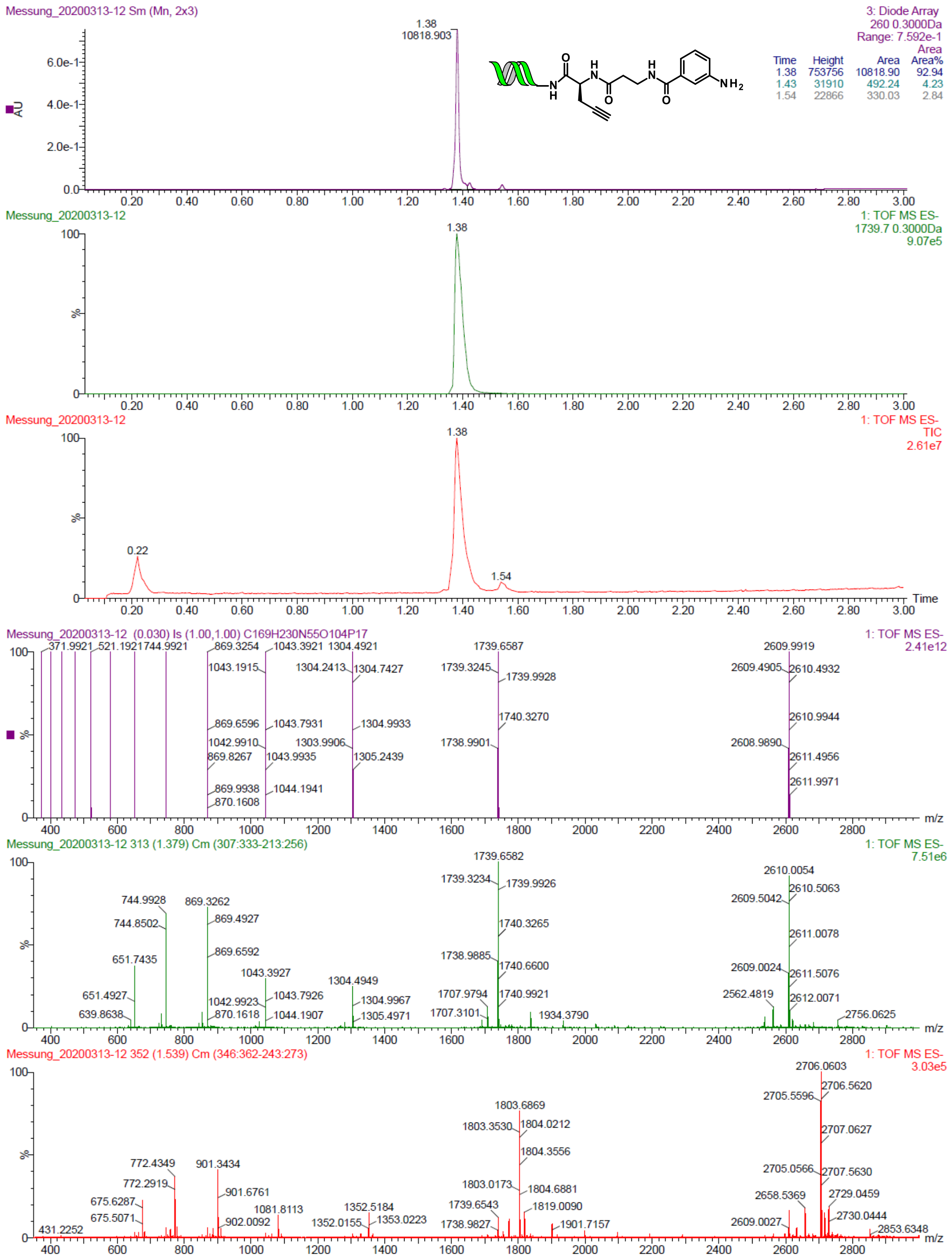

*Figure 3.111: LC-MS chromatogram of compound **Table 3 Entry 9 amine** t_R = 1,38 min TOF-MS-ESI$^-$ m/z = 1739.658(100%) $[M-3H]^{3-}$ (calc. 1739.659 for $C_{169}H_{230}N_{55}O_{104}P_{17}$)*

Azido-3-Abz-β-Ala-Pra-OH conjugated with HP-280 (Table 3 Entry 9 azide):

The reaction was carried out by using the general method DTR 1 (15 µl, 10 mM of **(Table 3 Entry 9 amine)**, 150nmol scale) followed by general method **GP 1**.

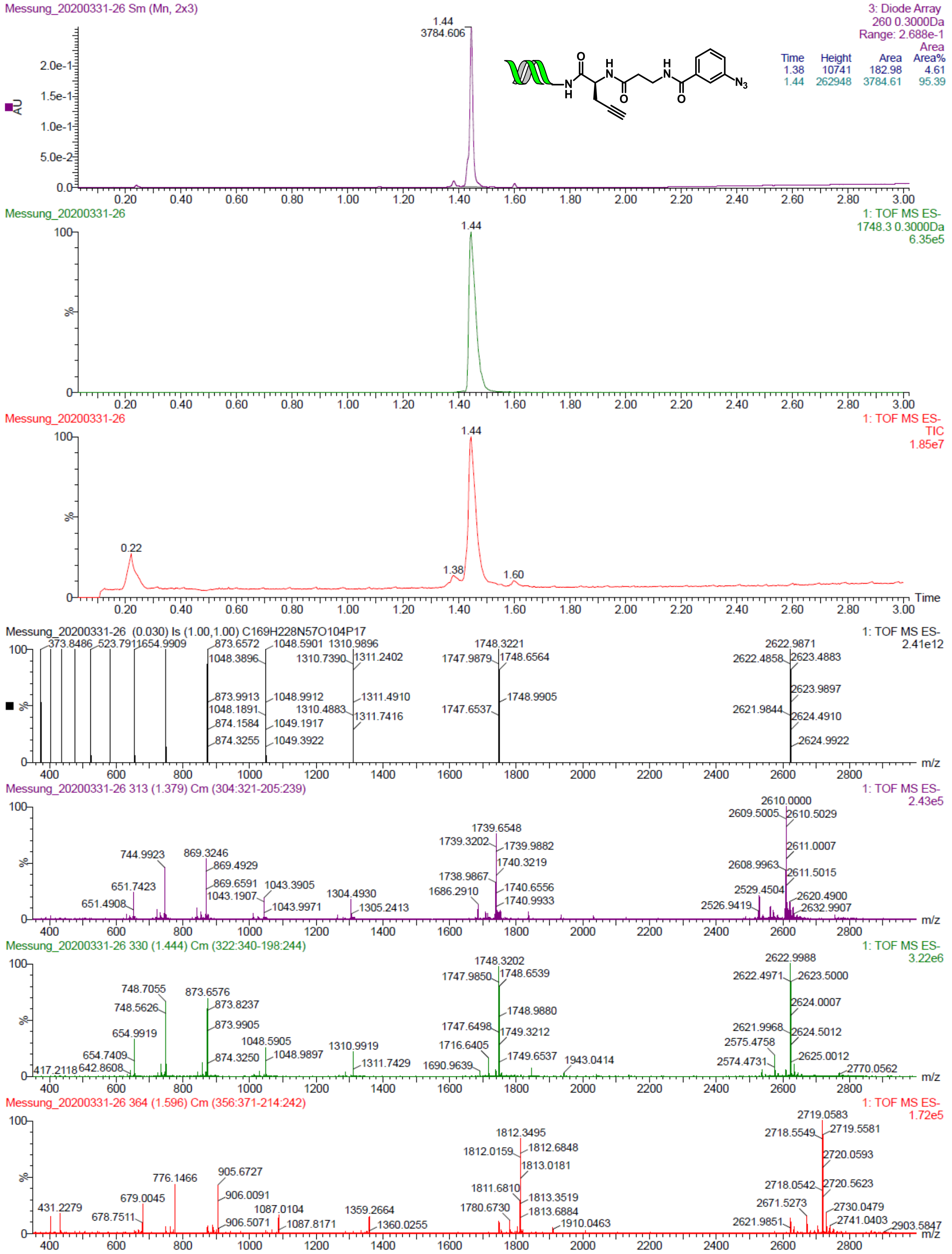

*Figure 3.112: LC-MS chromatogram of compound **Table 3 Entry 9 azide** t_R = 1,44 min TOF-MS-ESI⁻ m/z = 1748.320(100%) $[M-3H]^{3-}$ (calc. 1748.322 for $C_{169}H_{228}N_{57}O_{104}P_{17}$)*

[cyclo-3-Abz-β-Ala-Pra]-OH conjugated with HP-280 (Table 3 Entry 9 Cyclo-Triazole):

The reaction was carried out using method **CuAAC 1** (1 µl, 10 mM of **(Table 3 Entry 9 azide)**, 10nmol scale).

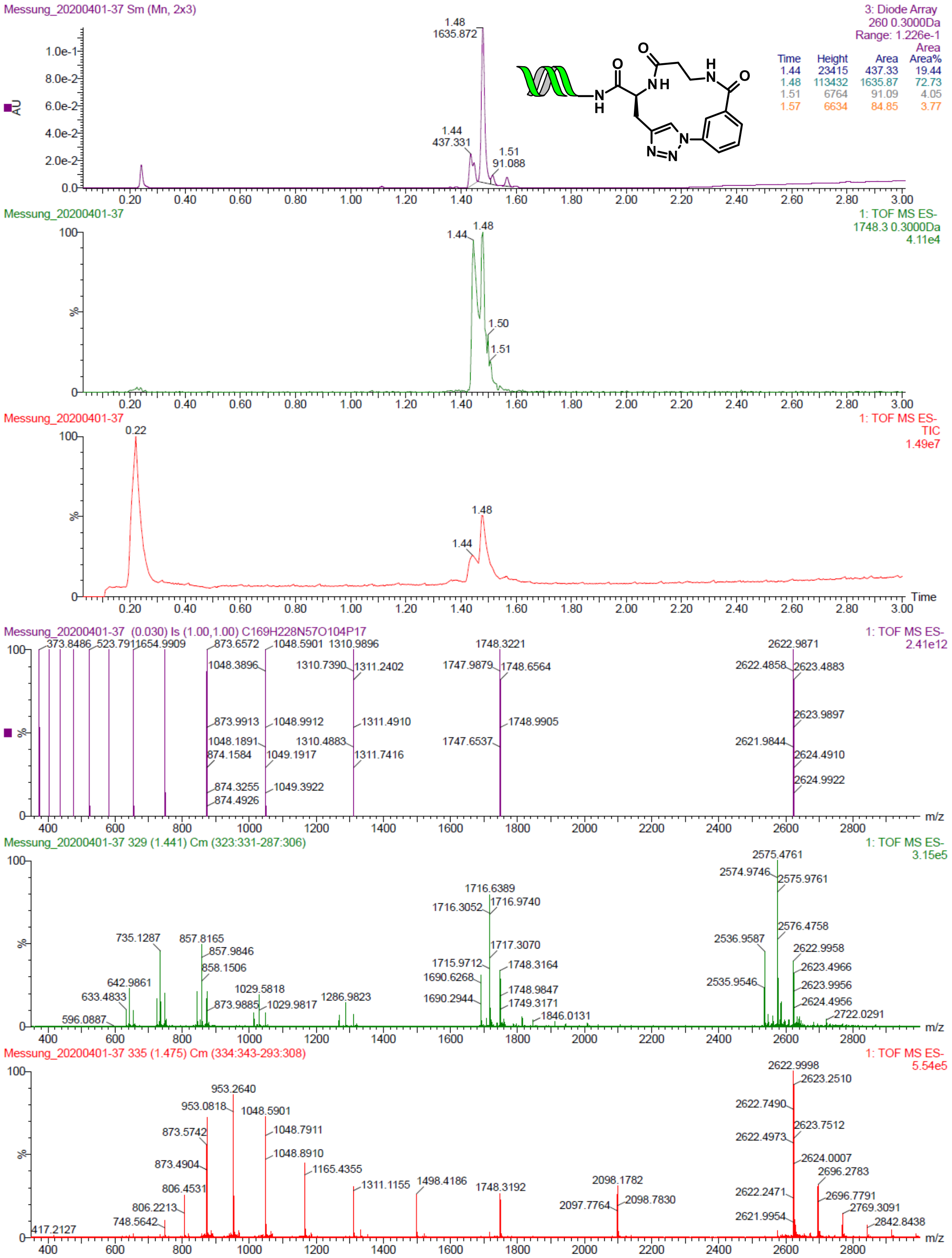

*Figure 3.113: LC-MS chromatogram of compound **Table 3 Entry 9 yclo-triazole** t_R = 1,48 min TOF-MS-ESI$^-$ m/z = 1748.319(100%) [M-3H]$^{3-}$ (calc. 1748.322 for $C_{169}H_{228}N_{57}O_{104}P_{17}$)*

Linear Triazole of Azido-3-Abz-β-Ala-Pra-OH conjugated with HP-280 (Table 3 Entry 9 Linear-Triazole):

The reaction was carried out using method **CuAAC 2** (1 µl, 10 mM of **(Table 3 Entry 9 azide)**, 10nmol scale).

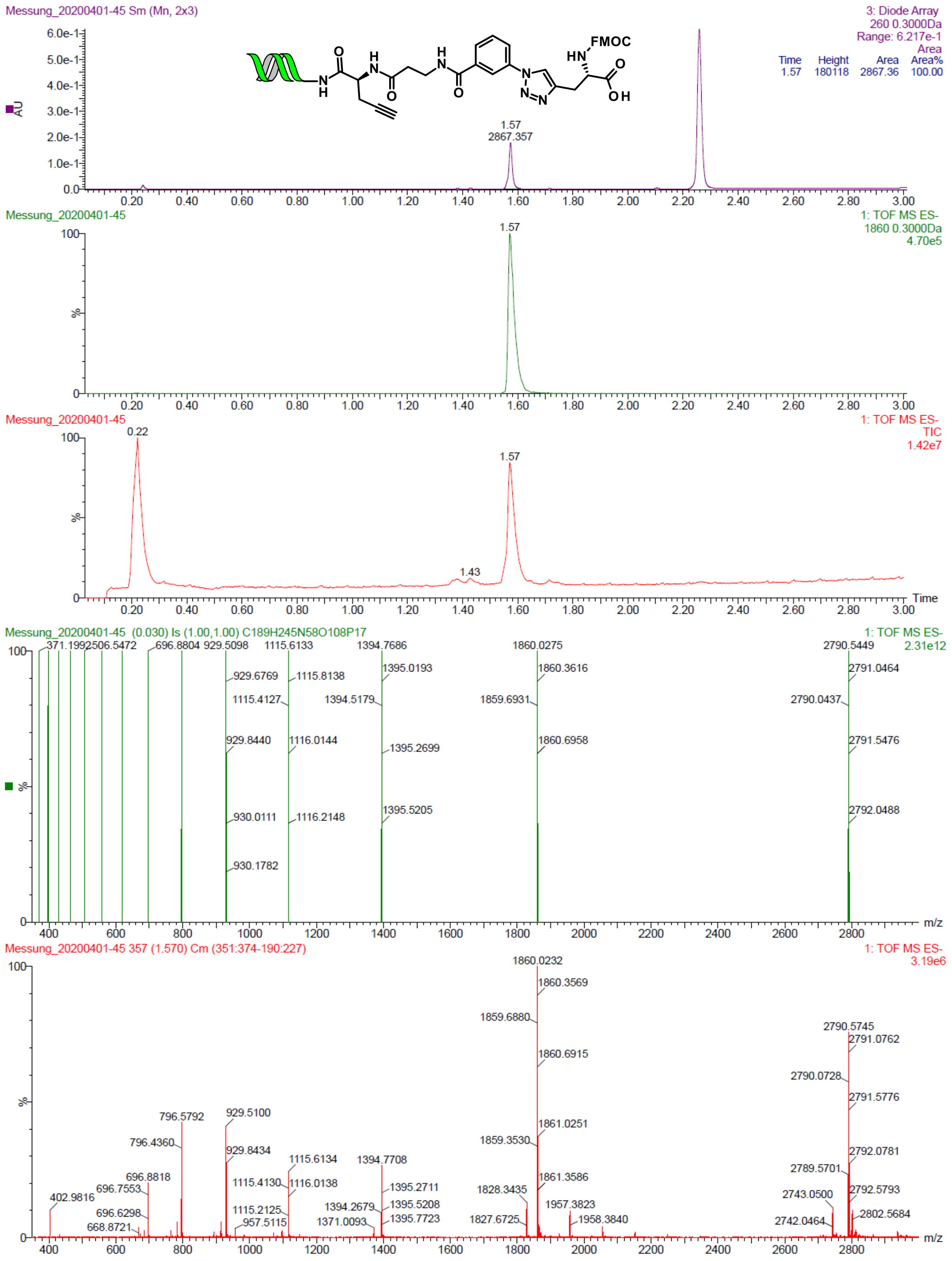

*Figure 3.114: LC-MS chromatogram of compound **Table 3 Entry 9 linear-triazole** t_R = 1,57 min TOF-MS-ESI$^-$ m/z = 1860.032(100%) [M-3H]$^{3-}$(calc. 1860.028 for $C_{189}H_{245}N_{58}O_{108}P_{17}$)*

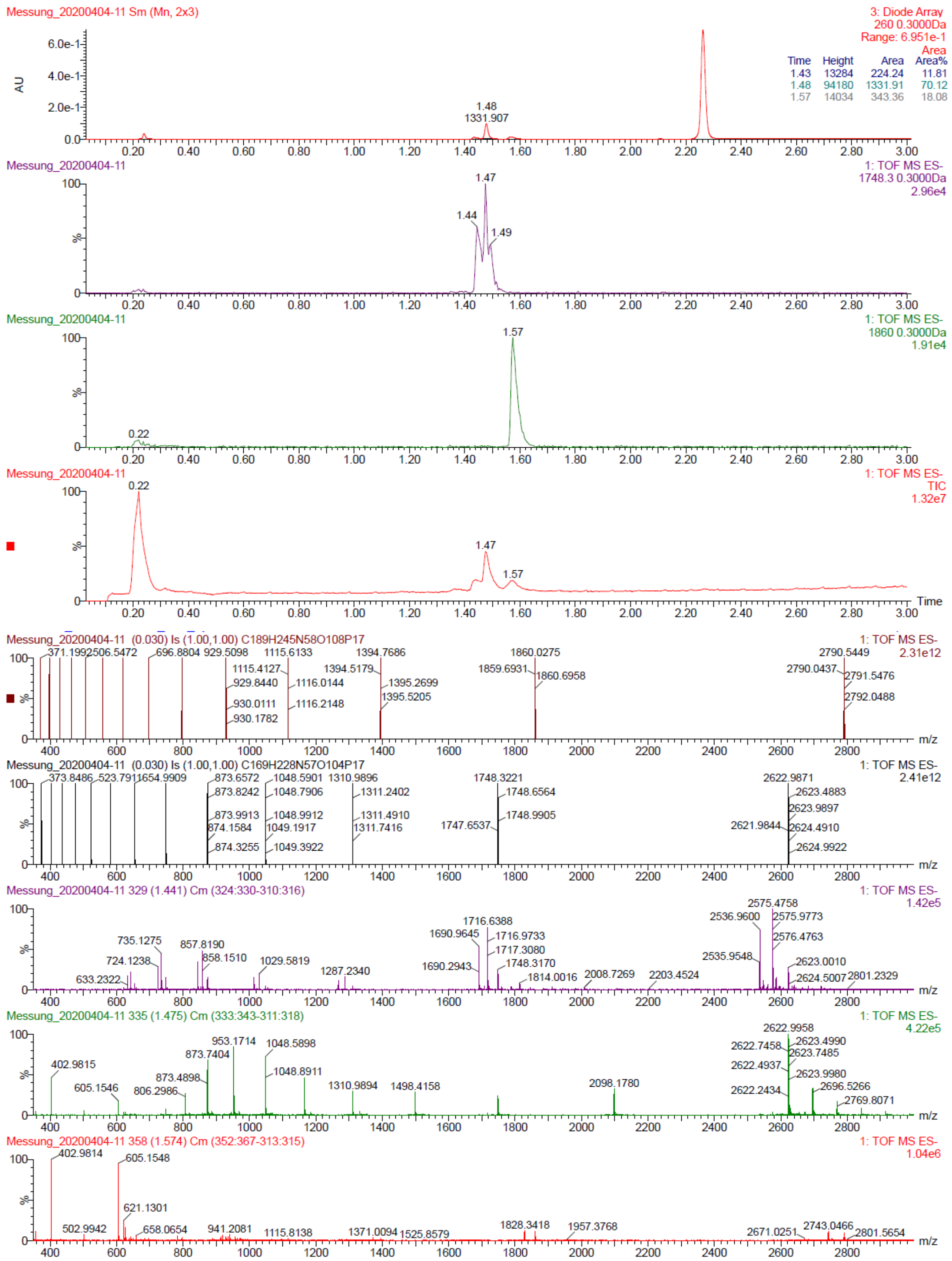

*Figure 3.115: LC-MS chromatogram of compound **Table 3 Entry 9 cyclo-triazole** reaction with Fmoc-Pra-OH*

3.5 Methods and NMRs of Off-DNA Compounds (8-19)

3.5.1 Azido-Gly-Phe-Pra-NH_2 (12)

H-Gly-Phe-Pra-NH_2.TFA (8):

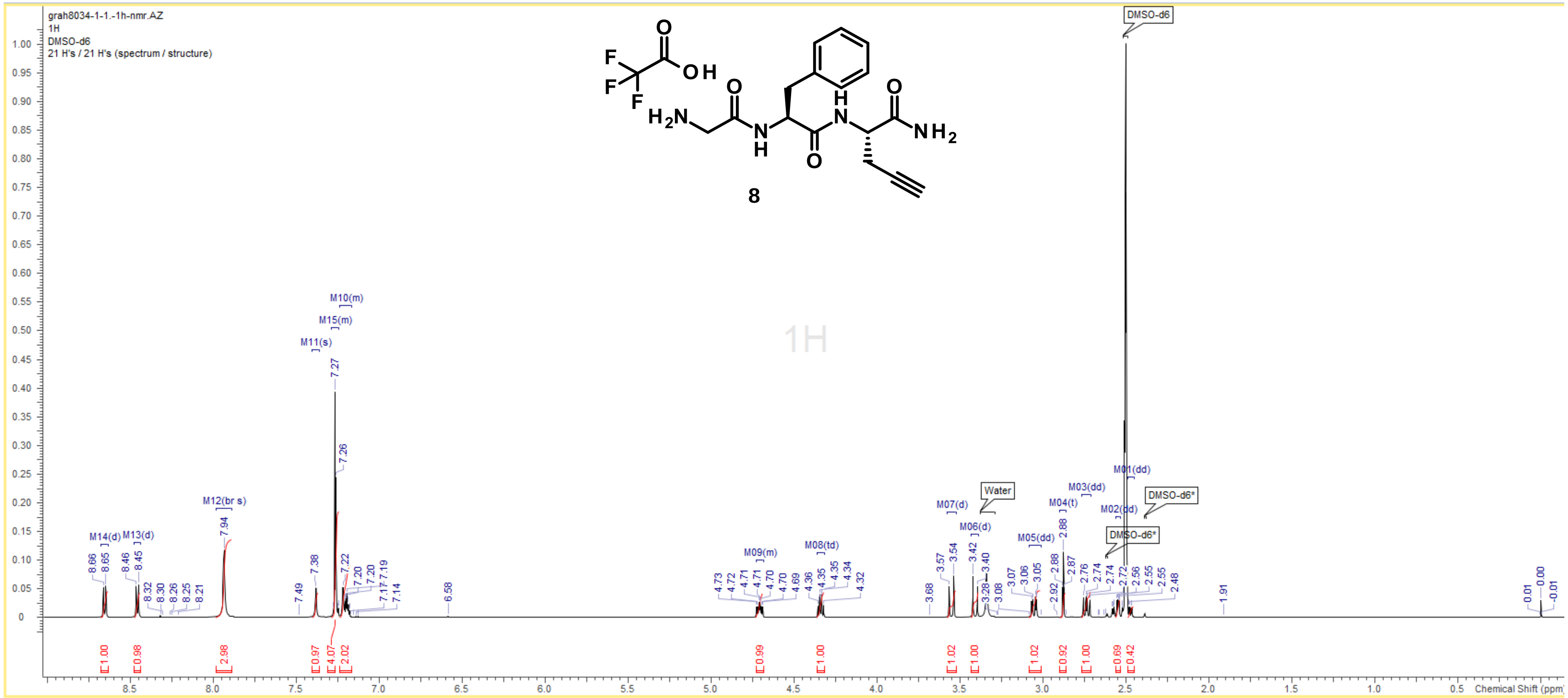

*Figure 3.116: ^{1}H-NMR of H-Gly-Phe-Pra-NH_2.TFA **(8)***

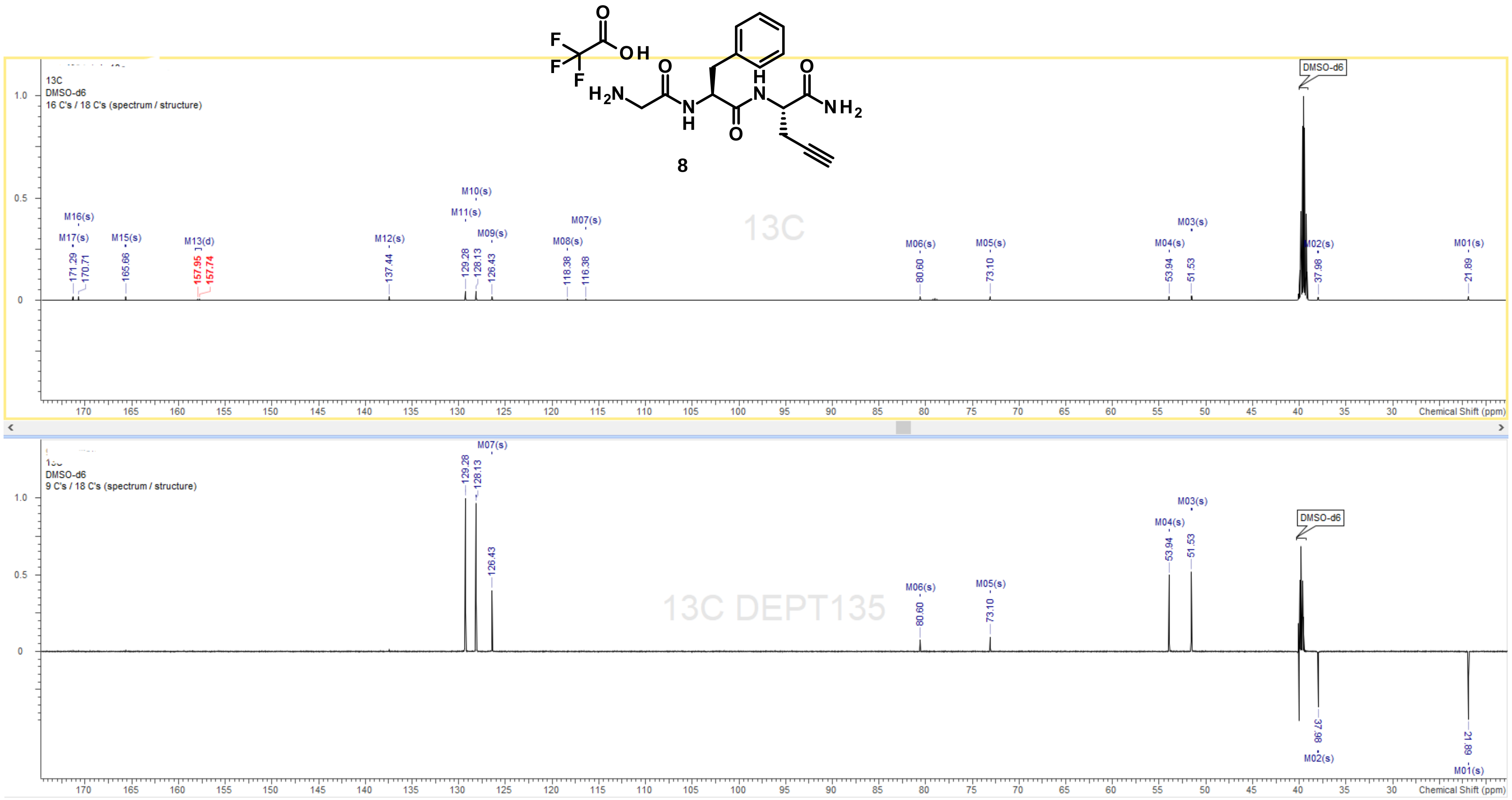

Figure 3.117: ^{13}C-NMR-BB (top) and ^{13}C-NMR-DEPT (bottom) of H-Gly-Phe-Pra-NH_2.TFA ***(8)***

Compound delivered by Peptide Synthesis Laboratory:

^{1}H NMR (600 MHz, DMSO-d_6) δ ppm 2.47 (dd, *J*=7.63, 2.67 Hz, 1 H), 2.55 (dd, *J* = 5.72, 2.67 Hz, 1 H), 2.74 (dd, *J* = 13.73, 9.92 Hz, 1 H), 2.88 (t, *J* = 2.67 Hz, 1 H), 3.05 (dd, *J* = 14.11, 4.20 Hz, 1 H), 3.41 (d, *J* = 16.02 Hz, 1 H), 3.55 (d, *J* = 16.02 Hz, 1 H), 4.34 (td, *J* = 7.72, 5.91 Hz, 1 H), 4.69 - 4.73 (m, 1 H), 7.17 - 7.24 (m, 2 H), 7.24 - 7.29 (m, 4 H), 7.38 (s, 1 H), 7.94 (br s, 3 H), 8.46 (d, *J* = 8.01 Hz, 1 H), 8.65 (d, *J* = 8.39 Hz, 1 H);

^{13}C NMR-BB (151 MHz, DMSO-d_6) δ ppm 21.89 (s, 1 C), 37.98 (s, 1 C), 40.05 (s, 1 C), 51.53 (s, 1 C), 53.94 (s, 1 C), 73.10 (s, 1 C), 80.60 (s, 1 C), 113.65 - 121.13 (m, 1 C), 126.43 (s, 1 C), 128.13 (s, 2 C), 129.28 (s, 2 C), 137.44 (s, 1 C), 157.84 (d, *J* = 30.52 Hz, 1 C), 165.66 (s, 1 C), 170.71 (s, 1 C), 171.29 (s, 1 C);

^{13}C NMR-DEPT (151 MHz, DMSO-d_6) δ ppm 21.89 (s, 1C), 37.98 (s, 1 C), 51.53 (s, 1 C), 53.94 (s, 1 C), 73.10 (s, 1 C), 80.60 (s, 1 C), 126.43 (s, 1 C), 128.13 (s, 2 C), 129.28 (s, 2 C);

MS ES+ *m/z* = 317.228 $[M+H]^+$ ($C_{16}H_{20}N_4O_5$).

Azido-Gly-Phe-Pra-NH_2 (12):

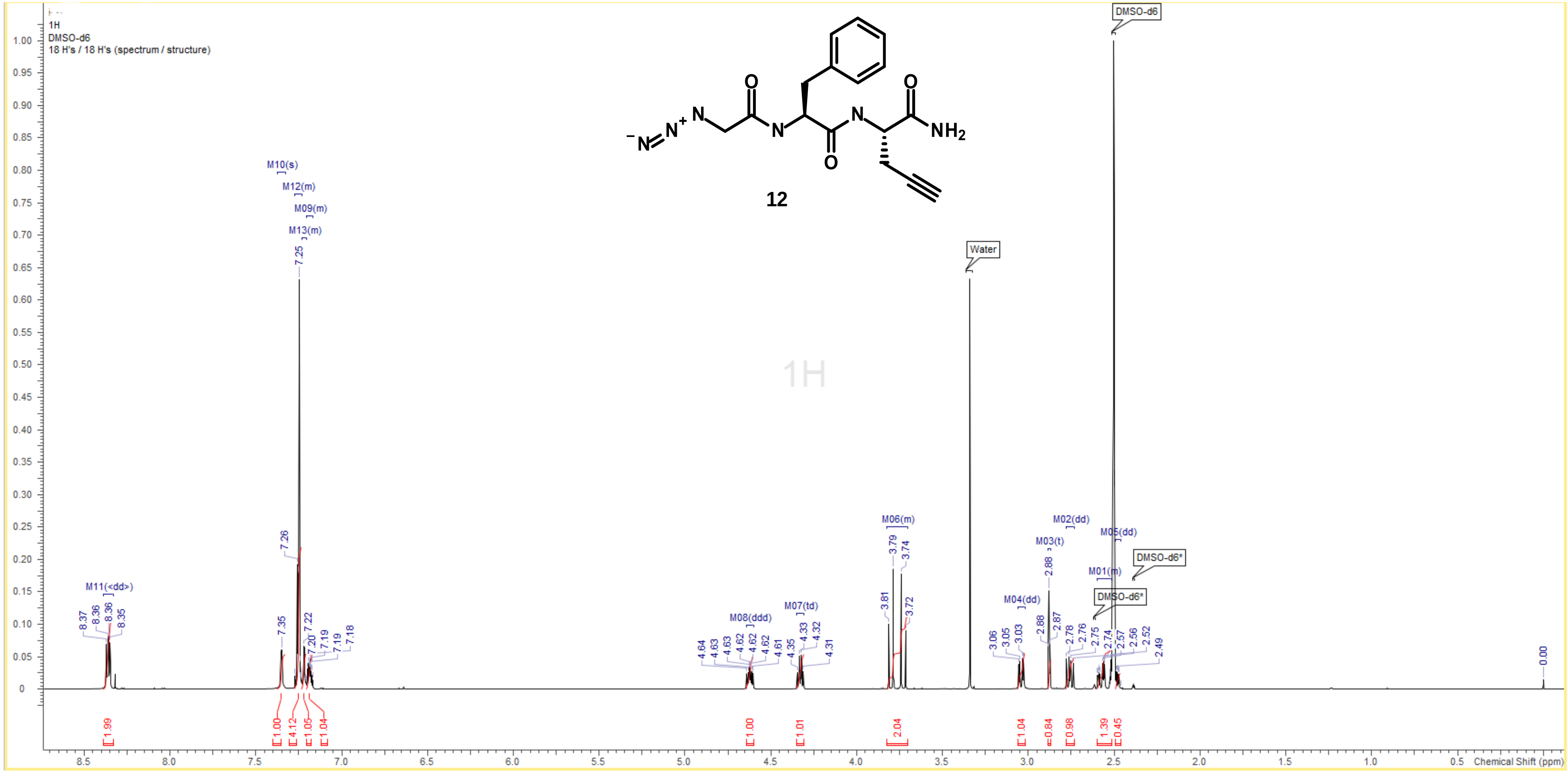

*Figure 3.118: ^{1}H-NMR of Azido-Gly-Phe-Pra-NH_2 **(12)***

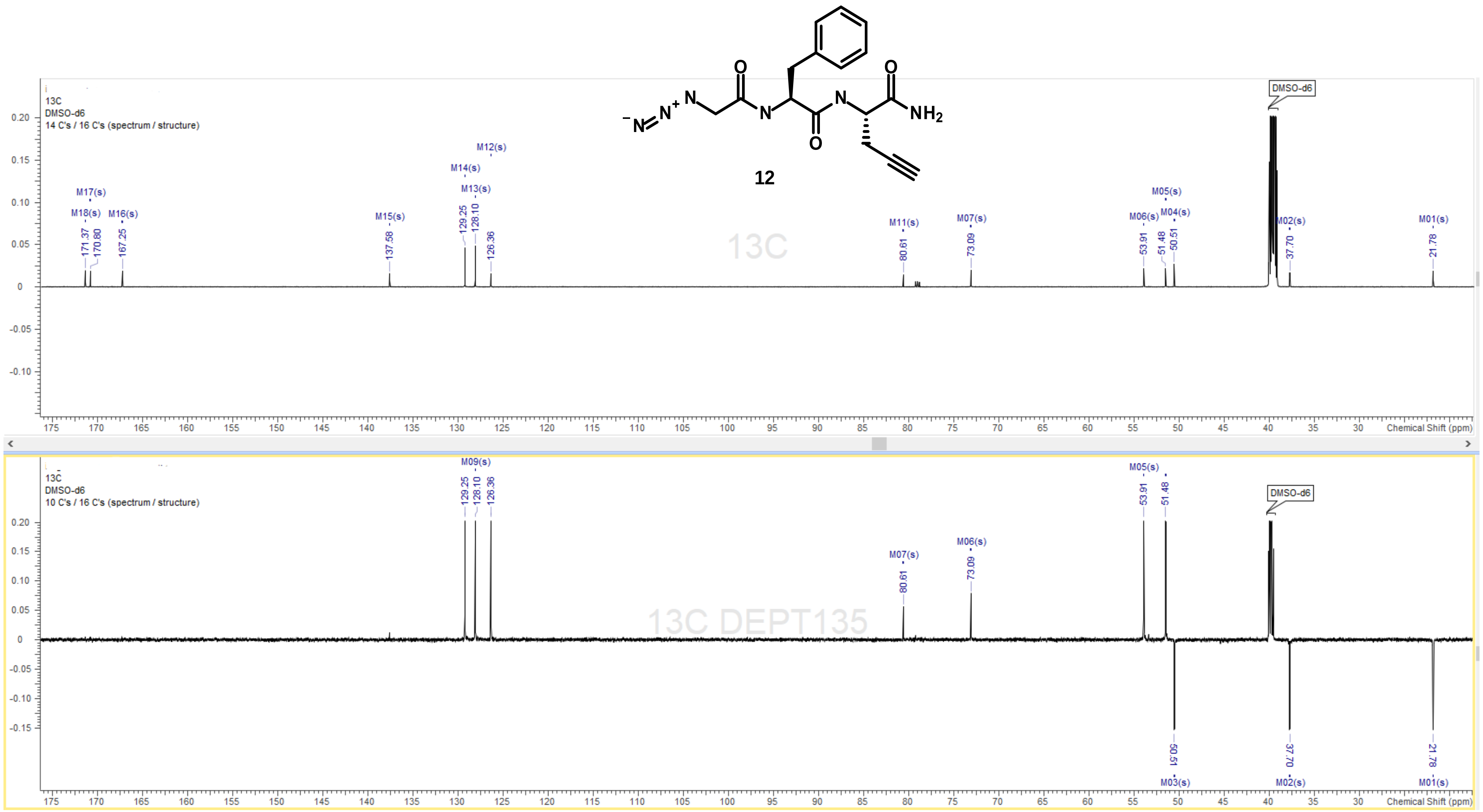

*Figure 3.119: ^{13}C-NMR-BB (top) and ^{13}C-NMR-DEPT (bottom) of Azido-Gly-Phe-Pra-NH_2 **(12)***

H-Gly-Phe-Pra-NH_2.TFA **(8)** (1 eq, 50 mg, 116 µmol) was dissolved in $NaHCO_3$ (7 eq, 4.07 ml, 200 mM aq.) and ISA×H_2SO_4 (2.5 eq, 563 µl, 500 mM aq.) and copper(II)sulfate pentahydrate (0.01 eq, 56.2 µl, 20 mM aq.) was added. The reaction was stirred for 16 h at room temperature and resulted in formation of s precipitate. The reaction was controlled by LC-MS. The precipitate was filtered, washed with water and dissolved in methanol. The LC-MS showed clean product **(12)**. The solution was concentrated. Yield: 33 mg white solid (96 µmol, 78.82%)

^{1}H NMR (600 MHz, DMSO-*d*$_6$) δ ppm 2.48 (dd, *J* = 7.63, 2.67 Hz, 1 H), 2.52 - 2.60 (m, 1 H), 2.76 (dd, *J* = 13.92, 9.73 Hz, 1 H), 2.88 (t, *J* = 2.67 Hz, 1 H), 3.04 (dd, *J* = 13.92, 4.39 Hz, 1 H), 3.70 - 3.82 (m, 2 H), 4.33 (td, *J* = 7.82, 6.10 Hz, 1 H), 4.62 (ddd, *J* = 9.73, 8.39, 4.39 Hz, 1 H), 7.17 - 7.20 (m, 1 H), 7.20 - 7.23 (m, 1 H), 7.23 - 7.27 (m, 4 H), 7.35 (s, 1 H), 8.36 (dd, *J* = 8.20, 5.53 Hz, 2 H);

^{13}C NMR-BB (151 MHz, DMSO-*d*$_6$) δ ppm 21.78 (s, 1 C), 37.70 (s, 1 C), 50.51 (s, 1 C), 51.48 (s, 1 C), 53.91 (s, 1 C), 73.09 (s, 1 C), 80.61 (s, 1 C), 126.36 (s, 1 C), 128.10 (s, 2 C), 129.25 (s, 2 C), 137.58 (s, 1 C), 167.25 (s, 1 C), 170.80 (s, 1 C), 171.37 (s, 1 C);

^{13}C NMR-DEPT (151 MHz, DMSO-*d*$_6$) δ ppm 21.78 (s, 1 C), 37.70 (s, 1 C), 50.51 (s, 1 C), 51.48 (s, 1 C), 53.91 (s, 1 C), 73.09 (s, 1 C), 80.61 (s, 1 C), 126.36 (s, 1 C), 128.10 (s, 2 C), 129.25 (s, 2 C);

MS ES+ *m/z* = 343.229 $[M+H]^+$, 365.216 $[M+Na]^+$ ($C_{16}H_{18}N_6O_3$).

3.5.2 Cyclo-1,4-Triazol[-β-Ala-Phe-Pra]-NH_2 (17)

H-β-Ala-Phe-Pra-NH_2.TFA (9):

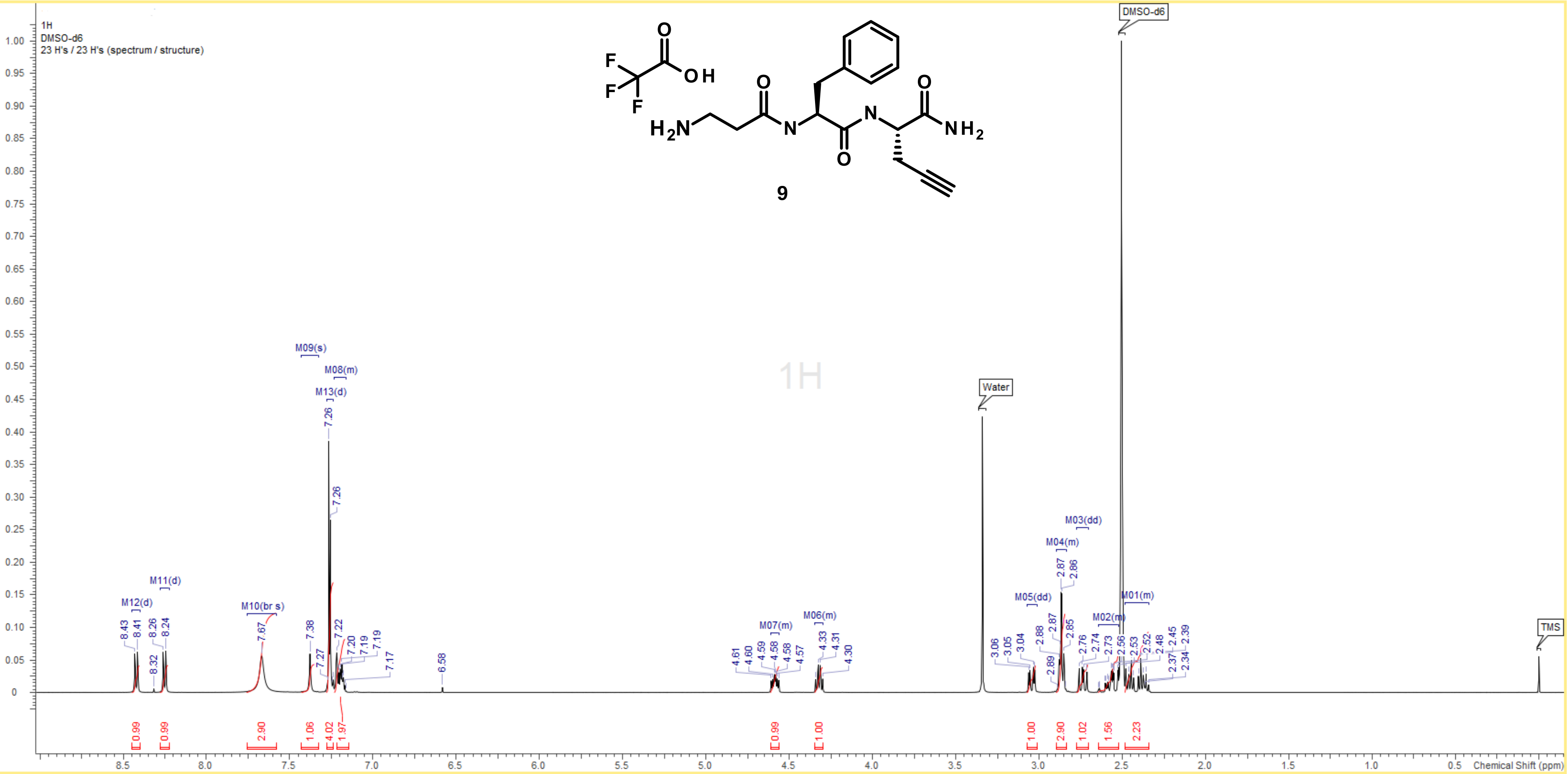

*Figure 3.120: ^{1}H-NMR of H-β-Ala-Phe-Pra-NH_2.TFA **(9)***

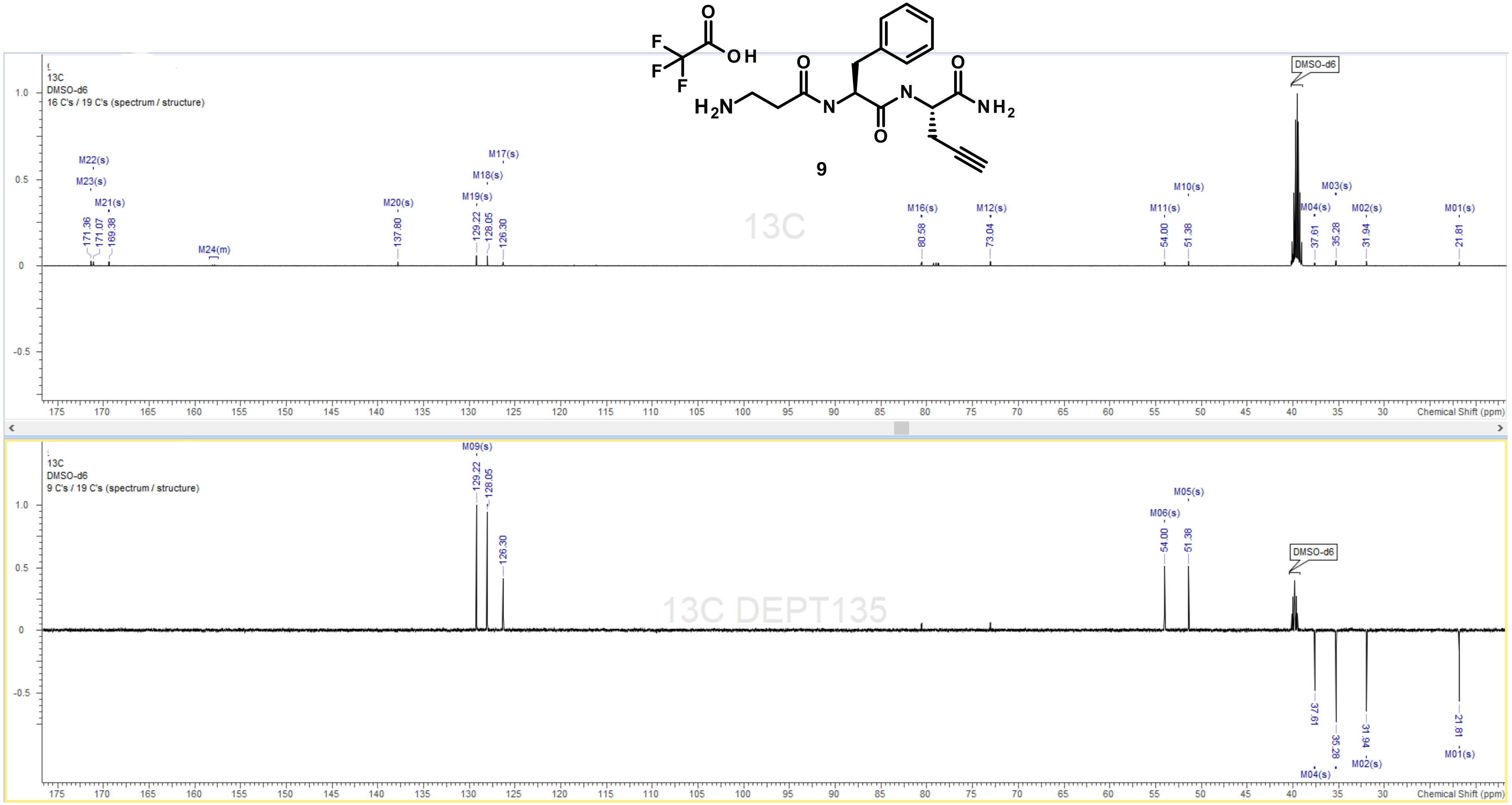

Figure 3.121: ^{13}C-NMR-BB (top) and ^{13}C-NMR-DEPT (bottom) of H-β-Ala-Phe-Pra-NH_2.TFA ***(9)***

Compound delivered by Peptide Synthesis Laboratory:

^{1}H NMR (500 MHz, DMSO-d_6) δ ppm 2.34 - 2.48 (m, 2 H), 2.52 - 2.64 (m, 2 H), 2.74 (dd, *J* = 13.67, 10.17 Hz, 1H), 2.84 - 2.90 (m, 3 H), 3.04 (dd, *J* = 13.67, 4.45 Hz, 1 H), 4.30 - 4.35 (m, 1 H), 4.56 - 4.61 (m, 1 H), 7.16 - 7.23 (m, 2 H), 7.26 (d, *J* = 4.45 Hz, 4 H), 7.38 (s, 1 H), 7.67 (br s, 3 H), 8.25 (d, *J* = 7.95 Hz, 1 H), 8.42 (d, *J* = 8.27 Hz, 1 H);

^{13}C NMR-BB (126 MHz, DMSO-d_6) δ ppm 21.81 (s, 1 C), 31.94 (s, 1 C), 35.28 (s, 1 C), 37.61 (s, 1 C), 51.38 (s, 1 C), 54.00 (s, 1 C), 73.04 (s, 1 C), 80.58 (s, 1 C), 126.30 (s, 1 C), 128.05 (s, 2 C), 129.22 (s, 2 C), 137.80 (s, 1 C), 157.46 - 158.43 (m, 1 C), 169.38 (s, 1 C), 171.07 (s, 1 C), 171.36 (s, 1 C);

^{13}C NMR DEPT (126 MHz, DMSO-d_6) δ ppm 21.81 (s, 1 C), 31.94 (s, 1 C), 35.28 (s, 1 C), 37.61 (s, 1 C), 51.38 (s, 1 C), 54.00 (s, 1 C), 126.30 (s, 1 C), 128.05 (s, 2 C), 129.22 (s, 2 C);

MS ES+ *m/z* = 331.247 [*M*+H]$^+$ ($C_{17}H_{22}N_4O_5$).

Azido-β-Ala-Phe-Pra-NH_2 (13):

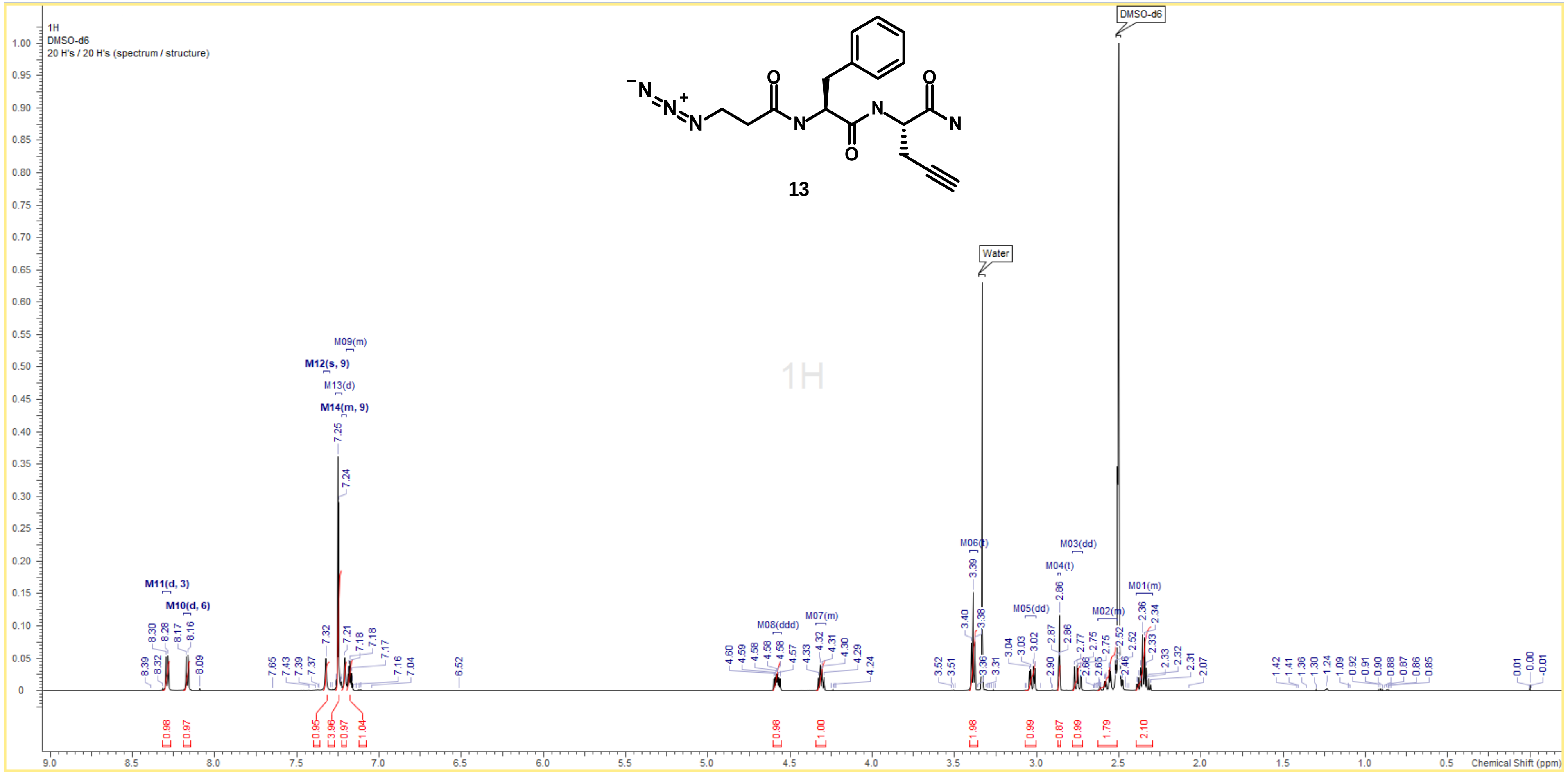

*Figure 3.122: ^{1}H-NMR of Azido-β-Ala-Phe-Pra-NH_2 **(13)***

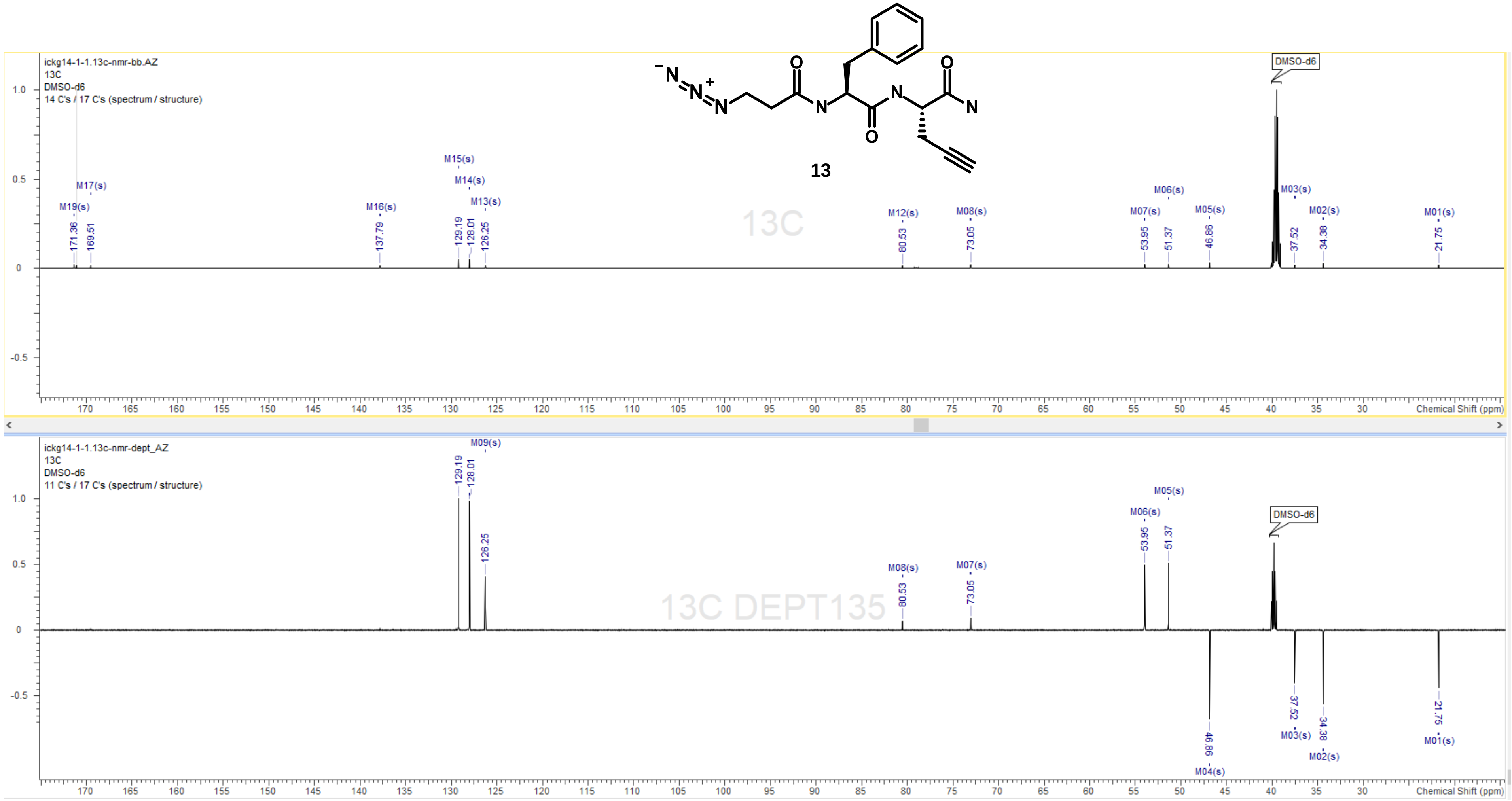

Figure 3.123: ^{13}C-NMR-BB (top) and ^{13}C-NMR-DEPT (bottom) of Azido-β-Ala-Phe-Pra-NH_2 ***(13)***

H-β-Ala-Phe-Pra-NH_2.TFA **(9)** (1 eq, 50 mg, 112 µmol) was dissolved in $NaHCO_3$ (7 eq, 3.96 ml, 200 mM aq.) and ISA×H_2SO_4 (2.5 eq, 563 µl, 500 mM aq.) and cupper(II)sulfate pentahydrate (0.01 eq, 56.2 µl, 20 mM aq.) was added. The reaction was stirred for 16 h at room and resulted in formation of s precipitate. The reaction was controlled by LC-MS. The precipitate was filtered, washed with water and dissolved in methanol. The LC-MS showed clean product **(13)**. The solution was concentrated. Yield: 39.1 mg white solid (110 µmol, 98.21%)

^{1}H NMR (600 MHz, DMSO-*d*$_6$) δ ppm 2.30 - 2.40 (m, 2 H) 2.51 - 2.63 (m, 2 H) 2.75 (dd, *J* = 13.73, 9.92 Hz, 1 H) 2.86 (t, *J* = 2.67 Hz, 1 H) 3.03 (dd, *J* = 14.11, 4.58 Hz, 1 H) 3.39 (t, *J* = 6.48 Hz, 2 H) 4.28 - 4.34 (m, 1 H) 4.58 (ddd, *J* = 9.63, 8.30, 4.58 Hz, 1 H) 7.15 - 7.20 (m, 1 H) 7.20 - 7.23 (m, 1 H) 7.25 (d, *J* = 4.20 Hz, 4 H) 7.32 (s, 1 H) 8.17 (d, *J* = 8.01 Hz, 1 H) 8.29 (d, *J* = 8.01 Hz, 1 H);

^{13}C NMR-BB (151 MHz, DMSO-*d*$_6$) δ ppm 21.75 (s, 1 C), 34.38 (s, 1 C), 37.52 (s, 1 C), 46.86 (s, 1 C), 51.37 (s, 1 C), 53.95 (s, 1 C), 73.05 (s, 1 C), 80.53 (s, 1 C), 126.25 (s, 1 C), 128.01 (s, 2 C), 129.19 (s, 2 C), 137.79 (s, 1 C), 169.51 (s, 1 C), 171.07 (s, 1 C), 171.36 (s, 1 C);

^{13}C NMR-DEPT (151 MHz, DMSO-*d*$_6$) δ ppm 21.75 (s, 1 C) 34.38 (s, 1 C) 37.52 (s, 1 C) 46.86 (s, 1 C) 51.37 (s, 1 C) 53.95 (s, 1 C) 73.05 (s, 1 C) 80.53 (s, 1 C) 126.25 (s, 1 C) 128.01 (s, 2 C) 129.19 (s, 2 C);

MS ES+ *m/z* = 357.248 $[M+H]^+$, 379.235 $[M+Na]^+$ ($C_{17}H_{20}N_6O_3$).

Cyclo-1,4-Triazol[-β-Ala-Phe-Pra]-NH_2 (17):

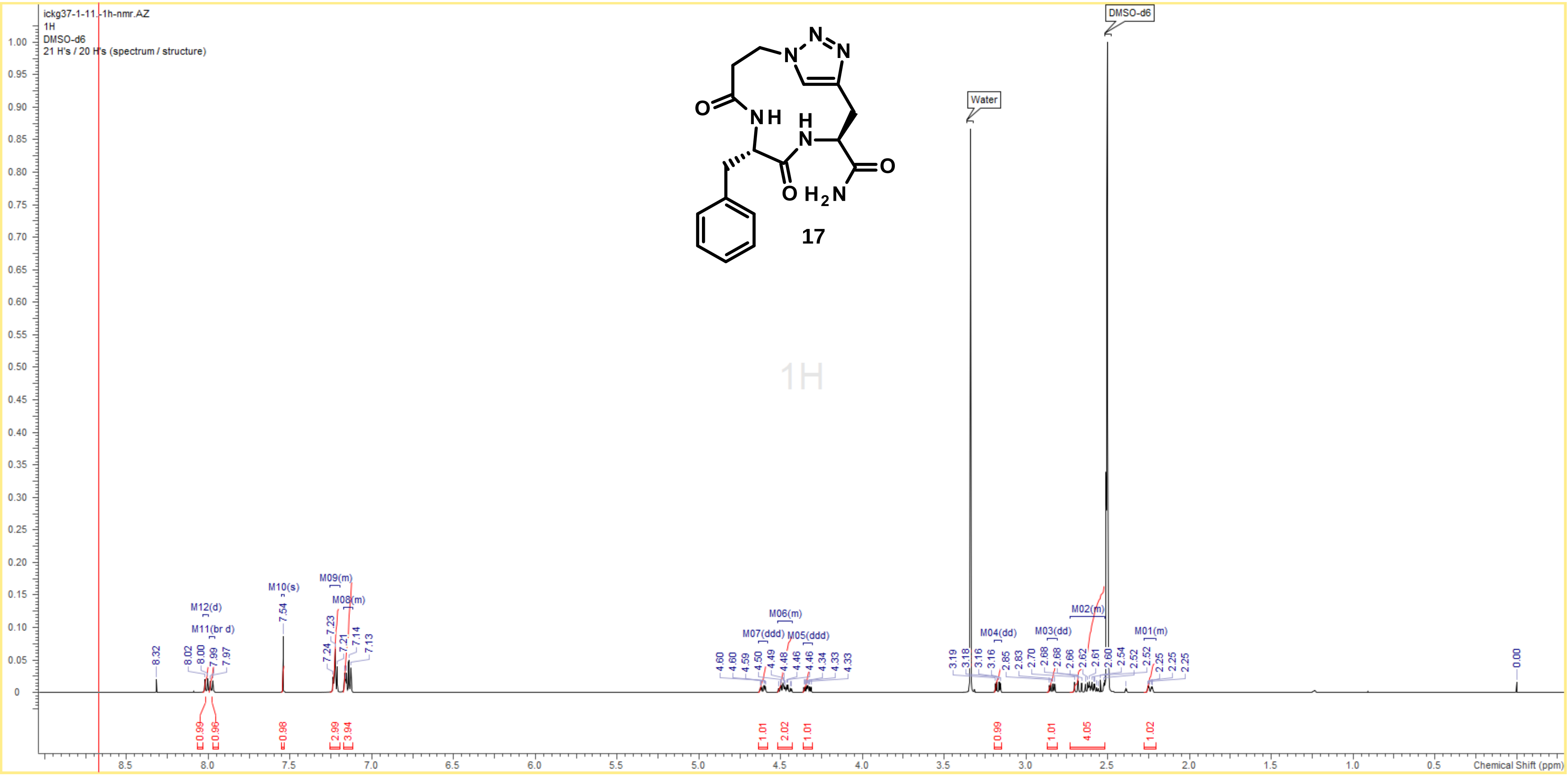

*Figure 3.124: ^{1}H-NMR of Cyclo-1,4-Triazol[-β-Ala-Phe-Pra]-NH_2 **(17)***

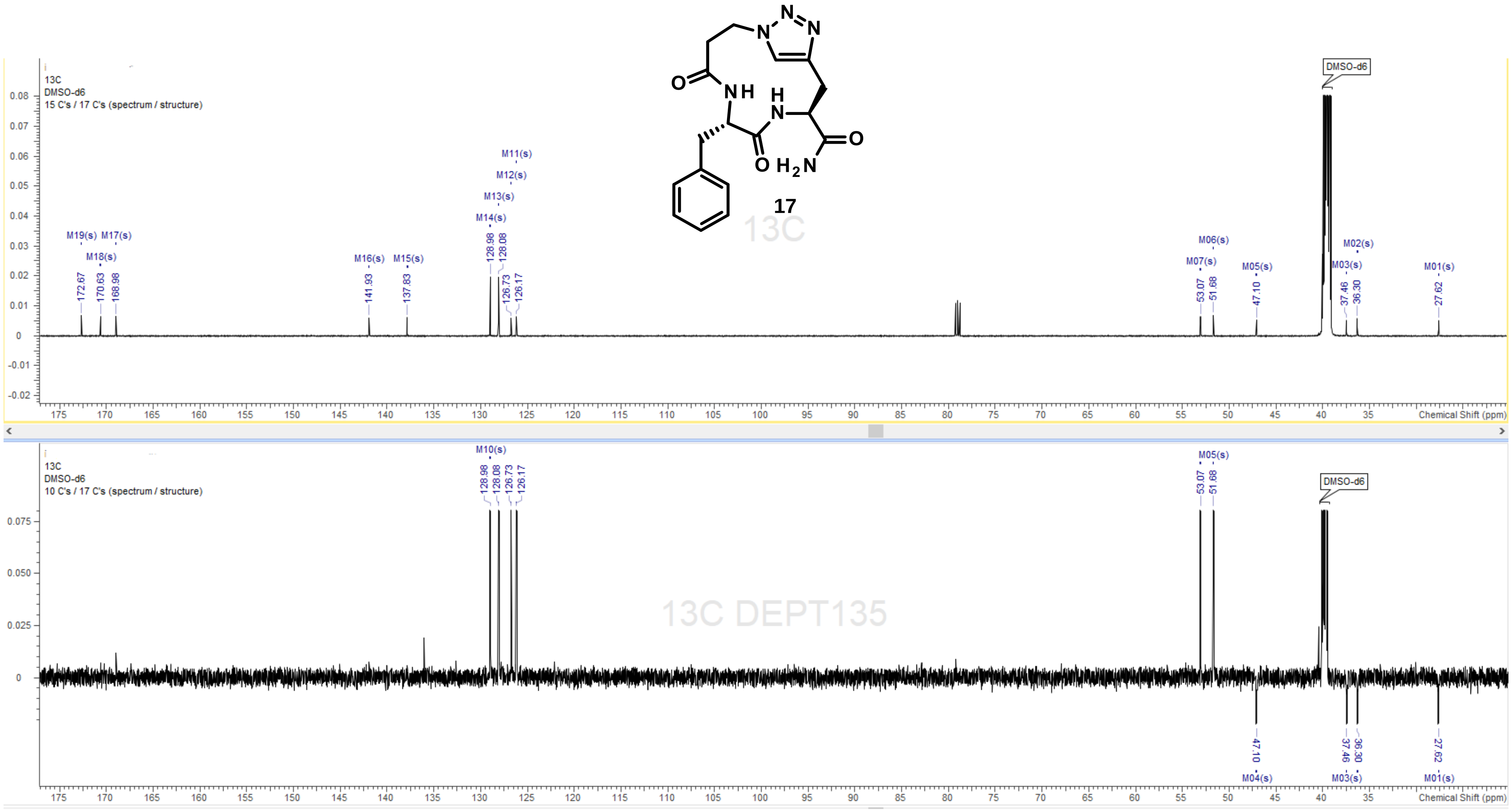

Figure 3.125: ^{13}C-NMR-BB (top) and ^{13}C-NMR-DEPT (bottom) of Cyclo-1,4-Triazol[-β-Ala-Phe-Pra]-NH$_2$ ***(17)***

Azido-β-Ala-Phe-Pra-NH_2 **(13)** (20 mg, 112 µmol) was added to ethanol (3.6 ml). To this suspension was added $CuSO_4 \times 5H_2O$ (0.2 eq, 112 µl, 200 mM aq.) and L-sodium ascorbate (673 µl, 0.3 eq, 50 mM aq.). The reaction was stirred for 16 h at room temperature and resulted in formation of a precipitate. The reaction was controlled by LC-MS. The precipitate was filtered, washed with water and methanol. The filtrate was dissolved in DMSO and was purified with preparative HPLC. Yield: 6 mg white solid (16.8 µmol, 15.00%).

^{1}H NMR (600 MHz, DMSO-*d*$_6$) δ ppm 2.20 - 2.28 (m, 1 H), 2.52 - 2.73 (m, 4 H), 2.84 (dd, *J* = 13.73, 6.48 Hz, 1 H), 3.17 (dd, *J* = 13.92, 4.77 Hz, 1 H), 4.34 (ddd, *J* = 12.21, 9.54, 4.96 Hz, 1 H), 4.43 - 4.52 (m, 2 H), 4.61 (ddd, *J* = 13.45, 5.44, 1.34 Hz, 1 H), 7.12 - 7.18 (m, 4 H), 7.19 - 7.25 (m, 3 H), 7.54 (s, 1 H), 7.98 (br d, *J* = 9.54 Hz, 1 H), 8.01 (d, *J* = 9.92 Hz, 1 H);

^{13}C NMR-BB (151 MHz, DMSO-*d*$_6$) δ ppm 27.62 (s, 1 C), 36.30 (s, 1 C), 37.46 (s, 1 C), 47.10 (s, 1 C), 51.68 (s, 1 C), 53.07 (s, 1 C), 126.17 (s, 1 C), 126.73 (s, 1 C), 128.08 (s, 2 C), 128.98 (s, 2 C), 137.83 (s, 1 C), 141.93 (s, 1 C), 168.98 (s, 1 C), 170.63 (s, 1 C), 172.67 (s, 1 C);

^{13}C NMR-DEPT (151 MHz, DMSO-*d*$_6$) δ ppm 27.62 (s, 1 C), 36.30 (s, 1 C), 37.46 (s, 1 C), 47.10 (s, 1 C), 51.68 (s, 1 C), 53.07 (s, 1 C), 126.17 (s, 1 C), 126.73 (s, 1 C), 128.08 (s, 1 C), 128.98 (s, 1 C);

MS ES+ *m/z* = 357.237 $[M+H]^+$ ($C_{17}H_{22}N_6O_3$).

3.5.3 Cyclo-1,4-Triazol[-2-Abz-Phe-Pra]-OH (18)

H-2-Abz-Pra-OH.TFA (10):

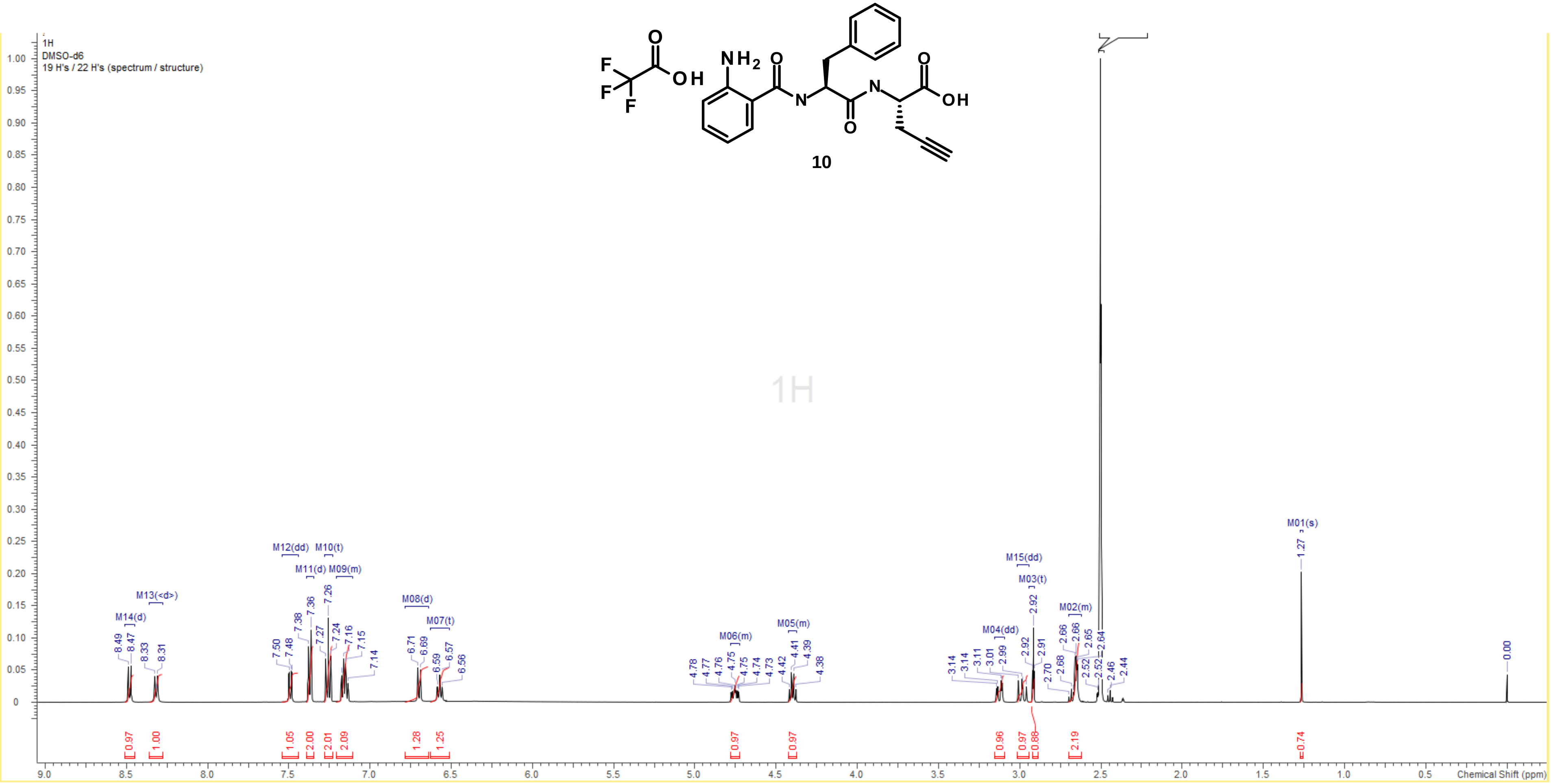

*Figure 3.126: ^{1}H-NMR of H-2-Abz-Pra-OH.TFA **(10)***

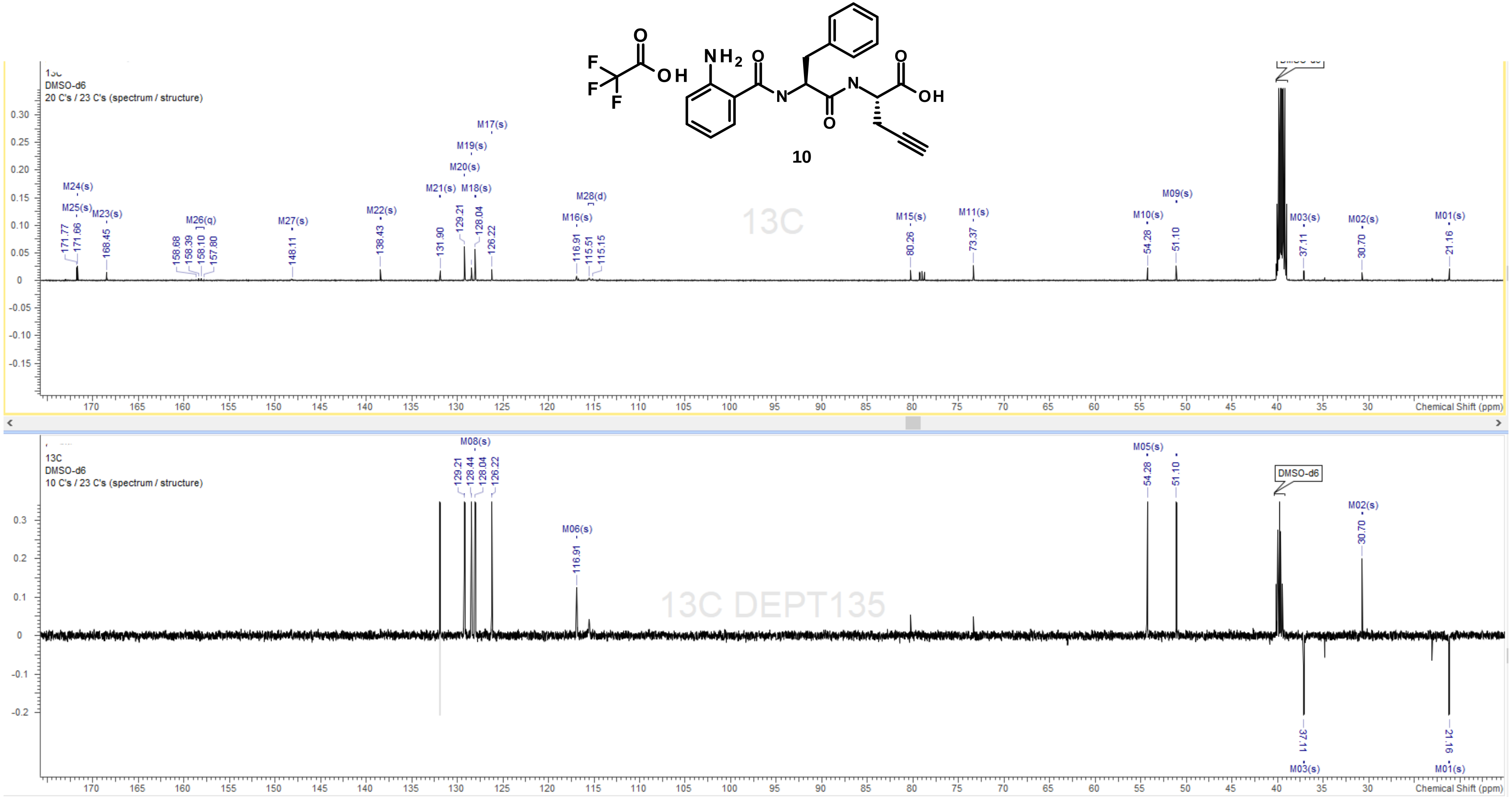

Figure 3.127: ^{13}C-NMR-BB (top) and ^{13}C-NMR-DEPT (bottom) of H-2-Abz-Pra-OH.TFA ***(10)***

^{1}H NMR (500 MHz, DMSO-d_6) δ ppm 1.27 (s, 1 H), 2.62 - 2.70 (m, 2 H), 2.92 (t, *J* = 2.54 Hz, 1 H), 2.98 (dd, *J* = 13.67, 11.13Hz, 1 H), 3.13 (dd, *J* = 13.83, 3.66 Hz, 1 H), 4.37 - 4.42 (m, 1 H), 4.72 - 4.78 (m, 1 H), 6.57 (t, *J* = 7.31 Hz, 1 H), 6.70 (d, *J* = 7.95 Hz, 1 H), 7.11 - 7.21 (m, 2 H), 7.26 (t, *J* = 7.47 Hz, 2 H), 7.37 (d, *J* = 6.99 Hz, 2 H), 7.49 (dd, *J* = 7.95, 1.27 Hz, 1 H), 8.32 (d, *J* = 8.06 Hz, 1 H), 8.48 (d, *J* = 7.95 Hz, 1 H);

^{13}C NMR-BB (126 MHz, DMSO-d_6) δ ppm 21.16 (s, 1 C), 30.70 (s, 1 C), 37.11 (s, 1 C), 51.10 (s, 1 C), 54.28 (s, 1 C), 73.37 (s, 1 C), 80.26 (s, 1 C), 115.33 (d, *J* = 45.56 Hz, 1 C), 116.91 (s, 1 C), 126.22 (s, 1 C), 128.04 (s, 2 C), 128.44 (s, 1 C), 129.21 (s, 2 C), 131.90 (s, 1 C), 138.43 (s, 1 C), 148.11 (s, 1 C), 158.24 (q, *J* = 36.91 Hz, 1 C), 168.45 (s, 1 C), 171.66 (s, 1 C), 171.77 (s, 1 C);

^{13}C NMR-DEPT (126 MHz, DMSO-d_6) δ ppm 21.16 (s, 1 C), 30.70 (s, 1 C), 37.11 (s, 1 C), 51.10 (s, 1 C), 54.28 (s, 1 C), 116.91 (s, 1 C), 126.22 (s, 1 C), 128.04 (s, 2 C), 128.44 (s, 1 C), 129.21 (s, 2 C), 131.90 (s, 1 C);

MS ES+ *m/z* = 380.241 [*M*+H]$^+$ ($C_{21}H_{21}N_3O_4$).

Azido-2-Abz-Phe-Pra-OH (14):

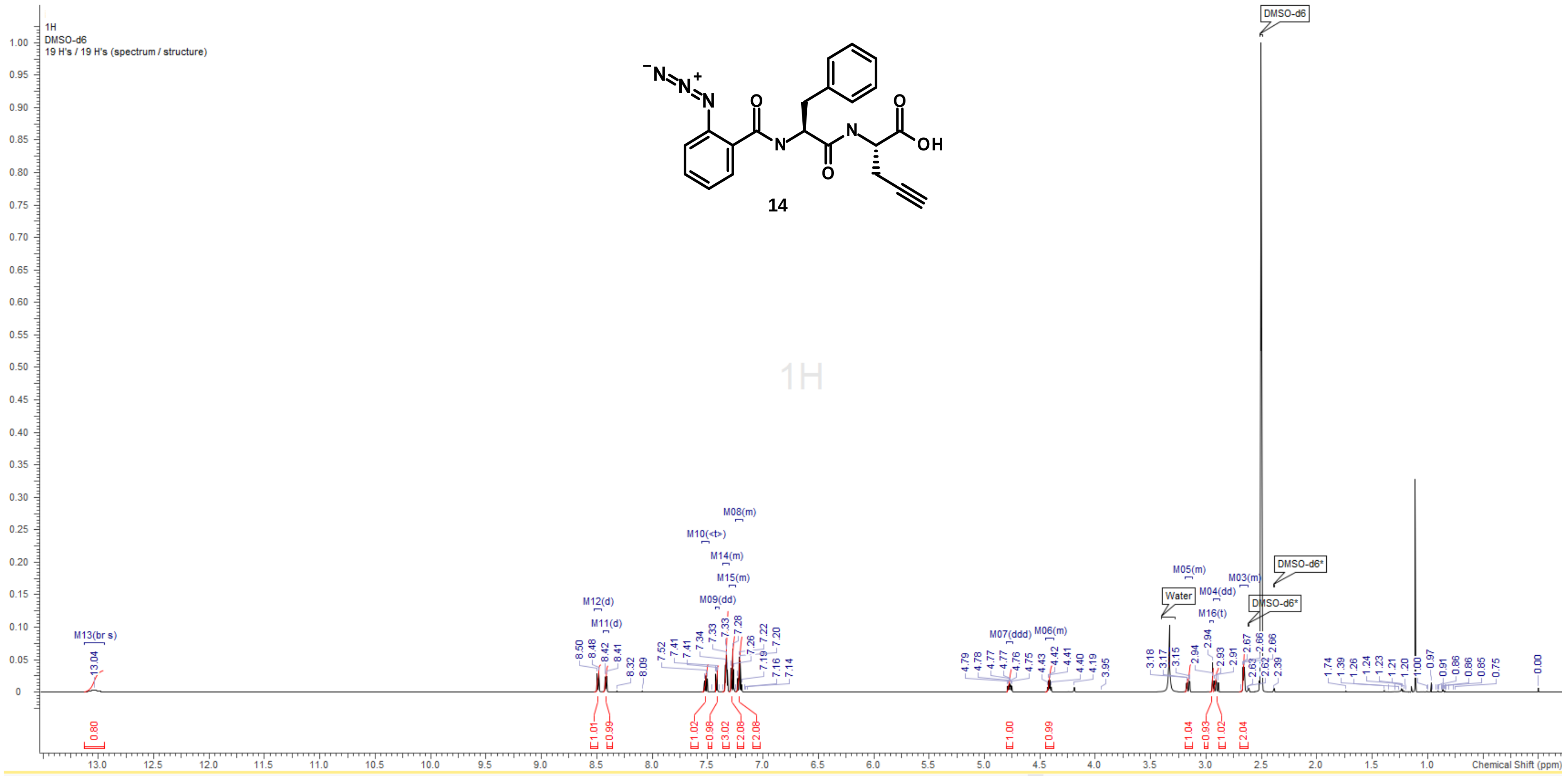

*Figure 3.128: ^{1}H-NMR of Azido-2-Abz-Phe-Pra-OH **(14)***

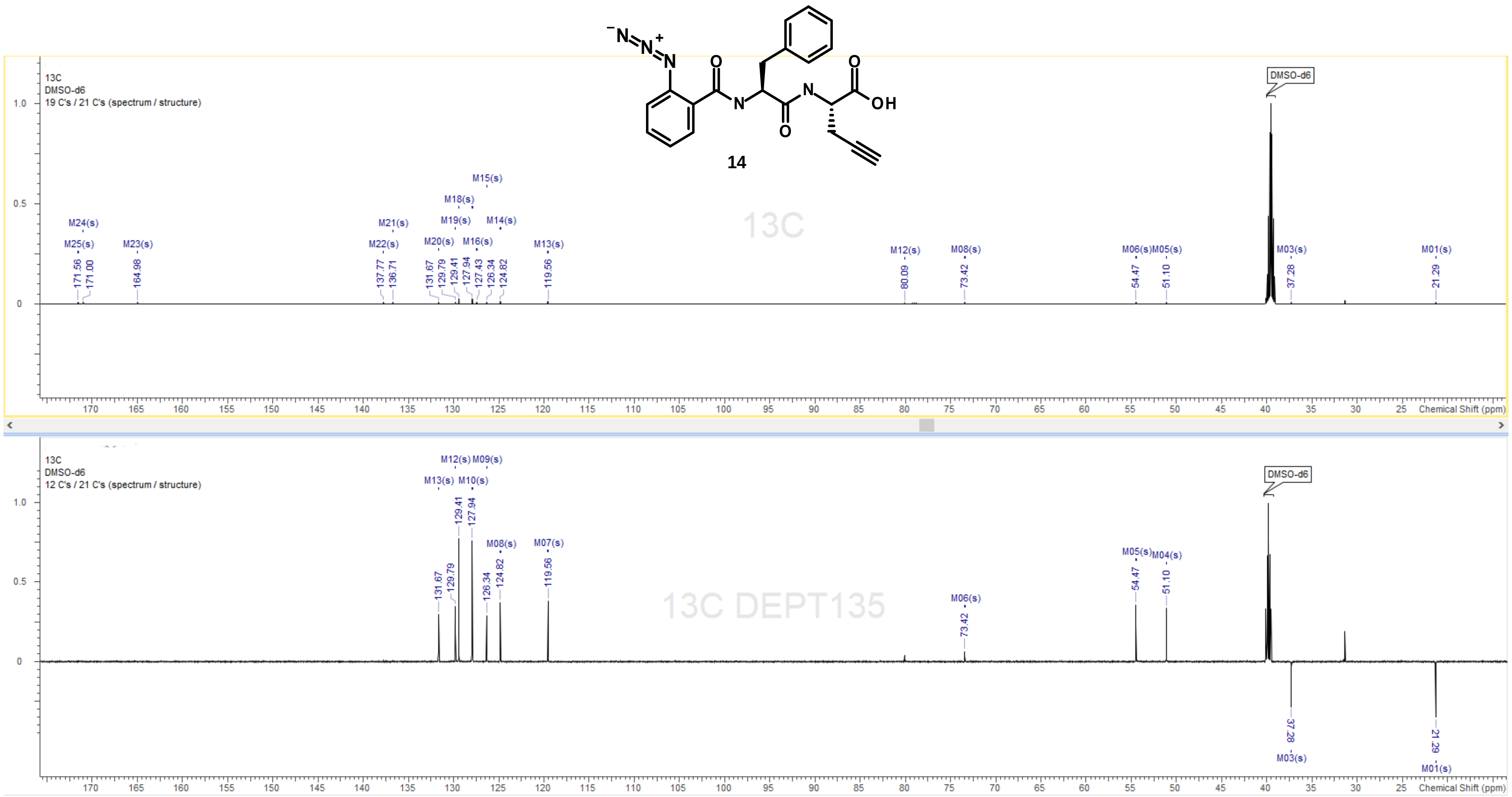

Figure 3.129: ^{13}C-NMR-BB (top) and ^{13}C-NMR-DEPT (bottom) of Azido-2-Abz-Phe-Pra-OH ***(14)***

H-2-Abz-Phe-Pra-OH.TFA **(10)** (1 eq, 50 mg, 101 µmol) was dissolved in $NaHCO_3$ (7 eq, 3.55 ml, 200 mM aq.) and ISA×H_2SO_4 (2.5 eq, 506 µl, 500 mM aq.) and copper(II)sulfate pentahydrate (0.01 eq, 56.2 µl, 20 mM aq.) was added. The reaction was stirred for 16 h at room temperature and resulted in formation of a precipitate. The reaction was controlled by LC-MS. The precipitate was filtered, washed with water and dissolved in methanol. The solution was concentrated, dissolved in DMSO and was purified with preparative HPLC. Yield: 34.2 mg white solid (84.4 µmol, 83.25%)

^{1}H NMR (600 MHz, DMSO-*d*$_6$) δ ppm 2.62 - 2.70 (m, 2H), 2.91 (dd, *J*=13.92, 10.11Hz, 1H), 2.94 (t, *J*=2.48Hz, 1H), 3.13 - 3.19 (m, 1H), 4.38 - 4.45 (m, 1H), 4.77 (ddd, *J*=10.11, 8.39, 4.01Hz, 1H), 7.18 - 7.24 (m, 2H), 7.25 - 7.31 (m, 2H), 7.31 - 7.37 (m, 3H), 7.42 (dd, *J*=7.63, 1.53Hz, 1H), 7.51 (t, *J*=7.70Hz, 1H), 8.42 (d, *J*=7.63Hz, 1H), 8.49 (d, *J*=8.39Hz, 1H), 13.04 (br s, 1H);

^{13}C NMR-BB (151 MHz, DMSO-*d*$_6$) δ ppm 21.29 (s, 1C), 37.28 (s, 1C), 51.10 (s, 1C), 54.47 (s, 1C), 73.42 (s, 1C), 80.09 (s, 1C), 119.56 (s, 1C), 124.82 (s, 1C), 126.34 (s, 1C), 127.43 (s, 1C), 127.94 (s, 2C), 129.41 (s, 2C), 129.79 (s, 1C), 131.67 (s, 1C), 136.71 (s, 1C), 137.77 (s, 1C), 164.98 (s, 1C), 171.00 (s, 1C), 171.56 (s, 1C);

^{13}C NMR (151 MHz, DMSO-*d*$_6$) δ ppm 21.29 (s, 1C), 37.28 (s, 1C), 51.10 (s, 1C), 54.47 (s, 1C), 73.42 (s, 1C), 119.56 (s, 1C), 124.82 (s, 1C), 126.34 (s, 1C), 127.94 (s, 1C), 129.41 (s, 1C), 129.79 (s, 1C), 131.67 (s, 1C);

MS ES+ *m/z*=406.243 $[M+H]^+$ *m/z*=428.229 $[M+Na]^+$ ($C_{21}H_{19}N_5O_4$).

Cyclo-1,4-Triazol[-2-Abz-Phe-Pra]-OH (18):

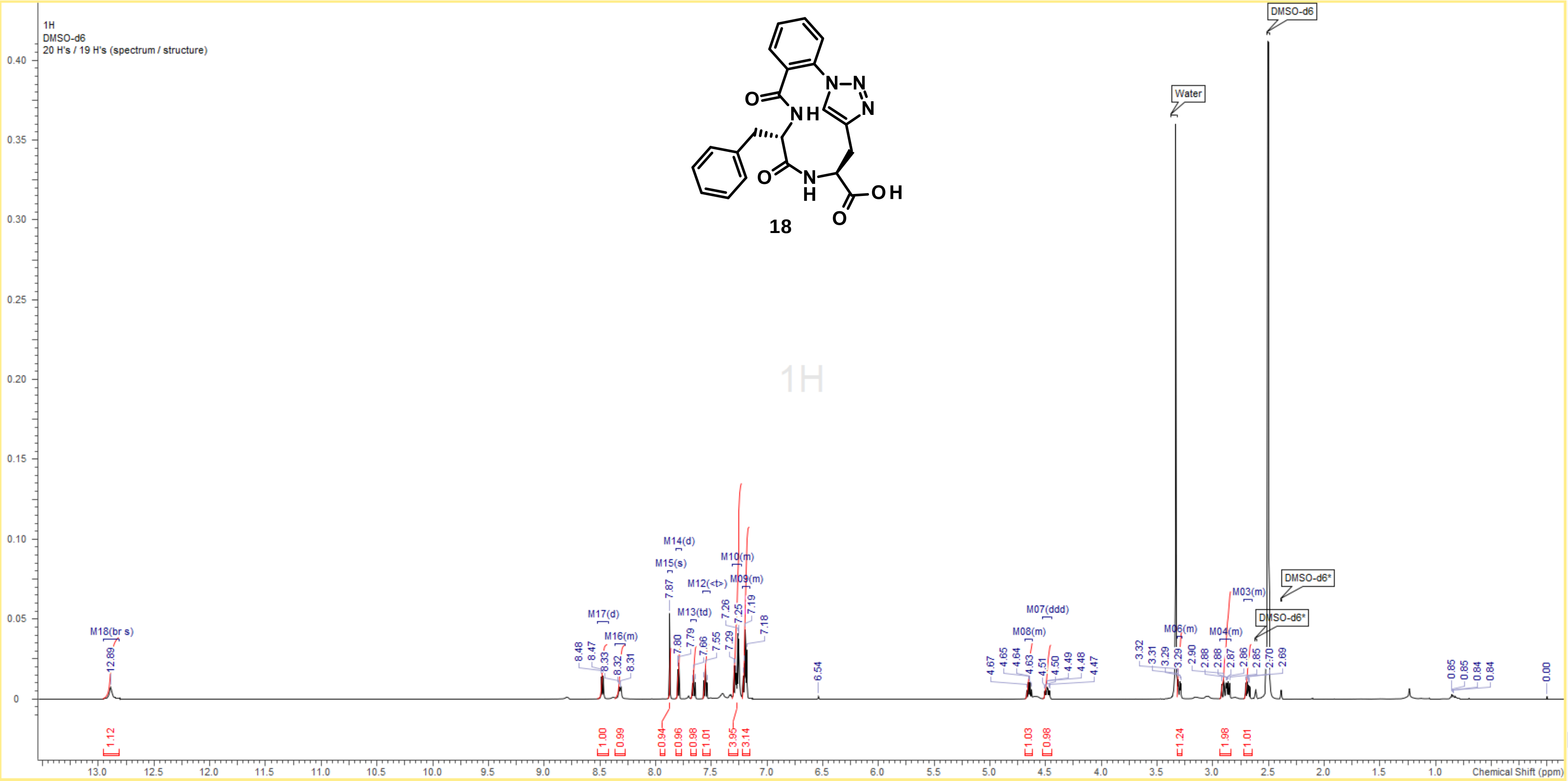

*Figure 3.130: ¹H-NMR of Cyclo-1,4-Triazol[-2-Abz-Phe-Pra]-OH **(18)***

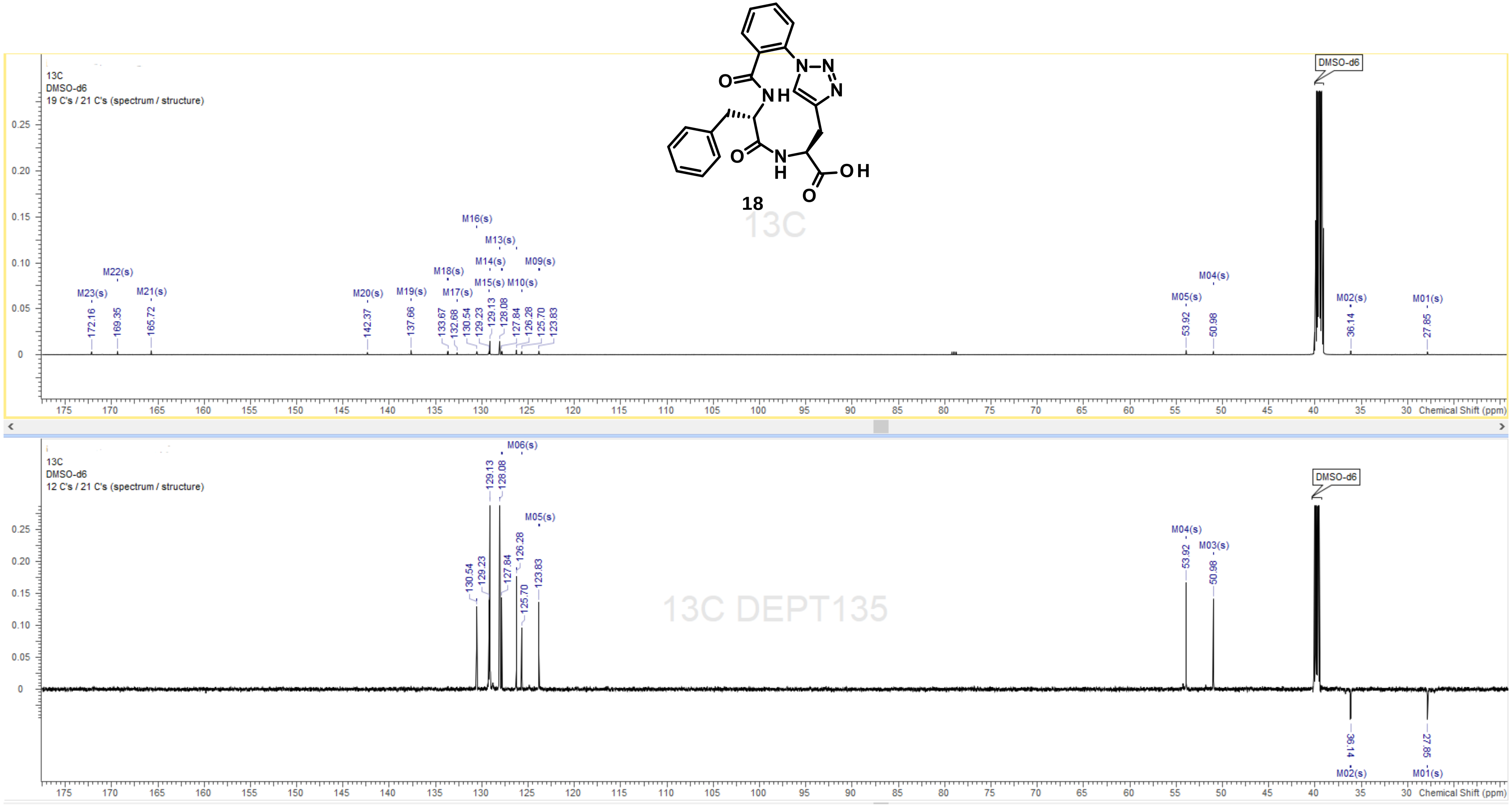

Figure 3.131: ^{13}C-NMR-BB (top) and ^{13}C-NMR-DEPT (bottom) of Cyclo-1,4-Triazol[-2-Abz-Phe-Pra]-OH ***(18)***

Azido-2-Abz-Phe-Pra-OH **(14)** (20 mg, 49.3 µmol) was added to ethanol (1.6 ml). To this suspension was added $CuSO_4 \times 5H_2O$ (0.2 eq, 49. µl, 200 mM aq.) and L-sodium ascorbate (296 µl, 0.3 eq, 50 mM aq.). The reaction was stirred for 16 h at room temperature and resulted in formation of a precipitate. The reaction was controlled by LC-MS. The precipitate was filtered, washed with 0.5 M HCl and methanol and dried. The filtrate was clean product **(18)**. Yield: 6.7 mg white solid (16.5 µmol, 33.50%).

^{1}H NMR (600 MHz, DMSO-*d*$_6$) δ ppm 2.65 - 2.72 (m, 1 H), 2.83 - 2.94 (m, 2 H), 3.27 - 3.32 (m, 1 H), 4.49 (ddd, *J* = 11.83, 9.73, 4.77 Hz, 1 H), 4.62 - 4.68 (m, 1 H), 7.15 - 7.22 (m, 3 H), 7.22 - 7.31 (m, 4 H), 7.55 (t, *J* = 7.64 Hz, 1 H), 7.66 (td, *J* = 7.82, 1.53 Hz, 1 H), 7.80 (d, *J* = 7.25 Hz, 1 H), 7.87 (s, 1 H), 8.27 - 8.36 (m, 1 H), 8.48 (d, *J* = 9.92 Hz, 1 H), 12.89 (br s, 1 H);

^{13}C NMR-BB (151 MHz, DMSO-*d*$_6$) δ ppm 27.85 (s, 1 C), 36.14 (s, 1 C), 50.98 (s, 1 C), 53.92 (s, 1 C), 123.83 (s, 1 C), 125.70 (s, 1 C), 126.28 (s, 1 C), 127.84 (s, 1 C), 128.08 (s, 2 C), 129.13 (s, 2 C), 129.23 (s, 1 C), 130.54 (s, 1 C), 132.68 (s, 1 C), 133.67 (s, 1 C), 137.66 (s, 1 C), 142.37 (s, 1 C), 165.72 (s, 1 C), 169.35 (s, 1 C), 172.16 (s, 1 C);

^{13}C NMR-DEPT (151 MHz, DMSO-*d*$_6$) δ ppm 27.85 (s, 1 C), 36.14 (s, 1 C), 50.98 (s, 1 C), 53.92 (s, 1 C), 123.83 (s, 1 C), 125.70 (s, 1 C), 126.28 (s, 1 C), 127.84 (s, 1 C), 128.08 (s, 2 C), 129.13 (s, 2 C), 129.23 (s, 1 C), 130.54 (s, 1 C);

MS ES+ *m/z* = 406.244 [*M*+H]$^+$ ($C_{21}H_{19}N_5O_4$).

3.5.4 Cyclo-1,4-Triazol-[-3-Abz-Phe-Pra]-OH (19):

H-3-Abz-Pra-OH.TFA (11):

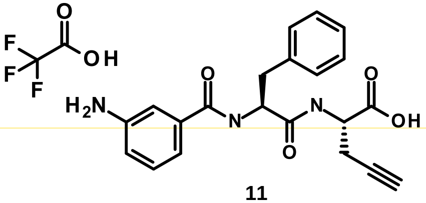

11

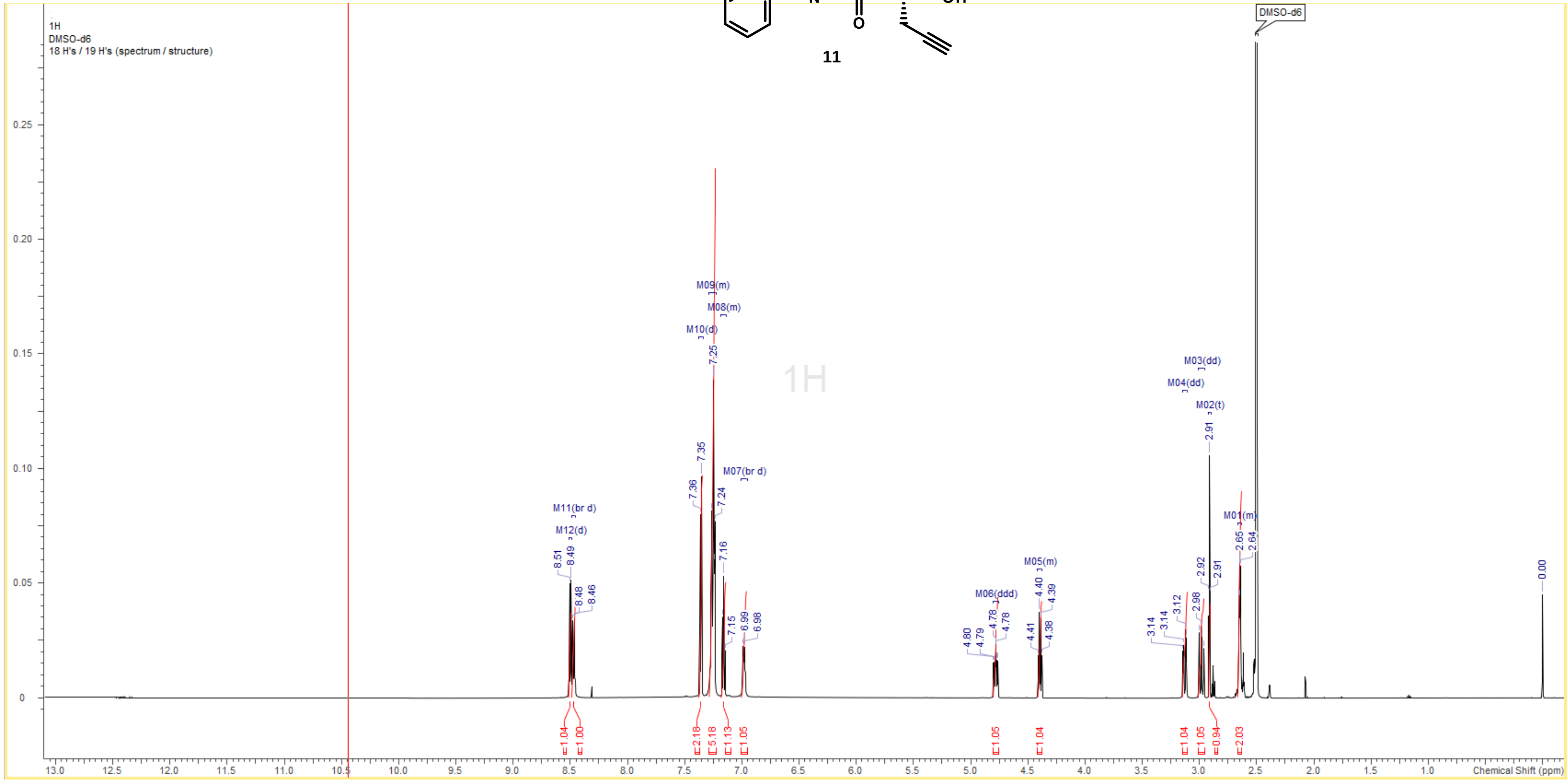

*Figure 3.132: ¹H-NMR of H-3-Abz-Pra-OH.TFA **(11)***

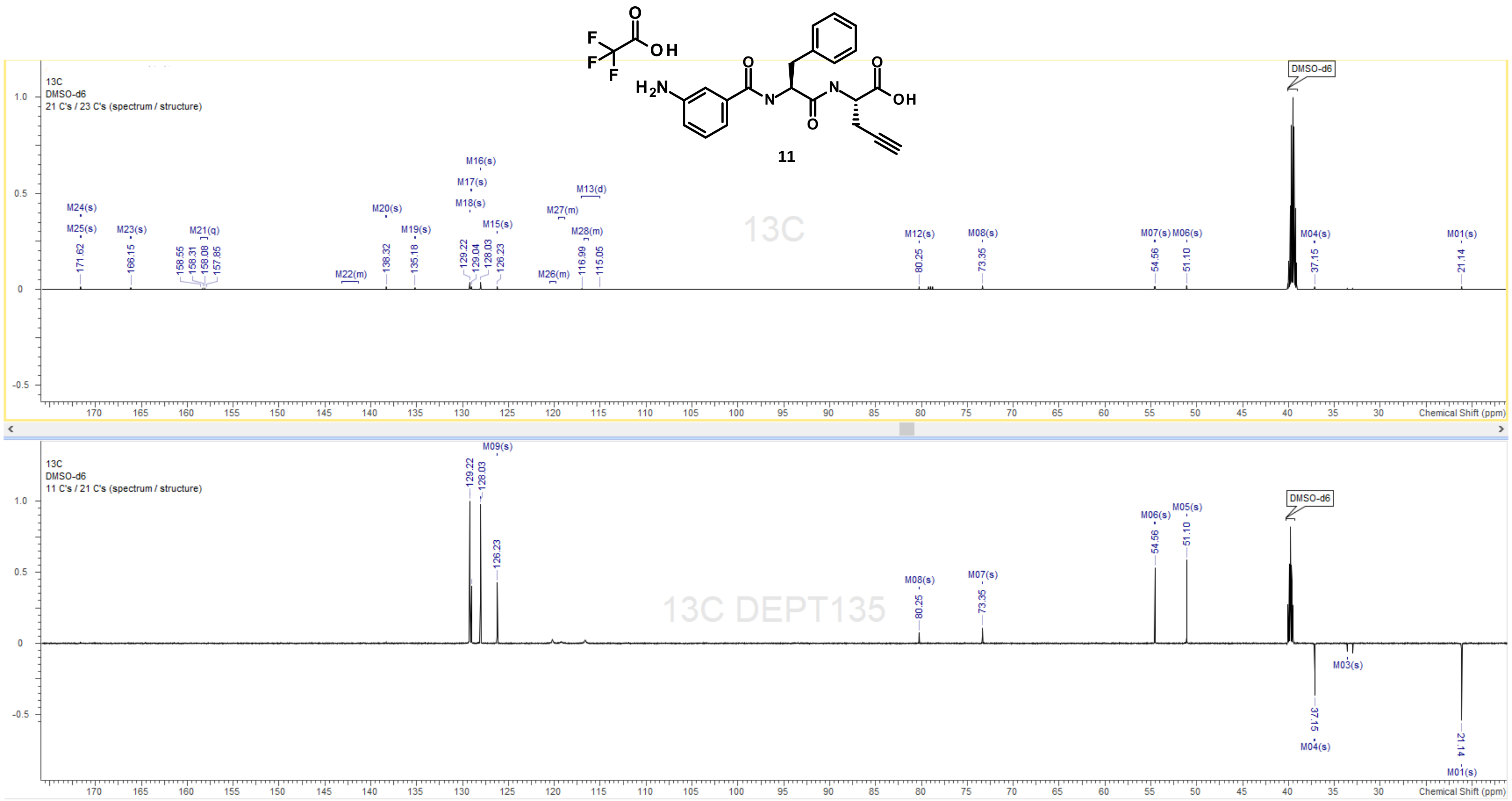

Figure 3.133: ^{13}C-NMR-BB (top) and ^{13}C-NMR-DEPT (bottom) of H-3-Abz-Pra-OH.TFA ***(11)***

^{1}H NMR (600 MHz, DMSO-*d_6*) δ ppm 2.63 - 2.67 (m, 2 H), 2.91 (t, *J* = 2.67 Hz, 1 H), 2.98 (dd, *J* = 13.73, 10.68 Hz, 1 H), 3.13 (dd, *J* = 13.73, 3.81 Hz, 1 H), 4.37 - 4.41 (m, 1 H), 4.78 (ddd, *J* = 10.97, 8.68, 3.62 Hz, 1 H), 6.98 (br d, *J* = 7.25 Hz, 1 H), 7.13 - 7.18 (m, 1 H), 7.23 - 7.29 (m, 5 H), 7.36 (d, *J* = 7.25 Hz, 2 H), 8.47 (br d, *J* = 8.77 Hz, 1 H), 8.50 (d, *J* = 8.01 Hz, 1 H);

^{13}C NMR-BB (151 MHz, DMSO-*d_6*) δ ppm 21.14 (s, 1 C), 37.15 (s, 1 C), 40.08 (s, 1 C), 51.10 (s, 1 C), 54.56 (s, 1 C), 73.35 (s, 1 C), 80.25 (s, 1 C), 116.02 (d, *J* = 293.73 Hz, 1 C), 116.30 - 116.79 (m, 1 C), 118.88 - 119.52 (m, 1 C), 119.81 - 120.53 (m, 1 C), 126.23 (s, 1 C), 128.03 (s, 2 C), 129.04 (s, 1 C), 129.22 (s, 2 C), 135.18 (s, 1 C), 138.32 (s, 1 C), 141.31 - 143.16 (m, 1 C), 158.20 (q, *J* = 35.18 Hz, 1 C), 166.15 (s, 1 C), 171.58 (s, 1 C), 171.62 (s, 1 C);

^{13}C NMR-DEPT (151 MHz, DMSO-*d_6*) δ ppm 21.14 (s, 1 C), 33.01 (s, 1 C), 33.60 (s, 1 C), 37.15 (s, 1 C), 51.10 (s, 1 C), 54.56 (s, 1 C), 73.35 (s, 1 C), 80.25 (s, 1 C), 116.37 - 116.90 (m, 1 C), 118.99 - 119.47 (m, 1 C), 120.00 - 120.39 (m, 1 C), 126.23 (s, 1 C), 128.03 (s, 2 C), 129.04 (s, 1 C), 129.22 (s, 2 C);

MS ES+ *m/z* = 380.241 [*M*+H]$^+$ ($C_{21}H_{21}N_3O_4$).

Azido-3-Abz-Phe-Pra-OH (15):

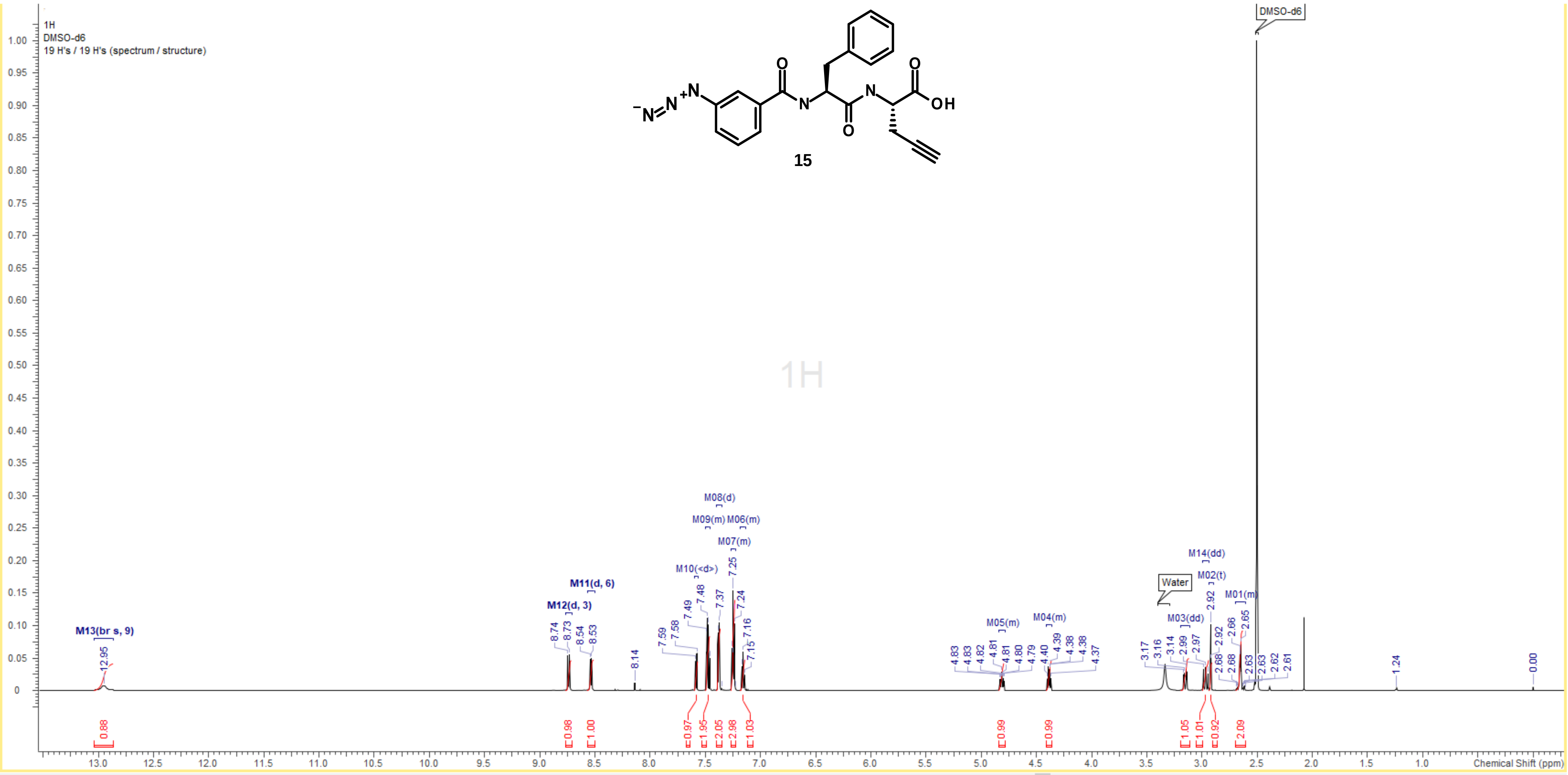

*Figure 3.134: ^{1}H-NMR of Azido-3-Abz-Phe-Pra-OH **(15)***

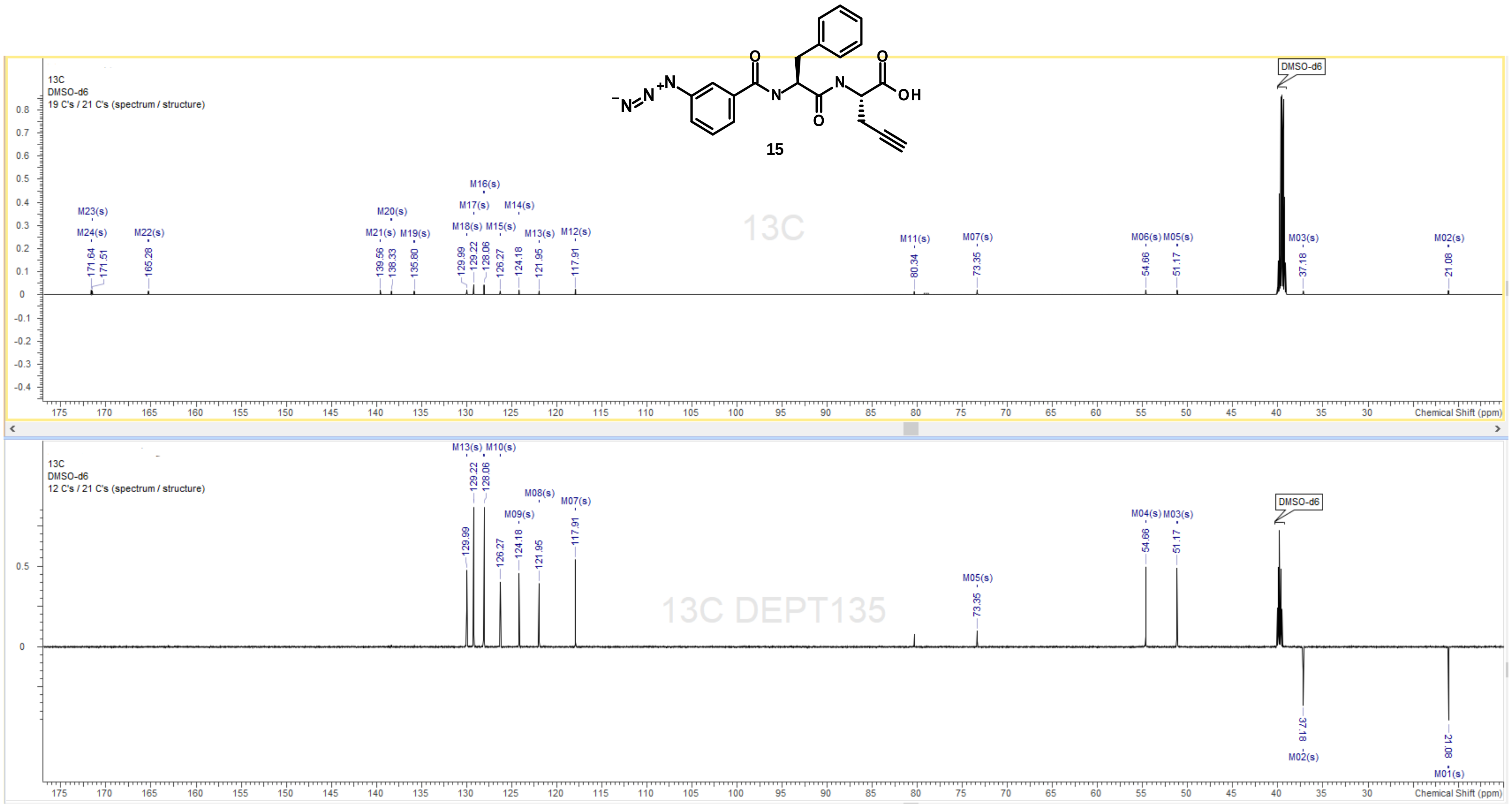

Figure 3.135: ^{13}C-NMR-BB (top) and ^{13}C-NMR-DEPT (bottom) of Azido-3-Abz-Phe-Pra-OH ***(15)***

H-3-Abz-Phe-Pra-OH**.**TFA **(11)** (1 eq, 300 mg, 608 µmol) was dissolved in $NaHCO_3$ (7 eq, 21.2 ml, 200 mM aq.) and ISA×H_2SO_4 (2.5 eq, 3.04 ml, 500 mM aq.) and copper(II)sulfate pentahydrate (0.01 eq, 304µl, 20 mM aq.) was added. The reaction was stirred for 16 h at room temperature and resulted in formation of a precipitate. The reaction was controlled by LC-MS. The precipitate was filtered and was purified with preparative HPLC. Yield **(15)**: 164 mg white solid (384 µmol, 63.21%)

^{1}H NMR (600 MHz, DMSO-*d*$_6$) δ ppm 2.61 - 2.69 (m, 2 H), 2.92 (t, *J* = 2.48 Hz, 1 H), 2.97 (dd, *J* = 13.54, 11.25 Hz, 1 H), 3.15 (dd, *J* = 13.73, 3.43 Hz, 1 H), 4.36 - 4.41 (m, 1 H), 4.79 - 4.84 (m, 1 H), 7.13 - 7.19 (m, 1 H), 7.22 - 7.27 (m, 3 H), 7.38 (d, *J* = 7.25 Hz, 2 H), 7.46 - 7.50 (m, 2 H), 7.58 (d, *J* = 7.59 Hz, 1 H), 8.54 (d, *J* = 7.63 Hz, 1 H), 8.74 (d, *J* = 8.77 Hz, 1 H), 12.95 (br s, 1 H);

^{13}C NMR-BB (151 MHz, DMSO-*d*$_6$) δ ppm 21.08 (s, 1 C), 37.18 (s, 1 C), 51.17 (s, 1 C), 54.66 (s, 1 C), 73.35 (s, 1 C), 80.34 (s, 1 C), 117.91 (s, 1 C), 121.95 (s, 1 C), 124.18 (s, 1 C), 126.27 (s, 1 C), 128.06 (s, 2 C), 129.22 (s, 2 C), 129.99 (s, 1 C), 135.80 (s, 1 C), 138.33 (s, 1 C), 139.56 (s, 1 C), 165.28 (s, 1 C), 171.51 (s, 1 C), 171.64 (s, 1 C);

^{13}C NMR-DEPT (151 MHz, DMSO-*d*$_6$) δ ppm 21.08 (s, 1 C), 37.18 (s, 1 C), 51.17 (s, 1 C), 54.66 (s, 1 C), 73.35 (s, 1 C), 117.91 (s, 1 C), 121.95 (s, 1 C), 124.18 (s, 1 C), 126.27 (s, 1 C), 128.06 (s, 2 C), 129.22 (s, 2 C), 129.99 (s, 1 C);

MS ES+ *m/z* = 406.243 $[M+H]^+$ *m/z* = 428.229 $[M+Na]^+$ ($C_{21}H_{19}N_5O_4$).

Cyclo-1,4-Triazol-[-3-Abz-Phe-Pra]-OH (19):

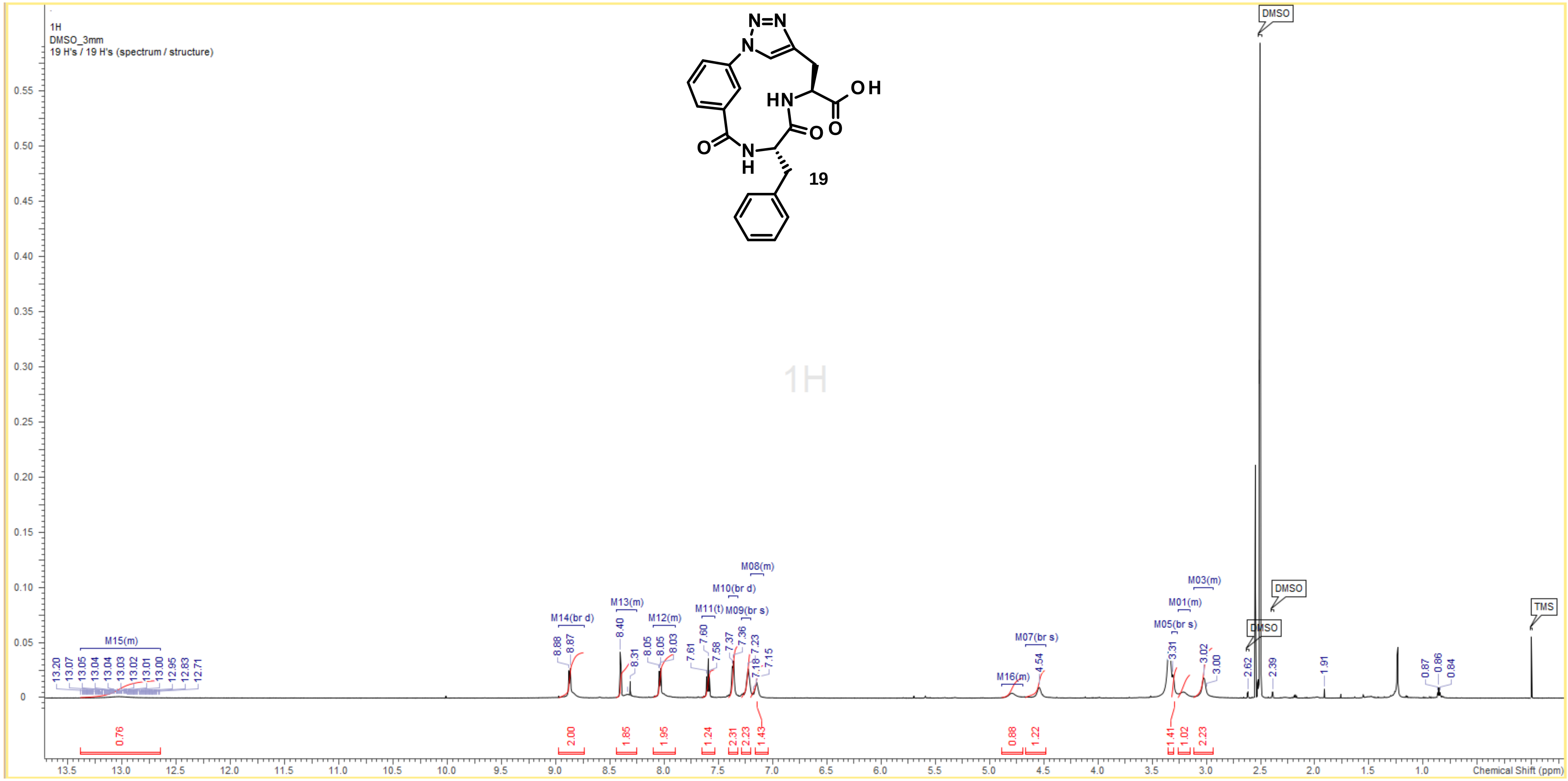

Figure 3.136: ^{1}H-NMR of Cyclo-1,4-Triazol-[-3-Abz-Phe-Pra]-OH

Azido-3-Abz-Phe-Pra-OH **(15)** (20 mg, 49.3 µmol) was added to DMSO (1 ml) and Borate buffer (1 ml, 500 mM), added $CuSO_4 \times 5H_2O$ (1 eq, 247 µl, 200 mM aq.), L-sodium ascorbate (247 µl, 1 eq, 50 mM aq.) and TBTA (247µl, 1eq, 200mM in DMSO). The reaction was stirred for 16 h at room temperature and resulted in formation of a precipitate. The reaction was controlled by LC-MS. The precipitate was filtered, washed with 0,5 M HCl and DMSO. The mother liquid was purified with preparative HPLC. Yield: 2 mg white solid (4.93 µmol, 10%).

^{1}H NMR (600 MHz, DMSO-*d*$_6$) δ ppm 2.94 - 3.12 (m, 2 H) 3.15 - 3.26 (m, 1 H) 3.31 (br s, 1 H) 4.54 (br s, 1 H) 4.69 - 4.88 (m, 1 H) 7.08 - 7.20 (m, 1 H) 7.23 (br s, 2 H) 7.37 (br d, *J* = 6.10 Hz, 2 H) 7.60 (t, *J* = 8.01 Hz, 1 H) 7.90 - 8.10 (m, 2 H) 8.25 - 8.44 (m, 2 H) 8.87 (br d, *J* = 7.63 Hz, 2 H) 12.65 - 13.38 (m, 1 H);

MS ES+ *m/z* = 406.262 [*M*+H]$^+$, 811.513 [2*M*+H]$^+$ ($C_{21}H_{19}N_5O_4$).
